# Engineering of Baeyer-Villiger monooxygenase-based *Escherichia coli* biocatalyst for large scale biotransformation of ricinoleic acid into (*Z*)-11-(heptanoyloxy)undec-9-enoic acid

**DOI:** 10.1038/srep28223

**Published:** 2016-06-17

**Authors:** Joo-Hyun Seo, Hwan-Hee Kim, Eun-Yeong Jeon, Young-Ha Song, Chul-Soo Shin, Jin-Byung Park

**Affiliations:** 1 Department of Food Science and Engineering, Ewha Womans University, Seoul 120-750, Republic of Korea; 2 AP Technology, Suwon, Kyunggi 443-702, Republic of Korea

## Abstract

Baeyer-Villiger monooxygenases (BVMOs) are able to catalyze regiospecific Baeyer-Villiger oxygenation of a variety of cyclic and linear ketones to generate the corresponding lactones and esters, respectively. However, the enzymes are usually difficult to express in a functional form in microbial cells and are rather unstable under process conditions hindering their large-scale applications. Thereby, we investigated engineering of the BVMO from *Pseudomonas putida* KT2440 and the gene expression system to improve its activity and stability for large-scale biotransformation of ricinoleic acid (**1**) into the ester (i.e., (*Z*)-11-(heptanoyloxy)undec-9-enoic acid) (**3**), which can be hydrolyzed into 11-hydroxyundec-9-enoic acid (**5**) (i.e., a precursor of polyamide-11) and *n*-heptanoic acid (**4**). The polyionic tag-based fusion engineering of the BVMO and the use of a synthetic promoter for constitutive enzyme expression allowed the recombinant *Escherichia coli* expressing the BVMO and the secondary alcohol dehydrogenase of *Micrococcus luteus* to produce the ester (**3**) to 85 mM (26.6 g/L) within 5 h. The 5 L scale biotransformation process was then successfully scaled up to a 70 L bioreactor; **3** was produced to over 70 mM (21.9 g/L) in the culture medium 6 h after biotransformation. This study demonstrated that the BVMO-based whole-cell reactions can be applied for large-scale biotransformations.

Baeyer-Villiger monooxygenases (BVMOs, EC 1.14.13.X) catalyze a variety of oxygenations including the nucleophilic oxygenation of ketones (Baeyer-Villiger oxidation) and boron as well as the electrophilic oxygenation of various heteroatoms (e.g., sulfur, selenium, nitrogen and phosphorous)^1^,^2^,^3^,^4^,^5^. Thereby, the BVMOs are involved in diverse metabolisms such as steroid degradation, metabolism of terpenoids, and degradation of linear, cyclic and aromatic ketones. The enzymes are also considered as one of the most important biocatalysts for organic synthesis. For instance, the BVMOs from *Pseudomonas putida* KT2440 (i.e., EthA), *P. fluorescens* DSM50106 (BmoF1), and *Rhodococcus jostii* RHA1(MO16) are able to catalyze regiospecific oxygenation of 12-keto-*cis*-9-octadecenoic acid, 13-keto-*cis*-9-octadecenoic acid, 10-keto-octadecanoic acid, 10-keto-*cis*-12-octadecenoic acid, 13-keto-*cis*-6,9-octadecadienoic acid, and 10-keto-*cis*-6,12-octadecadienoic acid into the corresponding esters, which can be enzymatically converted into ω-hydroxycarboxylic acids, α,ω-dicarboxylic acids, and ω-aminocarboxylic acids^5^,^6^,^7^,^8^,^9^. However, a number of BVMOs including EthA, BmoF1, and MO16 were difficult to be expressed in a functional form in conventional microbial biocatalysts (e.g., *Escherichia coli* and *Saccharomyces cerevisiae*), in particular, under room temperatures^10^,^11^,^12^. Moreover, they are rather unstable under process conditions hindering their large-scale applications.

In order to improve functional expression and structural stability of the oxygenases (e.g., EthA of *P. putida* KT2440)^12^ in microbial cells, a variety of approaches have been explored. Not only the induction conditions for gene expression (e.g., cultivation temperature, inducer type and concentration), but also the gene expression systems including the promoters, ribosome binding sites (RBSs), 5′-untranslated region (5′UTR), and codon usage have been largely investigated to enhance soluble expression of the enzymes^11^,^12^,^13^,^14^. In addition, introduction of molecular chaperones^15^,^16^, the protein fusion with soluble peptides and proteins^17^,^18^, introduction of disulfide bonds^19^,^20^, and other protein engineering methods (e.g., directed evolution)^21^,^22^ have also been intensively examined to increase functional expression and stability of the oxygenases in microbial cells. However, these approaches are not so satisfactory enough for large scale biotransformations.

In this study, the BVMO from *P. putida* KT2440 12 was engineered to enhance its functional expression and stability in *E. coli* BL21(DE3) by assuming that negatively charged residues in the N- or C-terminal are of great importance to thermal stability of proteins. For instance, the number of glutamate residues in the C-terminus of Group II chaperonins was reported to be proportional to protein thermal stability^23^. The polyionic tags (e.g., hexa-glutamate (E6) tag, hexa-lysine (K6) tag) were fused to N-terminal of the BVMO to increase soluble expression and structural stability in *E. coli* under room temperatures, which is important for feasibility to industrial applications. Furthermore, a synthetic promoter was used for inducer free-constitutive expression of the BVMO, which is also critical for large scale biotransformations. Finally, the *E. coli*-based biocatalyst expressing the engineered enzyme by using the constitutive promoter was applied for ricinoleic acid biotransformation at high cell density culture in a 5 L and 70 L scale bioreactor.

## Results

### Polyionic tag-based engineering of BVMO

**Figure 1:**
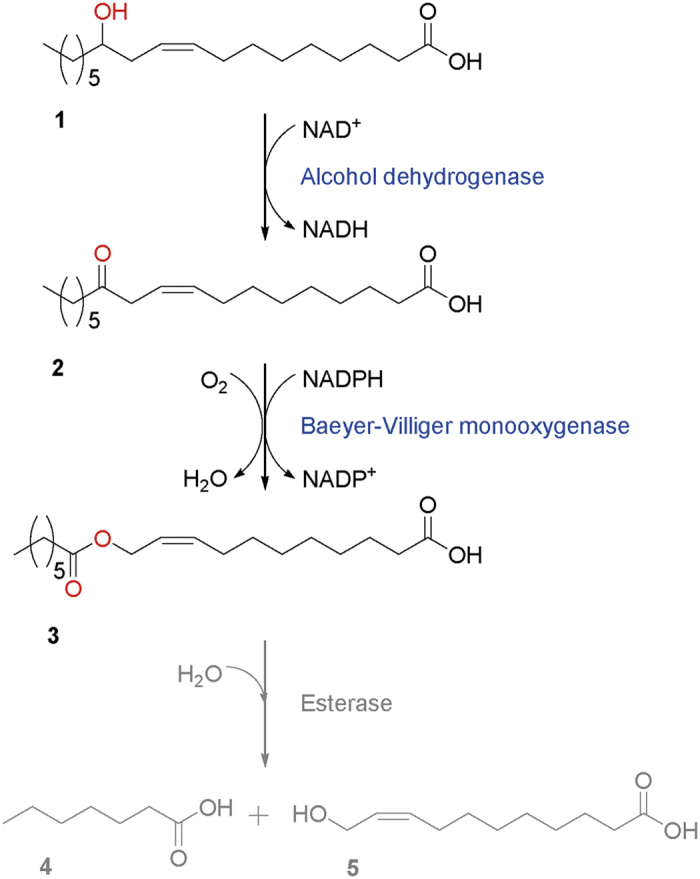
Designed biotransformation pathway. Ricinoleic acid (**1**) is enzymatically converted into the ester (**3)**, which can be hydrolyzed into *n*-heptanoic acid (**4**) and (*Z*)-11-hydroxyundec-9-enoic acid (**5**)^7^. Adopted from our previous study^7^.

The fusion of soluble peptides or proteins to target proteins is one of the strategies to enhance functional expression of the enzymes and proteins^18^,^24^,^25^. Thereby, the widely used soluble tags (e.g., ubiquitin (Ub)^26^,^27^ and polyionic tags (i.e., hexa-glutamates (E6) and hexa-lysines (K6)) were introduced into N-terminal of the BVMO from *P. putida* KT2440 12. After construction of E6-, K6-, Ub-BVMO fusion genes, they were expressed in *E. coli* BL21(DE3) at 20 °C. The soluble expression level of the BVMO was in the order of K6-BVMO > Ub-BVMO > the native BVMO > E6-BVMO, indicating that the K6 and Ub served as soluble tags. However, catalytic activity of the fusion enzymes isolated remained low because of low enzyme stability outside cells, as previously reported^12^,^17^. Thereby, we had to evaluate catalytic activity of the fusion enzymes by whole-cell biocatalysis. After construction of the recombinant *E. coli* BL21(DE3) pACYC-ADH, pJOE-tag-BVMO expressing the fusion enzymes and the long chain secondary alcohol dehydrogenase (ADH) of *Micrococcus luteus* NCTC2665 7, whole-cell biotransformation of ricinoleic acid (**1**) into the ester (**3**) via 12-ketooleic acid (**2**) (Fig. 1) was carried out. After the recombinant genes were expressed at 20 °C, ricinoleic acid was added into 15 mM in the culture broth at the stationary growth phase, as previously described^28^. The biotransformation activity of *E. coli* pACYC-ADH, pJOE-E6/K6-BVMO expressing the E6- or K6-BVMO fusion enzymes was comparable to that of *E. coli* pACYC-ADH, pJOE-BVMO, whereas that of *E. coli* pACYC-ADH, pJOE-Ub-BVMO was lower (see the Supplementary Fig. S1).

**Figure 2:**
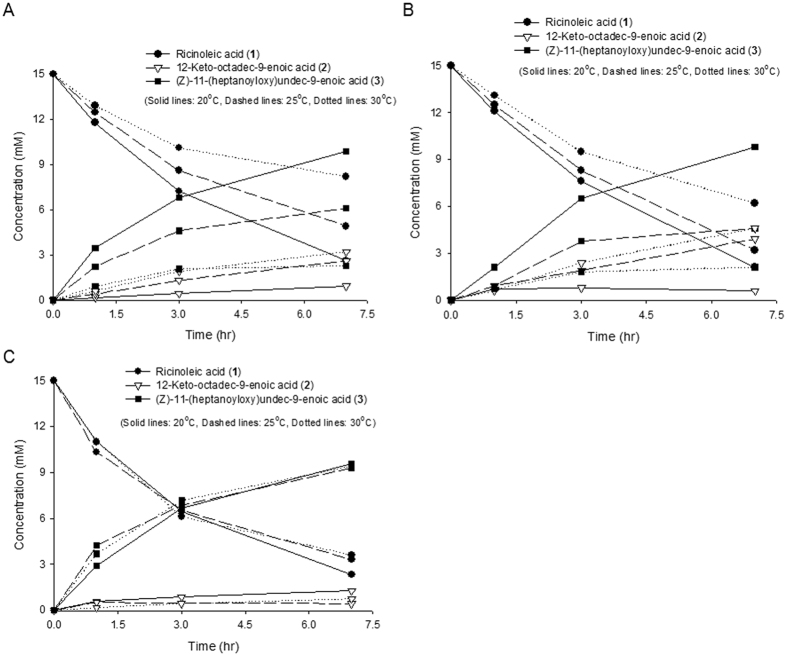
Time course of the biotransformation of ricinoleic acid (**1**) by the recombinant (**A**) *E. coli* BL21(DE3) pACYC-ADH, pJOE-BVMO^28^, (**B**) *E. coli* BL21(DE3) pACYC-ADH, pJOE-K6-BVMO, and (**C**) *E. coli* BL21(DE3) pACYC-ADH, pJOE-E6-BVMO. The recombinant cells express not only the BVMOs but also the alcohol dehydrogenase (ADH) from *M. luteus*. The target gene expression was induced by adding 0.1 mM IPTG and 2 g/L rhamnose at 20 °C (solid lines), 25 °C (dashed lines), and 30 °C (dotted lines) at the exponential growth phase (cell density: 0.2 g dry cells/L). The biotransformation was initiated at the stationary growth phase (cell density: 3 g dry cells/L) by adding 15 mM ricinoleic acid and 0.5 g/L Tween80 to the culture broth. The average values of three-independent experiments were used for the plotting. The standard deviation was less than 10%.

The biotransformation activity of *E. coli* pACYC-ADH, pJOE-E6/K6-BVMO was next evaluated after the BVMO gene expression was induced at room temperatures (i.e., 25 °C and 30 °C), which is important for large scale biotransformations. The ester product (**3**) formation rate of *E. coli* pACYC-ADH, pJOE-BVMO and *E. coli* pACYC-ADH, pJOE-K6-BVMO was markedly reduced with increase of the culture temperature from 20 to 30 °C (Fig. 2A,B). However, the biotransformation rate of *E. coli* pACYC-ADH, pJOE-E6-BVMO remained rather unchanged (Fig. 2C); the ester production rate and the final ester concentration in the culture broth reached ca. 0.44 mmol/g dry cells/h and 9.3 mM, respectively, even when the gene expression was induced at 30 °C. These values were ca. 4-fold greater as compared to the biotransformation activity of *E. coli* pACYC-ADH, pJOE-BVMO. The same profile was also observed with other reaction substrates (e.g., 10-hydroxyoctadecanoic acid) (Supplementary Fig. S2,3). The SDS-PAGE analysis of the BVMO enzymes expressed in the recombinant *E. coli* clearly showed that majority of the native BVMO was expressed in an insoluble form when the gene expression was induced at 30 °C (Supplementary Fig. S4A). In contrast, the soluble and insoluble expression level of the E6-BVMO was not significantly affected by the induction temperatures (Supplementary Fig. S4B). Therefore, it was assumed that the negatively charged E6 tag allowed proper folding of the BVMO and maintenance of the catalytic activity in *E. coli* cells at the high temperatures up to 30 °C.

Effects of codon optimization of the BVMO were investigated via SDS-PAGE analysis and the *E. coli*-based whole-cell biocatalysis. The codon optimization of the BVMO led to an increase in soluble expression level in *E. coli* BL21(DE3) pACYC-ADH, pJOE-E6-BVMO_opt_, but to only slight increase in the ester (**3**) production rate of the recombinant (Supplementary Fig. S5).

### Construction of a constitutive gene expression system

One of the critical issues in industrial biotransformations includes construction of the gene expression system, which does not need expensive inducers. In fact, the gene expression system pACYC-ADH and pJOE-E6-BVMO_opt_ depend on IPTG and rhamnose as inducers, respectively (Supplementary Table S1). Thereby, an inducer-free promoter system was investigated for expression of the cascade enzymes in *E. coli*. A very strong synthetic promoter (i.e., J23100 (Identifier: BBa_J23100 (http://parts.igem.org/Promoters/Catalog/Anderson)-driven gene expression system was designed and constructed (Supplementary Table S1 and Supplementary Fig. S6).

**Figure 3:**
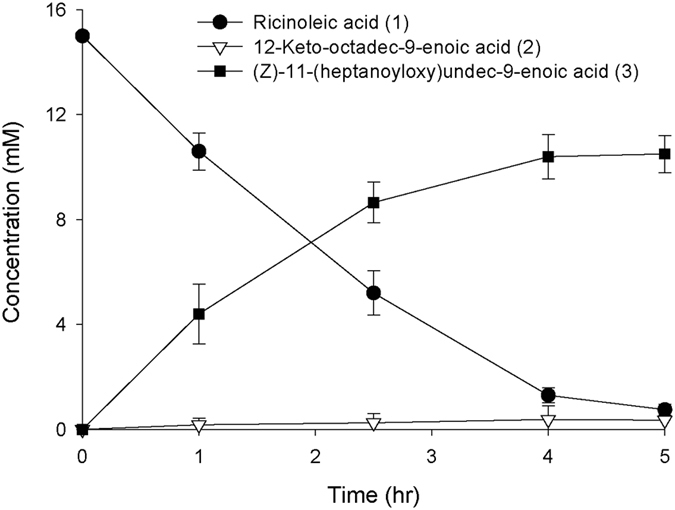
Time course of the biotransformation of ricinoleic acid (**1**) by the recombinant *E. coli* BL21(DE3) pAPTm-E6-BVMO_opt_-ADH in a flask, expressing the E6-BVMO_opt_ and the ADH from *M. luteus*. The target gene expression was initiated from the beginning of the cultivation at 25 °C. The biotransformation was carried out at the same conditions as in the experiment in Fig. 2. The error bars indicate the standard deviation.

Performance of the resulting inducer-free synthetic promoter system (i.e., pAPTm-E6-BVMO_opt_-ADH) was examined via the *E. coli*-based whole-cell biocatalysis. The whole-cell biotransformation rate of ricinoleic acid, which was estimated with the ester (**3**) concentration in the reaction medium and the reaction time when 90% of the reaction substrate was consumed, was increased up to 0.88 mmol/g dry cells/h (Fig. 3). This value is over 40% higher than that of *E. coli* BL21(DE3) pACYC-ADH, pJOE-E6-BVMO_opt_ under comparable conditions. Overall, it was assumed that the synthetic promoter system is quite efficient in terms of functional expression of the BVMO in the *E. coli* cells.

### Biotransformation performance of *E. coli* BL21(DE3) pAPTm-E6-BVMO_opt_-ADH

**Figure 4:**
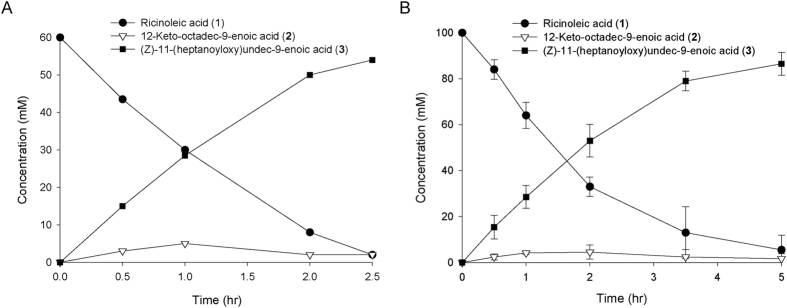
Time course of the biotransformation of ricinoleic acid (1) by the recombinant *E. coli* BL21(DE3) pAPTm-E6-BVMO_opt_-ADH in a lab-scale bioreactor, expressing the E6BVMO_opt_ and the ADH from *M. luteus*. The target gene expression was initiated from the beginning of the cultivation at 30 °C. The biotransformation was initiated by adding (**A**) 60 mM or (**B**) 100 mM ricinoleic acid and 0.5 g/L Tween80 to the culture broth after fed-batch cultivation to a cell density of (**A**) 20 g dry cells/L or (**B**) 25 g dry cells/L. The error bars indicate standard deviation.

The biotransformation activity of recombinant *E. coli* BL21(DE3) pAPTm-E6-BVMO_opt_-ADH was evaluated by doing ricinoleic acid biotransformation at a lab scale (5 L) bioreactor (working volume: 2 to 2.5 L in final) based on our previous studies^28^,^29^. After high cell density cultivation to ca. 20 g dry cells/L by pH-stat fed-batch fermentation, ricinoleic acid was added to 60 mM into the culture broth. Ricinoleic acid was converted into the ester (**3**) to a final concentration of 54 mM in the culture broth (Fig. 4A). The volumetric productivity and bioconversion yield reached 21.6 mM/h and 88%, respectively (see the Biotransformation 1 in Table 1). The volumetric productivity was ca. 3-fold higher as compared to that of *E. coli* BL21(DE3) pACYC-ADH, pJOE-BVMO under comparable conditions (see the Biotransformation 1 and 4 in Table 1).

With an aim to enhance the final ester concentration in the reaction medium, ricinoleic acid was added to 100 mM in the culture broth after cultivation to 25 g dry cells/L. Further increases in the substrate and biocatalyst concentrations led to a final product concentration and volumetric productivity of 85 mM (conversion yield, 85%) and 17.2 mM/h, respectively (Fig. 4B and see the Biotransformation 2 in Table 1). The ester products were isolated from the reaction medium, after separation of the cell mass from the culture broth by a vacuum filtration using the Celite filter aid (Sigma-Aldrich) and extraction with ethylacetate (see Supplementary Fig. S7 for details). Over 95% of the ester products isolated were found in the cell mass fraction, indicating that the ester (**3**), which structure is similar to the fatty acid constituents of phospholipids, might stay in the cell membrane layer, as hydrophobic molecules do^30^,^31^,^32^. The crude ester products isolated were subjected to hydrogenation of the double bond in carbon skeleton, hydrolysis of the ester bond, and oxidation of the resulting hydroxyl group to carboxylic acid, as described previously^28^. The conversion yield of the three chemical steps was over 80% (Supplementary Fig. S8 and Supplementary Table S3). Finally, 27.4 g of 1,11-undecanedioic acid (purity >95%) was obtained from 53.2 g esters in the reaction medium. The overall molar yield of 1,11-undecanedioic acid from ricinoleic acid reached ca. 60%.

### Scale-up of *E. coli* BL21(DE3) pAPTm-E6-BVMO_opt_-ADH biotransformation process

The next step in this study has focused on scale-up of the ricinoleic acid biotransformation process of *E. coli* BL21(DE3) pAPTm-E6-BVMO_opt_-ADH, which was shown in Fig. 4B. Based on the microbial Baeyer–Villiger oxidation of bicyclo[3.2.0]-hept-2-ene-6-one^33^, the oxygen supply conditions (e.g., agitation speed, aeration rate, and pure oxygen supply rate) were considered as a major scale-up parameter. The agitation speed, aeration rate, and pure oxygen supply rate were automatically controlled to avoid oxygen limitation (see the Materials and Methods for details).

**Figure 5:**
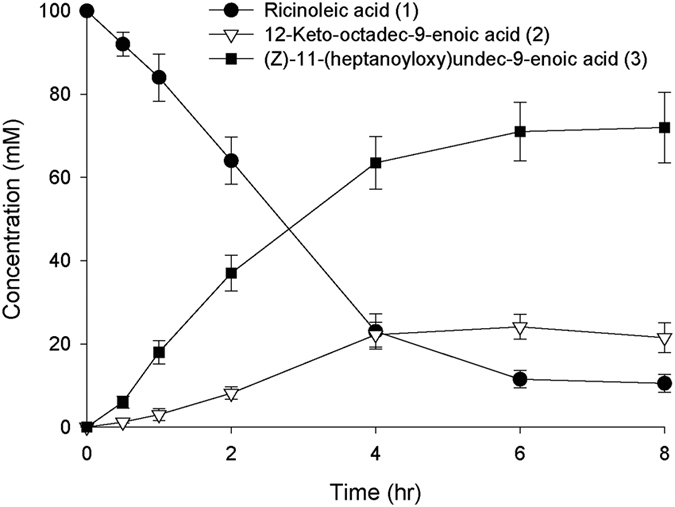
Time course of the biotransformation of ricinoleic acid (**1**) by the recombinant *E. coli* BL21(DE3) pAPTm-E6-BVMO_opt_-ADH in a 70L-scale bioreactor. The target gene expression was initiated from the beginning of the cultivation at 30 °C. The biotransformation was initiated by adding 100 mM ricinoleic acid and 0.5 g/L Tween80 to the culture broth after fed-batch cultivation to a cell density of 30 g dry cells/L. The error bars indicate standard deviation.

The biotransformation was initiated by adding 100 mM ricinoleic acid into the culture broth when *E. coli* BL21(DE3) pAPTm-E6-BVMO_opt_-ADH grew to ca. 30 g dry cells/L (see Supplementary Fig. S9 for the growth curve). The biotransformation dynamics was similar to that of the lab scale experiment (Fig. 5). The ester product (**3**) was produced to a rate of 15.7 mM/h with a bioconversion yield of 72% (see the Biotransformation 3 in Table 1). The final product concentration reached 72 mM or 22 g/L in the culture medium. The accumulation of the ADH reaction products (**2**) in the reaction medium and lower bioconversion yield at the pilot-scale experiment might be ascribed to low expression level of the BVMO in the *E. coli* cells. Optimization of the cultivation conditions may lead to further increase of the BVMO reaction rates and thereby of the bioconversion yield. Overall, it was assumed that the lab scale biotransformation process shown in Fig. 4B could be successfully scaled-up for industrial applications.

## Discussion

Since the BVMOs have been identified in 1976^34^, numerous studies have reported the type of catalytic reactions, the structural properties and reaction mechanisms, and the protein engineering to improve their catalytic activities and structural stability^1^,^2^,^3^,^4^,^5^,^35^,^36^,^37^,^38^. In addition, biotransformation process engineering such as whole-cell reactions (at high cell density)^28^, bioprocess optimization^33^, *in situ* product recovery^39^,^40^,^41^,^42^, and bioreactor design^39^,^40^ has been intensively investigated to enhance the final product concentrations in the reaction medium and volumetric productivities. One of the milestones was biotransformation of bicyclo[3.2.0]hept-2-ene-6-one to a mixture of the two corresponding lactones by recombinant *E. coli* expressing the cyclohexanone monooxygenase from *Acinetobacter calcoaceticus* NCIMB 9871^39^,^41^. The whole-cell biotransformation process allowed high product concentration and bioconversion yield of ca. 16 g/L and 83%, respectively, by attenuating product toxicity via *in situ* product recovery with 100 g/L hydrophobic absorbent resins.

In this study, the BVMO of *P. putida* KT2440 and the gene expression system was engineered for the large-scale biotransformation of ricinoleic acid (**1**) into the ester (**3**) (Fig. 1). With an aim to improve the catalytic activity and stability under process conditions, the BVMO was subjected not only to directed evolution but also rational protein engineering. Since the BVMO of *P. putida* KT2440 was able to catalyze the regiospecific oxygenation of 4-hydroxy-2-decanone (**11**) (Supplementary Fig. S10), the screening method, which was based on Baeyer-Villiger monooxygenation of 4-hydroxy-2-decanone^22^, was used in the study. Thousands colonies, which had been produced via error-prone PCR, were examined. However, the variant, which is more active than the native enzyme, was not isolated.

The BVMO from *P. putida* KT2440^12^ was engineered by assuming that negatively charged residues in the N- or C-terminal play a critical role in thermal stability of proteins^23^. Remarkably, functional expression and probably structural stability of the BVMO in *E. coli* under room temperatures up to 30 °C was significantly improved via fusion with a polyionic peptide tag (i.e., hexa-glutamate (E6)) (Fig. 2 and Supplementary Fig. S3,4). Introduction of the E6 tag into N-terminal of the enzyme appears to enhance proper folding of the newly synthesized polypeptides and structural stability of the active enzymes at the high temperatures. Although the mechanism(s) remain to be investigated, one of the reasons might be similar to that of Group II chaperonins, which thermal stability was dependent upon the number of Glu residues rather than Lys residues in the C-terminal^23^. One possibility would be generation of salt bridge between Glu residues of the tag and the Arg residue sitting around N-terminal of the BVMO (Supplementary Fig. S11, structure prediction was performed as previously reported^44^). Since the BVMOs were reported to undergo a great deal of conformational change during catalysis^45^, the resulting salt bridge might contribute to stabilization of the transition states of the enzyme-NADP(H)-substrate complex in addition to correct folding at high temperatures. Overall, it was assumed that fusion of a polyionic tag (e.g., hexa-glutamate (E6)) to the target enzymes is one of the strategies to enhance functional expression and probably structural stability of the enzymes and proteins in *E. coli* at high temperatures. The further engineering of the E6-BVMO was conducted by using Rosetta^43^. For all the residues in BVMO sequence, *in silico* construction of single mutation and the calculation of ΔΔG were performed. Top five putative thermostable mutants were constructed and subjected to the *E. coli*-based whole-cell biocatalysis (see Experiment 1 in the Supporting information for details). However, this approach did not generate any superior variant to the native (i.e., E6-BVMO) in terms of the catalytic activity at high temperatures.

Engineering of the gene expression system for the cascade enzymes (i.g., ADH and E6-BVMO_opt_) to overexpress the target proteins without any inducer allowed the biotransformation rate to increase up to 21.6 mM/h (6.7 g/L/h); 60 mM ricinoleic acid (**1**) was converted into 54 mM ester (**3**) in the culture medium within 2.5 h (Fig. 4 and Table 1). This value is ca. 3-fold higher as compared to the ricinoleic acid biotransformation rate of *E. coli* BL21(DE3) pACYC-ADH, pJOE-BVMO under comparable conditions (see the Biotransformation 1 and 4 in Table 1). The bioprocess was also successfully scaled up to a 70 L bioreactor (Fig. 5). This is the highest productivity of BVMO-based whole-cell biocatalysts with rather high bioconversion yield to our knowledge. In particular, the biotransformation process was very simple; the biotransformation was done by adding ca. 30 g/L reaction substrate into an aqueous culture broth in a conventional bioreactor without applying any complex systems such as *in situ* product recovery.

One of the factors to influence the whole-cell biotransformations could be product toxicity toward *E. coli* cells. Most of the ester product (**3**) was found in cell mass fraction rather than in extracellular space (Supplementary Fig. S7). Since its chemical structure is similar to the constituent of phospholipids of cellular membranes, it may accumulate in the cell membranes. If the ester (**3**) accumulates in the cell membranes, it may affect permeability and structural properties of cell membranes as shown with hydrocarbons^30^,^31^,^32^,^46^. This may in turn disturb cellular metabolic reactions including NADPH regeneration essential for the BVMO reactions and enzyme turn-over in the whole-cell biocatalyst. Thereby, our future research will focus on extraction and recovery of the ester products from cell mass. If the product is isolated from the biomass without metabolic stress or damage, the whole-cells could be further used as biocatalysts for the next round of biotransformations.

## Conclusion

This study demonstrated that the hexa-glutamate tag played a critical role to improve functional expression and probably structural stability of the BVMO from *P. putida* KT2440 in *E. coli* BL21(DE3). By applying the polyionic tag (E6)-based BVMO engineering and the synthetic promoter-driven constitutive gene expression system, the biotransformation activity of the *E. coli*-based whole-cell biocatalyst was significantly enhanced under process conditions. Furthermore, the biocatalyst system was shown to be applicable for large scale biotransformations. This study will contribute to industrial application of BVMO-based whole-cell biocatalysis.

## Materials and Methods

### Microbial strains, culture conditions and expression of heterologous genes

For seed culture, recombinant *E. coli* BL21(DE3) strains were cultivated overnight in 3 mL of lysogeny broth (LB) medium supplemented with appropriate antibiotics (see the Supplementary Table S1). The Riesenberg medium^47^ supplemented with 10 g/L glucose and the appropriate antibiotics was used for the main cultivation and biotransformation. The Riesenberg medium consisted of 4 g/L (NH_4_)_2_HPO_4_, 13.5 g/L KH_2_PO_4_, 1.7 g/L citric acid, 1.4 g/L MgSO_4_, and 10 ml/L trace metal solution (10 g/L FeSO_4_, 2.25 g/L ZnSO_4_, 1.0 g/L CuSO_4_, 0.5 g/L MnSO_4_, 0.23 g/L Na_2_B_4_O_7_, 2.0 g/L CaCl_2_, and 0.1 g/L (NH_4_)_6_Mo_7_O_24_). When OD_600_ is 0.6–0.7, heterologous gene expression was induced by adding 0.1 mM isopropyl-β-D-thiogalactopyranoside (IPTG) and/or 2 g/L rhamnose to the culture broth. Then, the cultures were further incubated at 20 to 30 °C.

### Chemicals and materials

Ricinoleic acid and rhamnose were purchased from Tokyo Chemical Co (Tokyo, Japan). Glucose was purchased from Junsei Chemical Co (Tokyo, Japan). Antibiotics, trace elements for culture medium, IPTG, and Tween80 were purchased from Sigma (St. Louis, MO, USA). Ethyl acetate was purchased from Duksan Pure Chemical Co. (Ansan, Republic of Korea). *N*-Methyl-*N*-(trimethylsilyl)trifluoroacetamide (TMS) was obtained from Tokyo Chemical Industry Co. (Tokyo, Japan).

### Cloning of soluble tag-BVMO fusion enzymes

E6-BVMO and K6-BVMO was constructed using the primers including E6 gene (primer 1) or K6 gene (primer 2) in their sequences (Supplementary Table S2). E6-BVMO and K6-BVMO were amplified using primer 1 & 3 and primer 2 & 3, respectively. Primer 4 & 5 and primer 6 & 7 were used to amplify ubiquitin (Ub) and BVMO, respectively. Ub-BVMO gene was constructed using Gibson assembly method. Each insert was digested using appropriate restriction enzymes. Double digested genes were ligated into appropriate vector using T4 DNA ligase (New England Biolabs, Ipswich, MA, USA). For pAPTm-E6-BVMO_opt_-ADH cloning, pAPT vector except promoter and multi-cloning site was amplified using primer 8 & 9. E6-BVMO_opt_ and ADH were amplified using primer 10 & 11 and primer 12 & 13, respectively. Ligated plasmid was introduced into *E. coli* DH5α. Transformants were inoculated in 3 mL LB medium and cultivated overnight. Recombinant plasmids were prepared from cultured *E. coli* cells using Exprep plasmid purification kit (GeneAll Biotechnology Co, Seoul, Korea).

### Biotransformation of ricinoleic acid

Biotransformation of ricinoleic acid by recombinant *E. coli* in a 250 ml flask (reaction volume: 20 mL) was carried out on the basis of our earlier work^7^,^28^. Briefly, the recombinant cells were cultivated in Riesenberg medium at 30 °C, and expression of the target genes was induced with 0.1 mM IPTG at an OD_600_ of 0.6 at 20 °C. When the culture reached the stationary growth phase (cell concentration: 3.0 g dry cells/L), the biotransformation was initiated by adding 15 mM ricinoleic acid and 0.5 g/L Tween 80 into the culture broth. The pH and temperature of culture broth was set to 8.0 and 35 °C, respectively.

The biotransformations in a lab scale (5 L) bioreactor (working volume: 2 to 2.5 L in final) (Biotron, Bucheon, Korea) was based on our previous study^28^. The recombinant *E. coli* BL21(DE3) pAPTm-E6-BVMO_opt_-ADH was grown batch-wise at 30 °C until the initially added glucose (20 g/L) was completely exhausted. Upon glucose depletion and concomitant elevation of pH >6.9, a mixture of glucose (600 g/L) and MgSO_4_·7H_2_O (20 g/L) was fed using the pH-stat feeding strategy. Cultivation pH was automatically maintained at pH 6.9 by feeding 28% ammonia solution into the culture broth. Agitation speed and aeration rate were automatically controlled to keep the DOT of culture broth over 40%. When the agitation speed and aeration rate required reached over 1000 rpm and 2 vvm, respectively, pure oxygen was automatically supplied into the bioreactor. The biotransformation was initiated by adding 60 to 100 mM ricinoleic acid and 0.5 g/L Tween80 into the culture broth. During the biotransformation, glucose feeding was stopped. The reaction pH and temperature was kept at pH 8.0 and 35 °C, respectively, as previously reported^28^. Agitation speed and aeration rate were set to 800 rpm and 1 vvm, respectively. Pure oxygen was not supplied into the reaction medium during the biotransformation because of low oxygen requirement in the absence of glucose.

### Scale-up of the lab-scale biotransformation process

The whole-cell biotransformation process consisted of two stages; the first is the fed-batch cultivation of the *E. coli* recombinants and the second is the biotransformation of ricinoleic acid into the ester (**3**) (Fig. 1). Scale-up of the fed-batch cultivation of the *E. coli* cells is well-known, whereas the whole-cell biotransformation of lipophilic substrates at high concentrations in aqueous medium remained rather untouched. One of the key factors in hydrophobic substrate biotransformations may include transport of the reaction substrates into the whole-cells, where the cascade enzymes are present. Another factor could be oxygen supply into the oxidative enzymes in whole-cells, as demonstrated with the microbial Baeyer–Villiger oxidation of bicyclo[3.2.0]-hept-2-ene-6-one^33^.

During the fed-batch cultivation of the recombinant *E. coli* BL21(DE3) pAPTm-E6-BVMO_opt_-ADH, the agitation speed and aeration rate were automatically controlled to keep the DOT of culture broth over 40%. When the agitation speed and aeration rate reached over 500 rpm and 2 vvm, respectively, pure oxygen was automatically supplied into the bioreactor, as in the lab-scale fed-batch cultivation. The rest conditions were the same as in the lab-scale experiment. During the biotransformation of ricinoleic acid, the agitation speed and aeration rate were set to 400 rpm and 1 vvm, respectively, which allowed to maintain the DOT of culture broth over 40%. Pure oxygen was not supplied because of low oxygen requirement in the absence of glucose in the reaction medium. The rest biotransformation conditions were the same as in the lab-scale experiment.

### Analytical methods

Concentrations of ricinoleic acid, final product and other intermediates were measured according to the method based on our earlier work^7^. The reaction broth was mixed with a twice volume of ethyl acetate containing 0.5 g/L methyl palmitate as an internal standard. The organic phase was harvested after vigorous vortexing and then subjected to derivatization with TMS. The TMS derivatives of the fatty acids were analyzed by a gas chromatography mass spectrometry (GC-MS) (Agilent, Santa Clara, CA, USA) equipped with a flame ionization detector and a split injection system (split ratio set at 1:10) and fitted with a SPB-1 capillary column (15 m × 0.32 mm inside diameter, 0.25 μm thickness) (Supelco, Bellfonte, PA, USA). Column temperature was increased from 90 to 255 °C at a rate of 5 °C/min, and then maintained at 255 °C. The injector and detector temperatures were 260 and 250 °C, respectively.

### Homology modeling of the BVMO of *P. putida* KT2440

Homology model of the BVMO of *P. putida* KT2440 was constructed using the method of Joo *et al*.^44^. Template selection was performed using CRFalign utilizing probabilistic pairwise alignment of sequence and structure with boosted regression trees as a score function. 3UOV A chain (sequence identity: 22.1%), 3UCL A chain (sequence identity: 19.5%), 1W4X A chain (sequence identity: 18.8%), and 4AOS A chain (sequence identity: 18.7%) were selected as templates. Tertiary structures were constructed using Modeller and structure with the lowest energy was searched using conformational space annealing. Side chain remodeling was performed using RotamerCSA with residue-dependent rotamer library and standard SCWRL4 rotamer library. Homology model in PDB format was provided as Appendix in Supplementary Information.

### Accession information of the enzyme sequences

The sequences of the enzymes used in this study are found at the following Uniprot accession number: Q88J44 (BVMO from *P. putida* KT2440), C5C716 (ADH from *M. luteus*).

## Additional Information

**How to cite this article**: Seo, J.-H. *et al*. Engineering of Baeyer-Villiger monooxygenase-based *Escherichia coli* biocatalyst for large scale biotransformation of ricinoleic acid into (*Z*)-11-(heptanoyloxy)undec-9-enoic acid. *Sci. Rep.* **6**, 28223; doi: 10.1038/srep28223 (2016).

## Supplementary Material

Supplementary Information

## Figures and Tables

**Table 1:** Biocatalytic performance of the recombinant *Escherichia coli*-based biocatalysts.

	Biotransformation 1^a^	Biotransformation 2^a^	Biotransformation 3^a^	Biotransformation 4^a^
Induction temperature (°C)^b^	30	30	30	20
Inducer for the target gene expression	Not applied	Not applied	Not applied	IPTG, Rhamnose
Substrate concentration (mM)	60	100	100	63
Biocatalyst concentration (g dry cells/L)	20	25	30	20
Final product concentration (mM)	54	85 ± 4	72 ± 6	53
Volumetric productivity (mM/h)^c^	21.6	17.2 ± 0.7	15.7 ± 1.3	6.6
Product yield (%)^d^	88	85 ± 4	72 ± 6	84

^a^Biotransformation 1, 2, 3, and 4 indicates the experiment shown in Figs 4A,B and 5, and in our previous study^28^, respectively. The biotransformation 4 was conducted by the recombinant *E. coli* BL21(DE3) pACYC-ADH, pJOE-BVMO at the reaction conditions identical to the Biotransformation 1.

^b^The cultivation temperature, which was maintained after induction of expression of the cascade enzymes in *E. coli*.

^c^Volumetric productivity was calculated based on the ester product concentration, which was determined by gas chromatography/liquid chromatography (GC/MS), and the biotransformation time, which was measured when >90% of the starting material was converted to the products.

^d^Product yield was calculated based on the initial substrate concentration and the final ester product concentration, which were determined by GC/MS.
