# Supplementary material for: Engineering of Baeyer-Villiger monooxygenase-based Escherichia coli biocatalyst for large scale biotransformation of ricinoleic acid into (Z)-11-(heptanoyloxy)undec-9-enoic acid
Source: Sci Rep. 2016 Jun 17;6:28223. doi: 10.1038/srep28223 (PMC4911592; doi:10.1038/srep28223)
Supplement: Supplementary Information [file srep28223-s1.doc]

**Supplementary Information**

**Engineering of Baeyer-Villiger monooxygenase-based *Escherichia coli* biocatalyst for large scale biotransformation of ricinoleic acid into (*Z*)-11-(heptanoyloxy)undec-9-enoic acid**

Joo-Hyun Seo^1^, Hwan-Hee Kim^1^, Eun-Yeong Jeon^1^, Young-Ha Song^2^, Chul-Soo Shin^2^, and Jin-Byung Park^1*^

^1^Department of Food Science and Engineering, Ewha Womans University, Seoul 120-750, Republic of Korea

^2^AP Technology, Suwon, Kyunggi 443-702, Republic of Korea

**Table S1. Strains, plasmids, ubiquitin gene used in this study**

| Strain or Plasmids | Relevant properties or strain designation | References |
| --- | --- | --- |
| Strain |  |  |
| *E. coli* BL21 | F^-^ *omp*T lon *hsdS_B_*(r^-^_B_,m^-^_B_) *gal* *dcm* |  |
| Plasmids |  |  |
| pACYCDuet-1 | p15A origin, T7 lac promoter, Cm^R^ |  |
| pETDuet | pBR322 origin, T7 promoter, Amp^R^ |  |
| pJOE | pBR322 origin, rhamnose-inducible promoter, Amp^R^ |  |
| pAPTm | pBR322 origin, constitutive promoter (BBa_J23100, http://parts.igem.org/Promoters/Catalog/Anderson), Kan^R^ |  |
| pACYC-ADH | The secondary alcohol dehydrogenase gene of *Micrococcus luteus* was cloned into pACYC vector | Song et al.^1^ |
| pJOE-BVMO | The BVMO gene of *Pseudomonas putida* KT2440 was cloned into pJOE vector | Song et al.^1^ |
| pJOE-E6-BVMO | The BVMO gene of *P. putida* KT2440 with E6-tag was cloned into pJOE vector | This study |
| pJOE-K6-BVMO | The BVMO gene of *P. putida* KT2440 with K6-tag was cloned into pJOE vector | This study |
| pJOE-Ub-BVMO | The BVMO gene of *P. putida* KT2440 with Ub-tag was cloned into pJOE vector | This study |
| pJOE-E6-BVMO_opt_ | Codon-optimized BVMO gene with E6 tag of *P. putida* KT2440 was cloned into pJOE vector | This study |
| pAPTm-E6-BVMO_opt_-ADH | The secondary alcohol dehydrogenase gene of *M. luteus* and codon-optimized gene of E6-tagged BVMO of *P. putida* KT2440 were cloned into pAPTm vector | This study |
| pETDuet-E6-BVMO_opt_ | The E6-BVMO_opt_ gene was cloned into pETDuet vector | This study |
| DNA sequence of ubiquitin | ttttcgtcaagactttgaccggtaaaaccataacattggaagttgaatcttccgataccatcgacaacgttaagtcgaaaattcaagacaaggaaggtatccctccagatcaacaaagattgatctttgccggtaagcagctagaagacggtagaacgctgtctgattacaacattcagaaggagtccaccttacatcttgtgctaaggctaagaggtggc |  |

**Table S2**. Primers used in this study

| Primer | Sequence |
| --- | --- |
| Primer 1 (sense primer for pJOE-E6-BVMO cloning) | AGATATACATATGGAAGAAGAAGAAGAAGAATCTTCTCACACCGCACT (NdeI) |
| Primer 2 (sense primer for pJOE-K6-BVMO cloning) | AGATATACATATGAAGAAAAAAAAGAAAAAGTCTTCTCACACCGCACT (NdeI) |
| Primer 3 (antisense primer for pJOE-E6-BVMO and pJOE-K6-BVMO cloning) | TCAGCCAAGCTTTTAACGGCGGCTGCCTTG (HindIII) |
| Primer 4 (sense primer for the amplification of Ub to construct Ub-BVMO) | AGATATACATATGCAGATTTTCGTCAA (NdeI) |
| Primer 5 (antisense primer for the amplification of Ub to construct Ub-BVMO) | GCCACCTCTTAGCCTTAGCAC |
| Primer 6 (sense primer for the amplification of BVMO to construct Ub-BVMO) | CTAAGAGGTGGCTCCTCTCACACTGCTCTT |
| Primer 7 (antisense primer for the amplification of BVMO to construct Ub-BVMO) | TCAGCCGGATCCTCATCGGCGGCTACCTTG (BamHI) |
| Primer 8 (sense primer for pAPTm vector amplification) | GCGGCCGCTGCTAACAAAGCCCGAAA (NotI) |
| Primer 9 (antisense primer for pAPTm vector amplification) | GTTTAAACTCATCAGTAACCCGTATC (PmeI) |
| Primer 10 (sense primer for BVMO_opt_ amplification including promoter for pAPTm-E6-BVMO_opt_,ADH cloning) | GTTACTGATGAGTTTAAACTTGACGGCTAGCTCAGTCCTAGGTACAGTGCTAGCTTAACTTTAAGAAGGAGATATACAT (PmeI) |
| Primer 11 (antisense primer for BVMO_opt_ amplification including promoter for pAPTm-E6-BVMO_opt_,ADH cloning) | AGATGCTCCTTTGAATTCTTAACGGCGGCTGCCTTG (EcoRI) |
| Primer 12 (sense primer for ADH amplification for pAPTm-E6-BVMO_opt_,ADH cloning) | GAATTCAAAGGAGCATCTATGTCCGAGTTCACCCGTTT (EcoRI) |
| Primer 13 (antisense primer for ADH amplification for pAPTm-E6-BVMO_opt_,ADH cloning) | CTTTGTTAGCAGCGGCCGCTCAGCCGAGCGGGGTGTC (NotI) |

**Table S3**. (Bio)synthesis of 1,11-undecanedioic acid from ricinoleic acid^a^

|  | Conversion yield (%) | Recovery yield (%) | Purity (%) |
| --- | --- | --- | --- |
| Ricinoleic acid  (starting material) | - | - | 81 |
| Biotransformation ^b^ | 85 | - | 64 |
| Ester (**3**) extraction | - | 96 | 64 |
| Chemical reaction steps  (Fig. S8) | 81 | - |  |
| Recrystallization | - | 90 | **>95** |

^a^ The ester (**3**) products, which were produced in the Biotransformation 2 (Table 1), were isolated from the reaction medium via extraction with ethylacetate (see Supplementary Fig. S7 for details). The crude ester products isolated were subjected to hydrogenation of the double bond, hydrolysis of the ester bond, and oxidation of the resulting hydroxyl group to carboxylic acid, as described previously^2^. The conversion yield of the three chemical steps was over 80% (Supplementary Fig. S8).

^b^ The biotransformation indicates the Biotransformation 2 shown in Table 1.

A


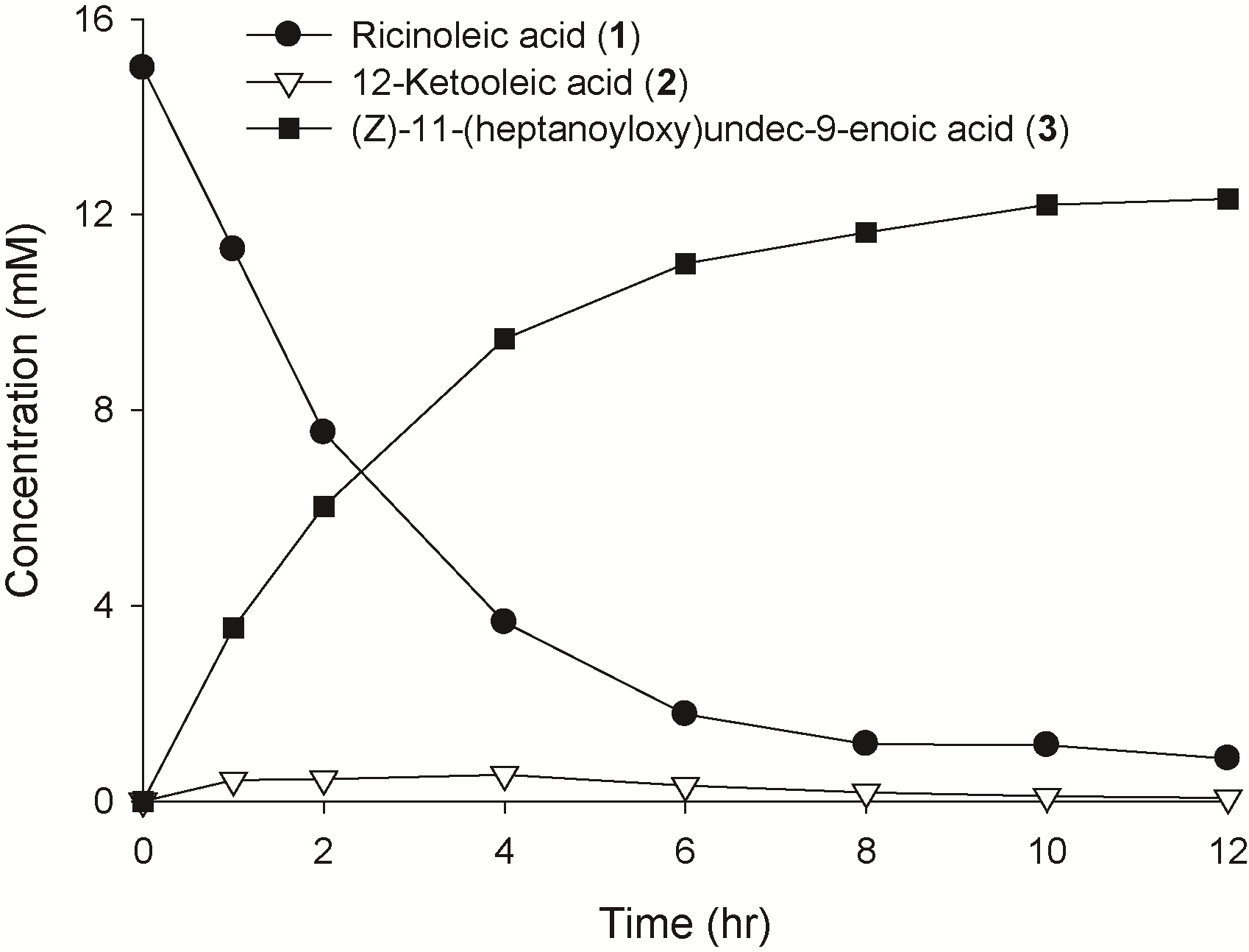


B


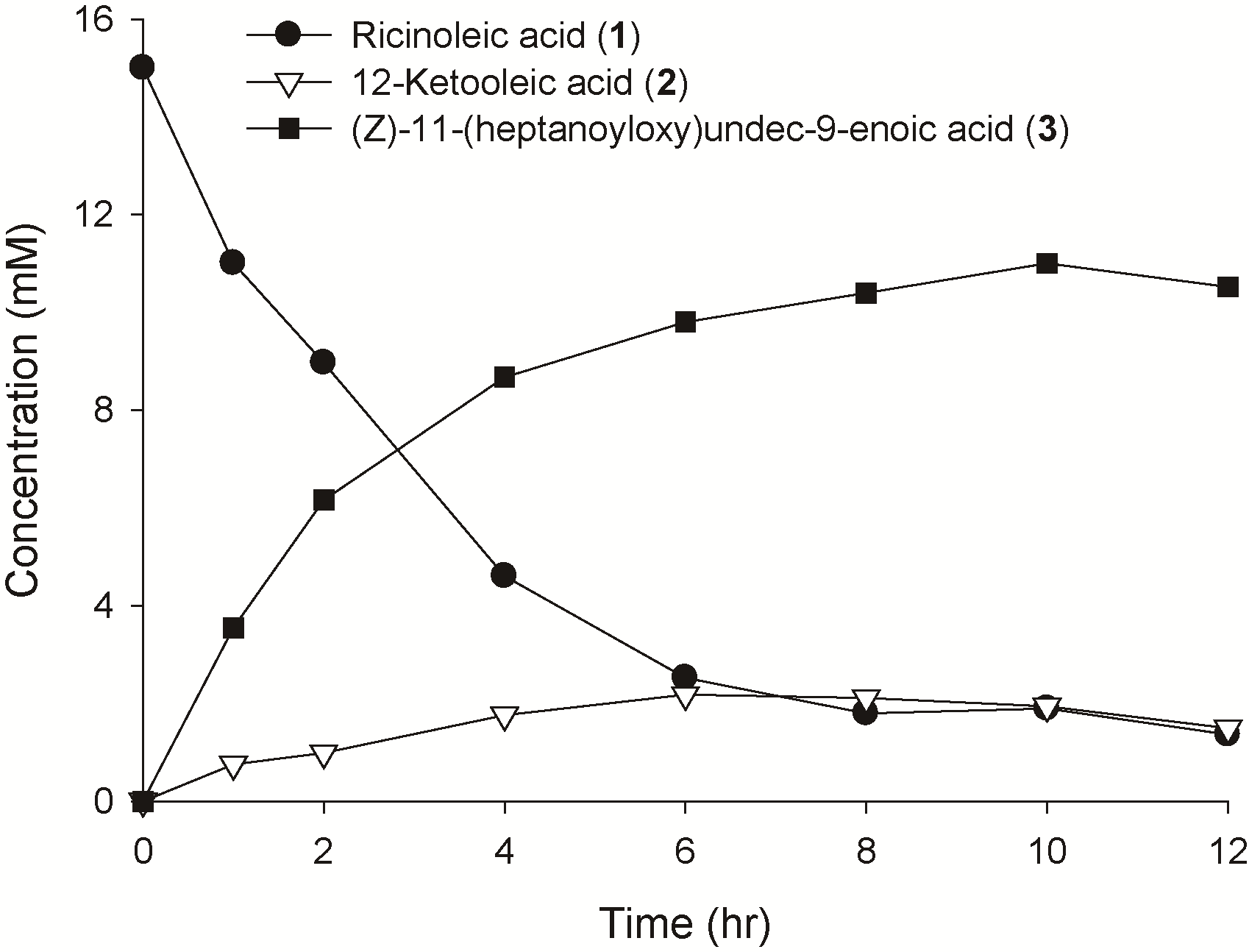


C


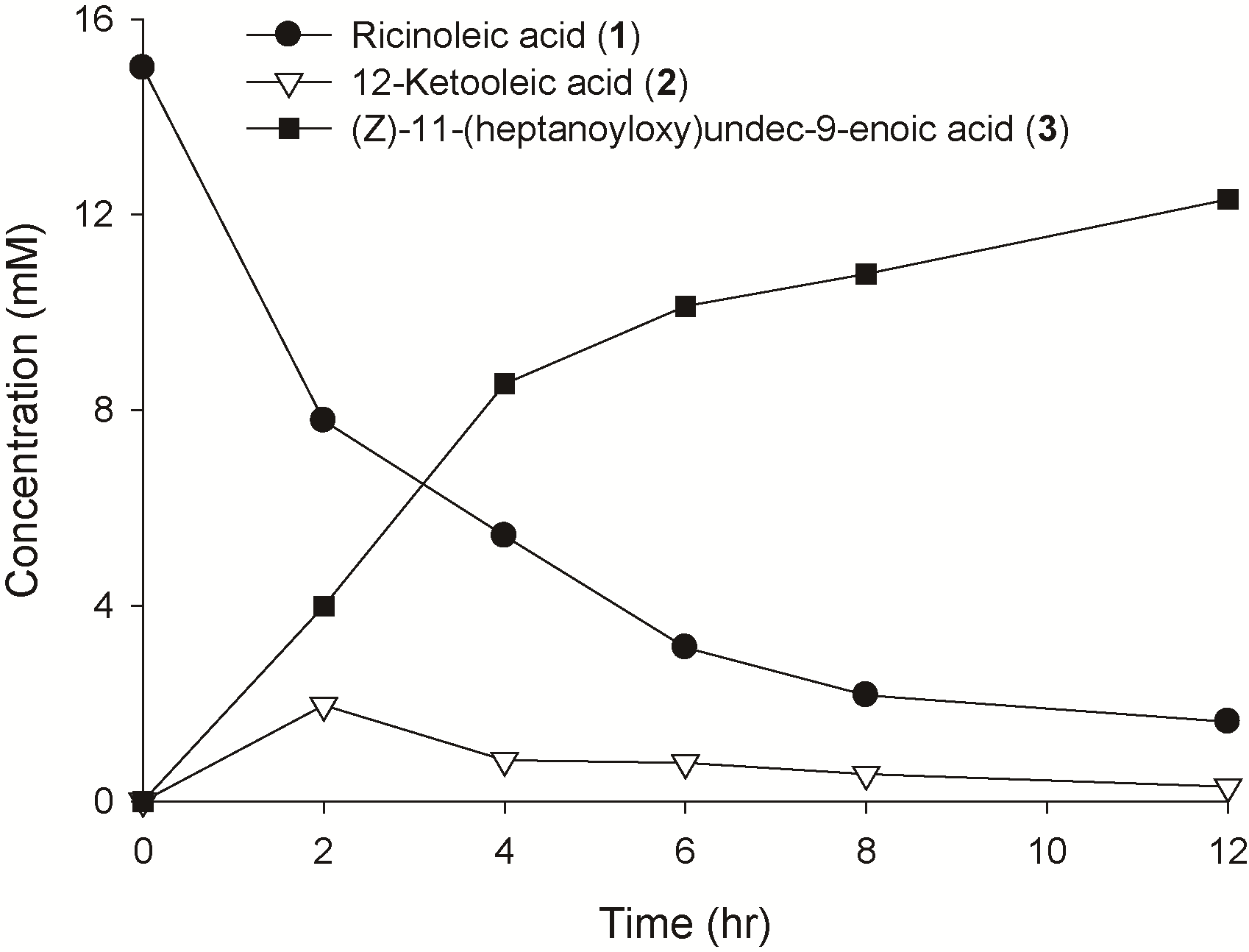


D


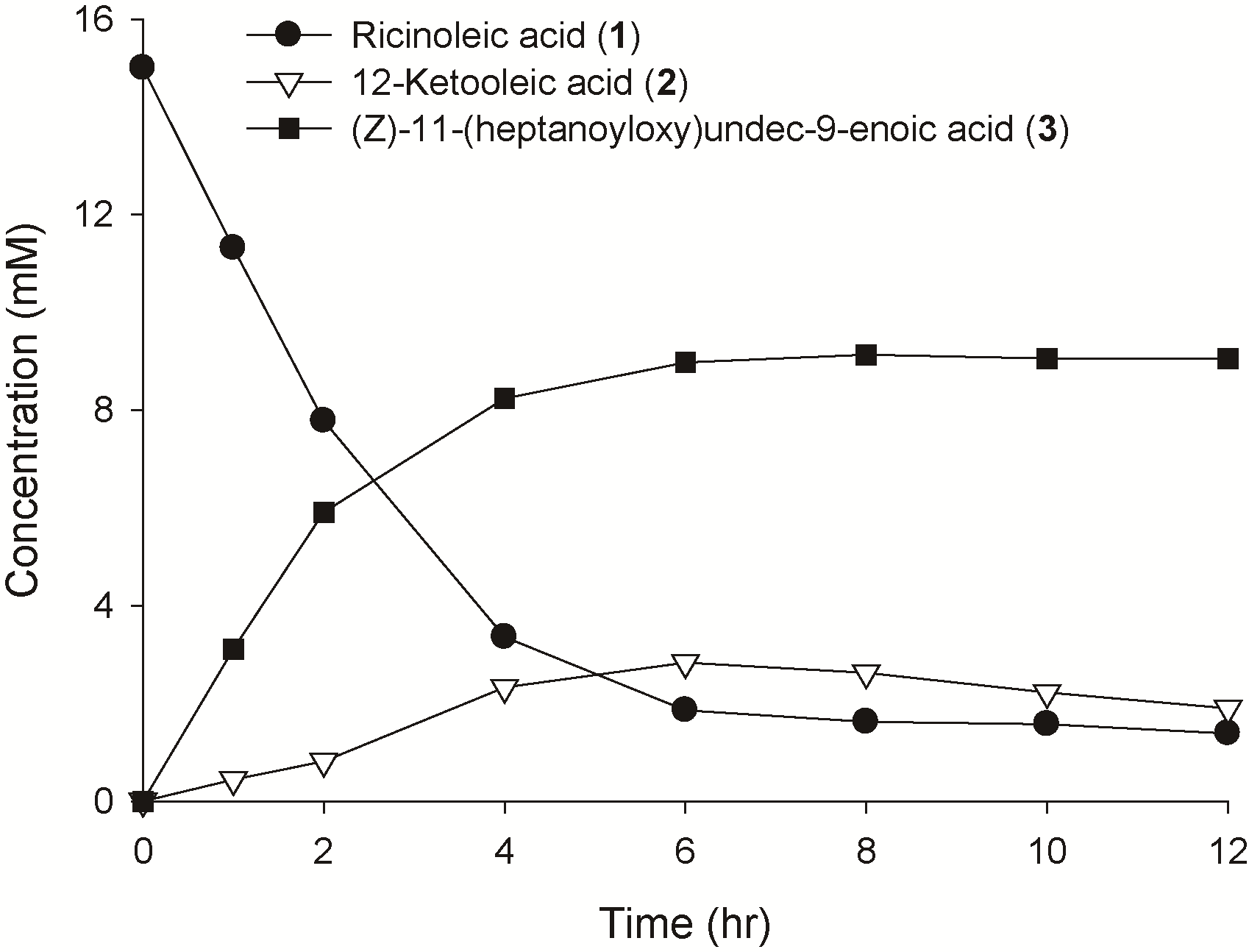


**Fig. S1**. Time course of the biotransformation of ricinoleic acid (**1**) by the recombinant (A) *Escherichia coli* BL21(DE3) pACYC-ADH, pJOE-BVMO, (B) *E. coli* BL21(DE3) pACYC-ADH, pJOE-E6-BVMO, (C) *E. coli* BL21(DE3) pACYC-ADH, pJOE-K6-BVMO, and (D) *E. coli* BL21(DE3) pACYC-ADH, pJOE-Ub-BVMO. The recombinant cells express not only the BVMOs but also the alcohol dehydrogenase (ADH) from *Micrococcus luteus*. The target gene expression was induced by adding 0.1 mM IPTG and 2 g/L rhamnose at 20^o^C at the exponential growth phase (cell density: 0.2 g dry cells/L). The biotransformation was initiated at the stationary growth phase (cell density: 3 g dry cells/L) by adding 15 mM ricinoleic acid and 0.5 g/L Tween80 to the culture broth.

**Fig. S2**. Designed biotransformation pathway. 10-Hydroxyoctadecanoic acid (**6**) is enzymatically converted into the ester **8**, which can be hydrolyzed into *n*-nonanoic acid (**9**) and 9-hydroxynonanoic acid (**10**). Adopted from our previous study^1^.

A

B

**Fig. S3**. Time course of the biotransformation of 10-hydroxyoctadecanoic acid (**6**) by the recombinant (A) *E. coli* BL21(DE3) pACYC-ADH, pJOE-BVMO and (B) *E. coli* BL21(DE3) pACYC-ADH, pJOE-E6-BVMO. The recombinant cells express not only the BVMOs but also the ADH from *M. luteus*. The target gene expression was induced by adding 0.1 mM IPTG and 2 g/L rhamnose at 20^o^C (solid lines), 25^o^C (dashed lines), and 30^o^C (dotted lines) at the exponential growth phase (cell density: 0.2 g dry cells/L). The biotransformation was initiated at the stationary growth phase (cell density: 3 g dry cells/L) by adding 6 mM 10-hydroxyoctadecanoic acid and 0.5 g/L Tween80 to the culture broth. The average values of three-independent experiments were used for the plotting. The standard deviation was less than 10%.

A


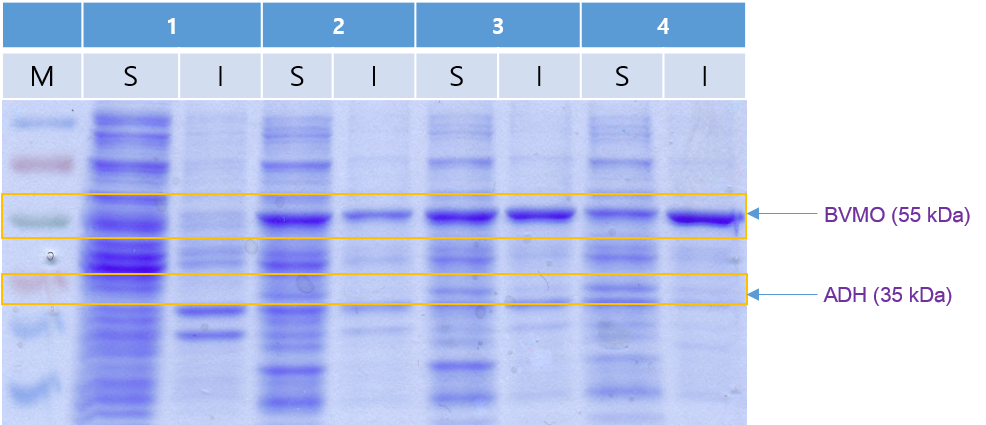


B


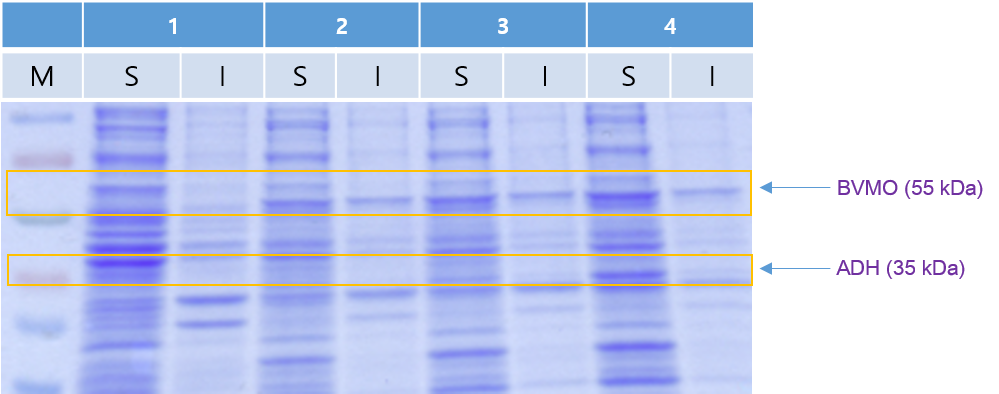


**Fig. S4**. SDS-PAGE analysis of the proteins expressed in the recombinant (A) *E. coli* BL21(DE3) pACYC-ADH, pJOE-BVMO and (B) *E. coli* BL21(DE3) pACYC-ADH, pJOE-E6-BVMO. Expression of the cascade enzymes (i.e., ADH and BVMOs) was induced at 20°C (lane 2), 25°C (lane 3), and 30°C (lane 4). Lane 1 indicates the proteins expressed at 20°C in wildtype *E. coli* BL21(DE3). M, S, and I indicate marker, soluble fraction, and insoluble fraction of the cell lysates, respectively.

A


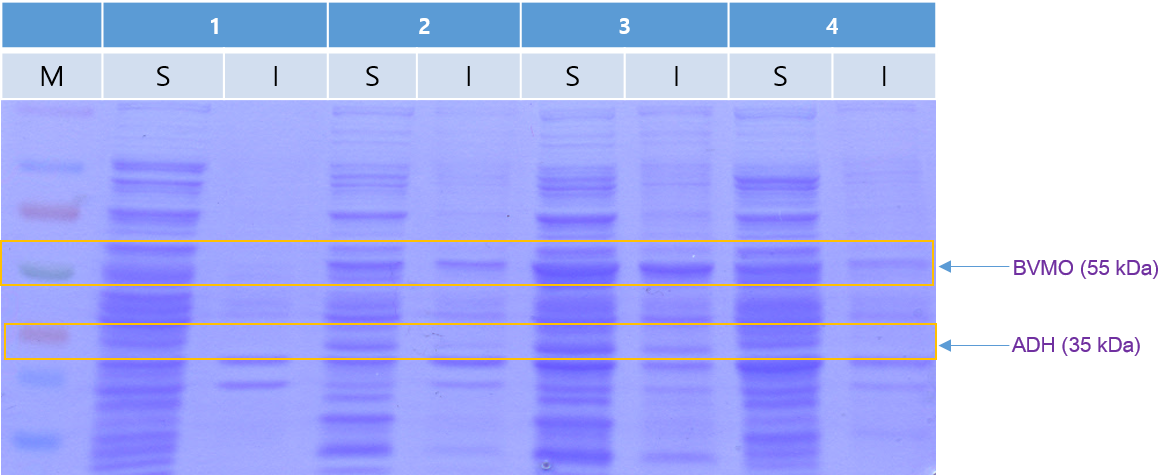


B

**Fig. S5**. (A) SDS-PAGE analysis of the proteins expressed in wildtype *E. coli* BL21(DE3) (lane 1), in the recombinant *E. coli* BL21(DE3) pACYC-ADH, pJOE-E6-BVMO (lane 2), *E. coli* BL21(DE3) pACYC-ADH, pJOE-E6-BVMO_opt_ (lane 3), and *E. coli* BL21(DE3) pAPTm-E6-BVMO_opt_-ADH (lane 4). The cascade enzymes (i.e., ADH and BVMOs) were expressed at 25°C. M, S, and I indicate marker, soluble fraction, and insoluble fraction of the cell lysates, respectively.

(B) Time course of the biotransformation of ricinoleic acid (**1**) by the recombinant *E. coli* BL21(DE3) pACYC-ADH, pJOE-E6-BVMO_opt_. The biotransformation was initiated at the stationary growth phase (cell density: 3 g dry cells/L) by adding 15 mM ricinoleic acid and 0.5 g/L Tween80 to the culture broth.


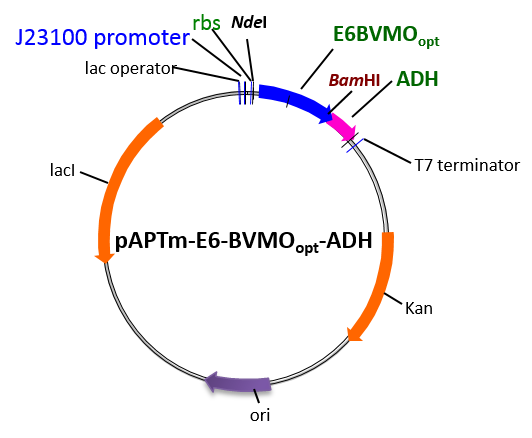


**Fig. S6**. Map of pAPTm-E6-BVMO_opt_-ADH plasmid.

A

B

**Fig. S7**. Separation of the ester (**3**) products from the reaction medium. The ester products, which were produced in the Biotransformation 2 (Table 1), were isolated from the reaction medium, after separation of the cell mass from the culture broth. The ester was extracted from the cell mass and supernatant fraction, respectively, by using the same volume of ethylacetate three times. The upper panel (A) indicates the extraction rate of ester (**3**) at each round of extraction. The lower panel (B) indicates the ester contents, which were recovered from the cell mass and supernatant fraction, respectively. Overall, over 95% of the ester products isolated were found in the cell mass fraction.


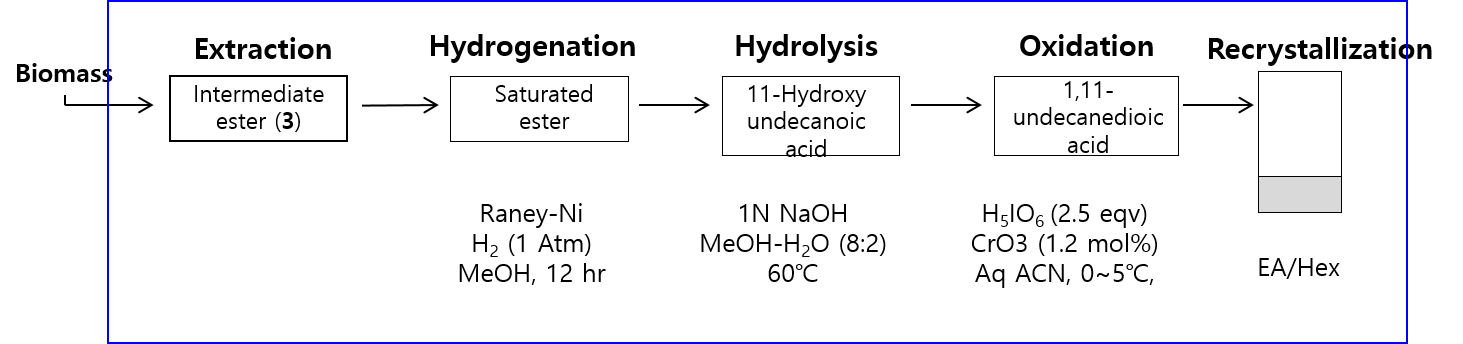


**Fig S8**. Synthesis of 1,11-undecanedioic acid from the crude ester products. The crude ester products isolated were subjected to hydrogenation of the double bond in carbon skeleton, hydrolysis of the ester bond, and oxidation of the resulting hydroxyl group to carboxylic acid, as described previously^2^. See the conversion yields and recovery yields in the Table S3.

**Fig. S9**. Growth culture of the recombinant *E. coli* BL21(DE3) pAPTm-E6-BVMO_opt_-ADH in a 70 L bioreactor.

A


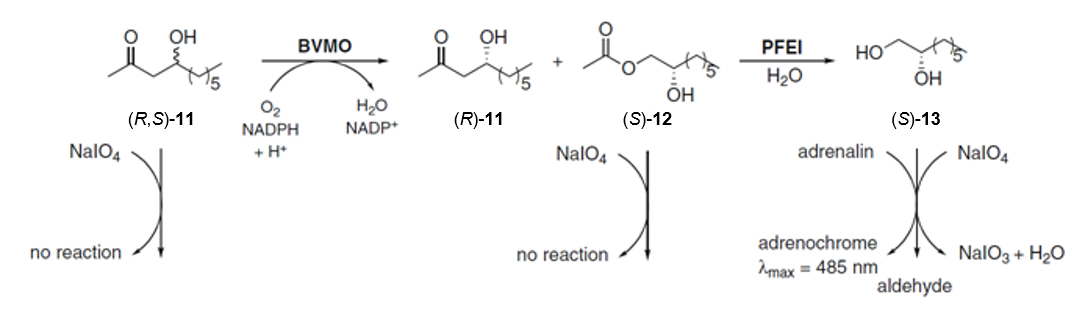


B


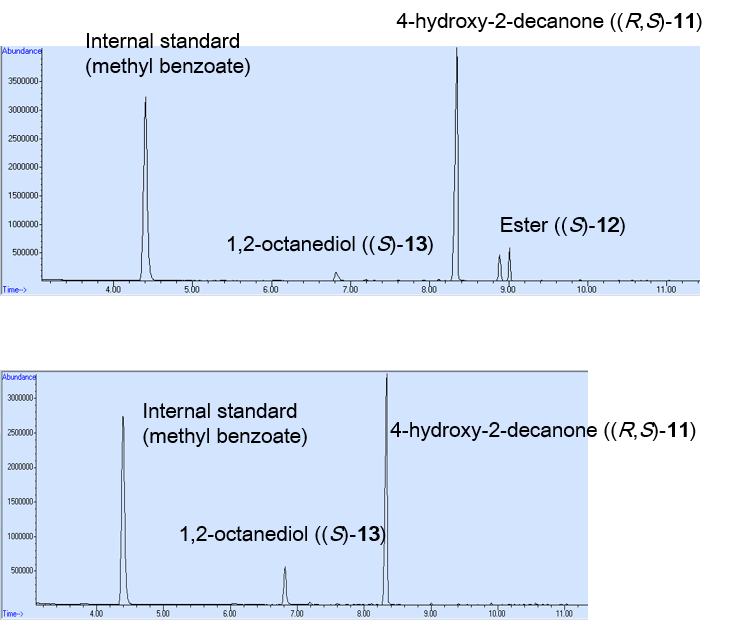


**Fig. S10**. (A) Principle of the adrenalin assay for the detection of BVMO activity^3^ and (B) GC/MS analysis of (*R*,*S*)-**11**, (*S*)-**12**, and (*S*)-**13** produced by the recombinant *E. coli* BL21(DE3) expressing the variants of *P. putida* KT2440 BVMO. The ester (i.e., (*S*)-**12**), which was produced by the BVMOs, is hydrolyzed by an esterase (PFEI) yielding the 1,2-diol (**13**). Only this 1,2-diol reacts with NaIO_4_, and the remaining amount of NaIO_4_ is subsequently back-titrated with adrenalin yielding the chromophore adrenochrome. (B) The GC/MS analysis of 4-hydroxy-2-decanone ((*R*,*S*)-**11**) biotransformation products (upper panel) by the recombinant *E. coli* BL21(DE3) expressing the BVMO of *P. putida* KT2440. The biotransformation products were then subjected to the esterase reaction generating 1,2-octanediol ((*S*)-**13**) (lower panel).

A


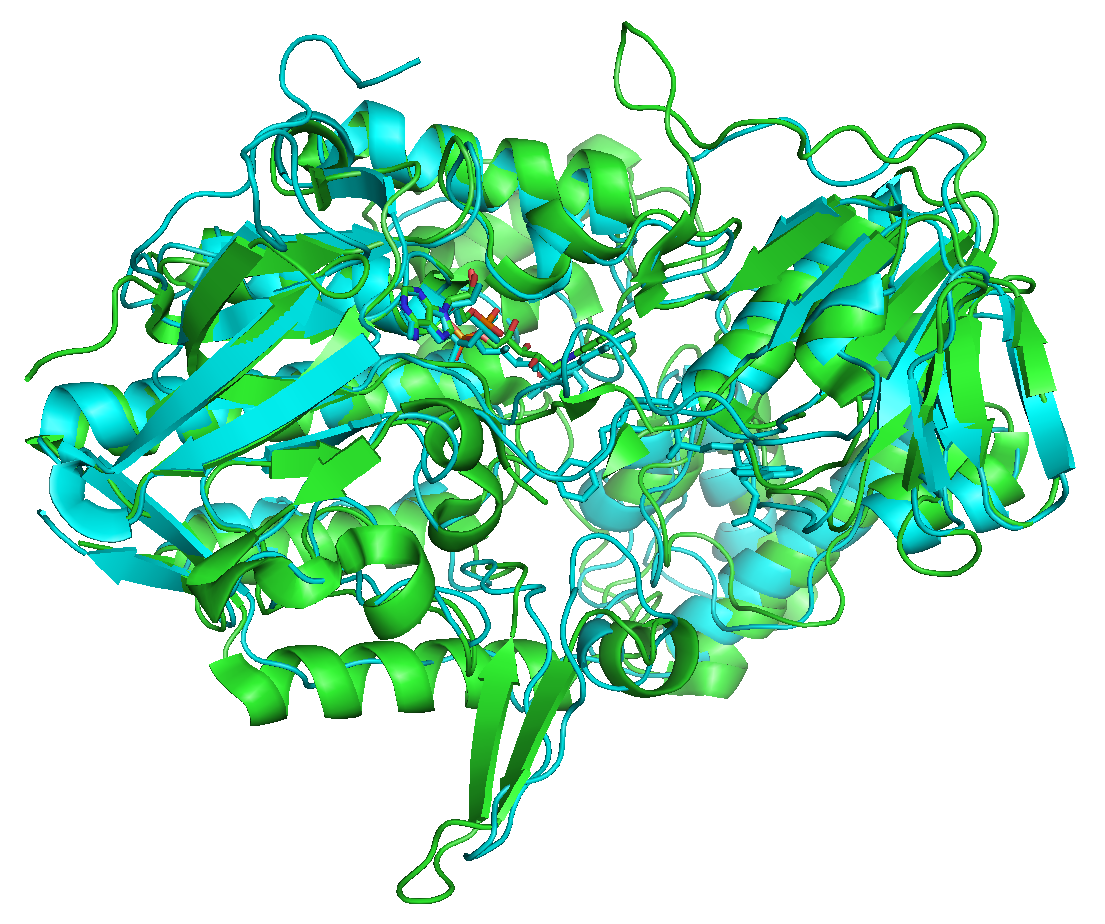


B


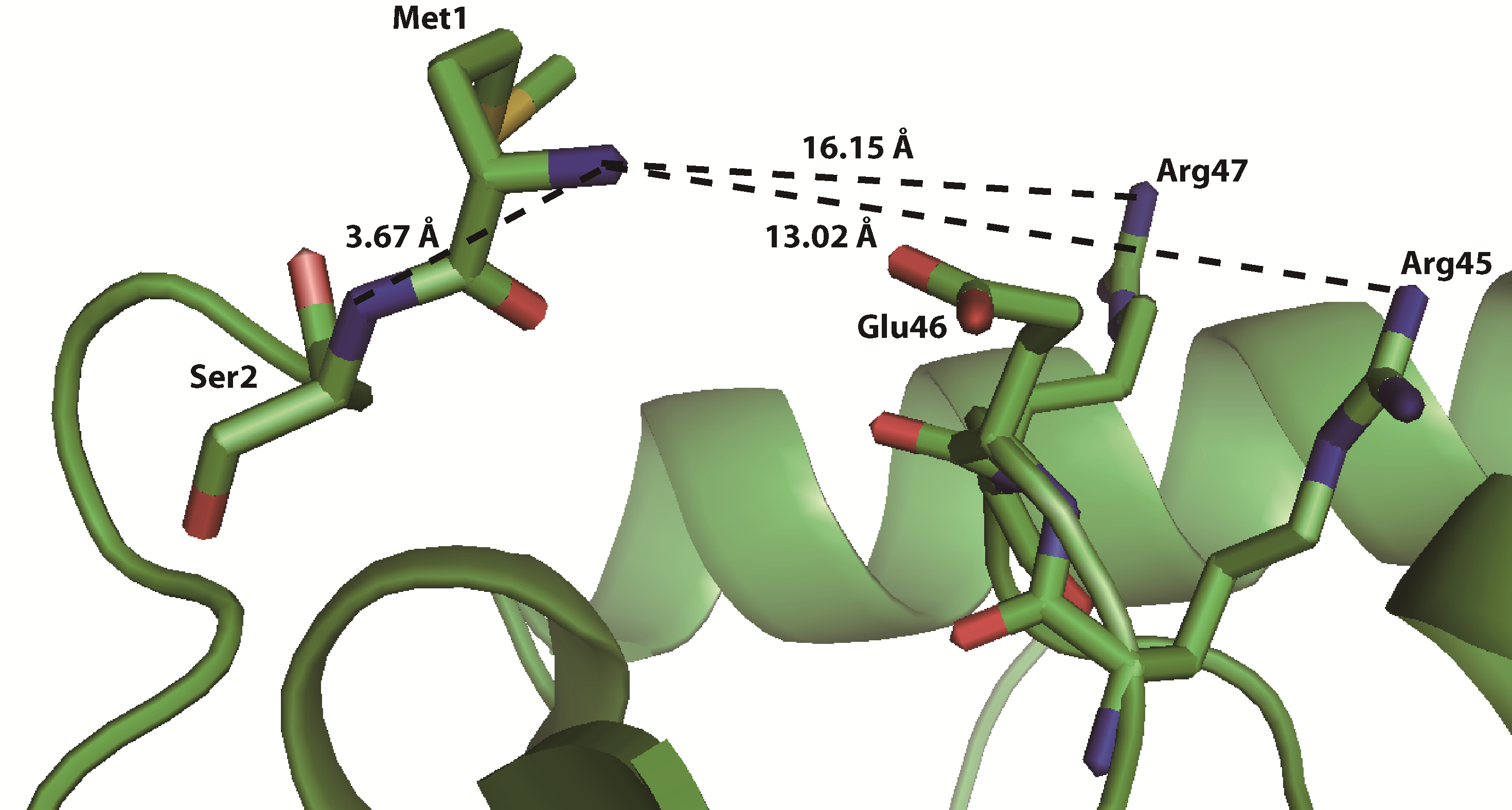


**Fig. S11**. (A) Homology model of BVMO of *P. putida* KT2440 and (B) its partial structure near the N-terminal. Figures were drawn using PyMol. (A) Superposed structure of homology model of BVMO of *P. putida* KT2440 and 3UOV (monooxygenase of *P. putida* ATCC 17453, best template). Green and cyan indicate 3UOV and homology model of *P. putida* KT2440, respectively. (B) Partial structure of BVMO of *P. putida* KT2440 near the N-terminal. Arg45 and Arg47 are located near the N-terminal. Because an approximate length of the stretch of E6 tag is 13.67 Å, the first glutamate residue of E6 tag may interact with Arg45 and Arg47 to build salt bridges. In detail, the length of the stretch of α-helix is 5.4 Å per 3.6 residues. Also, according to the molecular simulation of glutamic acid using Chem3D (PerkinElmer, Waltham, MA, USA), the length between Cα atom and OE1 and OE2 (oxygen atoms of carboxylic group in residue) are 4.64 Å and 4.70 Å, respectively. Therefore, the length of E6 tag are approximately 13.67 Å (5.4/3.6×6 + (4.64+4.70)/2)). If the approximate length of E6 tag was considered, the first glutamate of E6 tag may locate near the Arg45 and Arg47 residues because the distances between Arg45 or Arg47 and N-terminal of BVMO are 13.02 Å and 16.15 Å, respectively. Therefore, if the E6 tag stretches 13.67 Å from N-terminal of BVMO, the first glutamate is laid in a position sufficient to build hydrogen bonding with Arg45 and Arg47.

**Experiment 1**. The rational engineering of the BVMO based on Rosetta prediction.

**Selection of the mutant candidates**

*In silico* single mutation was performed one by one for whole residues and corresponding energy change was predicted by using Rosetta^4^. The residues located less than 5 Å from the cofactors were regarded as active sites and thus kept unchanged. Only mutations with an energy change (ΔΔG) above a threshold are selected as final point mutations (Table S4).

**Table S4**. ΔΔG values of top five stabilizing mutations.

| Candidates | E6-BVMO | K37F | D153M | K252G | K266S | G415E |
| --- | --- | --- | --- | --- | --- | --- |
| ΔΔG (REU) | 0 | -6.287 | -3.743 | -5.953 | -5.634 | -2.545 |

**Construction of the mutants**

The mutants were constructed using PCR-based site-directed mutagenesis. The primers used were listed in Table S5.

**Table S5**. Primers used to construct the mutants

| Mutation |  | Primer sequence |
| --- | --- | --- |
| K37F | forward | 5’-TTTACCTTCGCTATTCTGGAGTCTCGCG-3’ |
|  | backward | 5’-GTTCGGCTGGTTACGACGCAGATAAG-3’ |
| D153M | forward | 5’-ATGCAGGGCTTCAGCCCGCGTTTCGAAGGTA-3’ |
|  | backward | 5’-GTAACGGTAGTAACCACCCGCAGAGAACAG-3’ |
| K252G | forward | 5’-GGCATCACTCTGGCATTCTGGGGCTTTT-3’ |
|  | backward | 5’-AGCGTTTTTATAACGGGTCAGAGAATAGGCGG-3’ |
| K266S | forward | 5’-AGCCTGAGCAAAAAACTGTTGCTGTGG-3’ |
|  | backward | 5’-CGGGAAGCGCTGGCAAAAGCCCCAGAATGC-3’ |
| G415E | forward | 5’-GAACTGATGGAACGTGAAGGCTACAACGTC-3’ |
|  | backward | 5’-CAGCAGACGGCAGAAGTGGTCACACAG-3’ |

**SDS-PAGE analysis of the variants**

The native E6-BVMO and E6-BVMO variants listed in the Table S4 were cloned into the pETDuet vector and transformed into the *E. coli* BL21(DE3) pACYC-ADH. The proteins expressed in the *E. coli* recombinants were subjected to the SDS-PAGE analysis (Fig. S11). The soluble expression level of the BVMO variants expressed in the *E. coli* BL21(DE3) pACYC-ADH, pETDuet-E6-BVMO_K252G_, *E. coli* BL21(DE3) pACYC-ADH, pETDuet-E6-BVMO_K266S_, and *E. coli* BL21(DE3) pACYC-ADH, pETDuet-E6-BVMO_G415E_ was greater than that of the *E. coli* BL21(DE3) pACYC-ADH, pETDuet-E6-BVMO.


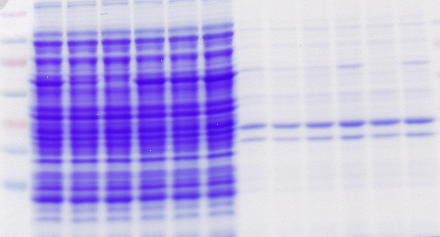


M123456

1’ 2’ 3’ 4’ 5’ 6’

BVMO (55 kDa)

**Fig. S11**. SDS-PAGE analysis of the proteins expressed in the recombinant *E. coli* BL21(DE3) pACYC-ADH, pETDuet-E6-BVMO (lane 1 and 1’), *E. coli* BL21(DE3) pACYC-ADH, pETDuet-E6-BVMO_K37F_ (lane 2 and 2’), *E. coli* BL21(DE3) pACYC-ADH, pETDuet-E6-BVMO_D153M_ (lane 3 and 3’), *E. coli* BL21(DE3) pACYC-ADH, pETDuet-E6-BVMO_K252G_ (lane 4 and 4’), *E. coli* BL21(DE3) pACYC-ADH, pETDuet-E6-BVMO_K266S_ (lane 5 and 5’), *E. coli* BL21(DE3) pACYC-ADH, pETDuet-E6-BVMO_G415E_ (lane 6 and 6’). Expression of the cascade enzymes (i.e., ADH and BVMOs) was induced at 20°C. Lane 1, 2, 3, 4, 5, and 6 indicate soluble fraction. Lane 1’, 2’, 3’, 4’, 5’, and 6’ indicate insoluble fraction.

**Whole cell biotransformation**

The recombinant *E. coli* pACYC-ADH, pETDuet-E6-BVMOs were cultivated in Riesenberg medium containing 10 g/L glucose. Biotransformation was initiated at the stationary growth phase, after induction of gene expression with 0.1 mM IPTG. After changing the pH and culture temperature of the culture broth to 8.0 and to 35°C, respectively, 15 mM ricinoleic acid and 0.5 g/L Tween80 were added to culture broth containing 3 g dry cells/L.

The biotransformation dynamics was very similar to the biotransformation of ricinoleic acid (**1**), which was shown in Fig. S5. The ester (**3**) concentration, which was determined 7 h after initiation of the biotransformation, increased to 9.3 mM in the reaction medium (Table S6). The recombinant *E. coli* BL21(DE3) pACYC-ADH, pETDuet-E6-BVMO_G425E_ showed the highest ester concentration. However, this value is almost identical to that of the recombinant *E. coli* BL21(DE3) pACYC-ADH, pETDuet-E6-BVMO, which express the E6-BVMO.

**Table S6**. The biotransformation results of *E. coli* BL21(DE3) pACYC-ADH, pETDuet-E6-BVMOs.

|  | **E6-BVMO** | **K37F** | **D153M** | **K252G** | **K266S** | **G415E** |
| --- | --- | --- | --- | --- | --- | --- |
| Ester (**3**) concentration  (mM)^a^ | 9.3 | 3.7 | 6.5 | 6.7 | 7.7 | 9.4 |

^a^ The ester (**3**) concentration, which was determined by GC/MS, 7 h after initiation of the biotransformation of ricinoleic acid (**1**) at the conditions identical to the biotransformations shown in Fig. S5. In brief, the target gene expression was induced by adding 0.1 mM IPTG at the exponential growth phase (cell density: 0.2 g dry cells/L). The biotransformation was initiated at the stationary growth phase (cell density: 3 g dry cells/L) by adding 15 mM ricinoleic acid and 0.5 g/L Tween80 to the culture broth.

**Conclusion**

Some of the E6-BVMO variants were more highly expressed in a soluble form in the *E. coli* BL21(DE3) pACYC-ADH as compared to the native enzyme-based E6-BVMO. However, the ricinoleic acid biotransformation activity of the *E. coli* BL21(DE3) pACYC-ADH, pETDuet-E6-BVMO_variants_ was not significantly greater than that of *E. coli* BL21(DE3) pACYC-ADH, pETDuet-E6-BVMO_native_. Thereby, it was assumed that the E6-BVMO is quite active and stable under reaction conditions.

**References**

1. Song, J.-W. *et al.* Multistep enzymatic synthesis of long-chain a,w-dicarboxylic and ω-hydroxycarboxylic acids from renewable fatty acids and plant oils. *Angew. Chem. Int. Ed. Engl.* **52**, 2534-2537 (2013).

2. Jang, H.-Y., Singha, K., Kim, H.-H., Kwon, Y.-U. & Park, J.-B. Chemo-enzymatic synthesis of 11-hydroxyundecanoic acid and 1,11-undecanedioic acid from ricinoleic acid. *Green Chem.* **18**, 1089-1095 (2016).

3. Kirschner, A. & Bornscheuer, U. T. Directed evolution of a Baeyer-Villiger monooxygenase to enhance enantioselectivity. *Appl. Microbiol. Biotechnol.* **81**, 465-472 (2008).

4. Kellogg, E. H., Leaver-Fay, A. & Baker, D. Role of conformational sampling in computing mutation-induced changes in protein structure and stability. *Proteins* **79**, 830-838 (2011).

**Appendix**. Atom coordinates of homology model of BVMO from *P. putida* KT2440 (Uniprot accession number Q88J44).

ATOM 1 N MET 1 17.079 48.339 54.733 1.00 0.00 N

ATOM 2 CA MET 1 17.224 47.452 55.909 1.00 0.00 C

ATOM 3 C MET 1 18.128 46.303 55.616 1.00 0.00 C

ATOM 4 O MET 1 18.405 45.993 54.458 1.00 0.00 O

ATOM 5 CB MET 1 15.846 46.926 56.348 1.00 0.00 C

ATOM 6 CG MET 1 15.052 46.264 55.219 1.00 0.00 C

ATOM 7 SD MET 1 15.708 44.672 54.642 1.00 0.00 S

ATOM 8 CE MET 1 14.436 44.435 53.368 1.00 0.00 C

ATOM 9 N SER 2 18.637 45.652 56.679 1.00 0.00 N

ATOM 10 CA SER 2 19.522 44.543 56.489 1.00 0.00 C

ATOM 11 C SER 2 18.685 43.378 56.089 1.00 0.00 C

ATOM 12 O SER 2 17.501 43.311 56.414 1.00 0.00 O

ATOM 13 CB SER 2 20.293 44.148 57.759 1.00 0.00 C

ATOM 14 OG SER 2 21.147 43.046 57.493 1.00 0.00 O

ATOM 15 N SER 3 19.283 42.421 55.355 1.00 0.00 N

ATOM 16 CA SER 3 18.501 41.293 54.956 1.00 0.00 C

ATOM 17 C SER 3 18.343 40.395 56.136 1.00 0.00 C

ATOM 18 O SER 3 19.118 40.458 57.088 1.00 0.00 O

ATOM 19 CB SER 3 19.132 40.468 53.820 1.00 0.00 C

ATOM 20 OG SER 3 18.290 39.373 53.490 1.00 0.00 O

ATOM 21 N HIS 4 17.310 39.531 56.097 1.00 0.00 N

ATOM 22 CA HIS 4 17.072 38.638 57.191 1.00 0.00 C

ATOM 23 C HIS 4 18.278 37.775 57.329 1.00 0.00 C

ATOM 24 O HIS 4 18.852 37.666 58.411 1.00 0.00 O

ATOM 25 CB HIS 4 15.867 37.713 56.950 1.00 0.00 C

ATOM 26 CG HIS 4 15.612 36.774 58.091 1.00 0.00 C

ATOM 27 CD2 HIS 4 15.963 35.468 58.244 1.00 0.00 C

ATOM 28 ND1 HIS 4 14.938 37.118 59.242 1.00 0.00 N

ATOM 29 CE1 HIS 4 14.915 36.012 60.028 1.00 0.00 C

ATOM 30 NE2 HIS 4 15.525 34.985 59.465 1.00 0.00 N

ATOM 31 N THR 5 18.710 37.148 56.220 1.00 0.00 N

ATOM 32 CA THR 5 19.901 36.360 56.296 1.00 0.00 C

ATOM 33 C THR 5 20.956 37.186 55.650 1.00 0.00 C

ATOM 34 O THR 5 21.779 36.697 54.879 1.00 0.00 O

ATOM 35 CB THR 5 19.800 35.068 55.538 1.00 0.00 C

ATOM 36 CG2 THR 5 19.523 35.371 54.054 1.00 0.00 C

ATOM 37 OG1 THR 5 21.000 34.322 55.682 1.00 0.00 O

ATOM 38 N ALA 6 20.950 38.486 55.983 1.00 0.00 N

ATOM 39 CA ALA 6 21.882 39.405 55.425 1.00 0.00 C

ATOM 40 C ALA 6 23.268 39.081 55.878 1.00 0.00 C

ATOM 41 O ALA 6 24.216 39.173 55.102 1.00 0.00 O

ATOM 42 CB ALA 6 21.598 40.863 55.822 1.00 0.00 C

ATOM 43 N LEU 7 23.421 38.667 57.148 1.00 0.00 N

ATOM 44 CA LEU 7 24.737 38.514 57.696 1.00 0.00 C

ATOM 45 C LEU 7 25.544 37.549 56.892 1.00 0.00 C

ATOM 46 O LEU 7 25.127 36.439 56.565 1.00 0.00 O

ATOM 47 CB LEU 7 24.737 38.027 59.157 1.00 0.00 C

ATOM 48 CG LEU 7 24.049 39.006 60.126 1.00 0.00 C

ATOM 49 CD1 LEU 7 22.553 39.151 59.800 1.00 0.00 C

ATOM 50 CD2 LEU 7 24.302 38.619 61.592 1.00 0.00 C

ATOM 51 N PRO 8 26.711 38.027 56.550 1.00 0.00 N

ATOM 52 CA PRO 8 27.705 37.249 55.860 1.00 0.00 C

ATOM 53 C PRO 8 28.393 36.409 56.891 1.00 0.00 C

ATOM 54 O PRO 8 28.387 36.794 58.058 1.00 0.00 O

ATOM 55 CB PRO 8 28.659 38.254 55.219 1.00 0.00 C

ATOM 56 CG PRO 8 28.400 39.567 55.981 1.00 0.00 C

ATOM 57 CD PRO 8 26.929 39.454 56.403 1.00 0.00 C

ATOM 58 N VAL 9 29.003 35.274 56.502 1.00 0.00 N

ATOM 59 CA VAL 9 29.658 34.454 57.484 1.00 0.00 C

ATOM 60 C VAL 9 31.079 34.896 57.612 1.00 0.00 C

ATOM 61 O VAL 9 31.652 35.441 56.672 1.00 0.00 O

ATOM 62 CB VAL 9 29.656 32.993 57.156 1.00 0.00 C

ATOM 63 CG1 VAL 9 30.476 32.799 55.877 1.00 0.00 C

ATOM 64 CG2 VAL 9 30.199 32.212 58.365 1.00 0.00 C

ATOM 65 N GLU 10 31.671 34.738 58.814 1.00 0.00 N

ATOM 66 CA GLU 10 33.038 35.138 58.956 1.00 0.00 C

ATOM 67 C GLU 10 33.984 34.241 58.191 1.00 0.00 C

ATOM 68 O GLU 10 34.815 34.780 57.463 1.00 0.00 O

ATOM 69 CB GLU 10 33.479 35.373 60.423 1.00 0.00 C

ATOM 70 CG GLU 10 32.926 34.406 61.474 1.00 0.00 C

ATOM 71 CD GLU 10 31.459 34.738 61.707 1.00 0.00 C

ATOM 72 OE1 GLU 10 30.980 35.752 61.132 1.00 0.00 O

ATOM 73 OE2 GLU 10 30.797 33.982 62.468 1.00 0.00 O

ATOM 74 N PRO 11 33.953 32.927 58.269 1.00 0.00 N

ATOM 75 CA PRO 11 34.865 32.177 57.438 1.00 0.00 C

ATOM 76 C PRO 11 34.208 31.818 56.145 1.00 0.00 C

ATOM 77 O PRO 11 32.985 31.688 56.128 1.00 0.00 O

ATOM 78 CB PRO 11 35.287 30.946 58.240 1.00 0.00 C

ATOM 79 CG PRO 11 34.201 30.808 59.316 1.00 0.00 C

ATOM 80 CD PRO 11 33.734 32.253 59.536 1.00 0.00 C

ATOM 81 N LEU 12 34.991 31.631 55.062 1.00 0.00 N

ATOM 82 CA LEU 12 34.417 31.245 53.802 1.00 0.00 C

ATOM 83 C LEU 12 35.492 30.701 52.914 1.00 0.00 C

ATOM 84 O LEU 12 36.619 31.191 52.918 1.00 0.00 O

ATOM 85 CB LEU 12 33.771 32.414 53.038 1.00 0.00 C

ATOM 86 CG LEU 12 32.375 32.794 53.549 1.00 0.00 C

ATOM 87 CD1 LEU 12 31.854 34.066 52.861 1.00 0.00 C

ATOM 88 CD2 LEU 12 31.411 31.610 53.358 1.00 0.00 C

ATOM 89 N ASP 13 35.170 29.640 52.143 1.00 0.00 N

ATOM 90 CA ASP 13 36.113 29.120 51.196 1.00 0.00 C

ATOM 91 C ASP 13 36.295 30.174 50.150 1.00 0.00 C

ATOM 92 O ASP 13 37.417 30.503 49.768 1.00 0.00 O

ATOM 93 CB ASP 13 35.611 27.854 50.474 1.00 0.00 C

ATOM 94 CG ASP 13 35.561 26.700 51.467 1.00 0.00 C

ATOM 95 OD1 ASP 13 35.876 26.934 52.664 1.00 0.00 O

ATOM 96 OD2 ASP 13 35.214 25.566 51.043 1.00 0.00 O

ATOM 97 N VAL 14 35.171 30.741 49.664 1.00 0.00 N

ATOM 98 CA VAL 14 35.239 31.770 48.668 1.00 0.00 C

ATOM 99 C VAL 14 34.190 32.804 48.886 1.00 0.00 C

ATOM 100 O VAL 14 33.095 32.533 49.378 1.00 0.00 O

ATOM 101 CB VAL 14 35.122 31.277 47.256 1.00 0.00 C

ATOM 102 CG1 VAL 14 36.491 30.773 46.796 1.00 0.00 C

ATOM 103 CG2 VAL 14 34.089 30.142 47.244 1.00 0.00 C

ATOM 104 N LEU 15 34.544 34.054 48.532 1.00 0.00 N

ATOM 105 CA LEU 15 33.616 35.138 48.599 1.00 0.00 C

ATOM 106 C LEU 15 33.466 35.590 47.187 1.00 0.00 C

ATOM 107 O LEU 15 34.454 35.720 46.465 1.00 0.00 O

ATOM 108 CB LEU 15 34.129 36.349 49.394 1.00 0.00 C

ATOM 109 CG LEU 15 33.129 37.512 49.518 1.00 0.00 C

ATOM 110 CD1 LEU 15 31.891 37.096 50.328 1.00 0.00 C

ATOM 111 CD2 LEU 15 33.809 38.768 50.085 1.00 0.00 C

ATOM 112 N ILE 16 32.217 35.815 46.746 1.00 0.00 N

ATOM 113 CA ILE 16 32.009 36.280 45.412 1.00 0.00 C

ATOM 114 C ILE 16 31.378 37.625 45.503 1.00 0.00 C

ATOM 115 O ILE 16 30.431 37.837 46.260 1.00 0.00 O

ATOM 116 CB ILE 16 31.126 35.391 44.590 1.00 0.00 C

ATOM 117 CG1 ILE 16 30.981 35.937 43.162 1.00 0.00 C

ATOM 118 CG2 ILE 16 29.810 35.173 45.350 1.00 0.00 C

ATOM 119 CD1 ILE 16 30.406 34.914 42.186 1.00 0.00 C

ATOM 120 N MET 17 31.923 38.590 44.741 1.00 0.00 N

ATOM 121 CA MET 17 31.373 39.906 44.802 1.00 0.00 C

ATOM 122 C MET 17 30.604 40.119 43.526 1.00 0.00 C

ATOM 123 O MET 17 31.171 40.060 42.437 1.00 0.00 O

ATOM 124 CB MET 17 32.452 40.998 44.894 1.00 0.00 C

ATOM 125 CG MET 17 33.302 40.882 46.161 1.00 0.00 C

ATOM 126 SD MET 17 34.662 42.082 46.286 1.00 0.00 S

ATOM 127 CE MET 17 35.688 41.317 45.001 1.00 0.00 C

ATOM 128 N GLY 18 29.277 40.353 43.635 1.00 0.00 N

ATOM 129 CA GLY 18 28.431 40.657 42.509 1.00 0.00 C

ATOM 130 C GLY 18 27.505 39.501 42.157 1.00 0.00 C

ATOM 131 O GLY 18 27.912 38.348 42.062 1.00 0.00 O

ATOM 132 N ALA 19 26.199 39.839 42.053 1.00 0.00 N

ATOM 133 CA ALA 19 24.936 39.189 41.726 1.00 0.00 C

ATOM 134 C ALA 19 24.577 38.995 40.271 1.00 0.00 C

ATOM 135 O ALA 19 23.477 38.509 40.006 1.00 0.00 O

ATOM 136 CB ALA 19 23.735 39.885 42.385 1.00 0.00 C

ATOM 137 N GLY 20 25.321 39.555 39.295 1.00 0.00 N

ATOM 138 CA GLY 20 24.872 39.441 37.924 1.00 0.00 C

ATOM 139 C GLY 20 25.071 38.036 37.433 1.00 0.00 C

ATOM 140 O GLY 20 25.156 37.095 38.220 1.00 0.00 O

ATOM 141 N VAL 21 25.148 37.869 36.094 1.00 0.00 N

ATOM 142 CA VAL 21 25.231 36.569 35.490 1.00 0.00 C

ATOM 143 C VAL 21 26.400 35.822 36.066 1.00 0.00 C

ATOM 144 O VAL 21 26.292 34.635 36.373 1.00 0.00 O

ATOM 145 CB VAL 21 25.383 36.622 33.994 1.00 0.00 C

ATOM 146 CG1 VAL 21 24.093 37.212 33.398 1.00 0.00 C

ATOM 147 CG2 VAL 21 26.649 37.422 33.643 1.00 0.00 C

ATOM 148 N SER 22 27.549 36.497 36.232 1.00 0.00 N

ATOM 149 CA SER 22 28.754 35.867 36.700 1.00 0.00 C

ATOM 150 C SER 22 28.674 35.388 38.123 1.00 0.00 C

ATOM 151 O SER 22 28.975 34.228 38.403 1.00 0.00 O

ATOM 152 CB SER 22 29.965 36.804 36.557 1.00 0.00 C

ATOM 153 OG SER 22 31.166 36.081 36.767 1.00 0.00 O

ATOM 154 N GLY 23 28.258 36.265 39.058 1.00 0.00 N

ATOM 155 CA GLY 23 28.250 35.961 40.470 1.00 0.00 C

ATOM 156 C GLY 23 27.319 34.829 40.757 1.00 0.00 C

ATOM 157 O GLY 23 27.627 33.944 41.554 1.00 0.00 O

ATOM 158 N ILE 24 26.150 34.835 40.101 1.00 0.00 N

ATOM 159 CA ILE 24 25.116 33.873 40.345 1.00 0.00 C

ATOM 160 C ILE 24 25.608 32.495 40.014 1.00 0.00 C

ATOM 161 O ILE 24 25.409 31.555 40.783 1.00 0.00 O

ATOM 162 CB ILE 24 23.923 34.144 39.474 1.00 0.00 C

ATOM 163 CG1 ILE 24 23.281 35.488 39.848 1.00 0.00 C

ATOM 164 CG2 ILE 24 22.966 32.949 39.545 1.00 0.00 C

ATOM 165 CD1 ILE 24 22.246 35.964 38.831 1.00 0.00 C

ATOM 166 N GLY 25 26.255 32.335 38.846 1.00 0.00 N

ATOM 167 CA GLY 25 26.721 31.044 38.426 1.00 0.00 C

ATOM 168 C GLY 25 27.805 30.566 39.339 1.00 0.00 C

ATOM 169 O GLY 25 27.860 29.387 39.689 1.00 0.00 O

ATOM 170 N ALA 26 28.707 31.481 39.739 1.00 0.00 N

ATOM 171 CA ALA 26 29.834 31.113 40.544 1.00 0.00 C

ATOM 172 C ALA 26 29.356 30.561 41.848 1.00 0.00 C

ATOM 173 O ALA 26 29.872 29.553 42.332 1.00 0.00 O

ATOM 174 CB ALA 26 30.759 32.302 40.853 1.00 0.00 C

ATOM 175 N ALA 27 28.342 31.210 42.448 1.00 0.00 N

ATOM 176 CA ALA 27 27.853 30.773 43.721 1.00 0.00 C

ATOM 177 C ALA 27 27.306 29.389 43.584 1.00 0.00 C

ATOM 178 O ALA 27 27.591 28.516 44.404 1.00 0.00 O

ATOM 179 CB ALA 27 26.717 31.662 44.256 1.00 0.00 C

ATOM 180 N ALA 28 26.524 29.148 42.517 1.00 0.00 N

ATOM 181 CA ALA 28 25.897 27.871 42.350 1.00 0.00 C

ATOM 182 C ALA 28 26.932 26.800 42.195 1.00 0.00 C

ATOM 183 O ALA 28 26.845 25.749 42.829 1.00 0.00 O

ATOM 184 CB ALA 28 24.991 27.814 41.108 1.00 0.00 C

ATOM 185 N TYR 29 27.958 27.044 41.360 1.00 0.00 N

ATOM 186 CA TYR 29 28.912 26.003 41.097 1.00 0.00 C

ATOM 187 C TYR 29 29.700 25.690 42.334 1.00 0.00 C

ATOM 188 O TYR 29 29.908 24.525 42.670 1.00 0.00 O

ATOM 189 CB TYR 29 29.914 26.357 39.986 1.00 0.00 C

ATOM 190 CG TYR 29 30.628 25.099 39.614 1.00 0.00 C

ATOM 191 CD1 TYR 29 31.636 24.588 40.401 1.00 0.00 C

ATOM 192 CD2 TYR 29 30.286 24.428 38.462 1.00 0.00 C

ATOM 193 CE1 TYR 29 32.287 23.430 40.046 1.00 0.00 C

ATOM 194 CE2 TYR 29 30.933 23.269 38.101 1.00 0.00 C

ATOM 195 CZ TYR 29 31.936 22.769 38.894 1.00 0.00 C

ATOM 196 OH TYR 29 32.603 21.580 38.527 1.00 0.00 O

ATOM 197 N LEU 30 30.161 26.729 43.053 1.00 0.00 N

ATOM 198 CA LEU 30 30.991 26.502 44.201 1.00 0.00 C

ATOM 199 C LEU 30 30.209 25.764 45.236 1.00 0.00 C

ATOM 200 O LEU 30 30.717 24.832 45.860 1.00 0.00 O

ATOM 201 CB LEU 30 31.519 27.809 44.816 1.00 0.00 C

ATOM 202 CG LEU 30 32.494 28.551 43.881 1.00 0.00 C

ATOM 203 CD1 LEU 30 33.010 29.852 44.515 1.00 0.00 C

ATOM 204 CD2 LEU 30 33.638 27.628 43.430 1.00 0.00 C

ATOM 205 N ARG 31 28.938 26.153 45.436 1.00 0.00 N

ATOM 206 CA ARG 31 28.155 25.506 46.443 1.00 0.00 C

ATOM 207 C ARG 31 28.022 24.069 46.060 1.00 0.00 C

ATOM 208 O ARG 31 28.172 23.182 46.899 1.00 0.00 O

ATOM 209 CB ARG 31 26.770 26.162 46.618 1.00 0.00 C

ATOM 210 CG ARG 31 25.848 26.107 45.399 1.00 0.00 C

ATOM 211 CD ARG 31 24.941 24.877 45.370 1.00 0.00 C

ATOM 212 NE ARG 31 23.910 25.107 46.423 1.00 0.00 N

ATOM 213 CZ ARG 31 22.825 25.893 46.155 1.00 0.00 C

ATOM 214 NH1 ARG 31 22.654 26.418 44.907 1.00 0.00 N

ATOM 215 NH2 ARG 31 21.920 26.171 47.139 1.00 0.00 N

ATOM 216 N ARG 32 27.751 23.788 44.771 1.00 0.00 N

ATOM 217 CA ARG 32 27.689 22.408 44.394 1.00 0.00 C

ATOM 218 C ARG 32 28.943 22.091 43.634 1.00 0.00 C

ATOM 219 O ARG 32 28.947 22.023 42.406 1.00 0.00 O

ATOM 220 CB ARG 32 26.468 22.075 43.514 1.00 0.00 C

ATOM 221 CG ARG 32 26.363 22.906 42.232 1.00 0.00 C

ATOM 222 CD ARG 32 25.147 22.556 41.373 1.00 0.00 C

ATOM 223 NE ARG 32 25.331 21.158 40.892 1.00 0.00 N

ATOM 224 CZ ARG 32 26.019 20.917 39.738 1.00 0.00 C

ATOM 225 NH1 ARG 32 26.542 21.958 39.027 1.00 0.00 N

ATOM 226 NH2 ARG 32 26.185 19.637 39.296 1.00 0.00 N

ATOM 227 N ASN 33 30.048 21.845 44.364 1.00 0.00 N

ATOM 228 CA ASN 33 31.302 21.551 43.732 1.00 0.00 C

ATOM 229 C ASN 33 31.717 20.228 44.264 1.00 0.00 C

ATOM 230 O ASN 33 31.125 19.771 45.237 1.00 0.00 O

ATOM 231 CB ASN 33 32.408 22.566 44.071 1.00 0.00 C

ATOM 232 CG ASN 33 33.519 22.446 43.036 1.00 0.00 C

ATOM 233 ND2 ASN 33 34.548 23.328 43.149 1.00 0.00 N

ATOM 234 OD1 ASN 33 33.472 21.598 42.147 1.00 0.00 O

ATOM 235 N GLN 34 32.744 19.586 43.659 1.00 0.00 N

ATOM 236 CA GLN 34 33.133 18.281 44.114 1.00 0.00 C

ATOM 237 C GLN 34 33.466 18.441 45.561 1.00 0.00 C

ATOM 238 O GLN 34 32.994 17.659 46.385 1.00 0.00 O

ATOM 239 CB GLN 34 34.340 17.699 43.351 1.00 0.00 C

ATOM 240 CG GLN 34 34.066 17.450 41.867 1.00 0.00 C

ATOM 241 CD GLN 34 35.336 16.883 41.249 1.00 0.00 C

ATOM 242 NE2 GLN 34 35.287 16.590 39.922 1.00 0.00 N

ATOM 243 OE1 GLN 34 36.348 16.711 41.927 1.00 0.00 O

ATOM 244 N PRO 35 34.235 19.427 45.929 1.00 0.00 N

ATOM 245 CA PRO 35 34.276 19.691 47.333 1.00 0.00 C

ATOM 246 C PRO 35 33.086 20.576 47.525 1.00 0.00 C

ATOM 247 O PRO 35 32.732 21.281 46.582 1.00 0.00 O

ATOM 248 CB PRO 35 35.602 20.392 47.609 1.00 0.00 C

ATOM 249 CG PRO 35 36.508 19.923 46.459 1.00 0.00 C

ATOM 250 CD PRO 35 35.530 19.661 45.304 1.00 0.00 C

ATOM 251 N ASN 36 32.437 20.574 48.699 1.00 0.00 N

ATOM 252 CA ASN 36 31.366 21.504 48.860 1.00 0.00 C

ATOM 253 C ASN 36 31.976 22.654 49.583 1.00 0.00 C

ATOM 254 O ASN 36 32.424 22.527 50.721 1.00 0.00 O

ATOM 255 CB ASN 36 30.162 20.951 49.655 1.00 0.00 C

ATOM 256 CG ASN 36 30.605 20.509 51.045 1.00 0.00 C

ATOM 257 ND2 ASN 36 29.839 20.931 52.087 1.00 0.00 N

ATOM 258 OD1 ASN 36 31.607 19.813 51.201 1.00 0.00 O

ATOM 259 N LYS 37 32.052 23.820 48.918 1.00 0.00 N

ATOM 260 CA LYS 37 32.692 24.907 49.590 1.00 0.00 C

ATOM 261 C LYS 37 31.674 25.877 50.056 1.00 0.00 C

ATOM 262 O LYS 37 30.618 26.047 49.449 1.00 0.00 O

ATOM 263 CB LYS 37 33.714 25.699 48.753 1.00 0.00 C

ATOM 264 CG LYS 37 33.314 25.937 47.302 1.00 0.00 C

ATOM 265 CD LYS 37 33.428 24.659 46.479 1.00 0.00 C

ATOM 266 CE LYS 37 34.864 24.132 46.447 1.00 0.00 C

ATOM 267 NZ LYS 37 34.889 22.761 45.898 1.00 0.00 N

ATOM 268 N THR 38 31.979 26.519 51.197 1.00 0.00 N

ATOM 269 CA THR 38 31.115 27.535 51.702 1.00 0.00 C

ATOM 270 C THR 38 31.366 28.721 50.840 1.00 0.00 C

ATOM 271 O THR 38 32.511 29.053 50.537 1.00 0.00 O

ATOM 272 CB THR 38 31.398 27.912 53.128 1.00 0.00 C

ATOM 273 CG2 THR 38 32.803 28.527 53.216 1.00 0.00 C

ATOM 274 OG1 THR 38 30.424 28.834 53.594 1.00 0.00 O

ATOM 275 N PHE 39 30.288 29.385 50.395 1.00 0.00 N

ATOM 276 CA PHE 39 30.497 30.467 49.490 1.00 0.00 C

ATOM 277 C PHE 39 29.497 31.521 49.848 1.00 0.00 C

ATOM 278 O PHE 39 28.357 31.218 50.202 1.00 0.00 O

ATOM 279 CB PHE 39 30.353 29.918 48.050 1.00 0.00 C

ATOM 280 CG PHE 39 30.746 30.855 46.959 1.00 0.00 C

ATOM 281 CD1 PHE 39 31.691 31.835 47.134 1.00 0.00 C

ATOM 282 CD2 PHE 39 30.210 30.671 45.703 1.00 0.00 C

ATOM 283 CE1 PHE 39 32.047 32.659 46.092 1.00 0.00 C

ATOM 284 CE2 PHE 39 30.558 31.487 44.656 1.00 0.00 C

ATOM 285 CZ PHE 39 31.475 32.486 44.854 1.00 0.00 C

ATOM 286 N ALA 40 29.920 32.799 49.823 1.00 0.00 N

ATOM 287 CA ALA 40 29.004 33.852 50.148 1.00 0.00 C

ATOM 288 C ALA 40 29.150 34.897 49.096 1.00 0.00 C

ATOM 289 O ALA 40 30.253 35.161 48.620 1.00 0.00 O

ATOM 290 CB ALA 40 29.277 34.528 51.501 1.00 0.00 C

ATOM 291 N ILE 41 28.029 35.532 48.709 1.00 0.00 N

ATOM 292 CA ILE 41 28.116 36.534 47.692 1.00 0.00 C

ATOM 293 C ILE 41 27.628 37.821 48.259 1.00 0.00 C

ATOM 294 O ILE 41 26.826 37.843 49.194 1.00 0.00 O

ATOM 295 CB ILE 41 27.283 36.273 46.481 1.00 0.00 C

ATOM 296 CG1 ILE 41 27.653 37.173 45.299 1.00 0.00 C

ATOM 297 CG2 ILE 41 25.841 36.462 46.911 1.00 0.00 C

ATOM 298 CD1 ILE 41 26.978 36.689 44.019 1.00 0.00 C

ATOM 299 N LEU 42 28.149 38.940 47.718 1.00 0.00 N

ATOM 300 CA LEU 42 27.749 40.225 48.206 1.00 0.00 C

ATOM 301 C LEU 42 27.323 41.037 47.026 1.00 0.00 C

ATOM 302 O LEU 42 28.002 41.038 46.001 1.00 0.00 O

ATOM 303 CB LEU 42 28.920 40.995 48.836 1.00 0.00 C

ATOM 304 CG LEU 42 29.746 40.125 49.800 1.00 0.00 C

ATOM 305 CD1 LEU 42 30.692 40.973 50.665 1.00 0.00 C

ATOM 306 CD2 LEU 42 28.858 39.153 50.587 1.00 0.00 C

ATOM 307 N GLU 43 26.175 41.738 47.116 1.00 0.00 N

ATOM 308 CA GLU 43 25.840 42.592 46.015 1.00 0.00 C

ATOM 309 C GLU 43 25.297 43.876 46.550 1.00 0.00 C

ATOM 310 O GLU 43 24.600 43.910 47.564 1.00 0.00 O

ATOM 311 CB GLU 43 24.817 42.042 45.020 1.00 0.00 C

ATOM 312 CG GLU 43 23.391 42.023 45.541 1.00 0.00 C

ATOM 313 CD GLU 43 22.536 42.004 44.292 1.00 0.00 C

ATOM 314 OE1 GLU 43 22.579 43.017 43.543 1.00 0.00 O

ATOM 315 OE2 GLU 43 21.849 40.978 44.055 1.00 0.00 O

ATOM 316 N SER 44 25.638 44.978 45.860 1.00 0.00 N

ATOM 317 CA SER 44 25.229 46.292 46.257 1.00 0.00 C

ATOM 318 C SER 44 23.753 46.459 46.052 1.00 0.00 C

ATOM 319 O SER 44 23.084 47.117 46.847 1.00 0.00 O

ATOM 320 CB SER 44 25.933 47.397 45.451 1.00 0.00 C

ATOM 321 OG SER 44 25.493 48.674 45.887 1.00 0.00 O

ATOM 322 N ARG 45 23.207 45.848 44.981 1.00 0.00 N

ATOM 323 CA ARG 45 21.827 46.019 44.611 1.00 0.00 C

ATOM 324 C ARG 45 20.943 45.263 45.550 1.00 0.00 C

ATOM 325 O ARG 45 21.391 44.449 46.357 1.00 0.00 O

ATOM 326 CB ARG 45 21.531 45.535 43.181 1.00 0.00 C

ATOM 327 CG ARG 45 20.108 45.837 42.709 1.00 0.00 C

ATOM 328 CD ARG 45 19.838 47.329 42.514 1.00 0.00 C

ATOM 329 NE ARG 45 18.431 47.471 42.049 1.00 0.00 N

ATOM 330 CZ ARG 45 17.788 48.667 42.180 1.00 0.00 C

ATOM 331 NH1 ARG 45 18.431 49.726 42.755 1.00 0.00 N

ATOM 332 NH2 ARG 45 16.505 48.805 41.737 1.00 0.00 N

ATOM 333 N GLU 46 19.641 45.594 45.488 1.00 0.00 N

ATOM 334 CA GLU 46 18.608 44.991 46.274 1.00 0.00 C

ATOM 335 C GLU 46 18.412 43.576 45.831 1.00 0.00 C

ATOM 336 O GLU 46 18.071 42.712 46.638 1.00 0.00 O

ATOM 337 CB GLU 46 17.257 45.708 46.121 1.00 0.00 C

ATOM 338 CG GLU 46 17.259 47.134 46.674 1.00 0.00 C

ATOM 339 CD GLU 46 17.269 47.051 48.193 1.00 0.00 C

ATOM 340 OE1 GLU 46 16.667 46.085 48.734 1.00 0.00 O

ATOM 341 OE2 GLU 46 17.875 47.951 48.832 1.00 0.00 O

ATOM 342 N ARG 47 18.587 43.308 44.521 1.00 0.00 N

ATOM 343 CA ARG 47 18.342 41.986 44.026 1.00 0.00 C

ATOM 344 C ARG 47 19.360 41.651 42.978 1.00 0.00 C

ATOM 345 O ARG 47 20.260 42.431 42.673 1.00 0.00 O

ATOM 346 CB ARG 47 16.948 41.823 43.397 1.00 0.00 C

ATOM 347 CG ARG 47 16.698 42.771 42.223 1.00 0.00 C

ATOM 348 CD ARG 47 15.303 42.635 41.610 1.00 0.00 C

ATOM 349 NE ARG 47 15.236 41.313 40.926 1.00 0.00 N

ATOM 350 CZ ARG 47 14.036 40.836 40.480 1.00 0.00 C

ATOM 351 NH1 ARG 47 12.896 41.557 40.688 1.00 0.00 N

ATOM 352 NH2 ARG 47 13.978 39.640 39.827 1.00 0.00 N

ATOM 353 N MET 48 19.224 40.434 42.423 1.00 0.00 N

ATOM 354 CA MET 48 20.072 39.842 41.431 1.00 0.00 C

ATOM 355 C MET 48 19.857 40.505 40.098 1.00 0.00 C

ATOM 356 O MET 48 18.857 41.189 39.889 1.00 0.00 O

ATOM 357 CB MET 48 19.684 38.393 41.228 1.00 0.00 C

ATOM 358 CG MET 48 18.170 38.391 41.092 1.00 0.00 C

ATOM 359 SD MET 48 17.440 36.845 40.554 1.00 0.00 S

ATOM 360 CE MET 48 15.788 37.302 41.158 1.00 0.00 C

ATOM 361 N GLY 49 20.797 40.280 39.144 1.00 0.00 N

ATOM 362 CA GLY 49 20.625 40.798 37.808 1.00 0.00 C

ATOM 363 C GLY 49 21.804 41.606 37.340 1.00 0.00 C

ATOM 364 O GLY 49 22.073 41.639 36.139 1.00 0.00 O

ATOM 365 N GLY 50 22.543 42.276 38.246 1.00 0.00 N

ATOM 366 CA GLY 50 23.694 43.033 37.822 1.00 0.00 C

ATOM 367 C GLY 50 23.259 44.144 36.920 1.00 0.00 C

ATOM 368 O GLY 50 22.362 44.923 37.230 1.00 0.00 O

ATOM 369 N THR 51 23.888 44.211 35.738 1.00 0.00 N

ATOM 370 CA THR 51 23.675 45.257 34.793 1.00 0.00 C

ATOM 371 C THR 51 22.219 45.281 34.463 1.00 0.00 C

ATOM 372 O THR 51 21.613 46.343 34.332 1.00 0.00 O

ATOM 373 CB THR 51 24.393 45.049 33.496 1.00 0.00 C

ATOM 374 CG2 THR 51 23.979 46.173 32.534 1.00 0.00 C

ATOM 375 OG1 THR 51 25.799 45.037 33.697 1.00 0.00 O

ATOM 376 N TRP 52 21.620 44.092 34.316 1.00 0.00 N

ATOM 377 CA TRP 52 20.240 43.984 33.960 1.00 0.00 C

ATOM 378 C TRP 52 19.394 44.502 35.078 1.00 0.00 C

ATOM 379 O TRP 52 18.265 44.926 34.834 1.00 0.00 O

ATOM 380 CB TRP 52 19.850 42.540 33.626 1.00 0.00 C

ATOM 381 CG TRP 52 20.639 42.051 32.436 1.00 0.00 C

ATOM 382 CD1 TRP 52 21.627 41.112 32.374 1.00 0.00 C

ATOM 383 CD2 TRP 52 20.511 42.594 31.112 1.00 0.00 C

ATOM 384 CE2 TRP 52 21.440 41.939 30.305 1.00 0.00 C

ATOM 385 CE3 TRP 52 19.692 43.566 30.613 1.00 0.00 C

ATOM 386 NE1 TRP 52 22.116 41.030 31.092 1.00 0.00 N

ATOM 387 CZ2 TRP 52 21.564 42.246 28.979 1.00 0.00 C

ATOM 388 CZ3 TRP 52 19.813 43.867 29.275 1.00 0.00 C

ATOM 389 CH2 TRP 52 20.731 43.220 28.474 1.00 0.00 C

ATOM 390 N ASP 53 19.850 44.387 36.347 1.00 0.00 N

ATOM 391 CA ASP 53 18.999 44.920 37.374 1.00 0.00 C

ATOM 392 C ASP 53 18.993 46.428 37.383 1.00 0.00 C

ATOM 393 O ASP 53 17.931 47.048 37.398 1.00 0.00 O

ATOM 394 CB ASP 53 19.262 44.409 38.812 1.00 0.00 C

ATOM 395 CG ASP 53 20.503 45.024 39.436 1.00 0.00 C

ATOM 396 OD1 ASP 53 20.406 46.208 39.857 1.00 0.00 O

ATOM 397 OD2 ASP 53 21.539 44.317 39.548 1.00 0.00 O

ATOM 398 N LEU 54 20.181 47.066 37.377 1.00 0.00 N

ATOM 399 CA LEU 54 20.266 48.504 37.474 1.00 0.00 C

ATOM 400 C LEU 54 19.832 49.204 36.210 1.00 0.00 C

ATOM 401 O LEU 54 19.178 50.243 36.278 1.00 0.00 O

ATOM 402 CB LEU 54 21.695 49.031 37.720 1.00 0.00 C

ATOM 403 CG LEU 54 22.394 48.537 39.000 1.00 0.00 C

ATOM 404 CD1 LEU 54 22.816 47.066 38.882 1.00 0.00 C

ATOM 405 CD2 LEU 54 23.569 49.454 39.379 1.00 0.00 C

ATOM 406 N PHE 55 20.206 48.680 35.019 1.00 0.00 N

ATOM 407 CA PHE 55 19.962 49.416 33.800 1.00 0.00 C

ATOM 408 C PHE 55 18.584 49.164 33.277 1.00 0.00 C

ATOM 409 O PHE 55 18.325 48.137 32.655 1.00 0.00 O

ATOM 410 CB PHE 55 20.921 49.036 32.659 1.00 0.00 C

ATOM 411 CG PHE 55 22.296 49.447 33.050 1.00 0.00 C

ATOM 412 CD1 PHE 55 23.037 48.666 33.907 1.00 0.00 C

ATOM 413 CD2 PHE 55 22.848 50.604 32.551 1.00 0.00 C

ATOM 414 CE1 PHE 55 24.309 49.039 34.269 1.00 0.00 C

ATOM 415 CE2 PHE 55 24.121 50.981 32.910 1.00 0.00 C

ATOM 416 CZ PHE 55 24.852 50.198 33.771 1.00 0.00 C

ATOM 417 N ARG 56 17.657 50.121 33.478 1.00 0.00 N

ATOM 418 CA ARG 56 16.340 49.882 32.967 1.00 0.00 C

ATOM 419 C ARG 56 15.872 51.090 32.220 1.00 0.00 C

ATOM 420 O ARG 56 14.750 51.549 32.423 1.00 0.00 O

ATOM 421 CB ARG 56 15.306 49.644 34.078 1.00 0.00 C

ATOM 422 CG ARG 56 15.640 48.433 34.944 1.00 0.00 C

ATOM 423 CD ARG 56 14.630 48.170 36.059 1.00 0.00 C

ATOM 424 NE ARG 56 15.112 46.973 36.803 1.00 0.00 N

ATOM 425 CZ ARG 56 14.629 46.706 38.051 1.00 0.00 C

ATOM 426 NH1 ARG 56 13.656 47.498 38.590 1.00 0.00 N

ATOM 427 NH2 ARG 56 15.124 45.652 38.762 1.00 0.00 N

ATOM 428 N TYR 57 16.702 51.609 31.300 1.00 0.00 N

ATOM 429 CA TYR 57 16.322 52.736 30.501 1.00 0.00 C

ATOM 430 C TYR 57 15.587 52.171 29.328 1.00 0.00 C

ATOM 431 O TYR 57 15.864 51.049 28.913 1.00 0.00 O

ATOM 432 CB TYR 57 17.520 53.559 29.993 1.00 0.00 C

ATOM 433 CG TYR 57 18.431 52.636 29.259 1.00 0.00 C

ATOM 434 CD1 TYR 57 18.187 52.297 27.949 1.00 0.00 C

ATOM 435 CD2 TYR 57 19.536 52.109 29.887 1.00 0.00 C

ATOM 436 CE1 TYR 57 19.032 51.446 27.279 1.00 0.00 C

ATOM 437 CE2 TYR 57 20.385 51.257 29.221 1.00 0.00 C

ATOM 438 CZ TYR 57 20.133 50.924 27.913 1.00 0.00 C

ATOM 439 OH TYR 57 20.999 50.051 27.222 1.00 0.00 O

ATOM 440 N PRO 58 14.614 52.877 28.818 1.00 0.00 N

ATOM 441 CA PRO 58 13.824 52.391 27.722 1.00 0.00 C

ATOM 442 C PRO 58 14.685 52.003 26.558 1.00 0.00 C

ATOM 443 O PRO 58 15.580 52.764 26.189 1.00 0.00 O

ATOM 444 CB PRO 58 12.884 53.537 27.361 1.00 0.00 C

ATOM 445 CG PRO 58 13.703 54.784 27.740 1.00 0.00 C

ATOM 446 CD PRO 58 14.559 54.324 28.934 1.00 0.00 C

ATOM 447 N GLY 59 14.412 50.822 25.964 1.00 0.00 N

ATOM 448 CA GLY 59 15.100 50.375 24.786 1.00 0.00 C

ATOM 449 C GLY 59 16.263 49.496 25.143 1.00 0.00 C

ATOM 450 O GLY 59 16.845 48.874 24.257 1.00 0.00 O

ATOM 451 N ILE 60 16.619 49.379 26.440 1.00 0.00 N

ATOM 452 CA ILE 60 17.779 48.601 26.794 1.00 0.00 C

ATOM 453 C ILE 60 17.619 47.205 26.285 1.00 0.00 C

ATOM 454 O ILE 60 16.610 46.547 26.543 1.00 0.00 O

ATOM 455 CB ILE 60 18.025 48.547 28.273 1.00 0.00 C

ATOM 456 CG1 ILE 60 19.312 47.762 28.580 1.00 0.00 C

ATOM 457 CG2 ILE 60 16.757 48.002 28.947 1.00 0.00 C

ATOM 458 CD1 ILE 60 19.795 47.921 30.020 1.00 0.00 C

ATOM 459 N ARG 61 18.633 46.728 25.527 1.00 0.00 N

ATOM 460 CA ARG 61 18.546 45.431 24.922 1.00 0.00 C

ATOM 461 C ARG 61 19.923 44.910 24.642 1.00 0.00 C

ATOM 462 O ARG 61 20.880 45.667 24.495 1.00 0.00 O

ATOM 463 CB ARG 61 17.779 45.470 23.590 1.00 0.00 C

ATOM 464 CG ARG 61 17.362 44.100 23.062 1.00 0.00 C

ATOM 465 CD ARG 61 16.604 44.178 21.737 1.00 0.00 C

ATOM 466 NE ARG 61 15.422 45.065 21.941 1.00 0.00 N

ATOM 467 CZ ARG 61 14.267 44.573 22.479 1.00 0.00 C

ATOM 468 NH1 ARG 61 14.212 43.283 22.918 1.00 0.00 N

ATOM 469 NH2 ARG 61 13.168 45.377 22.580 1.00 0.00 N

ATOM 470 N SER 62 20.053 43.572 24.557 1.00 0.00 N

ATOM 471 CA SER 62 21.344 42.994 24.333 1.00 0.00 C

ATOM 472 C SER 62 21.700 43.071 22.874 1.00 0.00 C

ATOM 473 O SER 62 20.853 43.162 21.987 1.00 0.00 O

ATOM 474 CB SER 62 21.422 41.515 24.756 1.00 0.00 C

ATOM 475 OG SER 62 20.523 40.736 23.980 1.00 0.00 O

ATOM 476 N ASP 63 23.021 43.104 22.651 1.00 0.00 N

ATOM 477 CA ASP 63 23.823 43.063 21.461 1.00 0.00 C

ATOM 478 C ASP 63 23.771 41.678 20.806 1.00 0.00 C

ATOM 479 O ASP 63 23.779 41.572 19.584 1.00 0.00 O

ATOM 480 CB ASP 63 25.328 43.325 21.822 1.00 0.00 C

ATOM 481 CG ASP 63 26.046 43.122 20.496 1.00 0.00 C

ATOM 482 OD1 ASP 63 25.436 43.474 19.455 1.00 0.00 O

ATOM 483 OD2 ASP 63 27.181 42.582 20.485 1.00 0.00 O

ATOM 484 N SER 64 23.719 40.562 21.574 1.00 0.00 N

ATOM 485 CA SER 64 23.851 39.238 21.005 1.00 0.00 C

ATOM 486 C SER 64 22.555 38.510 21.045 1.00 0.00 C

ATOM 487 O SER 64 21.686 38.793 21.869 1.00 0.00 O

ATOM 488 CB SER 64 24.925 38.390 21.709 1.00 0.00 C

ATOM 489 OG SER 64 24.643 38.297 23.096 1.00 0.00 O

ATOM 490 N ASP 65 22.422 37.525 20.130 1.00 0.00 N

ATOM 491 CA ASP 65 21.216 36.768 20.000 1.00 0.00 C

ATOM 492 C ASP 65 20.941 36.197 21.339 1.00 0.00 C

ATOM 493 O ASP 65 21.837 35.750 22.052 1.00 0.00 O

ATOM 494 CB ASP 65 21.309 35.615 18.985 1.00 0.00 C

ATOM 495 CG ASP 65 21.367 36.215 17.587 1.00 0.00 C

ATOM 496 OD1 ASP 65 21.206 37.459 17.471 1.00 0.00 O

ATOM 497 OD2 ASP 65 21.574 35.439 16.616 1.00 0.00 O

ATOM 498 N LEU 66 19.657 36.216 21.706 1.00 0.00 N

ATOM 499 CA LEU 66 19.236 35.814 23.007 1.00 0.00 C

ATOM 500 C LEU 66 19.540 34.358 23.195 1.00 0.00 C

ATOM 501 O LEU 66 19.909 33.949 24.295 1.00 0.00 O

ATOM 502 CB LEU 66 17.732 36.085 23.220 1.00 0.00 C

ATOM 503 CG LEU 66 17.211 35.926 24.664 1.00 0.00 C

ATOM 504 CD1 LEU 66 15.751 36.391 24.759 1.00 0.00 C

ATOM 505 CD2 LEU 66 17.386 34.498 25.204 1.00 0.00 C

ATOM 506 N TYR 67 19.437 33.528 22.131 1.00 0.00 N

ATOM 507 CA TYR 67 19.632 32.125 22.369 1.00 0.00 C

ATOM 508 C TYR 67 21.091 31.822 22.306 1.00 0.00 C

ATOM 509 O TYR 67 21.500 30.672 22.158 1.00 0.00 O

ATOM 510 CB TYR 67 18.941 31.241 21.314 1.00 0.00 C

ATOM 511 CG TYR 67 19.538 31.557 19.983 1.00 0.00 C

ATOM 512 CD1 TYR 67 20.668 30.906 19.541 1.00 0.00 C

ATOM 513 CD2 TYR 67 18.963 32.509 19.174 1.00 0.00 C

ATOM 514 CE1 TYR 67 21.216 31.201 18.316 1.00 0.00 C

ATOM 515 CE2 TYR 67 19.506 32.809 17.947 1.00 0.00 C

ATOM 516 CZ TYR 67 20.634 32.155 17.516 1.00 0.00 C

ATOM 517 OH TYR 67 21.194 32.461 16.258 1.00 0.00 O

ATOM 518 N THR 68 21.923 32.855 22.484 1.00 0.00 N

ATOM 519 CA THR 68 23.331 32.653 22.544 1.00 0.00 C

ATOM 520 C THR 68 23.718 33.317 23.822 1.00 0.00 C

ATOM 521 O THR 68 24.568 32.824 24.561 1.00 0.00 O

ATOM 522 CB THR 68 24.071 33.336 21.431 1.00 0.00 C

ATOM 523 CG2 THR 68 25.575 33.063 21.598 1.00 0.00 C

ATOM 524 OG1 THR 68 23.622 32.847 20.176 1.00 0.00 O

ATOM 525 N PHE 69 23.061 34.459 24.117 1.00 0.00 N

ATOM 526 CA PHE 69 23.313 35.189 25.326 1.00 0.00 C

ATOM 527 C PHE 69 22.413 34.612 26.376 1.00 0.00 C

ATOM 528 O PHE 69 21.363 35.168 26.698 1.00 0.00 O

ATOM 529 CB PHE 69 22.972 36.688 25.201 1.00 0.00 C

ATOM 530 CG PHE 69 23.263 37.369 26.498 1.00 0.00 C

ATOM 531 CD1 PHE 69 24.553 37.721 26.823 1.00 0.00 C

ATOM 532 CD2 PHE 69 22.250 37.673 27.381 1.00 0.00 C

ATOM 533 CE1 PHE 69 24.829 38.353 28.012 1.00 0.00 C

ATOM 534 CE2 PHE 69 22.521 38.305 28.572 1.00 0.00 C

ATOM 535 CZ PHE 69 23.813 38.645 28.890 1.00 0.00 C

ATOM 536 N GLY 70 22.832 33.481 26.969 1.00 0.00 N

ATOM 537 CA GLY 70 22.041 32.857 27.986 1.00 0.00 C

ATOM 538 C GLY 70 22.860 31.734 28.551 1.00 0.00 C

ATOM 539 O GLY 70 23.871 31.349 27.969 1.00 0.00 O

ATOM 540 N PHE 71 22.433 31.165 29.699 1.00 0.00 N

ATOM 541 CA PHE 71 23.189 30.126 30.354 1.00 0.00 C

ATOM 542 C PHE 71 23.023 28.834 29.618 1.00 0.00 C

ATOM 543 O PHE 71 21.920 28.451 29.234 1.00 0.00 O

ATOM 544 CB PHE 71 22.743 29.874 31.804 1.00 0.00 C

ATOM 545 CG PHE 71 23.095 31.068 32.623 1.00 0.00 C

ATOM 546 CD1 PHE 71 22.528 32.288 32.346 1.00 0.00 C

ATOM 547 CD2 PHE 71 23.951 30.967 33.695 1.00 0.00 C

ATOM 548 CE1 PHE 71 22.835 33.393 33.102 1.00 0.00 C

ATOM 549 CE2 PHE 71 24.261 32.069 34.457 1.00 0.00 C

ATOM 550 CZ PHE 71 23.705 33.288 34.160 1.00 0.00 C

ATOM 551 N ASP 72 24.162 28.153 29.383 1.00 0.00 N

ATOM 552 CA ASP 72 24.255 26.891 28.703 1.00 0.00 C

ATOM 553 C ASP 72 23.783 25.750 29.554 1.00 0.00 C

ATOM 554 O ASP 72 23.151 24.821 29.052 1.00 0.00 O

ATOM 555 CB ASP 72 25.699 26.573 28.284 1.00 0.00 C

ATOM 556 CG ASP 72 26.138 27.591 27.239 1.00 0.00 C

ATOM 557 OD1 ASP 72 25.282 28.408 26.804 1.00 0.00 O

ATOM 558 OD2 ASP 72 27.339 27.562 26.860 1.00 0.00 O

ATOM 559 N PHE 73 24.087 25.767 30.867 1.00 0.00 N

ATOM 560 CA PHE 73 23.741 24.614 31.648 1.00 0.00 C

ATOM 561 C PHE 73 22.325 24.734 32.154 1.00 0.00 C

ATOM 562 O PHE 73 22.101 24.831 33.359 1.00 0.00 O

ATOM 563 CB PHE 73 24.728 24.400 32.817 1.00 0.00 C

ATOM 564 CG PHE 73 24.818 25.658 33.616 1.00 0.00 C

ATOM 565 CD1 PHE 73 25.524 26.733 33.124 1.00 0.00 C

ATOM 566 CD2 PHE 73 24.196 25.782 34.837 1.00 0.00 C

ATOM 567 CE1 PHE 73 25.619 27.903 33.839 1.00 0.00 C

ATOM 568 CE2 PHE 73 24.287 26.949 35.559 1.00 0.00 C

ATOM 569 CZ PHE 73 25.000 28.013 35.061 1.00 0.00 C

ATOM 570 N LYS 74 21.315 24.665 31.247 1.00 0.00 N

ATOM 571 CA LYS 74 19.944 24.800 31.688 1.00 0.00 C

ATOM 572 C LYS 74 19.022 24.995 30.497 1.00 0.00 C

ATOM 573 O LYS 74 19.466 25.337 29.403 1.00 0.00 O

ATOM 574 CB LYS 74 19.718 26.089 32.478 1.00 0.00 C

ATOM 575 CG LYS 74 19.939 27.277 31.539 1.00 0.00 C

ATOM 576 CD LYS 74 19.367 28.614 31.998 1.00 0.00 C

ATOM 577 CE LYS 74 19.514 29.693 30.923 1.00 0.00 C

ATOM 578 NZ LYS 74 18.648 29.377 29.766 1.00 0.00 N

ATOM 579 N PRO 75 17.736 24.746 30.698 1.00 0.00 N

ATOM 580 CA PRO 75 16.706 25.032 29.709 1.00 0.00 C

ATOM 581 C PRO 75 16.133 26.418 29.914 1.00 0.00 C

ATOM 582 O PRO 75 16.338 26.973 30.990 1.00 0.00 O

ATOM 583 CB PRO 75 15.641 23.950 29.881 1.00 0.00 C

ATOM 584 CG PRO 75 15.849 23.444 31.317 1.00 0.00 C

ATOM 585 CD PRO 75 17.356 23.633 31.551 1.00 0.00 C

ATOM 586 N TRP 76 15.420 26.999 28.913 1.00 0.00 N

ATOM 587 CA TRP 76 14.781 28.293 29.046 1.00 0.00 C

ATOM 588 C TRP 76 14.106 28.622 27.740 1.00 0.00 C

ATOM 589 O TRP 76 14.372 27.980 26.726 1.00 0.00 O

ATOM 590 CB TRP 76 15.764 29.428 29.382 1.00 0.00 C

ATOM 591 CG TRP 76 15.159 30.812 29.398 1.00 0.00 C

ATOM 592 CD1 TRP 76 15.220 31.779 28.438 1.00 0.00 C

ATOM 593 CD2 TRP 76 14.377 31.358 30.473 1.00 0.00 C

ATOM 594 CE2 TRP 76 14.008 32.651 30.100 1.00 0.00 C

ATOM 595 CE3 TRP 76 13.994 30.827 31.672 1.00 0.00 C

ATOM 596 NE1 TRP 76 14.536 32.897 28.850 1.00 0.00 N

ATOM 597 CZ2 TRP 76 13.251 33.436 30.922 1.00 0.00 C

ATOM 598 CZ3 TRP 76 13.231 31.622 32.498 1.00 0.00 C

ATOM 599 CH2 TRP 76 12.866 32.901 32.132 1.00 0.00 C

ATOM 600 N THR 77 13.191 29.622 27.728 1.00 0.00 N

ATOM 601 CA THR 77 12.532 29.997 26.500 1.00 0.00 C

ATOM 602 C THR 77 12.609 31.492 26.351 1.00 0.00 C

ATOM 603 O THR 77 12.496 32.228 27.327 1.00 0.00 O

ATOM 604 CB THR 77 11.079 29.626 26.464 1.00 0.00 C

ATOM 605 CG2 THR 77 10.954 28.110 26.692 1.00 0.00 C

ATOM 606 OG1 THR 77 10.370 30.331 27.472 1.00 0.00 O

ATOM 607 N LYS 78 12.807 31.984 25.109 1.00 0.00 N

ATOM 608 CA LYS 78 12.914 33.400 24.869 1.00 0.00 C

ATOM 609 C LYS 78 12.095 33.706 23.657 1.00 0.00 C

ATOM 610 O LYS 78 11.997 32.878 22.753 1.00 0.00 O

ATOM 611 CB LYS 78 14.338 33.826 24.506 1.00 0.00 C

ATOM 612 CG LYS 78 14.763 33.292 23.137 1.00 0.00 C

ATOM 613 CD LYS 78 14.734 31.765 23.048 1.00 0.00 C

ATOM 614 CE LYS 78 14.818 31.232 21.616 1.00 0.00 C

ATOM 615 NZ LYS 78 16.083 31.665 20.983 1.00 0.00 N

ATOM 616 N ALA 79 11.452 34.891 23.592 1.00 0.00 N

ATOM 617 CA ALA 79 10.749 35.121 22.363 1.00 0.00 C

ATOM 618 C ALA 79 11.170 36.425 21.763 1.00 0.00 C

ATOM 619 O ALA 79 10.351 37.331 21.620 1.00 0.00 O

ATOM 620 CB ALA 79 9.224 35.194 22.548 1.00 0.00 C

ATOM 621 N LYS 80 12.442 36.534 21.330 1.00 0.00 N

ATOM 622 CA LYS 80 12.902 37.735 20.693 1.00 0.00 C

ATOM 623 C LYS 80 14.264 37.413 20.174 1.00 0.00 C

ATOM 624 O LYS 80 15.000 36.655 20.802 1.00 0.00 O

ATOM 625 CB LYS 80 13.129 38.938 21.633 1.00 0.00 C

ATOM 626 CG LYS 80 11.919 39.432 22.432 1.00 0.00 C

ATOM 627 CD LYS 80 11.620 38.609 23.687 1.00 0.00 C

ATOM 628 CE LYS 80 10.459 39.158 24.519 1.00 0.00 C

ATOM 629 NZ LYS 80 10.833 40.461 25.112 1.00 0.00 N

ATOM 630 N SER 81 14.647 37.968 19.008 1.00 0.00 N

ATOM 631 CA SER 81 15.972 37.693 18.538 1.00 0.00 C

ATOM 632 C SER 81 16.934 38.275 19.529 1.00 0.00 C

ATOM 633 O SER 81 17.848 37.591 19.988 1.00 0.00 O

ATOM 634 CB SER 81 16.247 38.296 17.152 1.00 0.00 C

ATOM 635 OG SER 81 16.024 39.695 17.184 1.00 0.00 O

ATOM 636 N LEU 82 16.751 39.564 19.885 1.00 0.00 N

ATOM 637 CA LEU 82 17.565 40.180 20.902 1.00 0.00 C

ATOM 638 C LEU 82 16.654 40.576 22.018 1.00 0.00 C

ATOM 639 O LEU 82 15.727 41.363 21.837 1.00 0.00 O

ATOM 640 CB LEU 82 18.369 41.411 20.431 1.00 0.00 C

ATOM 641 CG LEU 82 17.786 42.160 19.216 1.00 0.00 C

ATOM 642 CD1 LEU 82 18.091 41.405 17.914 1.00 0.00 C

ATOM 643 CD2 LEU 82 16.285 42.444 19.378 1.00 0.00 C

ATOM 644 N ALA 83 16.914 40.024 23.218 1.00 0.00 N

ATOM 645 CA ALA 83 16.057 40.207 24.358 1.00 0.00 C

ATOM 646 C ALA 83 16.224 41.562 24.968 1.00 0.00 C

ATOM 647 O ALA 83 17.315 42.128 24.990 1.00 0.00 O

ATOM 648 CB ALA 83 16.310 39.190 25.481 1.00 0.00 C

ATOM 649 N ASP 84 15.105 42.103 25.496 1.00 0.00 N

ATOM 650 CA ASP 84 15.082 43.364 26.181 1.00 0.00 C

ATOM 651 C ASP 84 15.491 43.097 27.600 1.00 0.00 C

ATOM 652 O ASP 84 15.556 41.949 28.032 1.00 0.00 O

ATOM 653 CB ASP 84 13.688 44.020 26.205 1.00 0.00 C

ATOM 654 CG ASP 84 13.808 45.436 26.758 1.00 0.00 C

ATOM 655 OD1 ASP 84 14.076 46.360 25.944 1.00 0.00 O

ATOM 656 OD2 ASP 84 13.613 45.619 27.989 1.00 0.00 O

ATOM 657 N ALA 85 15.760 44.171 28.368 1.00 0.00 N

ATOM 658 CA ALA 85 16.240 44.066 29.718 1.00 0.00 C

ATOM 659 C ALA 85 15.259 43.370 30.600 1.00 0.00 C

ATOM 660 O ALA 85 15.649 42.581 31.458 1.00 0.00 O

ATOM 661 CB ALA 85 16.526 45.431 30.364 1.00 0.00 C

ATOM 662 N ALA 86 13.957 43.648 30.427 1.00 0.00 N

ATOM 663 CA ALA 86 12.989 43.047 31.295 1.00 0.00 C

ATOM 664 C ALA 86 13.071 41.562 31.137 1.00 0.00 C

ATOM 665 O ALA 86 13.001 40.822 32.116 1.00 0.00 O

ATOM 666 CB ALA 86 11.547 43.468 30.963 1.00 0.00 C

ATOM 667 N ASP 87 13.221 41.087 29.887 1.00 0.00 N

ATOM 668 CA ASP 87 13.263 39.676 29.633 1.00 0.00 C

ATOM 669 C ASP 87 14.483 39.084 30.269 1.00 0.00 C

ATOM 670 O ASP 87 14.407 38.027 30.894 1.00 0.00 O

ATOM 671 CB ASP 87 13.305 39.339 28.132 1.00 0.00 C

ATOM 672 CG ASP 87 13.099 37.837 27.977 1.00 0.00 C

ATOM 673 OD1 ASP 87 12.938 37.147 29.018 1.00 0.00 O

ATOM 674 OD2 ASP 87 13.096 37.361 26.811 1.00 0.00 O

ATOM 675 N ILE 88 15.651 39.746 30.133 1.00 0.00 N

ATOM 676 CA ILE 88 16.829 39.178 30.721 1.00 0.00 C

ATOM 677 C ILE 88 16.700 39.155 32.212 1.00 0.00 C

ATOM 678 O ILE 88 17.103 38.189 32.856 1.00 0.00 O

ATOM 679 CB ILE 88 18.137 39.861 30.419 1.00 0.00 C

ATOM 680 CG1 ILE 88 18.588 39.652 28.961 1.00 0.00 C

ATOM 681 CG2 ILE 88 19.161 39.313 31.428 1.00 0.00 C

ATOM 682 CD1 ILE 88 17.905 40.546 27.937 1.00 0.00 C

ATOM 683 N LEU 89 16.142 40.225 32.807 1.00 0.00 N

ATOM 684 CA LEU 89 16.043 40.301 34.238 1.00 0.00 C

ATOM 685 C LEU 89 15.205 39.152 34.706 1.00 0.00 C

ATOM 686 O LEU 89 15.533 38.499 35.696 1.00 0.00 O

ATOM 687 CB LEU 89 15.359 41.602 34.705 1.00 0.00 C

ATOM 688 CG LEU 89 15.402 41.875 36.226 1.00 0.00 C

ATOM 689 CD1 LEU 89 14.627 40.834 37.046 1.00 0.00 C

ATOM 690 CD2 LEU 89 16.847 42.066 36.710 1.00 0.00 C

ATOM 691 N GLU 90 14.104 38.866 33.987 1.00 0.00 N

ATOM 692 CA GLU 90 13.214 37.812 34.381 1.00 0.00 C

ATOM 693 C GLU 90 13.982 36.533 34.356 1.00 0.00 C

ATOM 694 O GLU 90 13.834 35.690 35.240 1.00 0.00 O

ATOM 695 CB GLU 90 12.017 37.647 33.429 1.00 0.00 C

ATOM 696 CG GLU 90 11.060 36.528 33.842 1.00 0.00 C

ATOM 697 CD GLU 90 10.287 36.998 35.067 1.00 0.00 C

ATOM 698 OE1 GLU 90 10.494 38.169 35.484 1.00 0.00 O

ATOM 699 OE2 GLU 90 9.480 36.192 35.602 1.00 0.00 O

ATOM 700 N TYR 91 14.844 36.363 33.338 1.00 0.00 N

ATOM 701 CA TYR 91 15.589 35.146 33.245 1.00 0.00 C

ATOM 702 C TYR 91 16.451 34.992 34.459 1.00 0.00 C

ATOM 703 O TYR 91 16.485 33.923 35.067 1.00 0.00 O

ATOM 704 CB TYR 91 16.526 35.076 32.027 1.00 0.00 C

ATOM 705 CG TYR 91 17.493 33.986 32.341 1.00 0.00 C

ATOM 706 CD1 TYR 91 17.139 32.662 32.232 1.00 0.00 C

ATOM 707 CD2 TYR 91 18.768 34.302 32.753 1.00 0.00 C

ATOM 708 CE1 TYR 91 18.044 31.673 32.539 1.00 0.00 C

ATOM 709 CE2 TYR 91 19.678 33.319 33.061 1.00 0.00 C

ATOM 710 CZ TYR 91 19.313 32.000 32.955 1.00 0.00 C

ATOM 711 OH TYR 91 20.243 30.988 33.273 1.00 0.00 O

ATOM 712 N LEU 92 17.159 36.062 34.864 1.00 0.00 N

ATOM 713 CA LEU 92 18.051 35.943 35.982 1.00 0.00 C

ATOM 714 C LEU 92 17.278 35.639 37.224 1.00 0.00 C

ATOM 715 O LEU 92 17.737 34.870 38.069 1.00 0.00 O

ATOM 716 CB LEU 92 18.897 37.203 36.244 1.00 0.00 C

ATOM 717 CG LEU 92 19.963 37.470 35.164 1.00 0.00 C

ATOM 718 CD1 LEU 92 20.806 38.707 35.515 1.00 0.00 C

ATOM 719 CD2 LEU 92 20.823 36.221 34.899 1.00 0.00 C

ATOM 720 N SER 93 16.086 36.243 37.373 1.00 0.00 N

ATOM 721 CA SER 93 15.297 36.028 38.550 1.00 0.00 C

ATOM 722 C SER 93 14.948 34.584 38.659 1.00 0.00 C

ATOM 723 O SER 93 15.059 33.985 39.728 1.00 0.00 O

ATOM 724 CB SER 93 13.975 36.814 38.545 1.00 0.00 C

ATOM 725 OG SER 93 13.250 36.550 39.738 1.00 0.00 O

ATOM 726 N GLU 94 14.532 33.981 37.534 1.00 0.00 N

ATOM 727 CA GLU 94 14.120 32.611 37.545 1.00 0.00 C

ATOM 728 C GLU 94 15.291 31.771 37.931 1.00 0.00 C

ATOM 729 O GLU 94 15.153 30.799 38.672 1.00 0.00 O

ATOM 730 CB GLU 94 13.642 32.110 36.171 1.00 0.00 C

ATOM 731 CG GLU 94 12.339 32.756 35.696 1.00 0.00 C

ATOM 732 CD GLU 94 11.205 32.225 36.563 1.00 0.00 C

ATOM 733 OE1 GLU 94 11.490 31.394 37.466 1.00 0.00 O

ATOM 734 OE2 GLU 94 10.039 32.644 36.333 1.00 0.00 O

ATOM 735 N ALA 95 16.487 32.136 37.437 1.00 0.00 N

ATOM 736 CA ALA 95 17.654 31.341 37.683 1.00 0.00 C

ATOM 737 C ALA 95 17.938 31.287 39.149 1.00 0.00 C

ATOM 738 O ALA 95 18.207 30.214 39.688 1.00 0.00 O

ATOM 739 CB ALA 95 18.911 31.905 36.992 1.00 0.00 C

ATOM 740 N ILE 96 17.868 32.434 39.849 1.00 0.00 N

ATOM 741 CA ILE 96 18.211 32.375 41.240 1.00 0.00 C

ATOM 742 C ILE 96 17.202 31.543 41.953 1.00 0.00 C

ATOM 743 O ILE 96 17.545 30.798 42.868 1.00 0.00 O

ATOM 744 CB ILE 96 18.255 33.672 41.985 1.00 0.00 C

ATOM 745 CG1 ILE 96 16.852 34.283 42.111 1.00 0.00 C

ATOM 746 CG2 ILE 96 19.323 34.556 41.323 1.00 0.00 C

ATOM 747 CD1 ILE 96 16.769 35.381 43.171 1.00 0.00 C

ATOM 748 N ASP 97 15.919 31.674 41.569 1.00 0.00 N

ATOM 749 CA ASP 97 14.880 30.963 42.252 1.00 0.00 C

ATOM 750 C ASP 97 15.114 29.494 42.108 1.00 0.00 C

ATOM 751 O ASP 97 14.990 28.741 43.073 1.00 0.00 O

ATOM 752 CB ASP 97 13.479 31.259 41.687 1.00 0.00 C

ATOM 753 CG ASP 97 13.095 32.682 42.069 1.00 0.00 C

ATOM 754 OD1 ASP 97 13.778 33.267 42.951 1.00 0.00 O

ATOM 755 OD2 ASP 97 12.106 33.201 41.485 1.00 0.00 O

ATOM 756 N GLU 98 15.491 29.049 40.898 1.00 0.00 N

ATOM 757 CA GLU 98 15.640 27.646 40.649 1.00 0.00 C

ATOM 758 C GLU 98 16.728 27.077 41.509 1.00 0.00 C

ATOM 759 O GLU 98 16.570 25.990 42.062 1.00 0.00 O

ATOM 760 CB GLU 98 15.991 27.335 39.182 1.00 0.00 C

ATOM 761 CG GLU 98 15.840 25.859 38.803 1.00 0.00 C

ATOM 762 CD GLU 98 17.024 25.077 39.356 1.00 0.00 C

ATOM 763 OE1 GLU 98 18.165 25.321 38.882 1.00 0.00 O

ATOM 764 OE2 GLU 98 16.803 24.221 40.253 1.00 0.00 O

ATOM 765 N HIS 99 17.857 27.795 41.671 1.00 0.00 N

ATOM 766 CA HIS 99 18.949 27.194 42.387 1.00 0.00 C

ATOM 767 C HIS 99 19.015 27.676 43.810 1.00 0.00 C

ATOM 768 O HIS 99 19.985 27.379 44.506 1.00 0.00 O

ATOM 769 CB HIS 99 20.317 27.513 41.759 1.00 0.00 C

ATOM 770 CG HIS 99 20.484 26.976 40.368 1.00 0.00 C

ATOM 771 CD2 HIS 99 21.005 25.793 39.942 1.00 0.00 C

ATOM 772 ND1 HIS 99 20.116 27.656 39.228 1.00 0.00 N

ATOM 773 CE1 HIS 99 20.430 26.858 38.177 1.00 0.00 C

ATOM 774 NE2 HIS 99 20.972 25.716 38.561 1.00 0.00 N

ATOM 775 N GLN 100 17.992 28.410 44.292 1.00 0.00 N

ATOM 776 CA GLN 100 17.944 28.853 45.665 1.00 0.00 C

ATOM 777 C GLN 100 19.228 29.537 46.045 1.00 0.00 C

ATOM 778 O GLN 100 19.791 29.287 47.110 1.00 0.00 O

ATOM 779 CB GLN 100 17.677 27.700 46.651 1.00 0.00 C

ATOM 780 CG GLN 100 17.503 28.142 48.106 1.00 0.00 C

ATOM 781 CD GLN 100 17.250 26.893 48.938 1.00 0.00 C

ATOM 782 NE2 GLN 100 17.294 25.706 48.277 1.00 0.00 N

ATOM 783 OE1 GLN 100 17.015 26.971 50.143 1.00 0.00 O

ATOM 784 N LEU 101 19.725 30.414 45.157 1.00 0.00 N

ATOM 785 CA LEU 101 20.944 31.161 45.314 1.00 0.00 C

ATOM 786 C LEU 101 20.834 32.295 46.301 1.00 0.00 C

ATOM 787 O LEU 101 21.831 32.692 46.899 1.00 0.00 O

ATOM 788 CB LEU 101 21.457 31.695 43.973 1.00 0.00 C

ATOM 789 CG LEU 101 21.803 30.532 43.027 1.00 0.00 C

ATOM 790 CD1 LEU 101 22.341 31.028 41.682 1.00 0.00 C

ATOM 791 CD2 LEU 101 22.741 29.526 43.711 1.00 0.00 C

ATOM 792 N ALA 102 19.629 32.862 46.496 1.00 0.00 N

ATOM 793 CA ALA 102 19.457 34.053 47.293 1.00 0.00 C

ATOM 794 C ALA 102 19.945 33.882 48.709 1.00 0.00 C

ATOM 795 O ALA 102 20.503 34.829 49.263 1.00 0.00 O

ATOM 796 CB ALA 102 17.987 34.504 47.373 1.00 0.00 C

ATOM 797 N PRO 103 19.768 32.755 49.346 1.00 0.00 N

ATOM 798 CA PRO 103 20.203 32.627 50.718 1.00 0.00 C

ATOM 799 C PRO 103 21.688 32.761 50.876 1.00 0.00 C

ATOM 800 O PRO 103 22.164 33.003 51.983 1.00 0.00 O

ATOM 801 CB PRO 103 19.630 31.296 51.199 1.00 0.00 C

ATOM 802 CG PRO 103 18.347 31.134 50.360 1.00 0.00 C

ATOM 803 CD PRO 103 18.641 31.885 49.050 1.00 0.00 C

ATOM 804 N PHE 104 22.418 32.548 49.777 1.00 0.00 N

ATOM 805 CA PHE 104 23.836 32.612 49.572 1.00 0.00 C

ATOM 806 C PHE 104 24.292 34.046 49.565 1.00 0.00 C

ATOM 807 O PHE 104 25.467 34.332 49.800 1.00 0.00 O

ATOM 808 CB PHE 104 24.130 31.982 48.198 1.00 0.00 C

ATOM 809 CG PHE 104 25.553 32.086 47.809 1.00 0.00 C

ATOM 810 CD1 PHE 104 26.042 33.273 47.323 1.00 0.00 C

ATOM 811 CD2 PHE 104 26.385 30.999 47.922 1.00 0.00 C

ATOM 812 CE1 PHE 104 27.357 33.378 46.955 1.00 0.00 C

ATOM 813 CE2 PHE 104 27.701 31.104 47.549 1.00 0.00 C

ATOM 814 CZ PHE 104 28.182 32.295 47.068 1.00 0.00 C

ATOM 815 N ILE 105 23.372 34.980 49.249 1.00 0.00 N

ATOM 816 CA ILE 105 23.726 36.337 48.939 1.00 0.00 C

ATOM 817 C ILE 105 23.331 37.299 50.017 1.00 0.00 C

ATOM 818 O ILE 105 22.298 37.151 50.669 1.00 0.00 O

ATOM 819 CB ILE 105 22.959 36.818 47.735 1.00 0.00 C

ATOM 820 CG1 ILE 105 23.012 35.793 46.585 1.00 0.00 C

ATOM 821 CG2 ILE 105 23.498 38.206 47.348 1.00 0.00 C

ATOM 822 CD1 ILE 105 22.033 36.090 45.450 1.00 0.00 C

ATOM 823 N GLN 106 24.191 38.316 50.245 1.00 0.00 N

ATOM 824 CA GLN 106 23.798 39.402 51.090 1.00 0.00 C

ATOM 825 C GLN 106 23.651 40.581 50.178 1.00 0.00 C

ATOM 826 O GLN 106 24.588 40.957 49.476 1.00 0.00 O

ATOM 827 CB GLN 106 24.772 39.740 52.231 1.00 0.00 C

ATOM 828 CG GLN 106 26.154 40.222 51.806 1.00 0.00 C

ATOM 829 CD GLN 106 26.885 40.516 53.106 1.00 0.00 C

ATOM 830 NE2 GLN 106 28.223 40.285 53.131 1.00 0.00 N

ATOM 831 OE1 GLN 106 26.272 40.936 54.086 1.00 0.00 O

ATOM 832 N TYR 107 22.467 41.224 50.200 1.00 0.00 N

ATOM 833 CA TYR 107 22.150 42.262 49.257 1.00 0.00 C

ATOM 834 C TYR 107 22.244 43.563 49.980 1.00 0.00 C

ATOM 835 O TYR 107 22.389 43.610 51.201 1.00 0.00 O

ATOM 836 CB TYR 107 20.697 42.223 48.743 1.00 0.00 C

ATOM 837 CG TYR 107 20.421 40.953 48.013 1.00 0.00 C

ATOM 838 CD1 TYR 107 20.753 40.812 46.685 1.00 0.00 C

ATOM 839 CD2 TYR 107 19.806 39.905 48.660 1.00 0.00 C

ATOM 840 CE1 TYR 107 20.485 39.638 46.020 1.00 0.00 C

ATOM 841 CE2 TYR 107 19.537 38.730 48.000 1.00 0.00 C

ATOM 842 CZ TYR 107 19.877 38.596 46.676 1.00 0.00 C

ATOM 843 OH TYR 107 19.602 37.391 45.995 1.00 0.00 O

ATOM 844 N GLN 108 22.209 44.658 49.194 1.00 0.00 N

ATOM 845 CA GLN 108 22.264 45.996 49.700 1.00 0.00 C

ATOM 846 C GLN 108 23.531 46.151 50.473 1.00 0.00 C

ATOM 847 O GLN 108 23.582 46.901 51.447 1.00 0.00 O

ATOM 848 CB GLN 108 21.106 46.347 50.651 1.00 0.00 C

ATOM 849 CG GLN 108 19.719 46.201 50.026 1.00 0.00 C

ATOM 850 CD GLN 108 19.355 44.725 50.073 1.00 0.00 C

ATOM 851 NE2 GLN 108 18.601 44.250 49.046 1.00 0.00 N

ATOM 852 OE1 GLN 108 19.734 44.012 51.001 1.00 0.00 O

ATOM 853 N GLN 109 24.603 45.447 50.062 1.00 0.00 N

ATOM 854 CA GLN 109 25.822 45.615 50.795 1.00 0.00 C

ATOM 855 C GLN 109 26.948 45.766 49.821 1.00 0.00 C

ATOM 856 O GLN 109 27.043 45.016 48.852 1.00 0.00 O

ATOM 857 CB GLN 109 26.203 44.424 51.674 1.00 0.00 C

ATOM 858 CG GLN 109 27.498 44.703 52.432 1.00 0.00 C

ATOM 859 CD GLN 109 28.062 43.372 52.871 1.00 0.00 C

ATOM 860 NE2 GLN 109 28.814 42.724 51.944 1.00 0.00 N

ATOM 861 OE1 GLN 109 27.847 42.926 53.997 1.00 0.00 O

ATOM 862 N LYS 110 27.856 46.734 50.065 1.00 0.00 N

ATOM 863 CA LYS 110 28.948 46.921 49.155 1.00 0.00 C

ATOM 864 C LYS 110 30.238 46.509 49.786 1.00 0.00 C

ATOM 865 O LYS 110 30.485 46.770 50.966 1.00 0.00 O

ATOM 866 CB LYS 110 29.183 48.371 48.705 1.00 0.00 C

ATOM 867 CG LYS 110 28.227 48.863 47.626 1.00 0.00 C

ATOM 868 CD LYS 110 28.426 50.341 47.297 1.00 0.00 C

ATOM 869 CE LYS 110 27.638 50.801 46.073 1.00 0.00 C

ATOM 870 NZ LYS 110 27.922 52.226 45.799 1.00 0.00 N

ATOM 871 N VAL 111 31.101 45.837 48.988 1.00 0.00 N

ATOM 872 CA VAL 111 32.401 45.530 49.506 1.00 0.00 C

ATOM 873 C VAL 111 33.284 46.709 49.267 1.00 0.00 C

ATOM 874 O VAL 111 33.497 47.125 48.131 1.00 0.00 O

ATOM 875 CB VAL 111 33.105 44.338 48.927 1.00 0.00 C

ATOM 876 CG1 VAL 111 32.483 43.064 49.492 1.00 0.00 C

ATOM 877 CG2 VAL 111 33.020 44.419 47.401 1.00 0.00 C

ATOM 878 N ILE 112 33.738 47.338 50.368 1.00 0.00 N

ATOM 879 CA ILE 112 34.625 48.463 50.319 1.00 0.00 C

ATOM 880 C ILE 112 36.059 48.083 50.066 1.00 0.00 C

ATOM 881 O ILE 112 36.719 48.668 49.211 1.00 0.00 O

ATOM 882 CB ILE 112 34.589 49.273 51.586 1.00 0.00 C

ATOM 883 CG1 ILE 112 35.092 48.448 52.783 1.00 0.00 C

ATOM 884 CG2 ILE 112 33.158 49.807 51.763 1.00 0.00 C

ATOM 885 CD1 ILE 112 35.356 49.290 54.030 1.00 0.00 C

ATOM 886 N SER 113 36.600 47.095 50.813 1.00 0.00 N

ATOM 887 CA SER 113 38.002 46.799 50.667 1.00 0.00 C

ATOM 888 C SER 113 38.210 45.346 50.911 1.00 0.00 C

ATOM 889 O SER 113 37.440 44.698 51.618 1.00 0.00 O

ATOM 890 CB SER 113 38.900 47.558 51.660 1.00 0.00 C

ATOM 891 OG SER 113 38.636 47.127 52.987 1.00 0.00 O

ATOM 892 N ALA 114 39.251 44.792 50.265 1.00 0.00 N

ATOM 893 CA ALA 114 39.657 43.454 50.557 1.00 0.00 C

ATOM 894 C ALA 114 41.138 43.522 50.637 1.00 0.00 C

ATOM 895 O ALA 114 41.772 44.200 49.835 1.00 0.00 O

ATOM 896 CB ALA 114 39.256 42.405 49.504 1.00 0.00 C

ATOM 897 N ASN 115 41.740 42.850 51.631 1.00 0.00 N

ATOM 898 CA ASN 115 43.163 42.935 51.735 1.00 0.00 C

ATOM 899 C ASN 115 43.703 41.551 51.730 1.00 0.00 C

ATOM 900 O ASN 115 43.209 40.678 52.443 1.00 0.00 O

ATOM 901 CB ASN 115 43.653 43.627 53.021 1.00 0.00 C

ATOM 902 CG ASN 115 43.196 42.807 54.219 1.00 0.00 C

ATOM 903 ND2 ASN 115 41.871 42.862 54.521 1.00 0.00 N

ATOM 904 OD1 ASN 115 43.996 42.139 54.872 1.00 0.00 O

ATOM 905 N TRP 116 44.728 41.319 50.889 1.00 0.00 N

ATOM 906 CA TRP 116 45.339 40.032 50.851 1.00 0.00 C

ATOM 907 C TRP 116 46.299 40.009 51.985 1.00 0.00 C

ATOM 908 O TRP 116 47.056 40.958 52.188 1.00 0.00 O

ATOM 909 CB TRP 116 46.097 39.762 49.539 1.00 0.00 C

ATOM 910 CG TRP 116 46.757 38.407 49.457 1.00 0.00 C

ATOM 911 CD1 TRP 116 46.197 37.190 49.201 1.00 0.00 C

ATOM 912 CD2 TRP 116 48.166 38.180 49.618 1.00 0.00 C

ATOM 913 CE2 TRP 116 48.385 36.813 49.451 1.00 0.00 C

ATOM 914 CE3 TRP 116 49.194 39.041 49.879 1.00 0.00 C

ATOM 915 NE1 TRP 116 47.167 36.218 49.198 1.00 0.00 N

ATOM 916 CZ2 TRP 116 49.640 36.283 49.543 1.00 0.00 C

ATOM 917 CZ3 TRP 116 50.459 38.502 49.974 1.00 0.00 C

ATOM 918 CH2 TRP 116 50.676 37.150 49.810 1.00 0.00 C

ATOM 919 N GLN 117 46.264 38.927 52.779 1.00 0.00 N

ATOM 920 CA GLN 117 47.180 38.825 53.869 1.00 0.00 C

ATOM 921 C GLN 117 48.171 37.793 53.464 1.00 0.00 C

ATOM 922 O GLN 117 47.848 36.616 53.324 1.00 0.00 O

ATOM 923 CB GLN 117 46.494 38.445 55.193 1.00 0.00 C

ATOM 924 CG GLN 117 45.756 37.108 55.171 1.00 0.00 C

ATOM 925 CD GLN 117 44.860 37.086 56.402 1.00 0.00 C

ATOM 926 NE2 GLN 117 44.922 38.174 57.216 1.00 0.00 N

ATOM 927 OE1 GLN 117 44.123 36.128 56.634 1.00 0.00 O

ATOM 928 N SER 118 49.425 38.237 53.270 1.00 0.00 N

ATOM 929 CA SER 118 50.449 37.392 52.742 1.00 0.00 C

ATOM 930 C SER 118 50.668 36.205 53.622 1.00 0.00 C

ATOM 931 O SER 118 50.683 35.074 53.142 1.00 0.00 O

ATOM 932 CB SER 118 51.798 38.119 52.596 1.00 0.00 C

ATOM 933 OG SER 118 52.288 38.502 53.873 1.00 0.00 O

ATOM 934 N ASP 119 50.831 36.415 54.938 1.00 0.00 N

ATOM 935 CA ASP 119 51.155 35.299 55.778 1.00 0.00 C

ATOM 936 C ASP 119 50.046 34.290 55.796 1.00 0.00 C

ATOM 937 O ASP 119 50.289 33.096 55.625 1.00 0.00 O

ATOM 938 CB ASP 119 51.459 35.713 57.231 1.00 0.00 C

ATOM 939 CG ASP 119 50.222 36.358 57.840 1.00 0.00 C

ATOM 940 OD1 ASP 119 49.469 37.037 57.092 1.00 0.00 O

ATOM 941 OD2 ASP 119 50.018 36.184 59.070 1.00 0.00 O

ATOM 942 N LYS 120 48.797 34.747 56.003 1.00 0.00 N

ATOM 943 CA LYS 120 47.658 33.877 56.112 1.00 0.00 C

ATOM 944 C LYS 120 47.318 33.254 54.792 1.00 0.00 C

ATOM 945 O LYS 120 46.867 32.111 54.745 1.00 0.00 O

ATOM 946 CB LYS 120 46.402 34.592 56.625 1.00 0.00 C

ATOM 947 CG LYS 120 46.522 35.082 58.069 1.00 0.00 C

ATOM 948 CD LYS 120 46.723 33.957 59.086 1.00 0.00 C

ATOM 949 CE LYS 120 46.845 34.451 60.529 1.00 0.00 C

ATOM 950 NZ LYS 120 45.553 35.011 60.986 1.00 0.00 N

ATOM 951 N GLY 121 47.494 33.996 53.682 1.00 0.00 N

ATOM 952 CA GLY 121 47.143 33.459 52.398 1.00 0.00 C

ATOM 953 C GLY 121 45.655 33.550 52.259 1.00 0.00 C

ATOM 954 O GLY 121 45.032 32.746 51.565 1.00 0.00 O

ATOM 955 N LEU 122 45.039 34.529 52.946 1.00 0.00 N

ATOM 956 CA LEU 122 43.615 34.687 52.879 1.00 0.00 C

ATOM 957 C LEU 122 43.313 36.102 52.515 1.00 0.00 C

ATOM 958 O LEU 122 44.194 36.960 52.481 1.00 0.00 O

ATOM 959 CB LEU 122 42.893 34.449 54.215 1.00 0.00 C

ATOM 960 CG LEU 122 42.988 33.013 54.756 1.00 0.00 C

ATOM 961 CD1 LEU 122 42.235 32.887 56.088 1.00 0.00 C

ATOM 962 CD2 LEU 122 42.526 31.980 53.717 1.00 0.00 C

ATOM 963 N TRP 123 42.029 36.361 52.205 1.00 0.00 N

ATOM 964 CA TRP 123 41.582 37.685 51.898 1.00 0.00 C

ATOM 965 C TRP 123 40.658 38.106 52.991 1.00 0.00 C

ATOM 966 O TRP 123 39.784 37.348 53.407 1.00 0.00 O

ATOM 967 CB TRP 123 40.732 37.765 50.626 1.00 0.00 C

ATOM 968 CG TRP 123 41.501 37.562 49.352 1.00 0.00 C

ATOM 969 CD1 TRP 123 41.841 36.414 48.699 1.00 0.00 C

ATOM 970 CD2 TRP 123 42.008 38.647 48.568 1.00 0.00 C

ATOM 971 CE2 TRP 123 42.639 38.091 47.459 1.00 0.00 C

ATOM 972 CE3 TRP 123 41.941 39.998 48.755 1.00 0.00 C

ATOM 973 NE1 TRP 123 42.532 36.719 47.551 1.00 0.00 N

ATOM 974 CZ2 TRP 123 43.216 38.883 46.514 1.00 0.00 C

ATOM 975 CZ3 TRP 123 42.536 40.796 47.805 1.00 0.00 C

ATOM 976 CH2 TRP 123 43.161 40.247 46.705 1.00 0.00 C

ATOM 977 N SER 124 40.836 39.340 53.496 1.00 0.00 N

ATOM 978 CA SER 124 39.914 39.828 54.476 1.00 0.00 C

ATOM 979 C SER 124 39.081 40.822 53.738 1.00 0.00 C

ATOM 980 O SER 124 39.608 41.772 53.162 1.00 0.00 O

ATOM 981 CB SER 124 40.590 40.556 55.649 1.00 0.00 C

ATOM 982 OG SER 124 39.610 41.008 56.572 1.00 0.00 O

ATOM 983 N VAL 125 37.747 40.632 53.724 1.00 0.00 N

ATOM 984 CA VAL 125 36.968 41.540 52.939 1.00 0.00 C

ATOM 985 C VAL 125 36.091 42.300 53.884 1.00 0.00 C

ATOM 986 O VAL 125 35.524 41.733 54.817 1.00 0.00 O

ATOM 987 CB VAL 125 36.112 40.844 51.915 1.00 0.00 C

ATOM 988 CG1 VAL 125 34.654 40.791 52.392 1.00 0.00 C

ATOM 989 CG2 VAL 125 36.357 41.480 50.541 1.00 0.00 C

ATOM 990 N ARG 126 35.969 43.626 53.672 1.00 0.00 N

ATOM 991 CA ARG 126 35.186 44.429 54.567 1.00 0.00 C

ATOM 992 C ARG 126 34.033 44.977 53.796 1.00 0.00 C

ATOM 993 O ARG 126 34.167 45.341 52.628 1.00 0.00 O

ATOM 994 CB ARG 126 35.970 45.616 55.146 1.00 0.00 C

ATOM 995 CG ARG 126 37.217 45.157 55.900 1.00 0.00 C

ATOM 996 CD ARG 126 38.044 46.290 56.504 1.00 0.00 C

ATOM 997 NE ARG 126 39.328 45.682 56.953 1.00 0.00 N

ATOM 998 CZ ARG 126 39.384 45.006 58.137 1.00 0.00 C

ATOM 999 NH1 ARG 126 38.264 44.888 58.908 1.00 0.00 N

ATOM 1000 NH2 ARG 126 40.557 44.440 58.543 1.00 0.00 N

ATOM 1001 N VAL 127 32.847 45.031 54.431 1.00 0.00 N

ATOM 1002 CA VAL 127 31.724 45.505 53.690 1.00 0.00 C

ATOM 1003 C VAL 127 30.923 46.507 54.456 1.00 0.00 C

ATOM 1004 O VAL 127 30.460 46.241 55.562 1.00 0.00 O

ATOM 1005 CB VAL 127 30.810 44.385 53.317 1.00 0.00 C

ATOM 1006 CG1 VAL 127 31.553 43.472 52.325 1.00 0.00 C

ATOM 1007 CG2 VAL 127 30.415 43.662 54.617 1.00 0.00 C

ATOM 1008 N GLU 128 30.836 47.727 53.891 1.00 0.00 N

ATOM 1009 CA GLU 128 29.956 48.803 54.259 1.00 0.00 C

ATOM 1010 C GLU 128 29.628 48.852 55.711 1.00 0.00 C

ATOM 1011 O GLU 128 28.442 48.890 56.035 1.00 0.00 O

ATOM 1012 CB GLU 128 28.640 48.796 53.456 1.00 0.00 C

ATOM 1013 CG GLU 128 27.794 50.058 53.639 1.00 0.00 C

ATOM 1014 CD GLU 128 28.515 51.213 52.959 1.00 0.00 C

ATOM 1015 OE1 GLU 128 29.642 50.984 52.444 1.00 0.00 O

ATOM 1016 OE2 GLU 128 27.950 52.339 52.945 1.00 0.00 O

ATOM 1017 N ASP 129 30.643 48.914 56.600 1.00 0.00 N

ATOM 1018 CA ASP 129 30.414 49.043 58.018 1.00 0.00 C

ATOM 1019 C ASP 129 29.331 48.104 58.456 1.00 0.00 C

ATOM 1020 O ASP 129 28.205 48.529 58.710 1.00 0.00 O

ATOM 1021 CB ASP 129 29.995 50.470 58.407 1.00 0.00 C

ATOM 1022 CG ASP 129 31.161 51.398 58.096 1.00 0.00 C

ATOM 1023 OD1 ASP 129 32.255 51.200 58.687 1.00 0.00 O

ATOM 1024 OD2 ASP 129 30.975 52.312 57.248 1.00 0.00 O

ATOM 1025 N GLY 130 29.633 46.795 58.527 1.00 0.00 N

ATOM 1026 CA GLY 130 28.639 45.814 58.862 1.00 0.00 C

ATOM 1027 C GLY 130 27.997 46.207 60.152 1.00 0.00 C

ATOM 1028 O GLY 130 28.484 47.091 60.855 1.00 0.00 O

ATOM 1029 N ARG 131 26.868 45.549 60.491 1.00 0.00 N

ATOM 1030 CA ARG 131 26.128 45.927 61.658 1.00 0.00 C

ATOM 1031 C ARG 131 26.875 45.486 62.871 1.00 0.00 C

ATOM 1032 O ARG 131 26.612 44.432 63.447 1.00 0.00 O

ATOM 1033 CB ARG 131 24.724 45.298 61.720 1.00 0.00 C

ATOM 1034 CG ARG 131 23.918 45.692 62.961 1.00 0.00 C

ATOM 1035 CD ARG 131 23.497 47.164 62.986 1.00 0.00 C

ATOM 1036 NE ARG 131 24.737 47.980 63.097 1.00 0.00 N

ATOM 1037 CZ ARG 131 25.275 48.233 64.326 1.00 0.00 C

ATOM 1038 NH1 ARG 131 24.662 47.756 65.448 1.00 0.00 N

ATOM 1039 NH2 ARG 131 26.429 48.953 64.431 1.00 0.00 N

ATOM 1040 N THR 132 27.842 46.329 63.268 1.00 0.00 N

ATOM 1041 CA THR 132 28.673 46.205 64.423 1.00 0.00 C

ATOM 1042 C THR 132 29.295 47.552 64.519 1.00 0.00 C

ATOM 1043 O THR 132 29.084 48.383 63.637 1.00 0.00 O

ATOM 1044 CB THR 132 29.796 45.222 64.258 1.00 0.00 C

ATOM 1045 CG2 THR 132 29.217 43.815 64.041 1.00 0.00 C

ATOM 1046 OG1 THR 132 30.612 45.597 63.158 1.00 0.00 O

ATOM 1047 N ALA 133 30.061 47.846 65.583 1.00 0.00 N

ATOM 1048 CA ALA 133 30.676 49.130 65.485 1.00 0.00 C

ATOM 1049 C ALA 133 31.902 48.876 64.679 1.00 0.00 C

ATOM 1050 O ALA 133 33.022 48.997 65.171 1.00 0.00 O

ATOM 1051 CB ALA 133 31.120 49.703 66.842 1.00 0.00 C

ATOM 1052 N GLN 134 31.689 48.552 63.389 1.00 0.00 N

ATOM 1053 CA GLN 134 32.745 48.229 62.478 1.00 0.00 C

ATOM 1054 C GLN 134 32.103 48.014 61.146 1.00 0.00 C

ATOM 1055 O GLN 134 30.977 48.442 60.895 1.00 0.00 O

ATOM 1056 CB GLN 134 33.487 46.906 62.770 1.00 0.00 C

ATOM 1057 CG GLN 134 34.331 46.867 64.048 1.00 0.00 C

ATOM 1058 CD GLN 134 33.484 46.295 65.179 1.00 0.00 C

ATOM 1059 NE2 GLN 134 34.145 45.956 66.318 1.00 0.00 N

ATOM 1060 OE1 GLN 134 32.269 46.154 65.054 1.00 0.00 O

ATOM 1061 N ILE 135 32.850 47.340 60.255 1.00 0.00 N

ATOM 1062 CA ILE 135 32.424 46.973 58.949 1.00 0.00 C

ATOM 1063 C ILE 135 32.433 45.478 58.987 1.00 0.00 C

ATOM 1064 O ILE 135 33.293 44.884 59.635 1.00 0.00 O

ATOM 1065 CB ILE 135 33.416 47.404 57.912 1.00 0.00 C

ATOM 1066 CG1 ILE 135 33.600 48.929 57.939 1.00 0.00 C

ATOM 1067 CG2 ILE 135 32.950 46.873 56.558 1.00 0.00 C

ATOM 1068 CD1 ILE 135 34.811 49.410 57.141 1.00 0.00 C

ATOM 1069 N ARG 136 31.458 44.818 58.334 1.00 0.00 N

ATOM 1070 CA ARG 136 31.465 43.388 58.405 1.00 0.00 C

ATOM 1071 C ARG 136 32.697 42.925 57.712 1.00 0.00 C

ATOM 1072 O ARG 136 33.053 43.430 56.649 1.00 0.00 O

ATOM 1073 CB ARG 136 30.250 42.715 57.741 1.00 0.00 C

ATOM 1074 CG ARG 136 28.953 42.900 58.532 1.00 0.00 C

ATOM 1075 CD ARG 136 27.734 42.227 57.899 1.00 0.00 C

ATOM 1076 NE ARG 136 27.414 42.957 56.640 1.00 0.00 N

ATOM 1077 CZ ARG 136 26.550 44.013 56.664 1.00 0.00 C

ATOM 1078 NH1 ARG 136 25.993 44.415 57.844 1.00 0.00 N

ATOM 1079 NH2 ARG 136 26.241 44.667 55.507 1.00 0.00 N

ATOM 1080 N THR 137 33.406 41.957 58.323 1.00 0.00 N

ATOM 1081 CA THR 137 34.602 41.475 57.708 1.00 0.00 C

ATOM 1082 C THR 137 34.471 39.998 57.559 1.00 0.00 C

ATOM 1083 O THR 137 33.975 39.312 58.452 1.00 0.00 O

ATOM 1084 CB THR 137 35.841 41.733 58.514 1.00 0.00 C

ATOM 1085 CG2 THR 137 37.050 41.171 57.747 1.00 0.00 C

ATOM 1086 OG1 THR 137 36.003 43.127 58.728 1.00 0.00 O

ATOM 1087 N VAL 138 34.903 39.471 56.398 1.00 0.00 N

ATOM 1088 CA VAL 138 34.848 38.058 56.181 1.00 0.00 C

ATOM 1089 C VAL 138 36.207 37.639 55.717 1.00 0.00 C

ATOM 1090 O VAL 138 36.932 38.421 55.103 1.00 0.00 O

ATOM 1091 CB VAL 138 33.880 37.637 55.112 1.00 0.00 C

ATOM 1092 CG1 VAL 138 32.466 38.079 55.524 1.00 0.00 C

ATOM 1093 CG2 VAL 138 34.354 38.199 53.763 1.00 0.00 C

ATOM 1094 N GLU 139 36.595 36.385 56.027 1.00 0.00 N

ATOM 1095 CA GLU 139 37.867 35.878 55.601 1.00 0.00 C

ATOM 1096 C GLU 139 37.589 34.844 54.560 1.00 0.00 C

ATOM 1097 O GLU 139 36.707 34.004 54.736 1.00 0.00 O

ATOM 1098 CB GLU 139 38.664 35.188 56.720 1.00 0.00 C

ATOM 1099 CG GLU 139 39.140 36.149 57.810 1.00 0.00 C

ATOM 1100 CD GLU 139 40.181 37.072 57.193 1.00 0.00 C

ATOM 1101 OE1 GLU 139 40.411 36.964 55.958 1.00 0.00 O

ATOM 1102 OE2 GLU 139 40.759 37.899 57.947 1.00 0.00 O

ATOM 1103 N CYS 140 38.335 34.886 53.436 1.00 0.00 N

ATOM 1104 CA CYS 140 38.074 33.955 52.376 1.00 0.00 C

ATOM 1105 C CYS 140 39.384 33.421 51.903 1.00 0.00 C

ATOM 1106 O CYS 140 40.376 34.147 51.862 1.00 0.00 O

ATOM 1107 CB CYS 140 37.545 34.618 51.101 1.00 0.00 C

ATOM 1108 SG CYS 140 36.374 35.951 51.432 1.00 0.00 S

ATOM 1109 N ARG 141 39.427 32.122 51.549 1.00 0.00 N

ATOM 1110 CA ARG 141 40.610 31.594 50.936 1.00 0.00 C

ATOM 1111 C ARG 141 40.726 32.220 49.582 1.00 0.00 C

ATOM 1112 O ARG 141 41.796 32.680 49.186 1.00 0.00 O

ATOM 1113 CB ARG 141 40.555 30.076 50.674 1.00 0.00 C

ATOM 1114 CG ARG 141 40.737 29.190 51.905 1.00 0.00 C

ATOM 1115 CD ARG 141 40.817 27.700 51.558 1.00 0.00 C

ATOM 1116 NE ARG 141 41.938 27.528 50.589 1.00 0.00 N

ATOM 1117 CZ ARG 141 42.468 26.291 50.357 1.00 0.00 C

ATOM 1118 NH1 ARG 141 42.015 25.208 51.053 1.00 0.00 N

ATOM 1119 NH2 ARG 141 43.452 26.135 49.424 1.00 0.00 N

ATOM 1120 N TRP 142 39.603 32.267 48.835 1.00 0.00 N

ATOM 1121 CA TRP 142 39.657 32.785 47.498 1.00 0.00 C

ATOM 1122 C TRP 142 38.655 33.884 47.357 1.00 0.00 C

ATOM 1123 O TRP 142 37.571 33.846 47.939 1.00 0.00 O

ATOM 1124 CB TRP 142 39.333 31.742 46.414 1.00 0.00 C

ATOM 1125 CG TRP 142 40.359 30.642 46.273 1.00 0.00 C

ATOM 1126 CD1 TRP 142 41.467 30.581 45.478 1.00 0.00 C

ATOM 1127 CD2 TRP 142 40.310 29.403 46.996 1.00 0.00 C

ATOM 1128 CE2 TRP 142 41.409 28.645 46.593 1.00 0.00 C

ATOM 1129 CE3 TRP 142 39.419 28.934 47.919 1.00 0.00 C

ATOM 1130 NE1 TRP 142 42.112 29.381 45.661 1.00 0.00 N

ATOM 1131 CZ2 TRP 142 41.636 27.402 47.110 1.00 0.00 C

ATOM 1132 CZ3 TRP 142 39.651 27.681 48.439 1.00 0.00 C

ATOM 1133 CH2 TRP 142 40.738 26.930 48.042 1.00 0.00 C

ATOM 1134 N LEU 143 39.016 34.907 46.558 1.00 0.00 N

ATOM 1135 CA LEU 143 38.139 36.020 46.357 1.00 0.00 C

ATOM 1136 C LEU 143 37.810 35.999 44.894 1.00 0.00 C

ATOM 1137 O LEU 143 38.709 35.949 44.055 1.00 0.00 O

ATOM 1138 CB LEU 143 38.849 37.356 46.646 1.00 0.00 C

ATOM 1139 CG LEU 143 37.937 38.572 46.895 1.00 0.00 C

ATOM 1140 CD1 LEU 143 36.872 38.741 45.811 1.00 0.00 C

ATOM 1141 CD2 LEU 143 37.376 38.562 48.320 1.00 0.00 C

ATOM 1142 N PHE 144 36.508 36.012 44.544 1.00 0.00 N

ATOM 1143 CA PHE 144 36.147 35.995 43.156 1.00 0.00 C

ATOM 1144 C PHE 144 35.437 37.282 42.884 1.00 0.00 C

ATOM 1145 O PHE 144 34.434 37.591 43.527 1.00 0.00 O

ATOM 1146 CB PHE 144 35.191 34.844 42.806 1.00 0.00 C

ATOM 1147 CG PHE 144 34.987 34.855 41.333 1.00 0.00 C

ATOM 1148 CD1 PHE 144 35.903 34.251 40.502 1.00 0.00 C

ATOM 1149 CD2 PHE 144 33.881 35.459 40.782 1.00 0.00 C

ATOM 1150 CE1 PHE 144 35.722 34.254 39.140 1.00 0.00 C

ATOM 1151 CE2 PHE 144 33.695 35.465 39.420 1.00 0.00 C

ATOM 1152 CZ PHE 144 34.617 34.862 38.598 1.00 0.00 C

ATOM 1153 N SER 145 35.942 38.071 41.913 1.00 0.00 N

ATOM 1154 CA SER 145 35.321 39.335 41.652 1.00 0.00 C

ATOM 1155 C SER 145 34.471 39.223 40.415 1.00 0.00 C

ATOM 1156 O SER 145 34.968 39.218 39.291 1.00 0.00 O

ATOM 1157 CB SER 145 36.306 40.480 41.369 1.00 0.00 C

ATOM 1158 OG SER 145 36.956 40.865 42.567 1.00 0.00 O

ATOM 1159 N ALA 146 33.155 39.055 40.630 1.00 0.00 N

ATOM 1160 CA ALA 146 32.073 39.023 39.680 1.00 0.00 C

ATOM 1161 C ALA 146 31.694 40.421 39.208 1.00 0.00 C

ATOM 1162 O ALA 146 30.905 40.548 38.281 1.00 0.00 O

ATOM 1163 CB ALA 146 30.802 38.380 40.258 1.00 0.00 C

ATOM 1164 N GLY 147 31.981 41.461 40.025 1.00 0.00 N

ATOM 1165 CA GLY 147 31.935 42.897 39.802 1.00 0.00 C

ATOM 1166 C GLY 147 31.316 43.399 38.547 1.00 0.00 C

ATOM 1167 O GLY 147 30.381 44.194 38.578 1.00 0.00 O

ATOM 1168 N GLY 148 31.951 43.111 37.402 1.00 0.00 N

ATOM 1169 CA GLY 148 31.372 43.602 36.197 1.00 0.00 C

ATOM 1170 C GLY 148 32.454 44.184 35.327 1.00 0.00 C

ATOM 1171 O GLY 148 33.605 44.358 35.721 1.00 0.00 O

ATOM 1172 N TYR 149 32.030 44.538 34.103 1.00 0.00 N

ATOM 1173 CA TYR 149 32.632 45.263 33.013 1.00 0.00 C

ATOM 1174 C TYR 149 32.041 46.577 33.485 1.00 0.00 C

ATOM 1175 O TYR 149 31.511 47.329 32.670 1.00 0.00 O

ATOM 1176 CB TYR 149 31.671 44.986 31.856 1.00 0.00 C

ATOM 1177 CG TYR 149 31.521 43.532 31.624 1.00 0.00 C

ATOM 1178 CD1 TYR 149 30.606 42.837 32.373 1.00 0.00 C

ATOM 1179 CD2 TYR 149 32.255 42.876 30.668 1.00 0.00 C

ATOM 1180 CE1 TYR 149 30.434 41.494 32.197 1.00 0.00 C

ATOM 1181 CE2 TYR 149 32.076 41.528 30.491 1.00 0.00 C

ATOM 1182 CZ TYR 149 31.172 40.832 31.255 1.00 0.00 C

ATOM 1183 OH TYR 149 30.993 39.444 31.078 1.00 0.00 O

ATOM 1184 N TYR 150 32.165 46.920 34.800 1.00 0.00 N

ATOM 1185 CA TYR 150 31.125 47.790 35.280 1.00 0.00 C

ATOM 1186 C TYR 150 31.270 49.068 35.887 1.00 0.00 C

ATOM 1187 O TYR 150 32.065 49.388 36.761 1.00 0.00 O

ATOM 1188 CB TYR 150 29.922 47.092 35.924 1.00 0.00 C

ATOM 1189 CG TYR 150 28.981 47.097 34.773 1.00 0.00 C

ATOM 1190 CD1 TYR 150 29.118 46.219 33.728 1.00 0.00 C

ATOM 1191 CD2 TYR 150 27.973 48.032 34.738 1.00 0.00 C

ATOM 1192 CE1 TYR 150 28.241 46.279 32.675 1.00 0.00 C

ATOM 1193 CE2 TYR 150 27.095 48.092 33.686 1.00 0.00 C

ATOM 1194 CZ TYR 150 27.231 47.207 32.646 1.00 0.00 C

ATOM 1195 OH TYR 150 26.345 47.249 31.549 1.00 0.00 O

ATOM 1196 N ARG 151 30.307 49.799 35.342 1.00 0.00 N

ATOM 1197 CA ARG 151 29.838 51.027 35.774 1.00 0.00 C

ATOM 1198 C ARG 151 30.939 51.917 36.271 1.00 0.00 C

ATOM 1199 O ARG 151 32.117 51.784 35.950 1.00 0.00 O

ATOM 1200 CB ARG 151 28.828 50.893 36.932 1.00 0.00 C

ATOM 1201 CG ARG 151 27.468 50.330 36.519 1.00 0.00 C

ATOM 1202 CD ARG 151 26.468 50.216 37.673 1.00 0.00 C

ATOM 1203 NE ARG 151 26.918 49.106 38.562 1.00 0.00 N

ATOM 1204 CZ ARG 151 27.800 49.346 39.577 1.00 0.00 C

ATOM 1205 NH1 ARG 151 28.264 50.610 39.796 1.00 0.00 N

ATOM 1206 NH2 ARG 151 28.213 48.321 40.379 1.00 0.00 N

ATOM 1207 N TYR 152 30.469 52.931 37.014 1.00 0.00 N

ATOM 1208 CA TYR 152 31.078 53.978 37.782 1.00 0.00 C

ATOM 1209 C TYR 152 30.078 53.957 38.883 1.00 0.00 C

ATOM 1210 O TYR 152 29.522 52.892 39.146 1.00 0.00 O

ATOM 1211 CB TYR 152 31.037 55.340 37.026 1.00 0.00 C

ATOM 1212 CG TYR 152 31.408 56.545 37.848 1.00 0.00 C

ATOM 1213 CD1 TYR 152 32.697 56.826 38.229 1.00 0.00 C

ATOM 1214 CD2 TYR 152 30.429 57.435 38.226 1.00 0.00 C

ATOM 1215 CE1 TYR 152 32.997 57.943 38.976 1.00 0.00 C

ATOM 1216 CE2 TYR 152 30.710 58.555 38.971 1.00 0.00 C

ATOM 1217 CZ TYR 152 32.002 58.813 39.352 1.00 0.00 C

ATOM 1218 OH TYR 152 32.302 59.960 40.118 1.00 0.00 O

ATOM 1219 N ASP 153 29.825 55.055 39.608 1.00 0.00 N

ATOM 1220 CA ASP 153 28.648 54.972 40.410 1.00 0.00 C

ATOM 1221 C ASP 153 27.550 55.184 39.409 1.00 0.00 C

ATOM 1222 O ASP 153 26.915 56.236 39.371 1.00 0.00 O

ATOM 1223 CB ASP 153 28.550 56.070 41.484 1.00 0.00 C

ATOM 1224 CG ASP 153 29.535 55.726 42.592 1.00 0.00 C

ATOM 1225 OD1 ASP 153 30.158 54.633 42.511 1.00 0.00 O

ATOM 1226 OD2 ASP 153 29.673 56.548 43.537 1.00 0.00 O

ATOM 1227 N GLN 154 27.309 54.153 38.568 1.00 0.00 N

ATOM 1228 CA GLN 154 26.375 54.198 37.492 1.00 0.00 C

ATOM 1229 C GLN 154 26.602 55.453 36.721 1.00 0.00 C

ATOM 1230 O GLN 154 25.730 56.316 36.653 1.00 0.00 O

ATOM 1231 CB GLN 154 24.911 54.153 37.953 1.00 0.00 C

ATOM 1232 CG GLN 154 24.531 52.835 38.629 1.00 0.00 C

ATOM 1233 CD GLN 154 25.176 52.819 40.007 1.00 0.00 C

ATOM 1234 NE2 GLN 154 25.435 51.599 40.548 1.00 0.00 N

ATOM 1235 OE1 GLN 154 25.446 53.869 40.588 1.00 0.00 O

ATOM 1236 N GLY 155 27.825 55.635 36.191 1.00 0.00 N

ATOM 1237 CA GLY 155 27.973 56.663 35.209 1.00 0.00 C

ATOM 1238 C GLY 155 28.587 57.932 35.673 1.00 0.00 C

ATOM 1239 O GLY 155 28.343 58.437 36.769 1.00 0.00 O

ATOM 1240 N PHE 156 29.395 58.481 34.745 1.00 0.00 N

ATOM 1241 CA PHE 156 30.114 59.693 34.905 1.00 0.00 C

ATOM 1242 C PHE 156 29.146 60.786 34.623 1.00 0.00 C

ATOM 1243 O PHE 156 28.345 60.690 33.695 1.00 0.00 O

ATOM 1244 CB PHE 156 31.284 59.852 33.923 1.00 0.00 C

ATOM 1245 CG PHE 156 31.931 61.161 34.215 1.00 0.00 C

ATOM 1246 CD1 PHE 156 32.873 61.267 35.209 1.00 0.00 C

ATOM 1247 CD2 PHE 156 31.587 62.282 33.495 1.00 0.00 C

ATOM 1248 CE1 PHE 156 33.469 62.475 35.479 1.00 0.00 C

ATOM 1249 CE2 PHE 156 32.181 63.493 33.762 1.00 0.00 C

ATOM 1250 CZ PHE 156 33.124 63.591 34.755 1.00 0.00 C

ATOM 1251 N SER 157 29.172 61.857 35.434 1.00 0.00 N

ATOM 1252 CA SER 157 28.295 62.941 35.127 1.00 0.00 C

ATOM 1253 C SER 157 29.181 64.023 34.611 1.00 0.00 C

ATOM 1254 O SER 157 30.103 64.462 35.297 1.00 0.00 O

ATOM 1255 CB SER 157 27.527 63.502 36.334 1.00 0.00 C

ATOM 1256 OG SER 157 28.432 64.106 37.244 1.00 0.00 O

ATOM 1257 N PRO 158 28.930 64.452 33.404 1.00 0.00 N

ATOM 1258 CA PRO 158 29.720 65.488 32.801 1.00 0.00 C

ATOM 1259 C PRO 158 29.603 66.647 33.722 1.00 0.00 C

ATOM 1260 O PRO 158 28.535 66.824 34.303 1.00 0.00 O

ATOM 1261 CB PRO 158 29.040 65.782 31.469 1.00 0.00 C

ATOM 1262 CG PRO 158 27.557 65.482 31.765 1.00 0.00 C

ATOM 1263 CD PRO 158 27.608 64.350 32.805 1.00 0.00 C

ATOM 1264 N ARG 159 30.668 67.443 33.905 1.00 0.00 N

ATOM 1265 CA ARG 159 30.479 68.547 34.789 1.00 0.00 C

ATOM 1266 C ARG 159 30.168 69.735 33.943 1.00 0.00 C

ATOM 1267 O ARG 159 31.060 70.425 33.453 1.00 0.00 O

ATOM 1268 CB ARG 159 31.707 68.859 35.672 1.00 0.00 C

ATOM 1269 CG ARG 159 33.004 69.163 34.911 1.00 0.00 C

ATOM 1270 CD ARG 159 33.962 67.974 34.817 1.00 0.00 C

ATOM 1271 NE ARG 159 33.402 67.022 33.818 1.00 0.00 N

ATOM 1272 CZ ARG 159 33.707 67.162 32.495 1.00 0.00 C

ATOM 1273 NH1 ARG 159 34.548 68.157 32.089 1.00 0.00 N

ATOM 1274 NH2 ARG 159 33.174 66.302 31.579 1.00 0.00 N

ATOM 1275 N PHE 160 28.867 70.000 33.735 1.00 0.00 N

ATOM 1276 CA PHE 160 28.541 71.181 32.999 1.00 0.00 C

ATOM 1277 C PHE 160 28.324 72.220 34.027 1.00 0.00 C

ATOM 1278 O PHE 160 27.767 71.945 35.088 1.00 0.00 O

ATOM 1279 CB PHE 160 27.238 71.128 32.164 1.00 0.00 C

ATOM 1280 CG PHE 160 27.312 70.117 31.062 1.00 0.00 C

ATOM 1281 CD1 PHE 160 28.045 70.352 29.922 1.00 0.00 C

ATOM 1282 CD2 PHE 160 26.646 68.919 31.169 1.00 0.00 C

ATOM 1283 CE1 PHE 160 28.105 69.417 28.913 1.00 0.00 C

ATOM 1284 CE2 PHE 160 26.701 67.982 30.162 1.00 0.00 C

ATOM 1285 CZ PHE 160 27.432 68.227 29.026 1.00 0.00 C

ATOM 1286 N GLU 161 28.788 73.446 33.755 1.00 0.00 N

ATOM 1287 CA GLU 161 28.567 74.463 34.727 1.00 0.00 C

ATOM 1288 C GLU 161 27.106 74.763 34.718 1.00 0.00 C

ATOM 1289 O GLU 161 26.480 74.818 33.661 1.00 0.00 O

ATOM 1290 CB GLU 161 29.326 75.769 34.436 1.00 0.00 C

ATOM 1291 CG GLU 161 28.960 76.394 33.089 1.00 0.00 C

ATOM 1292 CD GLU 161 29.557 75.518 31.997 1.00 0.00 C

ATOM 1293 OE1 GLU 161 30.805 75.546 31.826 1.00 0.00 O

ATOM 1294 OE2 GLU 161 28.769 74.797 31.327 1.00 0.00 O

ATOM 1295 N GLY 162 26.521 74.939 35.918 1.00 0.00 N

ATOM 1296 CA GLY 162 25.136 75.295 36.025 1.00 0.00 C

ATOM 1297 C GLY 162 24.264 74.084 35.877 1.00 0.00 C

ATOM 1298 O GLY 162 23.055 74.215 35.695 1.00 0.00 O

ATOM 1299 N SER 163 24.836 72.870 35.960 1.00 0.00 N

ATOM 1300 CA SER 163 24.037 71.685 35.790 1.00 0.00 C

ATOM 1301 C SER 163 23.030 71.594 36.898 1.00 0.00 C

ATOM 1302 O SER 163 21.876 71.231 36.674 1.00 0.00 O

ATOM 1303 CB SER 163 24.872 70.393 35.812 1.00 0.00 C

ATOM 1304 OG SER 163 25.486 70.225 37.082 1.00 0.00 O

ATOM 1305 N GLU 164 23.444 71.950 38.126 1.00 0.00 N

ATOM 1306 CA GLU 164 22.614 71.843 39.293 1.00 0.00 C

ATOM 1307 C GLU 164 21.447 72.769 39.162 1.00 0.00 C

ATOM 1308 O GLU 164 20.357 72.491 39.659 1.00 0.00 O

ATOM 1309 CB GLU 164 23.368 72.211 40.582 1.00 0.00 C

ATOM 1310 CG GLU 164 22.528 72.056 41.849 1.00 0.00 C

ATOM 1311 CD GLU 164 23.396 72.449 43.038 1.00 0.00 C

ATOM 1312 OE1 GLU 164 24.584 72.803 42.811 1.00 0.00 O

ATOM 1313 OE2 GLU 164 22.884 72.398 44.188 1.00 0.00 O

ATOM 1314 N GLN 165 21.673 73.907 38.489 1.00 0.00 N

ATOM 1315 CA GLN 165 20.734 74.975 38.315 1.00 0.00 C

ATOM 1316 C GLN 165 19.534 74.576 37.488 1.00 0.00 C

ATOM 1317 O GLN 165 18.455 75.136 37.674 1.00 0.00 O

ATOM 1318 CB GLN 165 21.457 76.239 37.811 1.00 0.00 C

ATOM 1319 CG GLN 165 20.596 77.374 37.277 1.00 0.00 C

ATOM 1320 CD GLN 165 20.877 77.335 35.792 1.00 0.00 C

ATOM 1321 NE2 GLN 165 20.752 78.502 35.104 1.00 0.00 N

ATOM 1322 OE1 GLN 165 21.253 76.286 35.271 1.00 0.00 O

ATOM 1323 N PHE 166 19.664 73.616 36.548 1.00 0.00 N

ATOM 1324 CA PHE 166 18.534 73.246 35.726 1.00 0.00 C

ATOM 1325 C PHE 166 17.363 72.836 36.577 1.00 0.00 C

ATOM 1326 O PHE 166 17.478 72.018 37.488 1.00 0.00 O

ATOM 1327 CB PHE 166 18.866 72.128 34.717 1.00 0.00 C

ATOM 1328 CG PHE 166 17.648 71.723 33.952 1.00 0.00 C

ATOM 1329 CD1 PHE 166 17.005 72.590 33.093 1.00 0.00 C

ATOM 1330 CD2 PHE 166 17.168 70.439 34.071 1.00 0.00 C

ATOM 1331 CE1 PHE 166 15.897 72.190 32.384 1.00 0.00 C

ATOM 1332 CE2 PHE 166 16.060 70.034 33.366 1.00 0.00 C

ATOM 1333 CZ PHE 166 15.420 70.909 32.521 1.00 0.00 C

ATOM 1334 N LYS 167 16.197 73.457 36.287 1.00 0.00 N

ATOM 1335 CA LYS 167 14.937 73.292 36.966 1.00 0.00 C

ATOM 1336 C LYS 167 14.278 71.968 36.693 1.00 0.00 C

ATOM 1337 O LYS 167 13.652 71.394 37.583 1.00 0.00 O

ATOM 1338 CB LYS 167 13.922 74.386 36.593 1.00 0.00 C

ATOM 1339 CG LYS 167 14.332 75.783 37.065 1.00 0.00 C

ATOM 1340 CD LYS 167 14.454 75.907 38.586 1.00 0.00 C

ATOM 1341 CE LYS 167 14.867 77.304 39.054 1.00 0.00 C

ATOM 1342 NZ LYS 167 14.961 77.336 40.531 1.00 0.00 N

ATOM 1343 N GLY 168 14.393 71.444 35.457 1.00 0.00 N

ATOM 1344 CA GLY 168 13.661 70.266 35.065 1.00 0.00 C

ATOM 1345 C GLY 168 14.305 69.038 35.624 1.00 0.00 C

ATOM 1346 O GLY 168 15.198 69.109 36.466 1.00 0.00 O

ATOM 1347 N GLN 169 13.828 67.858 35.176 1.00 0.00 N

ATOM 1348 CA GLN 169 14.354 66.630 35.694 1.00 0.00 C

ATOM 1349 C GLN 169 15.532 66.199 34.883 1.00 0.00 C

ATOM 1350 O GLN 169 15.588 66.400 33.670 1.00 0.00 O

ATOM 1351 CB GLN 169 13.327 65.479 35.745 1.00 0.00 C

ATOM 1352 CG GLN 169 12.826 65.015 34.378 1.00 0.00 C

ATOM 1353 CD GLN 169 11.724 63.984 34.594 1.00 0.00 C

ATOM 1354 NE2 GLN 169 11.873 62.791 33.959 1.00 0.00 N

ATOM 1355 OE1 GLN 169 10.754 64.234 35.308 1.00 0.00 O

ATOM 1356 N ILE 170 16.526 65.601 35.569 1.00 0.00 N

ATOM 1357 CA ILE 170 17.705 65.118 34.918 1.00 0.00 C

ATOM 1358 C ILE 170 17.701 63.640 35.123 1.00 0.00 C

ATOM 1359 O ILE 170 17.536 63.169 36.247 1.00 0.00 O

ATOM 1360 CB ILE 170 18.975 65.645 35.521 1.00 0.00 C

ATOM 1361 CG1 ILE 170 19.034 67.177 35.397 1.00 0.00 C

ATOM 1362 CG2 ILE 170 20.158 64.925 34.853 1.00 0.00 C

ATOM 1363 CD1 ILE 170 20.134 67.812 36.246 1.00 0.00 C

ATOM 1364 N ILE 171 17.855 62.861 34.033 1.00 0.00 N

ATOM 1365 CA ILE 171 17.824 61.439 34.197 1.00 0.00 C

ATOM 1366 C ILE 171 19.015 60.843 33.519 1.00 0.00 C

ATOM 1367 O ILE 171 19.429 61.285 32.448 1.00 0.00 O

ATOM 1368 CB ILE 171 16.597 60.794 33.618 1.00 0.00 C

ATOM 1369 CG1 ILE 171 15.338 61.325 34.326 1.00 0.00 C

ATOM 1370 CG2 ILE 171 16.761 59.270 33.728 1.00 0.00 C

ATOM 1371 CD1 ILE 171 14.033 60.916 33.644 1.00 0.00 C

ATOM 1372 N HIS 172 19.609 59.813 34.150 1.00 0.00 N

ATOM 1373 CA HIS 172 20.744 59.153 33.575 1.00 0.00 C

ATOM 1374 C HIS 172 20.301 57.763 33.232 1.00 0.00 C

ATOM 1375 O HIS 172 19.446 57.190 33.905 1.00 0.00 O

ATOM 1376 CB HIS 172 21.932 59.022 34.547 1.00 0.00 C

ATOM 1377 CG HIS 172 22.489 60.341 35.004 1.00 0.00 C

ATOM 1378 CD2 HIS 172 22.118 61.129 36.052 1.00 0.00 C

ATOM 1379 ND1 HIS 172 23.531 61.001 34.391 1.00 0.00 N

ATOM 1380 CE1 HIS 172 23.738 62.145 35.092 1.00 0.00 C

ATOM 1381 NE2 HIS 172 22.904 62.266 36.109 1.00 0.00 N

ATOM 1382 N PRO 173 20.852 57.222 32.177 1.00 0.00 N

ATOM 1383 CA PRO 173 20.547 55.865 31.827 1.00 0.00 C

ATOM 1384 C PRO 173 20.908 54.935 32.923 1.00 0.00 C

ATOM 1385 O PRO 173 20.321 53.857 32.993 1.00 0.00 O

ATOM 1386 CB PRO 173 21.187 55.636 30.469 1.00 0.00 C

ATOM 1387 CG PRO 173 21.002 57.011 29.801 1.00 0.00 C

ATOM 1388 CD PRO 173 20.959 58.013 30.970 1.00 0.00 C

ATOM 1389 N GLN 174 21.883 55.324 33.761 1.00 0.00 N

ATOM 1390 CA GLN 174 22.306 54.513 34.859 1.00 0.00 C

ATOM 1391 C GLN 174 21.263 54.443 35.942 1.00 0.00 C

ATOM 1392 O GLN 174 20.916 53.370 36.433 1.00 0.00 O

ATOM 1393 CB GLN 174 23.613 55.039 35.459 1.00 0.00 C

ATOM 1394 CG GLN 174 24.732 55.088 34.418 1.00 0.00 C

ATOM 1395 CD GLN 174 25.096 53.661 34.039 1.00 0.00 C

ATOM 1396 NE2 GLN 174 25.561 53.472 32.775 1.00 0.00 N

ATOM 1397 OE1 GLN 174 24.966 52.739 34.842 1.00 0.00 O

ATOM 1398 N HIS 175 20.759 55.621 36.336 1.00 0.00 N

ATOM 1399 CA HIS 175 19.828 55.880 37.402 1.00 0.00 C

ATOM 1400 C HIS 175 18.401 55.615 37.043 1.00 0.00 C

ATOM 1401 O HIS 175 17.552 55.541 37.928 1.00 0.00 O

ATOM 1402 CB HIS 175 19.907 57.337 37.894 1.00 0.00 C

ATOM 1403 CG HIS 175 19.024 57.609 39.074 1.00 0.00 C

ATOM 1404 CD2 HIS 175 17.804 58.208 39.136 1.00 0.00 C

ATOM 1405 ND1 HIS 175 19.339 57.264 40.369 1.00 0.00 N

ATOM 1406 CE1 HIS 175 18.301 57.667 41.145 1.00 0.00 C

ATOM 1407 NE2 HIS 175 17.346 58.246 40.441 1.00 0.00 N

ATOM 1408 N TRP 176 18.107 55.513 35.738 1.00 0.00 N

ATOM 1409 CA TRP 176 16.791 55.535 35.163 1.00 0.00 C

ATOM 1410 C TRP 176 15.739 54.877 36.002 1.00 0.00 C

ATOM 1411 O TRP 176 15.805 53.697 36.338 1.00 0.00 O

ATOM 1412 CB TRP 176 16.773 54.861 33.778 1.00 0.00 C

ATOM 1413 CG TRP 176 15.491 55.024 33.001 1.00 0.00 C

ATOM 1414 CD1 TRP 176 14.377 54.239 32.967 1.00 0.00 C

ATOM 1415 CD2 TRP 176 15.251 56.116 32.102 1.00 0.00 C

ATOM 1416 CE2 TRP 176 13.976 55.938 31.570 1.00 0.00 C

ATOM 1417 CE3 TRP 176 16.033 57.179 31.749 1.00 0.00 C

ATOM 1418 NE1 TRP 176 13.447 54.785 32.113 1.00 0.00 N

ATOM 1419 CZ2 TRP 176 13.459 56.828 30.672 1.00 0.00 C

ATOM 1420 CZ3 TRP 176 15.510 58.075 30.843 1.00 0.00 C

ATOM 1421 CH2 TRP 176 14.247 57.902 30.316 1.00 0.00 C

ATOM 1422 N PRO 177 14.781 55.671 36.412 1.00 0.00 N

ATOM 1423 CA PRO 177 13.591 55.113 36.987 1.00 0.00 C

ATOM 1424 C PRO 177 12.831 54.785 35.741 1.00 0.00 C

ATOM 1425 O PRO 177 12.899 55.602 34.827 1.00 0.00 O

ATOM 1426 CB PRO 177 12.963 56.223 37.833 1.00 0.00 C

ATOM 1427 CG PRO 177 13.674 57.510 37.371 1.00 0.00 C

ATOM 1428 CD PRO 177 15.045 57.017 36.885 1.00 0.00 C

ATOM 1429 N GLU 178 12.107 53.650 35.641 1.00 0.00 N

ATOM 1430 CA GLU 178 11.401 53.455 34.409 1.00 0.00 C

ATOM 1431 C GLU 178 10.147 54.263 34.518 1.00 0.00 C

ATOM 1432 O GLU 178 9.042 53.740 34.632 1.00 0.00 O

ATOM 1433 CB GLU 178 11.092 51.968 34.117 1.00 0.00 C

ATOM 1434 CG GLU 178 10.355 51.206 35.228 1.00 0.00 C

ATOM 1435 CD GLU 178 8.893 51.020 34.844 1.00 0.00 C

ATOM 1436 OE1 GLU 178 8.522 51.429 33.711 1.00 0.00 O

ATOM 1437 OE2 GLU 178 8.128 50.467 35.679 1.00 0.00 O

ATOM 1438 N ASP 179 10.312 55.598 34.470 1.00 0.00 N

ATOM 1439 CA ASP 179 9.227 56.517 34.592 1.00 0.00 C

ATOM 1440 C ASP 179 8.738 56.807 33.212 1.00 0.00 C

ATOM 1441 O ASP 179 9.202 56.215 32.239 1.00 0.00 O

ATOM 1442 CB ASP 179 9.634 57.852 35.245 1.00 0.00 C

ATOM 1443 CG ASP 179 8.378 58.555 35.743 1.00 0.00 C

ATOM 1444 OD1 ASP 179 7.286 57.929 35.683 1.00 0.00 O

ATOM 1445 OD2 ASP 179 8.494 59.724 36.198 1.00 0.00 O

ATOM 1446 N LEU 180 7.768 57.735 33.113 1.00 0.00 N

ATOM 1447 CA LEU 180 7.178 58.114 31.866 1.00 0.00 C

ATOM 1448 C LEU 180 8.203 58.886 31.127 1.00 0.00 C

ATOM 1449 O LEU 180 8.885 59.743 31.684 1.00 0.00 O

ATOM 1450 CB LEU 180 5.916 58.988 32.056 1.00 0.00 C

ATOM 1451 CG LEU 180 5.088 59.336 30.795 1.00 0.00 C

ATOM 1452 CD1 LEU 180 3.879 60.203 31.181 1.00 0.00 C

ATOM 1453 CD2 LEU 180 5.911 60.004 29.679 1.00 0.00 C

ATOM 1454 N ASP 181 8.373 58.574 29.835 1.00 0.00 N

ATOM 1455 CA ASP 181 9.424 59.272 29.190 1.00 0.00 C

ATOM 1456 C ASP 181 9.148 60.720 29.091 1.00 0.00 C

ATOM 1457 O ASP 181 9.643 61.507 29.892 1.00 0.00 O

ATOM 1458 CB ASP 181 9.678 58.774 27.756 1.00 0.00 C

ATOM 1459 CG ASP 181 10.242 57.362 27.842 1.00 0.00 C

ATOM 1460 OD1 ASP 181 10.522 56.905 28.983 1.00 0.00 O

ATOM 1461 OD2 ASP 181 10.402 56.722 26.769 1.00 0.00 O

ATOM 1462 N TYR 182 8.289 61.135 28.135 1.00 0.00 N

ATOM 1463 CA TYR 182 8.291 62.556 27.918 1.00 0.00 C

ATOM 1464 C TYR 182 7.030 62.886 27.201 1.00 0.00 C

ATOM 1465 O TYR 182 6.960 62.741 25.986 1.00 0.00 O

ATOM 1466 CB TYR 182 9.354 63.003 26.866 1.00 0.00 C

ATOM 1467 CG TYR 182 10.551 62.117 26.999 1.00 0.00 C

ATOM 1468 CD1 TYR 182 11.470 62.288 28.004 1.00 0.00 C

ATOM 1469 CD2 TYR 182 10.712 61.057 26.136 1.00 0.00 C

ATOM 1470 CE1 TYR 182 12.540 61.432 28.128 1.00 0.00 C

ATOM 1471 CE2 TYR 182 11.780 60.198 26.255 1.00 0.00 C

ATOM 1472 CZ TYR 182 12.699 60.387 27.257 1.00 0.00 C

ATOM 1473 OH TYR 182 13.797 59.515 27.401 1.00 0.00 O

ATOM 1474 N THR 183 6.014 63.422 27.879 1.00 0.00 N

ATOM 1475 CA THR 183 4.870 63.736 27.091 1.00 0.00 C

ATOM 1476 C THR 183 4.729 65.219 27.089 1.00 0.00 C

ATOM 1477 O THR 183 4.587 65.854 28.131 1.00 0.00 O

ATOM 1478 CB THR 183 3.597 63.109 27.597 1.00 0.00 C

ATOM 1479 CG2 THR 183 3.292 63.611 29.019 1.00 0.00 C

ATOM 1480 OG1 THR 183 2.523 63.405 26.716 1.00 0.00 O

ATOM 1481 N GLY 184 4.818 65.820 25.892 1.00 0.00 N

ATOM 1482 CA GLY 184 4.639 67.233 25.793 1.00 0.00 C

ATOM 1483 C GLY 184 5.874 67.912 26.289 1.00 0.00 C

ATOM 1484 O GLY 184 5.903 69.137 26.389 1.00 0.00 O

ATOM 1485 N LYS 185 6.941 67.155 26.618 1.00 0.00 N

ATOM 1486 CA LYS 185 8.082 67.889 27.081 1.00 0.00 C

ATOM 1487 C LYS 185 9.179 67.818 26.087 1.00 0.00 C

ATOM 1488 O LYS 185 9.210 66.955 25.213 1.00 0.00 O

ATOM 1489 CB LYS 185 8.684 67.503 28.451 1.00 0.00 C

ATOM 1490 CG LYS 185 9.016 66.031 28.677 1.00 0.00 C

ATOM 1491 CD LYS 185 7.816 65.203 29.124 1.00 0.00 C

ATOM 1492 CE LYS 185 7.549 65.336 30.627 1.00 0.00 C

ATOM 1493 NZ LYS 185 6.491 64.392 31.049 1.00 0.00 N

ATOM 1494 N ARG 186 10.107 68.785 26.195 1.00 0.00 N

ATOM 1495 CA ARG 186 11.223 68.806 25.311 1.00 0.00 C

ATOM 1496 C ARG 186 12.371 68.207 26.048 1.00 0.00 C

ATOM 1497 O ARG 186 12.672 68.581 27.182 1.00 0.00 O

ATOM 1498 CB ARG 186 11.557 70.214 24.794 1.00 0.00 C

ATOM 1499 CG ARG 186 11.858 71.255 25.867 1.00 0.00 C

ATOM 1500 CD ARG 186 11.478 72.666 25.411 1.00 0.00 C

ATOM 1501 NE ARG 186 12.034 72.856 24.041 1.00 0.00 N

ATOM 1502 CZ ARG 186 11.343 73.578 23.111 1.00 0.00 C

ATOM 1503 NH1 ARG 186 10.146 74.149 23.436 1.00 0.00 N

ATOM 1504 NH2 ARG 186 11.849 73.723 21.852 1.00 0.00 N

ATOM 1505 N VAL 187 13.029 67.221 25.409 1.00 0.00 N

ATOM 1506 CA VAL 187 14.068 66.495 26.075 1.00 0.00 C

ATOM 1507 C VAL 187 15.359 66.665 25.344 1.00 0.00 C

ATOM 1508 O VAL 187 15.399 66.807 24.123 1.00 0.00 O

ATOM 1509 CB VAL 187 13.796 65.021 26.139 1.00 0.00 C

ATOM 1510 CG1 VAL 187 14.983 64.321 26.828 1.00 0.00 C

ATOM 1511 CG2 VAL 187 12.444 64.814 26.841 1.00 0.00 C

ATOM 1512 N VAL 188 16.466 66.674 26.111 1.00 0.00 N

ATOM 1513 CA VAL 188 17.767 66.781 25.528 1.00 0.00 C

ATOM 1514 C VAL 188 18.516 65.548 25.915 1.00 0.00 C

ATOM 1515 O VAL 188 18.443 65.097 27.058 1.00 0.00 O

ATOM 1516 CB VAL 188 18.547 67.955 26.033 1.00 0.00 C

ATOM 1517 CG1 VAL 188 18.762 67.780 27.543 1.00 0.00 C

ATOM 1518 CG2 VAL 188 19.857 68.050 25.240 1.00 0.00 C

ATOM 1519 N VAL 189 19.240 64.945 24.954 1.00 0.00 N

ATOM 1520 CA VAL 189 19.986 63.779 25.312 1.00 0.00 C

ATOM 1521 C VAL 189 21.424 64.050 25.006 1.00 0.00 C

ATOM 1522 O VAL 189 21.777 64.511 23.921 1.00 0.00 O

ATOM 1523 CB VAL 189 19.586 62.530 24.571 1.00 0.00 C

ATOM 1524 CG1 VAL 189 19.945 62.667 23.085 1.00 0.00 C

ATOM 1525 CG2 VAL 189 20.284 61.340 25.237 1.00 0.00 C

ATOM 1526 N ILE 190 22.307 63.792 25.982 1.00 0.00 N

ATOM 1527 CA ILE 190 23.694 63.953 25.688 1.00 0.00 C

ATOM 1528 C ILE 190 24.342 62.604 25.783 1.00 0.00 C

ATOM 1529 O ILE 190 24.185 61.885 26.769 1.00 0.00 O

ATOM 1530 CB ILE 190 24.404 65.019 26.495 1.00 0.00 C

ATOM 1531 CG1 ILE 190 24.275 64.796 28.003 1.00 0.00 C

ATOM 1532 CG2 ILE 190 23.897 66.395 26.037 1.00 0.00 C

ATOM 1533 CD1 ILE 190 25.236 63.750 28.544 1.00 0.00 C

ATOM 1534 N GLY 191 25.084 62.217 24.721 1.00 0.00 N

ATOM 1535 CA GLY 191 25.696 60.914 24.646 1.00 0.00 C

ATOM 1536 C GLY 191 25.066 60.209 23.484 1.00 0.00 C

ATOM 1537 O GLY 191 23.848 60.256 23.328 1.00 0.00 O

ATOM 1538 N SER 192 25.897 59.570 22.620 1.00 0.00 N

ATOM 1539 CA SER 192 25.393 58.807 21.506 1.00 0.00 C

ATOM 1540 C SER 192 26.110 57.497 21.460 1.00 0.00 C

ATOM 1541 O SER 192 26.553 57.054 20.400 1.00 0.00 O

ATOM 1542 CB SER 192 25.648 59.475 20.147 1.00 0.00 C

ATOM 1543 OG SER 192 27.042 59.617 19.920 1.00 0.00 O

ATOM 1544 N GLY 193 26.207 56.819 22.614 1.00 0.00 N

ATOM 1545 CA GLY 193 26.830 55.531 22.646 1.00 0.00 C

ATOM 1546 C GLY 193 25.715 54.547 22.493 1.00 0.00 C

ATOM 1547 O GLY 193 24.647 54.890 21.992 1.00 0.00 O

ATOM 1548 N ALA 194 25.929 53.290 22.920 1.00 0.00 N

ATOM 1549 CA ALA 194 24.909 52.292 22.758 1.00 0.00 C

ATOM 1550 C ALA 194 23.713 52.657 23.573 1.00 0.00 C

ATOM 1551 O ALA 194 22.579 52.518 23.119 1.00 0.00 O

ATOM 1552 CB ALA 194 25.362 50.898 23.221 1.00 0.00 C

ATOM 1553 N THR 195 23.928 53.133 24.812 1.00 0.00 N

ATOM 1554 CA THR 195 22.784 53.414 25.618 1.00 0.00 C

ATOM 1555 C THR 195 22.048 54.588 24.995 1.00 0.00 C

ATOM 1556 O THR 195 20.819 54.641 24.988 1.00 0.00 O

ATOM 1557 CB THR 195 23.143 53.814 27.017 1.00 0.00 C

ATOM 1558 CG2 THR 195 21.846 54.052 27.804 1.00 0.00 C

ATOM 1559 OG1 THR 195 23.903 52.788 27.636 1.00 0.00 O

ATOM 1560 N ALA 196 22.800 55.580 24.467 1.00 0.00 N

ATOM 1561 CA ALA 196 22.281 56.780 23.854 1.00 0.00 C

ATOM 1562 C ALA 196 21.422 56.421 22.657 1.00 0.00 C

ATOM 1563 O ALA 196 20.289 56.883 22.511 1.00 0.00 O

ATOM 1564 CB ALA 196 23.429 57.586 23.302 1.00 0.00 C

ATOM 1565 N VAL 197 21.939 55.554 21.767 1.00 0.00 N

ATOM 1566 CA VAL 197 21.269 55.194 20.539 1.00 0.00 C

ATOM 1567 C VAL 197 20.017 54.430 20.829 1.00 0.00 C

ATOM 1568 O VAL 197 19.049 54.514 20.077 1.00 0.00 O

ATOM 1569 CB VAL 197 22.115 54.345 19.627 1.00 0.00 C

ATOM 1570 CG1 VAL 197 23.360 55.156 19.229 1.00 0.00 C

ATOM 1571 CG2 VAL 197 22.429 53.007 20.317 1.00 0.00 C

ATOM 1572 N THR 198 20.024 53.590 21.878 1.00 0.00 N

ATOM 1573 CA THR 198 18.838 52.839 22.164 1.00 0.00 C

ATOM 1574 C THR 198 17.763 53.719 22.722 1.00 0.00 C

ATOM 1575 O THR 198 16.587 53.561 22.397 1.00 0.00 O

ATOM 1576 CB THR 198 19.072 51.729 23.130 1.00 0.00 C

ATOM 1577 CG2 THR 198 19.467 52.306 24.494 1.00 0.00 C

ATOM 1578 OG1 THR 198 17.882 50.975 23.242 1.00 0.00 O

ATOM 1579 N LEU 199 18.154 54.650 23.610 1.00 0.00 N

ATOM 1580 CA LEU 199 17.253 55.526 24.303 1.00 0.00 C

ATOM 1581 C LEU 199 16.601 56.503 23.368 1.00 0.00 C

ATOM 1582 O LEU 199 15.399 56.747 23.456 1.00 0.00 O

ATOM 1583 CB LEU 199 18.008 56.300 25.400 1.00 0.00 C

ATOM 1584 CG LEU 199 17.169 57.282 26.232 1.00 0.00 C

ATOM 1585 CD1 LEU 199 16.026 56.565 26.960 1.00 0.00 C

ATOM 1586 CD2 LEU 199 18.068 58.066 27.203 1.00 0.00 C

ATOM 1587 N ILE 200 17.368 57.055 22.409 1.00 0.00 N

ATOM 1588 CA ILE 200 16.885 58.148 21.608 1.00 0.00 C

ATOM 1589 C ILE 200 15.661 57.802 20.804 1.00 0.00 C

ATOM 1590 O ILE 200 14.708 58.578 20.823 1.00 0.00 O

ATOM 1591 CB ILE 200 17.923 58.676 20.658 1.00 0.00 C

ATOM 1592 CG1 ILE 200 19.125 59.240 21.436 1.00 0.00 C

ATOM 1593 CG2 ILE 200 17.244 59.715 19.749 1.00 0.00 C

ATOM 1594 CD1 ILE 200 20.346 59.523 20.563 1.00 0.00 C

ATOM 1595 N PRO 201 15.598 56.708 20.100 1.00 0.00 N

ATOM 1596 CA PRO 201 14.426 56.424 19.315 1.00 0.00 C

ATOM 1597 C PRO 201 13.206 56.216 20.152 1.00 0.00 C

ATOM 1598 O PRO 201 12.105 56.509 19.686 1.00 0.00 O

ATOM 1599 CB PRO 201 14.800 55.256 18.397 1.00 0.00 C

ATOM 1600 CG PRO 201 16.212 54.832 18.853 1.00 0.00 C

ATOM 1601 CD PRO 201 16.777 56.089 19.533 1.00 0.00 C

ATOM 1602 N ALA 202 13.374 55.677 21.372 1.00 0.00 N

ATOM 1603 CA ALA 202 12.266 55.470 22.252 1.00 0.00 C

ATOM 1604 C ALA 202 11.740 56.806 22.670 1.00 0.00 C

ATOM 1605 O ALA 202 10.533 57.022 22.726 1.00 0.00 O

ATOM 1606 CB ALA 202 12.657 54.708 23.529 1.00 0.00 C

ATOM 1607 N MET 203 12.651 57.751 22.964 1.00 0.00 N

ATOM 1608 CA MET 203 12.283 59.059 23.439 1.00 0.00 C

ATOM 1609 C MET 203 11.493 59.764 22.390 1.00 0.00 C

ATOM 1610 O MET 203 10.501 60.432 22.676 1.00 0.00 O

ATOM 1611 CB MET 203 13.485 59.997 23.615 1.00 0.00 C

ATOM 1612 CG MET 203 14.478 59.627 24.706 1.00 0.00 C

ATOM 1613 SD MET 203 15.853 60.808 24.820 1.00 0.00 S

ATOM 1614 CE MET 203 16.305 60.318 26.503 1.00 0.00 C

ATOM 1615 N ALA 204 11.924 59.604 21.132 1.00 0.00 N

ATOM 1616 CA ALA 204 11.414 60.364 20.032 1.00 0.00 C

ATOM 1617 C ALA 204 9.941 60.180 19.858 1.00 0.00 C

ATOM 1618 O ALA 204 9.249 61.131 19.501 1.00 0.00 O

ATOM 1619 CB ALA 204 12.081 59.996 18.696 1.00 0.00 C

ATOM 1620 N ASP 205 9.396 58.968 20.057 1.00 0.00 N

ATOM 1621 CA ASP 205 7.996 58.900 19.749 1.00 0.00 C

ATOM 1622 C ASP 205 7.181 59.747 20.694 1.00 0.00 C

ATOM 1623 O ASP 205 6.275 60.455 20.259 1.00 0.00 O

ATOM 1624 CB ASP 205 7.398 57.472 19.696 1.00 0.00 C

ATOM 1625 CG ASP 205 7.318 56.809 21.064 1.00 0.00 C

ATOM 1626 OD1 ASP 205 8.134 57.148 21.957 1.00 0.00 O

ATOM 1627 OD2 ASP 205 6.423 55.938 21.229 1.00 0.00 O

ATOM 1628 N LYS 206 7.459 59.671 22.013 1.00 0.00 N

ATOM 1629 CA LYS 206 6.710 60.356 23.040 1.00 0.00 C

ATOM 1630 C LYS 206 6.997 61.827 23.162 1.00 0.00 C

ATOM 1631 O LYS 206 6.088 62.627 23.379 1.00 0.00 O

ATOM 1632 CB LYS 206 6.940 59.739 24.430 1.00 0.00 C

ATOM 1633 CG LYS 206 5.961 60.233 25.495 1.00 0.00 C

ATOM 1634 CD LYS 206 6.006 59.413 26.786 1.00 0.00 C

ATOM 1635 CE LYS 206 5.410 58.010 26.644 1.00 0.00 C

ATOM 1636 NZ LYS 206 6.261 57.185 25.759 1.00 0.00 N

ATOM 1637 N VAL 207 8.277 62.224 23.034 1.00 0.00 N

ATOM 1638 CA VAL 207 8.700 63.559 23.353 1.00 0.00 C

ATOM 1639 C VAL 207 8.160 64.569 22.386 1.00 0.00 C

ATOM 1640 O VAL 207 7.972 64.288 21.204 1.00 0.00 O

ATOM 1641 CB VAL 207 10.199 63.643 23.413 1.00 0.00 C

ATOM 1642 CG1 VAL 207 10.781 63.167 22.075 1.00 0.00 C

ATOM 1643 CG2 VAL 207 10.607 65.062 23.808 1.00 0.00 C

ATOM 1644 N ALA 208 7.833 65.777 22.903 1.00 0.00 N

ATOM 1645 CA ALA 208 7.353 66.844 22.072 1.00 0.00 C

ATOM 1646 C ALA 208 8.463 67.239 21.149 1.00 0.00 C

ATOM 1647 O ALA 208 8.254 67.400 19.947 1.00 0.00 O

ATOM 1648 CB ALA 208 6.954 68.093 22.876 1.00 0.00 C

ATOM 1649 N SER 209 9.686 67.401 21.696 1.00 0.00 N

ATOM 1650 CA SER 209 10.813 67.744 20.875 1.00 0.00 C

ATOM 1651 C SER 209 12.026 67.124 21.497 1.00 0.00 C

ATOM 1652 O SER 209 12.136 67.057 22.719 1.00 0.00 O

ATOM 1653 CB SER 209 11.071 69.257 20.777 1.00 0.00 C

ATOM 1654 OG SER 209 11.435 69.775 22.047 1.00 0.00 O

ATOM 1655 N ILE 210 12.975 66.640 20.668 1.00 0.00 N

ATOM 1656 CA ILE 210 14.143 66.027 21.234 1.00 0.00 C

ATOM 1657 C ILE 210 15.368 66.596 20.593 1.00 0.00 C

ATOM 1658 O ILE 210 15.449 66.726 19.371 1.00 0.00 O

ATOM 1659 CB ILE 210 14.192 64.536 21.056 1.00 0.00 C

ATOM 1660 CG1 ILE 210 15.326 63.933 21.903 1.00 0.00 C

ATOM 1661 CG2 ILE 210 14.311 64.235 19.553 1.00 0.00 C

ATOM 1662 CD1 ILE 210 15.097 64.053 23.409 1.00 0.00 C

ATOM 1663 N THR 211 16.366 66.956 21.423 1.00 0.00 N

ATOM 1664 CA THR 211 17.588 67.455 20.870 1.00 0.00 C

ATOM 1665 C THR 211 18.674 66.523 21.307 1.00 0.00 C

ATOM 1666 O THR 211 18.754 66.130 22.470 1.00 0.00 O

ATOM 1667 CB THR 211 17.934 68.853 21.300 1.00 0.00 C

ATOM 1668 CG2 THR 211 18.457 68.839 22.745 1.00 0.00 C

ATOM 1669 OG1 THR 211 18.923 69.388 20.437 1.00 0.00 O

ATOM 1670 N MET 212 19.541 66.117 20.363 1.00 0.00 N

ATOM 1671 CA MET 212 20.566 65.185 20.714 1.00 0.00 C

ATOM 1672 C MET 212 21.876 65.897 20.584 1.00 0.00 C

ATOM 1673 O MET 212 22.189 66.434 19.524 1.00 0.00 O

ATOM 1674 CB MET 212 20.552 63.958 19.784 1.00 0.00 C

ATOM 1675 CG MET 212 21.607 62.893 20.075 1.00 0.00 C

ATOM 1676 SD MET 212 23.289 63.361 19.594 1.00 0.00 S

ATOM 1677 CE MET 212 23.834 61.648 19.356 1.00 0.00 C

ATOM 1678 N LEU 213 22.668 65.928 21.679 1.00 0.00 N

ATOM 1679 CA LEU 213 23.967 66.545 21.659 1.00 0.00 C

ATOM 1680 C LEU 213 24.940 65.456 21.346 1.00 0.00 C

ATOM 1681 O LEU 213 25.126 64.529 22.136 1.00 0.00 O

ATOM 1682 CB LEU 213 24.391 67.185 22.990 1.00 0.00 C

ATOM 1683 CG LEU 213 23.625 68.481 23.302 1.00 0.00 C

ATOM 1684 CD1 LEU 213 22.114 68.226 23.357 1.00 0.00 C

ATOM 1685 CD2 LEU 213 24.166 69.163 24.569 1.00 0.00 C

ATOM 1686 N GLN 214 25.601 65.549 20.172 1.00 0.00 N

ATOM 1687 CA GLN 214 26.445 64.451 19.803 1.00 0.00 C

ATOM 1688 C GLN 214 27.877 64.880 19.700 1.00 0.00 C

ATOM 1689 O GLN 214 28.228 65.688 18.841 1.00 0.00 O

ATOM 1690 CB GLN 214 26.082 63.856 18.433 1.00 0.00 C

ATOM 1691 CG GLN 214 26.888 62.615 18.046 1.00 0.00 C

ATOM 1692 CD GLN 214 26.396 62.173 16.675 1.00 0.00 C

ATOM 1693 NE2 GLN 214 25.959 60.890 16.563 1.00 0.00 N

ATOM 1694 OE1 GLN 214 26.389 62.955 15.727 1.00 0.00 O

ATOM 1695 N ARG 215 28.736 64.390 20.622 1.00 0.00 N

ATOM 1696 CA ARG 215 30.140 64.660 20.507 1.00 0.00 C

ATOM 1697 C ARG 215 30.716 63.850 19.378 1.00 0.00 C

ATOM 1698 O ARG 215 31.279 64.401 18.433 1.00 0.00 O

ATOM 1699 CB ARG 215 30.929 64.265 21.766 1.00 0.00 C

ATOM 1700 CG ARG 215 30.611 65.143 22.973 1.00 0.00 C

ATOM 1701 CD ARG 215 31.126 66.573 22.806 1.00 0.00 C

ATOM 1702 NE ARG 215 30.697 67.353 23.998 1.00 0.00 N

ATOM 1703 CZ ARG 215 31.458 67.340 25.131 1.00 0.00 C

ATOM 1704 NH1 ARG 215 32.596 66.588 25.178 1.00 0.00 N

ATOM 1705 NH2 ARG 215 31.082 68.077 26.217 1.00 0.00 N

ATOM 1706 N THR 216 30.549 62.506 19.411 1.00 0.00 N

ATOM 1707 CA THR 216 31.148 61.757 18.345 1.00 0.00 C

ATOM 1708 C THR 216 30.129 60.815 17.760 1.00 0.00 C

ATOM 1709 O THR 216 29.358 60.184 18.480 1.00 0.00 O

ATOM 1710 CB THR 216 32.364 60.948 18.717 1.00 0.00 C

ATOM 1711 CG2 THR 216 31.920 59.559 19.197 1.00 0.00 C

ATOM 1712 OG1 THR 216 33.186 60.775 17.573 1.00 0.00 O

ATOM 1713 N PRO 217 30.086 60.719 16.454 1.00 0.00 N

ATOM 1714 CA PRO 217 29.136 59.827 15.824 1.00 0.00 C

ATOM 1715 C PRO 217 29.645 58.421 15.874 1.00 0.00 C

ATOM 1716 O PRO 217 30.860 58.236 15.926 1.00 0.00 O

ATOM 1717 CB PRO 217 28.971 60.330 14.393 1.00 0.00 C

ATOM 1718 CG PRO 217 29.354 61.817 14.472 1.00 0.00 C

ATOM 1719 CD PRO 217 30.352 61.893 15.636 1.00 0.00 C

ATOM 1720 N SER 218 28.752 57.411 15.841 1.00 0.00 N

ATOM 1721 CA SER 218 29.250 56.068 15.870 1.00 0.00 C

ATOM 1722 C SER 218 28.524 55.250 14.873 1.00 0.00 C

ATOM 1723 O SER 218 27.460 55.627 14.383 1.00 0.00 O

ATOM 1724 CB SER 218 29.069 55.377 17.231 1.00 0.00 C

ATOM 1725 OG SER 218 29.589 54.058 17.172 1.00 0.00 O

ATOM 1726 N TYR 219 29.121 54.090 14.548 1.00 0.00 N

ATOM 1727 CA TYR 219 28.521 53.193 13.616 1.00 0.00 C

ATOM 1728 C TYR 219 27.480 52.425 14.353 1.00 0.00 C

ATOM 1729 O TYR 219 27.711 51.943 15.461 1.00 0.00 O

ATOM 1730 CB TYR 219 29.489 52.156 13.017 1.00 0.00 C

ATOM 1731 CG TYR 219 30.463 52.858 12.135 1.00 0.00 C

ATOM 1732 CD1 TYR 219 31.636 53.370 12.634 1.00 0.00 C

ATOM 1733 CD2 TYR 219 30.196 53.005 10.796 1.00 0.00 C

ATOM 1734 CE1 TYR 219 32.526 54.014 11.805 1.00 0.00 C

ATOM 1735 CE2 TYR 219 31.082 53.648 9.965 1.00 0.00 C

ATOM 1736 CZ TYR 219 32.252 54.154 10.469 1.00 0.00 C

ATOM 1737 OH TYR 219 33.165 54.813 9.621 1.00 0.00 O

ATOM 1738 N ILE 220 26.274 52.350 13.766 1.00 0.00 N

ATOM 1739 CA ILE 220 25.210 51.591 14.339 1.00 0.00 C

ATOM 1740 C ILE 220 24.513 50.919 13.202 1.00 0.00 C

ATOM 1741 O ILE 220 24.338 51.494 12.130 1.00 0.00 O

ATOM 1742 CB ILE 220 24.219 52.452 15.086 1.00 0.00 C

ATOM 1743 CG1 ILE 220 23.143 51.628 15.823 1.00 0.00 C

ATOM 1744 CG2 ILE 220 23.645 53.467 14.089 1.00 0.00 C

ATOM 1745 CD1 ILE 220 22.127 50.933 14.917 1.00 0.00 C

ATOM 1746 N ILE 221 24.129 49.651 13.408 1.00 0.00 N

ATOM 1747 CA ILE 221 23.359 48.924 12.449 1.00 0.00 C

ATOM 1748 C ILE 221 22.155 48.506 13.236 1.00 0.00 C

ATOM 1749 O ILE 221 22.307 48.240 14.422 1.00 0.00 O

ATOM 1750 CB ILE 221 24.131 47.785 11.845 1.00 0.00 C

ATOM 1751 CG1 ILE 221 24.639 46.807 12.910 1.00 0.00 C

ATOM 1752 CG2 ILE 221 25.249 48.385 10.985 1.00 0.00 C

ATOM 1753 CD1 ILE 221 25.774 47.394 13.742 1.00 0.00 C

ATOM 1754 N ASN 222 20.932 48.494 12.644 1.00 0.00 N

ATOM 1755 CA ASN 222 19.771 48.361 13.496 1.00 0.00 C

ATOM 1756 C ASN 222 19.803 47.126 14.344 1.00 0.00 C

ATOM 1757 O ASN 222 20.337 47.088 15.445 1.00 0.00 O

ATOM 1758 CB ASN 222 18.447 48.435 12.698 1.00 0.00 C

ATOM 1759 CG ASN 222 18.435 47.401 11.574 1.00 0.00 C

ATOM 1760 ND2 ASN 222 19.367 47.553 10.596 1.00 0.00 N

ATOM 1761 OD1 ASN 222 17.618 46.482 11.571 1.00 0.00 O

ATOM 1762 N GLN 223 19.272 46.016 13.871 1.00 0.00 N

ATOM 1763 CA GLN 223 19.638 44.836 14.570 1.00 0.00 C

ATOM 1764 C GLN 223 20.060 44.058 13.368 1.00 0.00 C

ATOM 1765 O GLN 223 19.173 43.528 12.711 1.00 0.00 O

ATOM 1766 CB GLN 223 18.455 44.177 15.293 1.00 0.00 C

ATOM 1767 CG GLN 223 17.906 45.091 16.395 1.00 0.00 C

ATOM 1768 CD GLN 223 16.737 44.414 17.093 1.00 0.00 C

ATOM 1769 NE2 GLN 223 16.226 45.057 18.176 1.00 0.00 N

ATOM 1770 OE1 GLN 223 16.288 43.343 16.690 1.00 0.00 O

ATOM 1771 N PRO 224 21.346 44.094 13.002 1.00 0.00 N

ATOM 1772 CA PRO 224 21.797 43.503 11.745 1.00 0.00 C

ATOM 1773 C PRO 224 22.879 42.416 11.662 1.00 0.00 C

ATOM 1774 O PRO 224 22.762 41.328 12.206 1.00 0.00 O

ATOM 1775 CB PRO 224 22.184 44.673 10.845 1.00 0.00 C

ATOM 1776 CG PRO 224 21.818 45.908 11.661 1.00 0.00 C

ATOM 1777 CD PRO 224 21.953 45.405 13.096 1.00 0.00 C

ATOM 1778 N ALA 225 23.891 42.630 10.777 1.00 0.00 N

ATOM 1779 CA ALA 225 25.027 41.735 10.562 1.00 0.00 C

ATOM 1780 C ALA 225 25.824 42.199 9.263 1.00 0.00 C

ATOM 1781 O ALA 225 25.260 43.066 8.601 1.00 0.00 O

ATOM 1782 CB ALA 225 24.597 40.264 10.421 1.00 0.00 C

ATOM 1783 N ASN 226 27.106 41.693 8.874 1.00 0.00 N

ATOM 1784 CA ASN 226 27.992 42.070 7.710 1.00 0.00 C

ATOM 1785 C ASN 226 28.867 40.907 7.196 1.00 0.00 C

ATOM 1786 O ASN 226 29.601 40.300 7.966 1.00 0.00 O

ATOM 1787 CB ASN 226 28.947 43.243 7.999 1.00 0.00 C

ATOM 1788 CG ASN 226 29.271 43.974 6.704 1.00 0.00 C

ATOM 1789 ND2 ASN 226 29.834 43.251 5.701 1.00 0.00 N

ATOM 1790 OD1 ASN 226 29.024 45.173 6.587 1.00 0.00 O

ATOM 1791 N ASP 227 28.955 40.656 5.853 1.00 0.00 N

ATOM 1792 CA ASP 227 29.426 39.374 5.319 1.00 0.00 C

ATOM 1793 C ASP 227 30.713 39.294 4.536 1.00 0.00 C

ATOM 1794 O ASP 227 31.161 40.266 3.940 1.00 0.00 O

ATOM 1795 CB ASP 227 28.307 38.762 4.440 1.00 0.00 C

ATOM 1796 CG ASP 227 28.679 37.416 3.828 1.00 0.00 C

ATOM 1797 OD1 ASP 227 29.454 37.399 2.834 1.00 0.00 O

ATOM 1798 OD2 ASP 227 28.170 36.384 4.340 1.00 0.00 O

ATOM 1799 N GLY 228 31.328 38.073 4.532 1.00 0.00 N

ATOM 1800 CA GLY 228 32.539 37.741 3.818 1.00 0.00 C

ATOM 1801 C GLY 228 32.199 36.774 2.719 1.00 0.00 C

ATOM 1802 O GLY 228 31.548 35.757 2.953 1.00 0.00 O

ATOM 1803 N VAL 229 32.664 37.076 1.485 1.00 0.00 N

ATOM 1804 CA VAL 229 32.385 36.276 0.322 1.00 0.00 C

ATOM 1805 C VAL 229 32.835 37.096 -0.857 1.00 0.00 C

ATOM 1806 O VAL 229 33.726 37.934 -0.728 1.00 0.00 O

ATOM 1807 CB VAL 229 30.919 35.965 0.184 1.00 0.00 C

ATOM 1808 CG1 VAL 229 30.166 37.258 -0.164 1.00 0.00 C

ATOM 1809 CG2 VAL 229 30.732 34.820 -0.821 1.00 0.00 C

ATOM 1810 N ALA 230 32.253 36.849 -2.051 1.00 0.00 N

ATOM 1811 CA ALA 230 32.578 37.589 -3.243 1.00 0.00 C

ATOM 1812 C ALA 230 31.998 38.964 -3.097 1.00 0.00 C

ATOM 1813 O ALA 230 31.070 39.173 -2.320 1.00 0.00 O

ATOM 1814 CB ALA 230 32.003 36.971 -4.530 1.00 0.00 C

ATOM 1815 N ALA 231 32.553 39.944 -3.840 1.00 0.00 N

ATOM 1816 CA ALA 231 32.154 41.324 -3.753 1.00 0.00 C

ATOM 1817 C ALA 231 30.725 41.515 -4.165 1.00 0.00 C

ATOM 1818 O ALA 231 29.987 42.262 -3.524 1.00 0.00 O

ATOM 1819 CB ALA 231 33.013 42.248 -4.631 1.00 0.00 C

ATOM 1820 N PHE 232 30.290 40.856 -5.252 1.00 0.00 N

ATOM 1821 CA PHE 232 28.939 41.041 -5.702 1.00 0.00 C

ATOM 1822 C PHE 232 28.050 40.504 -4.638 1.00 0.00 C

ATOM 1823 O PHE 232 27.036 41.102 -4.278 1.00 0.00 O

ATOM 1824 CB PHE 232 28.625 40.276 -6.998 1.00 0.00 C

ATOM 1825 CG PHE 232 27.205 40.565 -7.350 1.00 0.00 C

ATOM 1826 CD1 PHE 232 26.880 41.684 -8.083 1.00 0.00 C

ATOM 1827 CD2 PHE 232 26.197 39.719 -6.948 1.00 0.00 C

ATOM 1828 CE1 PHE 232 25.572 41.955 -8.411 1.00 0.00 C

ATOM 1829 CE2 PHE 232 24.888 39.986 -7.273 1.00 0.00 C

ATOM 1830 CZ PHE 232 24.572 41.105 -8.005 1.00 0.00 C

ATOM 1831 N LEU 233 28.442 39.347 -4.088 1.00 0.00 N

ATOM 1832 CA LEU 233 27.669 38.728 -3.066 1.00 0.00 C

ATOM 1833 C LEU 233 27.649 39.685 -1.926 1.00 0.00 C

ATOM 1834 O LEU 233 26.637 39.808 -1.245 1.00 0.00 O

ATOM 1835 CB LEU 233 28.277 37.406 -2.571 1.00 0.00 C

ATOM 1836 CG LEU 233 28.322 36.319 -3.660 1.00 0.00 C

ATOM 1837 CD1 LEU 233 28.889 34.998 -3.117 1.00 0.00 C

ATOM 1838 CD2 LEU 233 26.950 36.150 -4.331 1.00 0.00 C

ATOM 1839 N ARG 234 28.754 40.432 -1.738 1.00 0.00 N

ATOM 1840 CA ARG 234 28.955 41.325 -0.630 1.00 0.00 C

ATOM 1841 C ARG 234 27.866 42.349 -0.575 1.00 0.00 C

ATOM 1842 O ARG 234 27.488 42.795 0.501 1.00 0.00 O

ATOM 1843 CB ARG 234 30.304 42.061 -0.690 1.00 0.00 C

ATOM 1844 CG ARG 234 31.485 41.126 -0.438 1.00 0.00 C

ATOM 1845 CD ARG 234 31.799 40.926 1.046 1.00 0.00 C

ATOM 1846 NE ARG 234 32.596 42.100 1.489 1.00 0.00 N

ATOM 1847 CZ ARG 234 33.949 42.076 1.313 1.00 0.00 C

ATOM 1848 NH1 ARG 234 34.539 40.980 0.755 1.00 0.00 N

ATOM 1849 NH2 ARG 234 34.708 43.147 1.687 1.00 0.00 N

ATOM 1850 N LYS 235 27.343 42.833 -1.704 1.00 0.00 N

ATOM 1851 CA LYS 235 26.268 43.755 -1.488 1.00 0.00 C

ATOM 1852 C LYS 235 25.130 43.009 -0.846 1.00 0.00 C

ATOM 1853 O LYS 235 24.575 43.442 0.163 1.00 0.00 O

ATOM 1854 CB LYS 235 25.746 44.369 -2.797 1.00 0.00 C

ATOM 1855 CG LYS 235 24.680 45.446 -2.584 1.00 0.00 C

ATOM 1856 CD LYS 235 24.393 46.276 -3.837 1.00 0.00 C

ATOM 1857 CE LYS 235 25.546 47.199 -4.234 1.00 0.00 C

ATOM 1858 NZ LYS 235 26.661 46.403 -4.794 1.00 0.00 N

ATOM 1859 N VAL 236 24.793 41.828 -1.397 1.00 0.00 N

ATOM 1860 CA VAL 236 23.669 41.068 -0.927 1.00 0.00 C

ATOM 1861 C VAL 236 23.857 40.565 0.480 1.00 0.00 C

ATOM 1862 O VAL 236 22.967 40.730 1.311 1.00 0.00 O

ATOM 1863 CB VAL 236 23.381 39.876 -1.788 1.00 0.00 C

ATOM 1864 CG1 VAL 236 22.251 39.062 -1.135 1.00 0.00 C

ATOM 1865 CG2 VAL 236 23.050 40.370 -3.207 1.00 0.00 C

ATOM 1866 N LEU 237 25.012 39.941 0.793 1.00 0.00 N

ATOM 1867 CA LEU 237 25.201 39.314 2.076 1.00 0.00 C

ATOM 1868 C LEU 237 25.213 40.311 3.201 1.00 0.00 C

ATOM 1869 O LEU 237 24.578 40.067 4.224 1.00 0.00 O

ATOM 1870 CB LEU 237 26.472 38.441 2.152 1.00 0.00 C

ATOM 1871 CG LEU 237 26.392 37.120 1.362 1.00 0.00 C

ATOM 1872 CD1 LEU 237 25.383 36.152 1.999 1.00 0.00 C

ATOM 1873 CD2 LEU 237 26.120 37.358 -0.130 1.00 0.00 C

ATOM 1874 N PRO 238 25.919 41.403 3.110 1.00 0.00 N

ATOM 1875 CA PRO 238 25.767 42.353 4.171 1.00 0.00 C

ATOM 1876 C PRO 238 24.385 42.889 4.278 1.00 0.00 C

ATOM 1877 O PRO 238 23.982 43.242 5.386 1.00 0.00 O

ATOM 1878 CB PRO 238 26.903 43.347 3.995 1.00 0.00 C

ATOM 1879 CG PRO 238 28.043 42.434 3.504 1.00 0.00 C

ATOM 1880 CD PRO 238 27.332 41.256 2.807 1.00 0.00 C

ATOM 1881 N ALA 239 23.647 42.963 3.157 1.00 0.00 N

ATOM 1882 CA ALA 239 22.291 43.414 3.224 1.00 0.00 C

ATOM 1883 C ALA 239 21.513 42.415 4.028 1.00 0.00 C

ATOM 1884 O ALA 239 20.736 42.784 4.906 1.00 0.00 O

ATOM 1885 CB ALA 239 21.626 43.519 1.841 1.00 0.00 C

ATOM 1886 N GLN 240 21.730 41.111 3.760 1.00 0.00 N

ATOM 1887 CA GLN 240 21.003 40.059 4.420 1.00 0.00 C

ATOM 1888 C GLN 240 21.362 40.056 5.866 1.00 0.00 C

ATOM 1889 O GLN 240 20.510 39.899 6.740 1.00 0.00 O

ATOM 1890 CB GLN 240 21.338 38.663 3.865 1.00 0.00 C

ATOM 1891 CG GLN 240 20.908 38.467 2.410 1.00 0.00 C

ATOM 1892 CD GLN 240 21.292 37.056 1.989 1.00 0.00 C

ATOM 1893 NE2 GLN 240 21.834 36.260 2.949 1.00 0.00 N

ATOM 1894 OE1 GLN 240 21.118 36.672 0.833 1.00 0.00 O

ATOM 1895 N THR 241 22.656 40.235 6.152 1.00 0.00 N

ATOM 1896 CA THR 241 23.123 40.237 7.493 1.00 0.00 C

ATOM 1897 C THR 241 22.491 41.418 8.175 1.00 0.00 C

ATOM 1898 O THR 241 22.057 41.303 9.319 1.00 0.00 O

ATOM 1899 CB THR 241 24.618 40.232 7.481 1.00 0.00 C

ATOM 1900 CG2 THR 241 25.096 38.907 6.861 1.00 0.00 C

ATOM 1901 OG1 THR 241 25.105 41.324 6.717 1.00 0.00 O

ATOM 1902 N ALA 242 22.348 42.559 7.462 1.00 0.00 N

ATOM 1903 CA ALA 242 21.741 43.748 8.003 1.00 0.00 C

ATOM 1904 C ALA 242 20.339 43.407 8.425 1.00 0.00 C

ATOM 1905 O ALA 242 19.865 43.849 9.470 1.00 0.00 O

ATOM 1906 CB ALA 242 21.649 44.893 6.980 1.00 0.00 C

ATOM 1907 N TYR 243 19.623 42.624 7.604 1.00 0.00 N

ATOM 1908 CA TYR 243 18.298 42.174 7.929 1.00 0.00 C

ATOM 1909 C TYR 243 18.364 41.197 9.068 1.00 0.00 C

ATOM 1910 O TYR 243 17.444 41.129 9.882 1.00 0.00 O

ATOM 1911 CB TYR 243 17.561 41.484 6.764 1.00 0.00 C

ATOM 1912 CG TYR 243 17.151 42.528 5.780 1.00 0.00 C

ATOM 1913 CD1 TYR 243 18.001 42.937 4.779 1.00 0.00 C

ATOM 1914 CD2 TYR 243 15.903 43.102 5.862 1.00 0.00 C

ATOM 1915 CE1 TYR 243 17.612 43.899 3.876 1.00 0.00 C

ATOM 1916 CE2 TYR 243 15.506 44.065 4.964 1.00 0.00 C

ATOM 1917 CZ TYR 243 16.363 44.465 3.967 1.00 0.00 C

ATOM 1918 OH TYR 243 15.962 45.452 3.042 1.00 0.00 O

ATOM 1919 N SER 244 19.451 40.400 9.133 1.00 0.00 N

ATOM 1920 CA SER 244 19.615 39.323 10.076 1.00 0.00 C

ATOM 1921 C SER 244 19.356 39.763 11.484 1.00 0.00 C

ATOM 1922 O SER 244 18.337 39.346 12.024 1.00 0.00 O

ATOM 1923 CB SER 244 21.033 38.725 10.049 1.00 0.00 C

ATOM 1924 OG SER 244 21.138 37.672 10.995 1.00 0.00 O

ATOM 1925 N LEU 245 20.272 40.551 12.114 1.00 0.00 N

ATOM 1926 CA LEU 245 20.124 41.136 13.442 1.00 0.00 C

ATOM 1927 C LEU 245 21.297 41.004 14.392 1.00 0.00 C

ATOM 1928 O LEU 245 21.717 39.897 14.709 1.00 0.00 O

ATOM 1929 CB LEU 245 18.849 40.791 14.256 1.00 0.00 C

ATOM 1930 CG LEU 245 17.599 41.676 13.995 1.00 0.00 C

ATOM 1931 CD1 LEU 245 17.123 41.725 12.536 1.00 0.00 C

ATOM 1932 CD2 LEU 245 16.457 41.255 14.925 1.00 0.00 C

ATOM 1933 N THR 246 21.911 42.177 14.745 1.00 0.00 N

ATOM 1934 CA THR 246 22.966 42.443 15.722 1.00 0.00 C

ATOM 1935 C THR 246 24.149 43.065 15.021 1.00 0.00 C

ATOM 1936 O THR 246 24.463 42.738 13.877 1.00 0.00 O

ATOM 1937 CB THR 246 23.427 41.269 16.545 1.00 0.00 C

ATOM 1938 CG2 THR 246 22.214 40.682 17.288 1.00 0.00 C

ATOM 1939 OG1 THR 246 24.044 40.283 15.736 1.00 0.00 O

ATOM 1940 N ARG 247 24.815 44.023 15.701 1.00 0.00 N

ATOM 1941 CA ARG 247 25.995 44.698 15.225 1.00 0.00 C

ATOM 1942 C ARG 247 27.134 43.749 15.262 1.00 0.00 C

ATOM 1943 O ARG 247 28.034 43.791 14.425 1.00 0.00 O

ATOM 1944 CB ARG 247 26.406 45.846 16.151 1.00 0.00 C

ATOM 1945 CG ARG 247 26.706 45.407 17.582 1.00 0.00 C

ATOM 1946 CD ARG 247 25.761 46.048 18.598 1.00 0.00 C

ATOM 1947 NE ARG 247 26.373 45.914 19.950 1.00 0.00 N

ATOM 1948 CZ ARG 247 25.807 46.550 21.016 1.00 0.00 C

ATOM 1949 NH1 ARG 247 24.669 47.284 20.845 1.00 0.00 N

ATOM 1950 NH2 ARG 247 26.379 46.454 22.252 1.00 0.00 N

ATOM 1951 N TYR 248 27.110 42.888 16.288 1.00 0.00 N

ATOM 1952 CA TYR 248 28.165 41.976 16.567 1.00 0.00 C

ATOM 1953 C TYR 248 28.246 41.050 15.378 1.00 0.00 C

ATOM 1954 O TYR 248 29.330 40.733 14.886 1.00 0.00 O

ATOM 1955 CB TYR 248 27.818 41.130 17.795 1.00 0.00 C

ATOM 1956 CG TYR 248 29.084 40.786 18.481 1.00 0.00 C

ATOM 1957 CD1 TYR 248 29.829 39.689 18.141 1.00 0.00 C

ATOM 1958 CD2 TYR 248 29.526 41.611 19.486 1.00 0.00 C

ATOM 1959 CE1 TYR 248 30.994 39.436 18.827 1.00 0.00 C

ATOM 1960 CE2 TYR 248 30.680 41.366 20.172 1.00 0.00 C

ATOM 1961 CZ TYR 248 31.415 40.268 19.837 1.00 0.00 C

ATOM 1962 OH TYR 248 32.604 40.008 20.540 1.00 0.00 O

ATOM 1963 N LYS 249 27.066 40.617 14.885 1.00 0.00 N

ATOM 1964 CA LYS 249 26.880 39.712 13.777 1.00 0.00 C

ATOM 1965 C LYS 249 27.478 40.388 12.601 1.00 0.00 C

ATOM 1966 O LYS 249 28.115 39.775 11.745 1.00 0.00 O

ATOM 1967 CB LYS 249 25.388 39.589 13.408 1.00 0.00 C

ATOM 1968 CG LYS 249 24.931 38.342 12.642 1.00 0.00 C

ATOM 1969 CD LYS 249 24.549 37.167 13.547 1.00 0.00 C

ATOM 1970 CE LYS 249 23.906 35.996 12.802 1.00 0.00 C

ATOM 1971 NZ LYS 249 23.551 34.924 13.759 1.00 0.00 N

ATOM 1972 N ASN 250 27.257 41.708 12.550 1.00 0.00 N

ATOM 1973 CA ASN 250 27.704 42.503 11.464 1.00 0.00 C

ATOM 1974 C ASN 250 29.162 42.332 11.319 1.00 0.00 C

ATOM 1975 O ASN 250 29.652 41.906 10.275 1.00 0.00 O

ATOM 1976 CB ASN 250 27.396 43.996 11.717 1.00 0.00 C

ATOM 1977 CG ASN 250 27.649 44.853 10.482 1.00 0.00 C

ATOM 1978 ND2 ASN 250 28.519 45.885 10.634 1.00 0.00 N

ATOM 1979 OD1 ASN 250 27.067 44.641 9.421 1.00 0.00 O

ATOM 1980 N ALA 251 29.904 42.661 12.377 1.00 0.00 N

ATOM 1981 CA ALA 251 31.311 42.554 12.214 1.00 0.00 C

ATOM 1982 C ALA 251 31.795 41.135 12.165 1.00 0.00 C

ATOM 1983 O ALA 251 32.541 40.753 11.267 1.00 0.00 O

ATOM 1984 CB ALA 251 32.083 43.244 13.334 1.00 0.00 C

ATOM 1985 N LYS 252 31.420 40.335 13.180 1.00 0.00 N

ATOM 1986 CA LYS 252 31.922 38.998 13.348 1.00 0.00 C

ATOM 1987 C LYS 252 31.259 37.930 12.542 1.00 0.00 C

ATOM 1988 O LYS 252 31.925 37.082 11.950 1.00 0.00 O

ATOM 1989 CB LYS 252 31.854 38.538 14.815 1.00 0.00 C

ATOM 1990 CG LYS 252 32.765 39.340 15.744 1.00 0.00 C

ATOM 1991 CD LYS 252 34.248 39.251 15.376 1.00 0.00 C

ATOM 1992 CE LYS 252 34.613 40.020 14.104 1.00 0.00 C

ATOM 1993 NZ LYS 252 36.061 39.886 13.830 1.00 0.00 N

ATOM 1994 N ILE 253 29.917 37.943 12.498 1.00 0.00 N

ATOM 1995 CA ILE 253 29.220 36.815 11.960 1.00 0.00 C

ATOM 1996 C ILE 253 29.587 36.642 10.560 1.00 0.00 C

ATOM 1997 O ILE 253 29.754 35.521 10.084 1.00 0.00 O

ATOM 1998 CB ILE 253 27.743 36.902 12.140 1.00 0.00 C

ATOM 1999 CG1 ILE 253 27.458 36.802 13.647 1.00 0.00 C

ATOM 2000 CG2 ILE 253 27.073 35.794 11.310 1.00 0.00 C

ATOM 2001 CD1 ILE 253 27.925 35.483 14.262 1.00 0.00 C

ATOM 2002 N THR 254 29.698 37.739 9.820 1.00 0.00 N

ATOM 2003 CA THR 254 30.226 37.392 8.571 1.00 0.00 C

ATOM 2004 C THR 254 31.403 38.266 8.311 1.00 0.00 C

ATOM 2005 O THR 254 31.625 39.228 9.040 1.00 0.00 O

ATOM 2006 CB THR 254 29.183 37.088 7.541 1.00 0.00 C

ATOM 2007 CG2 THR 254 28.028 38.103 7.536 1.00 0.00 C

ATOM 2008 OG1 THR 254 29.796 36.816 6.300 1.00 0.00 O

ATOM 2009 N LEU 255 32.232 37.903 7.320 1.00 0.00 N

ATOM 2010 CA LEU 255 33.539 38.477 7.153 1.00 0.00 C

ATOM 2011 C LEU 255 34.397 37.920 8.237 1.00 0.00 C

ATOM 2012 O LEU 255 34.128 38.136 9.420 1.00 0.00 O

ATOM 2013 CB LEU 255 33.744 39.992 7.359 1.00 0.00 C

ATOM 2014 CG LEU 255 33.210 40.982 6.316 1.00 0.00 C

ATOM 2015 CD1 LEU 255 33.698 40.632 4.901 1.00 0.00 C

ATOM 2016 CD2 LEU 255 31.711 41.238 6.485 1.00 0.00 C

ATOM 2017 N ALA 256 35.443 37.169 7.842 1.00 0.00 N

ATOM 2018 CA ALA 256 36.377 36.608 8.770 1.00 0.00 C

ATOM 2019 C ALA 256 37.199 37.685 9.407 1.00 0.00 C

ATOM 2020 O ALA 256 37.438 37.652 10.613 1.00 0.00 O

ATOM 2021 CB ALA 256 37.351 35.618 8.109 1.00 0.00 C

ATOM 2022 N PHE 257 37.687 38.672 8.626 1.00 0.00 N

ATOM 2023 CA PHE 257 38.506 39.618 9.319 1.00 0.00 C

ATOM 2024 C PHE 257 38.138 41.044 9.070 1.00 0.00 C

ATOM 2025 O PHE 257 38.853 41.779 8.392 1.00 0.00 O

ATOM 2026 CB PHE 257 40.020 39.397 9.106 1.00 0.00 C

ATOM 2027 CG PHE 257 40.349 39.285 7.657 1.00 0.00 C

ATOM 2028 CD1 PHE 257 40.316 38.051 7.050 1.00 0.00 C

ATOM 2029 CD2 PHE 257 40.696 40.384 6.906 1.00 0.00 C

ATOM 2030 CE1 PHE 257 40.621 37.905 5.717 1.00 0.00 C

ATOM 2031 CE2 PHE 257 41.003 40.243 5.572 1.00 0.00 C

ATOM 2032 CZ PHE 257 40.966 39.006 4.975 1.00 0.00 C

ATOM 2033 N TRP 258 37.012 41.483 9.662 1.00 0.00 N

ATOM 2034 CA TRP 258 36.669 42.874 9.673 1.00 0.00 C

ATOM 2035 C TRP 258 37.710 43.526 10.507 1.00 0.00 C

ATOM 2036 O TRP 258 38.189 44.610 10.210 1.00 0.00 O

ATOM 2037 CB TRP 258 35.402 43.216 10.474 1.00 0.00 C

ATOM 2038 CG TRP 258 34.055 43.241 9.805 1.00 0.00 C

ATOM 2039 CD1 TRP 258 33.340 42.289 9.148 1.00 0.00 C

ATOM 2040 CD2 TRP 258 33.217 44.402 9.882 1.00 0.00 C

ATOM 2041 CE2 TRP 258 32.012 44.082 9.262 1.00 0.00 C

ATOM 2042 CE3 TRP 258 33.431 45.628 10.446 1.00 0.00 C

ATOM 2043 NE1 TRP 258 32.099 42.781 8.814 1.00 0.00 N

ATOM 2044 CZ2 TRP 258 30.995 44.991 9.197 1.00 0.00 C

ATOM 2045 CZ3 TRP 258 32.413 46.550 10.358 1.00 0.00 C

ATOM 2046 CH2 TRP 258 31.219 46.235 9.745 1.00 0.00 C

ATOM 2047 N GLY 259 38.021 42.905 11.649 1.00 0.00 N

ATOM 2048 CA GLY 259 38.961 43.470 12.566 1.00 0.00 C

ATOM 2049 C GLY 259 40.372 43.391 12.062 1.00 0.00 C

ATOM 2050 O GLY 259 41.194 44.259 12.355 1.00 0.00 O

ATOM 2051 N PHE 260 40.716 42.275 11.392 1.00 0.00 N

ATOM 2052 CA PHE 260 42.063 42.034 10.956 1.00 0.00 C

ATOM 2053 C PHE 260 42.525 42.869 9.798 1.00 0.00 C

ATOM 2054 O PHE 260 43.584 43.488 9.874 1.00 0.00 O

ATOM 2055 CB PHE 260 42.306 40.564 10.574 1.00 0.00 C

ATOM 2056 CG PHE 260 42.186 39.756 11.819 1.00 0.00 C

ATOM 2057 CD1 PHE 260 40.954 39.328 12.256 1.00 0.00 C

ATOM 2058 CD2 PHE 260 43.304 39.427 12.551 1.00 0.00 C

ATOM 2059 CE1 PHE 260 40.839 38.582 13.405 1.00 0.00 C

ATOM 2060 CE2 PHE 260 43.195 38.681 13.701 1.00 0.00 C

ATOM 2061 CZ PHE 260 41.961 38.257 14.129 1.00 0.00 C

ATOM 2062 N CYS 261 41.756 42.933 8.691 1.00 0.00 N

ATOM 2063 CA CYS 261 42.315 43.593 7.536 1.00 0.00 C

ATOM 2064 C CYS 261 41.425 44.672 7.070 1.00 0.00 C

ATOM 2065 O CYS 261 40.270 44.406 6.790 1.00 0.00 O

ATOM 2066 CB CYS 261 42.488 42.679 6.315 1.00 0.00 C

ATOM 2067 SG CYS 261 43.748 41.403 6.557 1.00 0.00 S

ATOM 2068 N GLN 262 41.994 45.863 6.809 1.00 0.00 N

ATOM 2069 CA GLN 262 41.270 47.073 6.535 1.00 0.00 C

ATOM 2070 C GLN 262 40.338 46.932 5.368 1.00 0.00 C

ATOM 2071 O GLN 262 39.267 47.536 5.373 1.00 0.00 O

ATOM 2072 CB GLN 262 42.204 48.265 6.269 1.00 0.00 C

ATOM 2073 CG GLN 262 43.072 48.100 5.023 1.00 0.00 C

ATOM 2074 CD GLN 262 43.934 49.346 4.899 1.00 0.00 C

ATOM 2075 NE2 GLN 262 44.681 49.455 3.768 1.00 0.00 N

ATOM 2076 OE1 GLN 262 43.948 50.196 5.788 1.00 0.00 O

ATOM 2077 N ARG 263 40.693 46.149 4.333 1.00 0.00 N

ATOM 2078 CA ARG 263 39.828 46.070 3.188 1.00 0.00 C

ATOM 2079 C ARG 263 38.478 45.570 3.614 1.00 0.00 C

ATOM 2080 O ARG 263 37.450 46.099 3.198 1.00 0.00 O

ATOM 2081 CB ARG 263 40.361 45.123 2.101 1.00 0.00 C

ATOM 2082 CG ARG 263 41.650 45.625 1.447 1.00 0.00 C

ATOM 2083 CD ARG 263 42.143 44.741 0.299 1.00 0.00 C

ATOM 2084 NE ARG 263 41.259 44.999 -0.873 1.00 0.00 N

ATOM 2085 CZ ARG 263 41.534 46.035 -1.718 1.00 0.00 C

ATOM 2086 NH1 ARG 263 42.615 46.835 -1.488 1.00 0.00 N

ATOM 2087 NH2 ARG 263 40.727 46.272 -2.793 1.00 0.00 N

ATOM 2088 N PHE 264 38.427 44.545 4.477 1.00 0.00 N

ATOM 2089 CA PHE 264 37.152 44.039 4.892 1.00 0.00 C

ATOM 2090 C PHE 264 36.386 45.117 5.595 1.00 0.00 C

ATOM 2091 O PHE 264 35.189 45.258 5.357 1.00 0.00 O

ATOM 2092 CB PHE 264 37.258 42.805 5.803 1.00 0.00 C

ATOM 2093 CG PHE 264 37.567 41.680 4.880 1.00 0.00 C

ATOM 2094 CD1 PHE 264 38.776 41.627 4.229 1.00 0.00 C

ATOM 2095 CD2 PHE 264 36.651 40.676 4.670 1.00 0.00 C

ATOM 2096 CE1 PHE 264 39.064 40.590 3.374 1.00 0.00 C

ATOM 2097 CE2 PHE 264 36.934 39.635 3.818 1.00 0.00 C

ATOM 2098 CZ PHE 264 38.142 39.592 3.166 1.00 0.00 C

ATOM 2099 N PRO 265 37.005 45.863 6.470 1.00 0.00 N

ATOM 2100 CA PRO 265 36.333 46.954 7.077 1.00 0.00 C

ATOM 2101 C PRO 265 35.887 48.003 6.137 1.00 0.00 C

ATOM 2102 O PRO 265 34.807 48.541 6.354 1.00 0.00 O

ATOM 2103 CB PRO 265 37.260 47.500 8.159 1.00 0.00 C

ATOM 2104 CG PRO 265 38.415 46.495 8.170 1.00 0.00 C

ATOM 2105 CD PRO 265 37.788 45.260 7.501 1.00 0.00 C

ATOM 2106 N LYS 266 36.677 48.305 5.096 1.00 0.00 N

ATOM 2107 CA LYS 266 36.313 49.374 4.216 1.00 0.00 C

ATOM 2108 C LYS 266 35.020 49.023 3.562 1.00 0.00 C

ATOM 2109 O LYS 266 34.122 49.856 3.451 1.00 0.00 O

ATOM 2110 CB LYS 266 37.343 49.601 3.092 1.00 0.00 C

ATOM 2111 CG LYS 266 38.698 50.117 3.584 1.00 0.00 C

ATOM 2112 CD LYS 266 39.810 50.027 2.535 1.00 0.00 C

ATOM 2113 CE LYS 266 39.711 51.085 1.432 1.00 0.00 C

ATOM 2114 NZ LYS 266 40.181 52.394 1.937 1.00 0.00 N

ATOM 2115 N LEU 267 34.884 47.758 3.131 1.00 0.00 N

ATOM 2116 CA LEU 267 33.692 47.380 2.433 1.00 0.00 C

ATOM 2117 C LEU 267 32.518 47.517 3.333 1.00 0.00 C

ATOM 2118 O LEU 267 31.454 47.966 2.910 1.00 0.00 O

ATOM 2119 CB LEU 267 33.719 45.945 1.877 1.00 0.00 C

ATOM 2120 CG LEU 267 34.582 45.802 0.608 1.00 0.00 C

ATOM 2121 CD1 LEU 267 33.951 46.561 -0.571 1.00 0.00 C

ATOM 2122 CD2 LEU 267 36.037 46.221 0.854 1.00 0.00 C

ATOM 2123 N SER 268 32.679 47.121 4.604 1.00 0.00 N

ATOM 2124 CA SER 268 31.584 47.181 5.518 1.00 0.00 C

ATOM 2125 C SER 268 31.254 48.611 5.778 1.00 0.00 C

ATOM 2126 O SER 268 30.085 48.989 5.799 1.00 0.00 O

ATOM 2127 CB SER 268 31.923 46.532 6.868 1.00 0.00 C

ATOM 2128 OG SER 268 32.198 45.150 6.689 1.00 0.00 O

ATOM 2129 N LYS 269 32.291 49.452 5.943 1.00 0.00 N

ATOM 2130 CA LYS 269 32.115 50.833 6.290 1.00 0.00 C

ATOM 2131 C LYS 269 31.307 51.519 5.236 1.00 0.00 C

ATOM 2132 O LYS 269 30.381 52.268 5.544 1.00 0.00 O

ATOM 2133 CB LYS 269 33.464 51.569 6.386 1.00 0.00 C

ATOM 2134 CG LYS 269 33.359 53.050 6.751 1.00 0.00 C

ATOM 2135 CD LYS 269 34.715 53.696 7.047 1.00 0.00 C

ATOM 2136 CE LYS 269 34.643 55.214 7.229 1.00 0.00 C

ATOM 2137 NZ LYS 269 35.991 55.759 7.509 1.00 0.00 N

ATOM 2138 N LYS 270 31.635 51.264 3.959 1.00 0.00 N

ATOM 2139 CA LYS 270 30.972 51.925 2.874 1.00 0.00 C

ATOM 2140 C LYS 270 29.527 51.550 2.849 1.00 0.00 C

ATOM 2141 O LYS 270 28.666 52.412 2.680 1.00 0.00 O

ATOM 2142 CB LYS 270 31.560 51.551 1.504 1.00 0.00 C

ATOM 2143 CG LYS 270 33.000 52.026 1.317 1.00 0.00 C

ATOM 2144 CD LYS 270 33.698 51.413 0.102 1.00 0.00 C

ATOM 2145 CE LYS 270 35.138 51.895 -0.076 1.00 0.00 C

ATOM 2146 NZ LYS 270 35.150 53.339 -0.396 1.00 0.00 N

ATOM 2147 N LEU 271 29.221 50.254 3.046 1.00 0.00 N

ATOM 2148 CA LEU 271 27.870 49.792 2.923 1.00 0.00 C

ATOM 2149 C LEU 271 27.037 50.482 3.956 1.00 0.00 C

ATOM 2150 O LEU 271 25.918 50.901 3.667 1.00 0.00 O

ATOM 2151 CB LEU 271 27.753 48.260 3.088 1.00 0.00 C

ATOM 2152 CG LEU 271 26.373 47.641 2.761 1.00 0.00 C

ATOM 2153 CD1 LEU 271 26.444 46.110 2.842 1.00 0.00 C

ATOM 2154 CD2 LEU 271 25.236 48.188 3.641 1.00 0.00 C

ATOM 2155 N LEU 272 27.555 50.619 5.191 1.00 0.00 N

ATOM 2156 CA LEU 272 26.795 51.266 6.220 1.00 0.00 C

ATOM 2157 C LEU 272 26.577 52.708 5.881 1.00 0.00 C

ATOM 2158 O LEU 272 25.483 53.231 6.080 1.00 0.00 O

ATOM 2159 CB LEU 272 27.447 51.212 7.613 1.00 0.00 C

ATOM 2160 CG LEU 272 27.304 49.846 8.307 1.00 0.00 C

ATOM 2161 CD1 LEU 272 27.942 48.717 7.486 1.00 0.00 C

ATOM 2162 CD2 LEU 272 27.829 49.903 9.750 1.00 0.00 C

ATOM 2163 N LEU 273 27.603 53.390 5.340 1.00 0.00 N

ATOM 2164 CA LEU 273 27.485 54.792 5.059 1.00 0.00 C

ATOM 2165 C LEU 273 26.373 54.991 4.080 1.00 0.00 C

ATOM 2166 O LEU 273 25.591 55.932 4.212 1.00 0.00 O

ATOM 2167 CB LEU 273 28.763 55.393 4.443 1.00 0.00 C

ATOM 2168 CG LEU 273 29.985 55.329 5.381 1.00 0.00 C

ATOM 2169 CD1 LEU 273 31.231 55.946 4.725 1.00 0.00 C

ATOM 2170 CD2 LEU 273 29.669 55.941 6.755 1.00 0.00 C

ATOM 2171 N TRP 274 26.269 54.103 3.071 1.00 0.00 N

ATOM 2172 CA TRP 274 25.228 54.249 2.096 1.00 0.00 C

ATOM 2173 C TRP 274 23.902 54.091 2.770 1.00 0.00 C

ATOM 2174 O TRP 274 22.952 54.807 2.461 1.00 0.00 O

ATOM 2175 CB TRP 274 25.260 53.235 0.931 1.00 0.00 C

ATOM 2176 CG TRP 274 26.398 53.403 -0.050 1.00 0.00 C

ATOM 2177 CD1 TRP 274 26.590 54.389 -0.974 1.00 0.00 C

ATOM 2178 CD2 TRP 274 27.462 52.456 -0.245 1.00 0.00 C

ATOM 2179 CE2 TRP 274 28.263 52.940 -1.278 1.00 0.00 C

ATOM 2180 CE3 TRP 274 27.737 51.269 0.372 1.00 0.00 C

ATOM 2181 NE1 TRP 274 27.722 54.132 -1.710 1.00 0.00 N

ATOM 2182 CZ2 TRP 274 29.359 52.246 -1.708 1.00 0.00 C

ATOM 2183 CZ3 TRP 274 28.853 50.581 -0.051 1.00 0.00 C

ATOM 2184 CH2 TRP 274 29.649 51.060 -1.070 1.00 0.00 C

ATOM 2185 N LEU 275 23.791 53.144 3.718 1.00 0.00 N

ATOM 2186 CA LEU 275 22.521 52.927 4.344 1.00 0.00 C

ATOM 2187 C LEU 275 22.116 54.189 5.037 1.00 0.00 C

ATOM 2188 O LEU 275 20.972 54.627 4.929 1.00 0.00 O

ATOM 2189 CB LEU 275 22.560 51.816 5.408 1.00 0.00 C

ATOM 2190 CG LEU 275 22.918 50.431 4.841 1.00 0.00 C

ATOM 2191 CD1 LEU 275 22.933 49.360 5.945 1.00 0.00 C

ATOM 2192 CD2 LEU 275 22.010 50.058 3.658 1.00 0.00 C

ATOM 2193 N THR 276 23.058 54.827 5.753 1.00 0.00 N

ATOM 2194 CA THR 276 22.743 56.006 6.503 1.00 0.00 C

ATOM 2195 C THR 276 22.315 57.091 5.560 1.00 0.00 C

ATOM 2196 O THR 276 21.332 57.790 5.791 1.00 0.00 O

ATOM 2197 CB THR 276 23.920 56.501 7.289 1.00 0.00 C

ATOM 2198 CG2 THR 276 23.466 57.696 8.142 1.00 0.00 C

ATOM 2199 OG1 THR 276 24.406 55.472 8.139 1.00 0.00 O

ATOM 2200 N ARG 277 23.036 57.263 4.445 1.00 0.00 N

ATOM 2201 CA ARG 277 22.681 58.319 3.546 1.00 0.00 C

ATOM 2202 C ARG 277 21.317 58.045 2.984 1.00 0.00 C

ATOM 2203 O ARG 277 20.517 58.956 2.786 1.00 0.00 O

ATOM 2204 CB ARG 277 23.690 58.464 2.395 1.00 0.00 C

ATOM 2205 CG ARG 277 23.798 57.230 1.501 1.00 0.00 C

ATOM 2206 CD ARG 277 25.187 57.037 0.892 1.00 0.00 C

ATOM 2207 NE ARG 277 25.571 58.305 0.215 1.00 0.00 N

ATOM 2208 CZ ARG 277 26.866 58.732 0.280 1.00 0.00 C

ATOM 2209 NH1 ARG 277 27.795 57.989 0.949 1.00 0.00 N

ATOM 2210 NH2 ARG 277 27.231 59.903 -0.317 1.00 0.00 N

ATOM 2211 N LYS 278 21.004 56.779 2.672 1.00 0.00 N

ATOM 2212 CA LYS 278 19.709 56.536 2.109 1.00 0.00 C

ATOM 2213 C LYS 278 18.629 56.811 3.116 1.00 0.00 C

ATOM 2214 O LYS 278 17.706 57.580 2.853 1.00 0.00 O

ATOM 2215 CB LYS 278 19.541 55.083 1.637 1.00 0.00 C

ATOM 2216 CG LYS 278 20.489 54.712 0.494 1.00 0.00 C

ATOM 2217 CD LYS 278 20.564 53.209 0.224 1.00 0.00 C

ATOM 2218 CE LYS 278 19.327 52.655 -0.486 1.00 0.00 C

ATOM 2219 NZ LYS 278 19.481 51.201 -0.710 1.00 0.00 N

ATOM 2220 N GLU 279 18.730 56.192 4.309 1.00 0.00 N

ATOM 2221 CA GLU 279 17.700 56.287 5.307 1.00 0.00 C

ATOM 2222 C GLU 279 17.632 57.632 5.948 1.00 0.00 C

ATOM 2223 O GLU 279 16.546 58.167 6.166 1.00 0.00 O

ATOM 2224 CB GLU 279 17.846 55.257 6.443 1.00 0.00 C

ATOM 2225 CG GLU 279 17.470 53.832 6.031 1.00 0.00 C

ATOM 2226 CD GLU 279 18.655 53.208 5.313 1.00 0.00 C

ATOM 2227 OE1 GLU 279 19.609 52.779 6.016 1.00 0.00 O

ATOM 2228 OE2 GLU 279 18.624 53.152 4.055 1.00 0.00 O

ATOM 2229 N LEU 280 18.792 58.236 6.246 1.00 0.00 N

ATOM 2230 CA LEU 280 18.745 59.422 7.036 1.00 0.00 C

ATOM 2231 C LEU 280 18.527 60.619 6.186 1.00 0.00 C

ATOM 2232 O LEU 280 17.381 60.911 5.856 1.00 0.00 O

ATOM 2233 CB LEU 280 19.989 59.661 7.919 1.00 0.00 C

ATOM 2234 CG LEU 280 20.240 58.586 8.987 1.00 0.00 C

ATOM 2235 CD1 LEU 280 20.560 57.228 8.347 1.00 0.00 C

ATOM 2236 CD2 LEU 280 21.321 59.046 9.978 1.00 0.00 C

ATOM 2237 N PRO 281 19.606 61.264 5.796 1.00 0.00 N

ATOM 2238 CA PRO 281 19.535 62.600 5.266 1.00 0.00 C

ATOM 2239 C PRO 281 18.407 62.897 4.356 1.00 0.00 C

ATOM 2240 O PRO 281 18.536 62.698 3.149 1.00 0.00 O

ATOM 2241 CB PRO 281 20.826 62.831 4.495 1.00 0.00 C

ATOM 2242 CG PRO 281 21.157 61.426 3.984 1.00 0.00 C

ATOM 2243 CD PRO 281 20.658 60.518 5.119 1.00 0.00 C

ATOM 2244 N LYS 282 17.284 63.361 4.928 1.00 0.00 N

ATOM 2245 CA LYS 282 16.330 63.926 4.045 1.00 0.00 C

ATOM 2246 C LYS 282 17.137 65.084 3.613 1.00 0.00 C

ATOM 2247 O LYS 282 17.194 65.450 2.441 1.00 0.00 O

ATOM 2248 CB LYS 282 15.062 64.442 4.746 1.00 0.00 C

ATOM 2249 CG LYS 282 14.065 63.335 5.091 1.00 0.00 C

ATOM 2250 CD LYS 282 12.959 63.775 6.051 1.00 0.00 C

ATOM 2251 CE LYS 282 11.784 62.795 6.108 1.00 0.00 C

ATOM 2252 NZ LYS 282 12.285 61.407 6.210 1.00 0.00 N

ATOM 2253 N ASP 283 17.829 65.658 4.615 1.00 0.00 N

ATOM 2254 CA ASP 283 18.778 66.687 4.361 1.00 0.00 C

ATOM 2255 C ASP 283 20.110 66.042 4.555 1.00 0.00 C

ATOM 2256 O ASP 283 20.369 65.398 5.571 1.00 0.00 O

ATOM 2257 CB ASP 283 18.675 67.913 5.285 1.00 0.00 C

ATOM 2258 CG ASP 283 19.011 67.499 6.704 1.00 0.00 C

ATOM 2259 OD1 ASP 283 18.531 66.421 7.145 1.00 0.00 O

ATOM 2260 OD2 ASP 283 19.749 68.272 7.370 1.00 0.00 O

ATOM 2261 N TYR 284 20.989 66.214 3.557 1.00 0.00 N

ATOM 2262 CA TYR 284 22.275 65.587 3.512 1.00 0.00 C

ATOM 2263 C TYR 284 23.105 66.003 4.684 1.00 0.00 C

ATOM 2264 O TYR 284 23.810 65.167 5.245 1.00 0.00 O

ATOM 2265 CB TYR 284 23.057 65.943 2.236 1.00 0.00 C

ATOM 2266 CG TYR 284 22.304 65.366 1.087 1.00 0.00 C

ATOM 2267 CD1 TYR 284 21.176 65.992 0.610 1.00 0.00 C

ATOM 2268 CD2 TYR 284 22.725 64.202 0.485 1.00 0.00 C

ATOM 2269 CE1 TYR 284 20.475 65.464 -0.449 1.00 0.00 C

ATOM 2270 CE2 TYR 284 22.028 63.670 -0.574 1.00 0.00 C

ATOM 2271 CZ TYR 284 20.901 64.301 -1.043 1.00 0.00 C

ATOM 2272 OH TYR 284 20.184 63.758 -2.129 1.00 0.00 O

ATOM 2273 N PRO 285 23.078 67.239 5.094 1.00 0.00 N

ATOM 2274 CA PRO 285 23.939 67.614 6.177 1.00 0.00 C

ATOM 2275 C PRO 285 23.697 66.866 7.447 1.00 0.00 C

ATOM 2276 O PRO 285 24.652 66.663 8.194 1.00 0.00 O

ATOM 2277 CB PRO 285 23.834 69.133 6.277 1.00 0.00 C

ATOM 2278 CG PRO 285 23.566 69.557 4.819 1.00 0.00 C

ATOM 2279 CD PRO 285 22.837 68.355 4.191 1.00 0.00 C

ATOM 2280 N VAL 286 22.453 66.455 7.743 1.00 0.00 N

ATOM 2281 CA VAL 286 22.267 65.709 8.951 1.00 0.00 C

ATOM 2282 C VAL 286 22.985 64.406 8.812 1.00 0.00 C

ATOM 2283 O VAL 286 23.639 63.943 9.746 1.00 0.00 O

ATOM 2284 CB VAL 286 20.838 65.386 9.258 1.00 0.00 C

ATOM 2285 CG1 VAL 286 20.843 64.392 10.425 1.00 0.00 C

ATOM 2286 CG2 VAL 286 20.087 66.688 9.582 1.00 0.00 C

ATOM 2287 N ASP 287 22.896 63.791 7.619 1.00 0.00 N

ATOM 2288 CA ASP 287 23.483 62.502 7.393 1.00 0.00 C

ATOM 2289 C ASP 287 24.954 62.569 7.620 1.00 0.00 C

ATOM 2290 O ASP 287 25.529 61.679 8.243 1.00 0.00 O

ATOM 2291 CB ASP 287 23.359 62.030 5.945 1.00 0.00 C

ATOM 2292 CG ASP 287 24.008 60.661 5.814 1.00 0.00 C

ATOM 2293 OD1 ASP 287 24.220 60.002 6.865 1.00 0.00 O

ATOM 2294 OD2 ASP 287 24.298 60.253 4.658 1.00 0.00 O

ATOM 2295 N VAL 288 25.608 63.628 7.116 1.00 0.00 N

ATOM 2296 CA VAL 288 27.036 63.689 7.213 1.00 0.00 C

ATOM 2297 C VAL 288 27.433 63.720 8.653 1.00 0.00 C

ATOM 2298 O VAL 288 28.402 63.073 9.044 1.00 0.00 O

ATOM 2299 CB VAL 288 27.642 64.887 6.535 1.00 0.00 C

ATOM 2300 CG1 VAL 288 27.297 64.824 5.037 1.00 0.00 C

ATOM 2301 CG2 VAL 288 27.172 66.165 7.243 1.00 0.00 C

ATOM 2302 N HIS 289 26.683 64.458 9.492 1.00 0.00 N

ATOM 2303 CA HIS 289 27.041 64.567 10.878 1.00 0.00 C

ATOM 2304 C HIS 289 27.008 63.203 11.492 1.00 0.00 C

ATOM 2305 O HIS 289 27.841 62.875 12.334 1.00 0.00 O

ATOM 2306 CB HIS 289 26.089 65.459 11.696 1.00 0.00 C

ATOM 2307 CG HIS 289 26.234 66.925 11.411 1.00 0.00 C

ATOM 2308 CD2 HIS 289 27.119 67.825 11.920 1.00 0.00 C

ATOM 2309 ND1 HIS 289 25.437 67.635 10.541 1.00 0.00 N

ATOM 2310 CE1 HIS 289 25.875 68.919 10.566 1.00 0.00 C

ATOM 2311 NE2 HIS 289 26.894 69.083 11.390 1.00 0.00 N

ATOM 2312 N PHE 290 26.023 62.381 11.091 1.00 0.00 N

ATOM 2313 CA PHE 290 25.839 61.058 11.615 1.00 0.00 C

ATOM 2314 C PHE 290 26.947 60.112 11.234 1.00 0.00 C

ATOM 2315 O PHE 290 27.271 59.228 12.021 1.00 0.00 O

ATOM 2316 CB PHE 290 24.502 60.412 11.205 1.00 0.00 C

ATOM 2317 CG PHE 290 23.433 60.978 12.077 1.00 0.00 C

ATOM 2318 CD1 PHE 290 22.829 62.180 11.789 1.00 0.00 C

ATOM 2319 CD2 PHE 290 23.033 60.287 13.198 1.00 0.00 C

ATOM 2320 CE1 PHE 290 21.845 62.682 12.609 1.00 0.00 C

ATOM 2321 CE2 PHE 290 22.051 60.783 14.021 1.00 0.00 C

ATOM 2322 CZ PHE 290 21.454 61.984 13.726 1.00 0.00 C

ATOM 2323 N ASN 291 27.538 60.219 10.023 1.00 0.00 N

ATOM 2324 CA ASN 291 28.511 59.234 9.601 1.00 0.00 C

ATOM 2325 C ASN 291 29.850 59.458 10.248 1.00 0.00 C

ATOM 2326 O ASN 291 30.390 60.562 10.231 1.00 0.00 O

ATOM 2327 CB ASN 291 28.752 59.228 8.081 1.00 0.00 C

ATOM 2328 CG ASN 291 27.476 58.769 7.391 1.00 0.00 C

ATOM 2329 ND2 ASN 291 27.081 59.492 6.309 1.00 0.00 N

ATOM 2330 OD1 ASN 291 26.845 57.797 7.803 1.00 0.00 O

ATOM 2331 N PRO 292 30.383 58.418 10.856 1.00 0.00 N

ATOM 2332 CA PRO 292 31.687 58.541 11.473 1.00 0.00 C

ATOM 2333 C PRO 292 32.867 58.273 10.573 1.00 0.00 C

ATOM 2334 O PRO 292 32.772 57.361 9.752 1.00 0.00 O

ATOM 2335 CB PRO 292 31.700 57.563 12.644 1.00 0.00 C

ATOM 2336 CG PRO 292 30.219 57.340 12.976 1.00 0.00 C

ATOM 2337 CD PRO 292 29.508 57.555 11.634 1.00 0.00 C

ATOM 2338 N PRO 293 33.942 59.028 10.671 1.00 0.00 N

ATOM 2339 CA PRO 293 35.162 58.615 10.016 1.00 0.00 C

ATOM 2340 C PRO 293 35.909 57.769 11.009 1.00 0.00 C

ATOM 2341 O PRO 293 36.069 58.238 12.133 1.00 0.00 O

ATOM 2342 CB PRO 293 35.923 59.891 9.664 1.00 0.00 C

ATOM 2343 CG PRO 293 34.837 60.980 9.641 1.00 0.00 C

ATOM 2344 CD PRO 293 33.776 60.472 10.631 1.00 0.00 C

ATOM 2345 N TYR 294 36.427 56.585 10.617 1.00 0.00 N

ATOM 2346 CA TYR 294 37.182 55.692 11.468 1.00 0.00 C

ATOM 2347 C TYR 294 36.764 54.303 11.127 1.00 0.00 C

ATOM 2348 O TYR 294 35.616 54.070 10.757 1.00 0.00 O

ATOM 2349 CB TYR 294 37.099 55.874 13.008 1.00 0.00 C

ATOM 2350 CG TYR 294 35.752 55.581 13.585 1.00 0.00 C

ATOM 2351 CD1 TYR 294 35.323 54.285 13.752 1.00 0.00 C

ATOM 2352 CD2 TYR 294 34.931 56.600 14.011 1.00 0.00 C

ATOM 2353 CE1 TYR 294 34.092 54.010 14.298 1.00 0.00 C

ATOM 2354 CE2 TYR 294 33.699 56.335 14.558 1.00 0.00 C

ATOM 2355 CZ TYR 294 33.275 55.038 14.700 1.00 0.00 C

ATOM 2356 OH TYR 294 32.010 54.764 15.261 1.00 0.00 O

ATOM 2357 N ASN 295 37.706 53.344 11.198 1.00 0.00 N

ATOM 2358 CA ASN 295 37.379 51.999 10.827 1.00 0.00 C

ATOM 2359 C ASN 295 36.229 51.545 11.662 1.00 0.00 C

ATOM 2360 O ASN 295 36.138 51.830 12.854 1.00 0.00 O

ATOM 2361 CB ASN 295 38.530 50.999 11.034 1.00 0.00 C

ATOM 2362 CG ASN 295 39.615 51.303 10.011 1.00 0.00 C

ATOM 2363 ND2 ASN 295 40.886 51.392 10.486 1.00 0.00 N

ATOM 2364 OD1 ASN 295 39.340 51.461 8.823 1.00 0.00 O

ATOM 2365 N PRO 296 35.323 50.861 11.025 1.00 0.00 N

ATOM 2366 CA PRO 296 34.166 50.373 11.721 1.00 0.00 C

ATOM 2367 C PRO 296 34.495 49.158 12.532 1.00 0.00 C

ATOM 2368 O PRO 296 35.407 48.425 12.155 1.00 0.00 O

ATOM 2369 CB PRO 296 33.091 50.138 10.655 1.00 0.00 C

ATOM 2370 CG PRO 296 33.846 50.208 9.315 1.00 0.00 C

ATOM 2371 CD PRO 296 35.045 51.115 9.622 1.00 0.00 C

ATOM 2372 N TRP 297 33.768 48.931 13.646 1.00 0.00 N

ATOM 2373 CA TRP 297 33.972 47.775 14.472 1.00 0.00 C

ATOM 2374 C TRP 297 35.234 47.932 15.256 1.00 0.00 C

ATOM 2375 O TRP 297 35.529 47.124 16.138 1.00 0.00 O

ATOM 2376 CB TRP 297 34.064 46.485 13.638 1.00 0.00 C

ATOM 2377 CG TRP 297 34.525 45.269 14.403 1.00 0.00 C

ATOM 2378 CD1 TRP 297 35.735 44.641 14.370 1.00 0.00 C

ATOM 2379 CD2 TRP 297 33.731 44.573 15.372 1.00 0.00 C

ATOM 2380 CE2 TRP 297 34.511 43.530 15.872 1.00 0.00 C

ATOM 2381 CE3 TRP 297 32.457 44.788 15.810 1.00 0.00 C

ATOM 2382 NE1 TRP 297 35.738 43.582 15.246 1.00 0.00 N

ATOM 2383 CZ2 TRP 297 34.024 42.680 16.823 1.00 0.00 C

ATOM 2384 CZ3 TRP 297 31.967 43.922 16.758 1.00 0.00 C

ATOM 2385 CH2 TRP 297 32.734 42.889 17.256 1.00 0.00 C

ATOM 2386 N ASP 298 35.999 49.006 14.991 1.00 0.00 N

ATOM 2387 CA ASP 298 37.166 49.219 15.790 1.00 0.00 C

ATOM 2388 C ASP 298 36.587 49.449 17.126 1.00 0.00 C

ATOM 2389 O ASP 298 37.096 49.008 18.158 1.00 0.00 O

ATOM 2390 CB ASP 298 37.945 50.485 15.399 1.00 0.00 C

ATOM 2391 CG ASP 298 39.160 50.589 16.307 1.00 0.00 C

ATOM 2392 OD1 ASP 298 39.361 49.664 17.139 1.00 0.00 O

ATOM 2393 OD2 ASP 298 39.909 51.593 16.176 1.00 0.00 O

ATOM 2394 N GLN 299 35.457 50.164 17.099 1.00 0.00 N

ATOM 2395 CA GLN 299 34.713 50.368 18.282 1.00 0.00 C

ATOM 2396 C GLN 299 33.624 49.354 18.230 1.00 0.00 C

ATOM 2397 O GLN 299 33.258 48.894 17.151 1.00 0.00 O

ATOM 2398 CB GLN 299 34.065 51.757 18.393 1.00 0.00 C

ATOM 2399 CG GLN 299 35.080 52.892 18.523 1.00 0.00 C

ATOM 2400 CD GLN 299 34.301 54.192 18.653 1.00 0.00 C

ATOM 2401 NE2 GLN 299 35.030 55.325 18.832 1.00 0.00 N

ATOM 2402 OE1 GLN 299 33.072 54.198 18.607 1.00 0.00 O

ATOM 2403 N ARG 300 33.075 48.945 19.386 1.00 0.00 N

ATOM 2404 CA ARG 300 32.060 47.952 19.244 1.00 0.00 C

ATOM 2405 C ARG 300 30.900 48.548 18.504 1.00 0.00 C

ATOM 2406 O ARG 300 30.470 49.661 18.796 1.00 0.00 O

ATOM 2407 CB ARG 300 31.489 47.385 20.558 1.00 0.00 C

ATOM 2408 CG ARG 300 32.319 46.276 21.201 1.00 0.00 C

ATOM 2409 CD ARG 300 31.452 45.281 21.979 1.00 0.00 C

ATOM 2410 NE ARG 300 30.203 45.145 21.180 1.00 0.00 N

ATOM 2411 CZ ARG 300 30.283 44.520 19.973 1.00 0.00 C

ATOM 2412 NH1 ARG 300 31.494 44.040 19.574 1.00 0.00 N

ATOM 2413 NH2 ARG 300 29.193 44.434 19.154 1.00 0.00 N

ATOM 2414 N LEU 301 30.360 47.795 17.521 1.00 0.00 N

ATOM 2415 CA LEU 301 29.231 48.242 16.746 1.00 0.00 C

ATOM 2416 C LEU 301 28.058 48.200 17.679 1.00 0.00 C

ATOM 2417 O LEU 301 28.096 47.485 18.678 1.00 0.00 O

ATOM 2418 CB LEU 301 28.885 47.328 15.566 1.00 0.00 C

ATOM 2419 CG LEU 301 29.930 47.335 14.444 1.00 0.00 C

ATOM 2420 CD1 LEU 301 29.473 46.456 13.274 1.00 0.00 C

ATOM 2421 CD2 LEU 301 30.272 48.769 14.012 1.00 0.00 C

ATOM 2422 N CYS 302 27.000 48.996 17.408 1.00 0.00 N

ATOM 2423 CA CYS 302 25.853 48.974 18.279 1.00 0.00 C

ATOM 2424 C CYS 302 24.668 48.591 17.538 1.00 0.00 C

ATOM 2425 O CYS 302 24.547 48.780 16.328 1.00 0.00 O

ATOM 2426 CB CYS 302 25.591 50.316 18.988 1.00 0.00 C

ATOM 2427 SG CYS 302 25.456 51.715 17.840 1.00 0.00 S

ATOM 2428 N SER 303 23.762 48.019 18.326 1.00 0.00 N

ATOM 2429 CA SER 303 22.560 47.520 17.808 1.00 0.00 C

ATOM 2430 C SER 303 21.519 48.481 18.214 1.00 0.00 C

ATOM 2431 O SER 303 21.512 48.917 19.359 1.00 0.00 O

ATOM 2432 CB SER 303 22.219 46.138 18.396 1.00 0.00 C

ATOM 2433 OG SER 303 21.010 45.636 17.854 1.00 0.00 O

ATOM 2434 N VAL 304 20.655 48.911 17.278 1.00 0.00 N

ATOM 2435 CA VAL 304 19.581 49.681 17.805 1.00 0.00 C

ATOM 2436 C VAL 304 18.431 48.731 17.905 1.00 0.00 C

ATOM 2437 O VAL 304 17.944 48.212 16.895 1.00 0.00 O

ATOM 2438 CB VAL 304 19.169 50.896 17.028 1.00 0.00 C

ATOM 2439 CG1 VAL 304 17.928 51.488 17.718 1.00 0.00 C

ATOM 2440 CG2 VAL 304 20.337 51.895 17.037 1.00 0.00 C

ATOM 2441 N PRO 305 18.031 48.471 19.138 1.00 0.00 N

ATOM 2442 CA PRO 305 16.932 47.593 19.417 1.00 0.00 C

ATOM 2443 C PRO 305 15.788 48.344 18.878 1.00 0.00 C

ATOM 2444 O PRO 305 15.720 49.545 19.135 1.00 0.00 O

ATOM 2445 CB PRO 305 16.900 47.411 20.932 1.00 0.00 C

ATOM 2446 CG PRO 305 18.352 47.685 21.360 1.00 0.00 C

ATOM 2447 CD PRO 305 18.878 48.667 20.301 1.00 0.00 C

ATOM 2448 N GLU 306 14.894 47.688 18.116 1.00 0.00 N

ATOM 2449 CA GLU 306 13.868 48.408 17.421 1.00 0.00 C

ATOM 2450 C GLU 306 14.543 49.068 16.253 1.00 0.00 C

ATOM 2451 O GLU 306 14.082 48.956 15.118 1.00 0.00 O

ATOM 2452 CB GLU 306 13.134 49.474 18.259 1.00 0.00 C

ATOM 2453 CG GLU 306 11.965 50.123 17.515 1.00 0.00 C

ATOM 2454 CD GLU 306 11.318 51.149 18.435 1.00 0.00 C

ATOM 2455 OE1 GLU 306 11.837 51.340 19.567 1.00 0.00 O

ATOM 2456 OE2 GLU 306 10.296 51.755 18.017 1.00 0.00 O

ATOM 2457 N GLY 307 15.679 49.757 16.502 1.00 0.00 N

ATOM 2458 CA GLY 307 16.410 50.381 15.459 1.00 0.00 C

ATOM 2459 C GLY 307 15.756 51.686 15.199 1.00 0.00 C

ATOM 2460 O GLY 307 15.230 52.328 16.108 1.00 0.00 O

ATOM 2461 N ASP 308 15.792 52.102 13.926 1.00 0.00 N

ATOM 2462 CA ASP 308 15.201 53.333 13.513 1.00 0.00 C

ATOM 2463 C ASP 308 15.889 54.456 14.211 1.00 0.00 C

ATOM 2464 O ASP 308 15.307 55.523 14.398 1.00 0.00 O

ATOM 2465 CB ASP 308 13.703 53.428 13.845 1.00 0.00 C

ATOM 2466 CG ASP 308 12.957 52.447 12.955 1.00 0.00 C

ATOM 2467 OD1 ASP 308 13.598 51.878 12.032 1.00 0.00 O

ATOM 2468 OD2 ASP 308 11.733 52.256 13.185 1.00 0.00 O

ATOM 2469 N LEU 309 17.157 54.254 14.607 1.00 0.00 N

ATOM 2470 CA LEU 309 17.867 55.326 15.236 1.00 0.00 C

ATOM 2471 C LEU 309 18.017 56.388 14.196 1.00 0.00 C

ATOM 2472 O LEU 309 17.779 57.569 14.445 1.00 0.00 O

ATOM 2473 CB LEU 309 19.273 54.886 15.702 1.00 0.00 C

ATOM 2474 CG LEU 309 20.093 55.901 16.539 1.00 0.00 C

ATOM 2475 CD1 LEU 309 21.456 55.299 16.913 1.00 0.00 C

ATOM 2476 CD2 LEU 309 20.260 57.273 15.862 1.00 0.00 C

ATOM 2477 N PHE 310 18.403 55.965 12.981 1.00 0.00 N

ATOM 2478 CA PHE 310 18.658 56.841 11.877 1.00 0.00 C

ATOM 2479 C PHE 310 17.395 57.528 11.470 1.00 0.00 C

ATOM 2480 O PHE 310 17.386 58.727 11.198 1.00 0.00 O

ATOM 2481 CB PHE 310 19.134 56.078 10.636 1.00 0.00 C

ATOM 2482 CG PHE 310 20.474 55.483 10.904 1.00 0.00 C

ATOM 2483 CD1 PHE 310 21.598 56.274 10.943 1.00 0.00 C

ATOM 2484 CD2 PHE 310 20.608 54.127 11.087 1.00 0.00 C

ATOM 2485 CE1 PHE 310 22.836 55.726 11.179 1.00 0.00 C

ATOM 2486 CE2 PHE 310 21.843 53.571 11.323 1.00 0.00 C

ATOM 2487 CZ PHE 310 22.959 54.372 11.371 1.00 0.00 C

ATOM 2488 N LYS 311 16.290 56.765 11.429 1.00 0.00 N

ATOM 2489 CA LYS 311 15.016 57.238 10.967 1.00 0.00 C

ATOM 2490 C LYS 311 14.561 58.357 11.844 1.00 0.00 C

ATOM 2491 O LYS 311 13.871 59.267 11.390 1.00 0.00 O

ATOM 2492 CB LYS 311 13.928 56.153 11.010 1.00 0.00 C

ATOM 2493 CG LYS 311 14.198 54.989 10.057 1.00 0.00 C

ATOM 2494 CD LYS 311 14.316 55.418 8.593 1.00 0.00 C

ATOM 2495 CE LYS 311 12.989 55.863 7.975 1.00 0.00 C

ATOM 2496 NZ LYS 311 12.065 54.712 7.877 1.00 0.00 N

ATOM 2497 N ALA 312 14.958 58.326 13.127 1.00 0.00 N

ATOM 2498 CA ALA 312 14.498 59.290 14.083 1.00 0.00 C

ATOM 2499 C ALA 312 14.843 60.673 13.620 1.00 0.00 C

ATOM 2500 O ALA 312 14.061 61.604 13.800 1.00 0.00 O

ATOM 2501 CB ALA 312 15.128 59.095 15.474 1.00 0.00 C

ATOM 2502 N ILE 313 16.016 60.844 12.989 1.00 0.00 N

ATOM 2503 CA ILE 313 16.492 62.129 12.555 1.00 0.00 C

ATOM 2504 C ILE 313 15.543 62.743 11.569 1.00 0.00 C

ATOM 2505 O ILE 313 15.463 63.965 11.457 1.00 0.00 O

ATOM 2506 CB ILE 313 17.832 62.080 11.893 1.00 0.00 C

ATOM 2507 CG1 ILE 313 18.390 63.505 11.783 1.00 0.00 C

ATOM 2508 CG2 ILE 313 17.673 61.375 10.535 1.00 0.00 C

ATOM 2509 CD1 ILE 313 18.740 64.131 13.133 1.00 0.00 C

ATOM 2510 N SER 314 14.851 61.914 10.770 1.00 0.00 N

ATOM 2511 CA SER 314 13.961 62.409 9.757 1.00 0.00 C

ATOM 2512 C SER 314 12.832 63.171 10.383 1.00 0.00 C

ATOM 2513 O SER 314 12.281 64.080 9.763 1.00 0.00 O

ATOM 2514 CB SER 314 13.338 61.281 8.919 1.00 0.00 C

ATOM 2515 OG SER 314 14.346 60.606 8.181 1.00 0.00 O

ATOM 2516 N ALA 315 12.456 62.831 11.631 1.00 0.00 N

ATOM 2517 CA ALA 315 11.339 63.475 12.260 1.00 0.00 C

ATOM 2518 C ALA 315 11.644 64.927 12.462 1.00 0.00 C

ATOM 2519 O ALA 315 12.789 65.320 12.677 1.00 0.00 O

ATOM 2520 CB ALA 315 10.980 62.865 13.624 1.00 0.00 C

ATOM 2521 N GLY 316 10.595 65.766 12.359 1.00 0.00 N

ATOM 2522 CA GLY 316 10.701 67.189 12.518 1.00 0.00 C

ATOM 2523 C GLY 316 11.068 67.518 13.933 1.00 0.00 C

ATOM 2524 O GLY 316 11.809 68.466 14.185 1.00 0.00 O

ATOM 2525 N ASN 317 10.526 66.741 14.889 1.00 0.00 N

ATOM 2526 CA ASN 317 10.674 66.963 16.301 1.00 0.00 C

ATOM 2527 C ASN 317 12.082 66.733 16.771 1.00 0.00 C

ATOM 2528 O ASN 317 12.437 67.177 17.863 1.00 0.00 O

ATOM 2529 CB ASN 317 9.726 66.114 17.175 1.00 0.00 C

ATOM 2530 CG ASN 317 10.074 64.639 17.050 1.00 0.00 C

ATOM 2531 ND2 ASN 317 10.177 63.941 18.213 1.00 0.00 N

ATOM 2532 OD1 ASN 317 10.246 64.119 15.950 1.00 0.00 O

ATOM 2533 N ALA 318 12.910 65.983 16.014 1.00 0.00 N

ATOM 2534 CA ALA 318 14.235 65.700 16.503 1.00 0.00 C

ATOM 2535 C ALA 318 15.260 66.551 15.814 1.00 0.00 C

ATOM 2536 O ALA 318 15.181 66.799 14.611 1.00 0.00 O

ATOM 2537 CB ALA 318 14.657 64.234 16.306 1.00 0.00 C

ATOM 2538 N ASP 319 16.253 67.042 16.592 1.00 0.00 N

ATOM 2539 CA ASP 319 17.312 67.836 16.034 1.00 0.00 C

ATOM 2540 C ASP 319 18.622 67.354 16.587 1.00 0.00 C

ATOM 2541 O ASP 319 18.696 66.869 17.715 1.00 0.00 O

ATOM 2542 CB ASP 319 17.202 69.342 16.339 1.00 0.00 C

ATOM 2543 CG ASP 319 17.343 69.557 17.838 1.00 0.00 C

ATOM 2544 OD1 ASP 319 16.701 68.799 18.611 1.00 0.00 O

ATOM 2545 OD2 ASP 319 18.087 70.496 18.229 1.00 0.00 O

ATOM 2546 N ILE 320 19.701 67.480 15.785 1.00 0.00 N

ATOM 2547 CA ILE 320 21.013 67.068 16.206 1.00 0.00 C

ATOM 2548 C ILE 320 21.797 68.327 16.409 1.00 0.00 C

ATOM 2549 O ILE 320 21.716 69.244 15.592 1.00 0.00 O

ATOM 2550 CB ILE 320 21.749 66.254 15.181 1.00 0.00 C

ATOM 2551 CG1 ILE 320 20.984 64.957 14.870 1.00 0.00 C

ATOM 2552 CG2 ILE 320 23.175 66.015 15.705 1.00 0.00 C

ATOM 2553 CD1 ILE 320 20.808 64.046 16.084 1.00 0.00 C

ATOM 2554 N VAL 321 22.577 68.413 17.509 1.00 0.00 N

ATOM 2555 CA VAL 321 23.224 69.667 17.780 1.00 0.00 C

ATOM 2556 C VAL 321 24.722 69.576 17.778 1.00 0.00 C

ATOM 2557 O VAL 321 25.303 68.597 18.256 1.00 0.00 O

ATOM 2558 CB VAL 321 22.819 70.261 19.098 1.00 0.00 C

ATOM 2559 CG1 VAL 321 21.332 70.642 19.026 1.00 0.00 C

ATOM 2560 CG2 VAL 321 23.136 69.246 20.210 1.00 0.00 C

ATOM 2561 N THR 322 25.341 70.648 17.205 1.00 0.00 N

ATOM 2562 CA THR 322 26.753 70.926 17.117 1.00 0.00 C

ATOM 2563 C THR 322 27.006 72.063 18.062 1.00 0.00 C

ATOM 2564 O THR 322 26.138 72.394 18.867 1.00 0.00 O

ATOM 2565 CB THR 322 27.193 71.368 15.751 1.00 0.00 C

ATOM 2566 CG2 THR 322 26.593 72.754 15.462 1.00 0.00 C

ATOM 2567 OG1 THR 322 28.611 71.422 15.686 1.00 0.00 O

ATOM 2568 N ASP 323 28.200 72.694 17.991 1.00 0.00 N

ATOM 2569 CA ASP 323 28.502 73.793 18.869 1.00 0.00 C

ATOM 2570 C ASP 323 28.664 73.255 20.255 1.00 0.00 C

ATOM 2571 O ASP 323 27.871 72.440 20.719 1.00 0.00 O

ATOM 2572 CB ASP 323 27.390 74.852 18.935 1.00 0.00 C

ATOM 2573 CG ASP 323 27.351 75.595 17.613 1.00 0.00 C

ATOM 2574 OD1 ASP 323 28.406 76.163 17.225 1.00 0.00 O

ATOM 2575 OD2 ASP 323 26.261 75.618 16.980 1.00 0.00 O

ATOM 2576 N HIS 324 29.710 73.712 20.966 1.00 0.00 N

ATOM 2577 CA HIS 324 29.965 73.220 22.288 1.00 0.00 C

ATOM 2578 C HIS 324 28.916 73.736 23.225 1.00 0.00 C

ATOM 2579 O HIS 324 28.341 74.799 22.998 1.00 0.00 O

ATOM 2580 CB HIS 324 31.336 73.644 22.843 1.00 0.00 C

ATOM 2581 CG HIS 324 32.486 73.065 22.072 1.00 0.00 C

ATOM 2582 CD2 HIS 324 33.214 71.938 22.304 1.00 0.00 C

ATOM 2583 ND1 HIS 324 33.016 73.625 20.930 1.00 0.00 N

ATOM 2584 CE1 HIS 324 34.029 72.815 20.531 1.00 0.00 C

ATOM 2585 NE2 HIS 324 34.187 71.779 21.334 1.00 0.00 N

ATOM 2586 N ILE 325 28.606 72.971 24.300 1.00 0.00 N

ATOM 2587 CA ILE 325 27.659 73.498 25.244 1.00 0.00 C

ATOM 2588 C ILE 325 28.394 74.381 26.184 1.00 0.00 C

ATOM 2589 O ILE 325 29.351 73.971 26.840 1.00 0.00 O

ATOM 2590 CB ILE 325 26.884 72.499 26.080 1.00 0.00 C

ATOM 2591 CG1 ILE 325 27.783 71.538 26.875 1.00 0.00 C

ATOM 2592 CG2 ILE 325 25.866 71.800 25.178 1.00 0.00 C

ATOM 2593 CD1 ILE 325 28.428 70.446 26.021 1.00 0.00 C

ATOM 2594 N GLU 326 27.975 75.656 26.215 1.00 0.00 N

ATOM 2595 CA GLU 326 28.588 76.622 27.065 1.00 0.00 C

ATOM 2596 C GLU 326 28.222 76.371 28.493 1.00 0.00 C

ATOM 2597 O GLU 326 29.089 76.373 29.366 1.00 0.00 O

ATOM 2598 CB GLU 326 28.169 78.052 26.705 1.00 0.00 C

ATOM 2599 CG GLU 326 28.989 79.115 27.429 1.00 0.00 C

ATOM 2600 CD GLU 326 28.618 80.461 26.833 1.00 0.00 C

ATOM 2601 OE1 GLU 326 27.805 80.481 25.871 1.00 0.00 O

ATOM 2602 OE2 GLU 326 29.146 81.490 27.332 1.00 0.00 O

ATOM 2603 N ARG 327 26.926 76.133 28.779 1.00 0.00 N

ATOM 2604 CA ARG 327 26.549 75.981 30.154 1.00 0.00 C

ATOM 2605 C ARG 327 25.149 75.468 30.205 1.00 0.00 C

ATOM 2606 O ARG 327 24.442 75.433 29.199 1.00 0.00 O

ATOM 2607 CB ARG 327 26.579 77.321 30.917 1.00 0.00 C

ATOM 2608 CG ARG 327 26.189 77.235 32.395 1.00 0.00 C

ATOM 2609 CD ARG 327 26.227 78.583 33.119 1.00 0.00 C

ATOM 2610 NE ARG 327 25.816 78.342 34.531 1.00 0.00 N

ATOM 2611 CZ ARG 327 25.948 79.333 35.460 1.00 0.00 C

ATOM 2612 NH1 ARG 327 26.454 80.548 35.097 1.00 0.00 N

ATOM 2613 NH2 ARG 327 25.573 79.110 36.753 1.00 0.00 N

ATOM 2614 N PHE 328 24.718 75.040 31.406 1.00 0.00 N

ATOM 2615 CA PHE 328 23.381 74.567 31.563 1.00 0.00 C

ATOM 2616 C PHE 328 22.695 75.649 32.339 1.00 0.00 C

ATOM 2617 O PHE 328 23.298 76.267 33.216 1.00 0.00 O

ATOM 2618 CB PHE 328 23.296 73.282 32.398 1.00 0.00 C

ATOM 2619 CG PHE 328 22.046 72.619 31.962 1.00 0.00 C

ATOM 2620 CD1 PHE 328 20.826 73.119 32.335 1.00 0.00 C

ATOM 2621 CD2 PHE 328 22.107 71.522 31.135 1.00 0.00 C

ATOM 2622 CE1 PHE 328 19.682 72.499 31.905 1.00 0.00 C

ATOM 2623 CE2 PHE 328 20.960 70.899 30.708 1.00 0.00 C

ATOM 2624 CZ PHE 328 19.742 71.391 31.098 1.00 0.00 C

ATOM 2625 N THR 329 21.416 75.922 32.021 1.00 0.00 N

ATOM 2626 CA THR 329 20.691 76.967 32.684 1.00 0.00 C

ATOM 2627 C THR 329 19.430 76.370 33.225 1.00 0.00 C

ATOM 2628 O THR 329 19.148 75.192 33.014 1.00 0.00 O

ATOM 2629 CB THR 329 20.315 78.098 31.772 1.00 0.00 C

ATOM 2630 CG2 THR 329 21.602 78.692 31.173 1.00 0.00 C

ATOM 2631 OG1 THR 329 19.465 77.632 30.735 1.00 0.00 O

ATOM 2632 N GLU 330 18.639 77.176 33.962 1.00 0.00 N

ATOM 2633 CA GLU 330 17.437 76.658 34.543 1.00 0.00 C

ATOM 2634 C GLU 330 16.575 76.178 33.422 1.00 0.00 C

ATOM 2635 O GLU 330 15.934 75.133 33.523 1.00 0.00 O

ATOM 2636 CB GLU 330 16.620 77.710 35.317 1.00 0.00 C

ATOM 2637 CG GLU 330 17.280 78.203 36.607 1.00 0.00 C

ATOM 2638 CD GLU 330 18.254 79.314 36.249 1.00 0.00 C

ATOM 2639 OE1 GLU 330 18.331 79.665 35.041 1.00 0.00 O

ATOM 2640 OE2 GLU 330 18.936 79.827 37.176 1.00 0.00 O

ATOM 2641 N HIS 331 16.547 76.957 32.325 1.00 0.00 N

ATOM 2642 CA HIS 331 15.743 76.707 31.161 1.00 0.00 C

ATOM 2643 C HIS 331 16.197 75.488 30.403 1.00 0.00 C

ATOM 2644 O HIS 331 15.363 74.708 29.945 1.00 0.00 O

ATOM 2645 CB HIS 331 15.769 77.892 30.181 1.00 0.00 C

ATOM 2646 CG HIS 331 15.296 79.172 30.805 1.00 0.00 C

ATOM 2647 CD2 HIS 331 14.057 79.737 30.829 1.00 0.00 C

ATOM 2648 ND1 HIS 331 16.113 80.030 31.507 1.00 0.00 N

ATOM 2649 CE1 HIS 331 15.335 81.064 31.918 1.00 0.00 C

ATOM 2650 NE2 HIS 331 14.079 80.930 31.530 1.00 0.00 N

ATOM 2651 N GLY 332 17.521 75.280 30.228 1.00 0.00 N

ATOM 2652 CA GLY 332 17.951 74.158 29.429 1.00 0.00 C

ATOM 2653 C GLY 332 19.428 74.274 29.194 1.00 0.00 C

ATOM 2654 O GLY 332 20.163 74.689 30.085 1.00 0.00 O

ATOM 2655 N VAL 333 19.909 73.869 27.993 1.00 0.00 N

ATOM 2656 CA VAL 333 21.314 73.969 27.685 1.00 0.00 C

ATOM 2657 C VAL 333 21.556 75.099 26.739 1.00 0.00 C

ATOM 2658 O VAL 333 20.775 75.362 25.827 1.00 0.00 O

ATOM 2659 CB VAL 333 21.956 72.749 27.071 1.00 0.00 C

ATOM 2660 CG1 VAL 333 22.185 71.681 28.146 1.00 0.00 C

ATOM 2661 CG2 VAL 333 21.070 72.248 25.923 1.00 0.00 C

ATOM 2662 N LEU 334 22.677 75.809 26.966 1.00 0.00 N

ATOM 2663 CA LEU 334 23.074 76.928 26.165 1.00 0.00 C

ATOM 2664 C LEU 334 24.331 76.529 25.453 1.00 0.00 C

ATOM 2665 O LEU 334 25.251 75.991 26.068 1.00 0.00 O

ATOM 2666 CB LEU 334 23.347 78.164 27.050 1.00 0.00 C

ATOM 2667 CG LEU 334 23.839 79.438 26.338 1.00 0.00 C

ATOM 2668 CD1 LEU 334 25.292 79.287 25.869 1.00 0.00 C

ATOM 2669 CD2 LEU 334 22.879 79.875 25.222 1.00 0.00 C

ATOM 2670 N LEU 335 24.393 76.760 24.123 1.00 0.00 N

ATOM 2671 CA LEU 335 25.570 76.396 23.381 1.00 0.00 C

ATOM 2672 C LEU 335 26.344 77.642 23.068 1.00 0.00 C

ATOM 2673 O LEU 335 25.859 78.758 23.245 1.00 0.00 O

ATOM 2674 CB LEU 335 25.309 75.661 22.047 1.00 0.00 C

ATOM 2675 CG LEU 335 24.698 76.530 20.932 1.00 0.00 C

ATOM 2676 CD1 LEU 335 24.475 75.709 19.652 1.00 0.00 C

ATOM 2677 CD2 LEU 335 23.422 77.237 21.404 1.00 0.00 C

ATOM 2678 N LYS 336 27.581 77.466 22.563 1.00 0.00 N

ATOM 2679 CA LYS 336 28.465 78.561 22.274 1.00 0.00 C

ATOM 2680 C LYS 336 27.820 79.471 21.283 1.00 0.00 C

ATOM 2681 O LYS 336 28.061 80.678 21.298 1.00 0.00 O

ATOM 2682 CB LYS 336 29.827 78.128 21.701 1.00 0.00 C

ATOM 2683 CG LYS 336 30.843 77.709 22.768 1.00 0.00 C

ATOM 2684 CD LYS 336 30.480 76.429 23.520 1.00 0.00 C

ATOM 2685 CE LYS 336 31.509 76.032 24.581 1.00 0.00 C

ATOM 2686 NZ LYS 336 31.365 76.898 25.772 1.00 0.00 N

ATOM 2687 N SER 337 26.981 78.925 20.387 1.00 0.00 N

ATOM 2688 CA SER 337 26.351 79.760 19.407 1.00 0.00 C

ATOM 2689 C SER 337 25.539 80.782 20.139 1.00 0.00 C

ATOM 2690 O SER 337 25.234 81.842 19.594 1.00 0.00 O

ATOM 2691 CB SER 337 25.408 78.994 18.465 1.00 0.00 C

ATOM 2692 OG SER 337 26.155 78.111 17.642 1.00 0.00 O

ATOM 2693 N GLY 338 25.179 80.503 21.406 1.00 0.00 N

ATOM 2694 CA GLY 338 24.427 81.475 22.146 1.00 0.00 C

ATOM 2695 C GLY 338 22.975 81.143 22.041 1.00 0.00 C

ATOM 2696 O GLY 338 22.122 81.941 22.427 1.00 0.00 O

ATOM 2697 N LYS 339 22.658 79.958 21.485 1.00 0.00 N

ATOM 2698 CA LYS 339 21.290 79.540 21.396 1.00 0.00 C

ATOM 2699 C LYS 339 20.925 78.875 22.682 1.00 0.00 C

ATOM 2700 O LYS 339 21.751 78.237 23.333 1.00 0.00 O

ATOM 2701 CB LYS 339 21.015 78.532 20.269 1.00 0.00 C

ATOM 2702 CG LYS 339 19.550 78.103 20.192 1.00 0.00 C

ATOM 2703 CD LYS 339 19.197 77.334 18.919 1.00 0.00 C

ATOM 2704 CE LYS 339 17.729 76.908 18.854 1.00 0.00 C

ATOM 2705 NZ LYS 339 17.471 76.173 17.597 1.00 0.00 N

ATOM 2706 N MET 340 19.656 79.026 23.091 1.00 0.00 N

ATOM 2707 CA MET 340 19.209 78.420 24.305 1.00 0.00 C

ATOM 2708 C MET 340 18.226 77.376 23.908 1.00 0.00 C

ATOM 2709 O MET 340 17.189 77.686 23.325 1.00 0.00 O

ATOM 2710 CB MET 340 18.467 79.431 25.202 1.00 0.00 C

ATOM 2711 CG MET 340 17.939 78.902 26.539 1.00 0.00 C

ATOM 2712 SD MET 340 19.198 78.604 27.815 1.00 0.00 S

ATOM 2713 CE MET 340 19.241 76.807 27.578 1.00 0.00 C

ATOM 2714 N LEU 341 18.534 76.094 24.180 1.00 0.00 N

ATOM 2715 CA LEU 341 17.528 75.125 23.886 1.00 0.00 C

ATOM 2716 C LEU 341 16.828 74.845 25.171 1.00 0.00 C

ATOM 2717 O LEU 341 17.404 74.295 26.109 1.00 0.00 O

ATOM 2718 CB LEU 341 18.016 73.799 23.260 1.00 0.00 C

ATOM 2719 CG LEU 341 19.382 73.282 23.737 1.00 0.00 C

ATOM 2720 CD1 LEU 341 19.659 71.875 23.184 1.00 0.00 C

ATOM 2721 CD2 LEU 341 20.506 74.253 23.340 1.00 0.00 C

ATOM 2722 N LYS 342 15.548 75.255 25.241 1.00 0.00 N

ATOM 2723 CA LYS 342 14.783 75.066 26.434 1.00 0.00 C

ATOM 2724 C LYS 342 14.506 73.608 26.537 1.00 0.00 C

ATOM 2725 O LYS 342 14.230 72.952 25.535 1.00 0.00 O

ATOM 2726 CB LYS 342 13.425 75.786 26.414 1.00 0.00 C

ATOM 2727 CG LYS 342 12.609 75.594 27.694 1.00 0.00 C

ATOM 2728 CD LYS 342 13.168 76.359 28.893 1.00 0.00 C

ATOM 2729 CE LYS 342 12.826 77.850 28.864 1.00 0.00 C

ATOM 2730 NZ LYS 342 11.403 78.050 29.216 1.00 0.00 N

ATOM 2731 N ALA 343 14.598 73.047 27.758 1.00 0.00 N

ATOM 2732 CA ALA 343 14.324 71.648 27.866 1.00 0.00 C

ATOM 2733 C ALA 343 13.649 71.373 29.170 1.00 0.00 C

ATOM 2734 O ALA 343 13.964 71.981 30.193 1.00 0.00 O

ATOM 2735 CB ALA 343 15.587 70.771 27.827 1.00 0.00 C

ATOM 2736 N ASP 344 12.635 70.488 29.126 1.00 0.00 N

ATOM 2737 CA ASP 344 11.968 70.017 30.301 1.00 0.00 C

ATOM 2738 C ASP 344 12.852 69.026 30.990 1.00 0.00 C

ATOM 2739 O ASP 344 12.940 69.013 32.217 1.00 0.00 O

ATOM 2740 CB ASP 344 10.629 69.336 29.981 1.00 0.00 C

ATOM 2741 CG ASP 344 9.674 70.441 29.556 1.00 0.00 C

ATOM 2742 OD1 ASP 344 9.700 71.518 30.209 1.00 0.00 O

ATOM 2743 OD2 ASP 344 8.919 70.235 28.569 1.00 0.00 O

ATOM 2744 N ILE 345 13.529 68.147 30.218 1.00 0.00 N

ATOM 2745 CA ILE 345 14.343 67.178 30.889 1.00 0.00 C

ATOM 2746 C ILE 345 15.610 66.963 30.117 1.00 0.00 C

ATOM 2747 O ILE 345 15.648 67.119 28.897 1.00 0.00 O

ATOM 2748 CB ILE 345 13.687 65.848 31.036 1.00 0.00 C

ATOM 2749 CG1 ILE 345 13.559 65.152 29.683 1.00 0.00 C

ATOM 2750 CG2 ILE 345 12.318 66.093 31.689 1.00 0.00 C

ATOM 2751 CD1 ILE 345 13.277 63.672 29.873 1.00 0.00 C

ATOM 2752 N ILE 346 16.699 66.609 30.833 1.00 0.00 N

ATOM 2753 CA ILE 346 17.961 66.338 30.207 1.00 0.00 C

ATOM 2754 C ILE 346 18.318 64.925 30.540 1.00 0.00 C

ATOM 2755 O ILE 346 18.285 64.531 31.705 1.00 0.00 O

ATOM 2756 CB ILE 346 19.084 67.198 30.722 1.00 0.00 C

ATOM 2757 CG1 ILE 346 18.872 68.674 30.347 1.00 0.00 C

ATOM 2758 CG2 ILE 346 20.403 66.617 30.182 1.00 0.00 C

ATOM 2759 CD1 ILE 346 17.658 69.312 31.006 1.00 0.00 C

ATOM 2760 N VAL 347 18.662 64.111 29.519 1.00 0.00 N

ATOM 2761 CA VAL 347 19.002 62.753 29.819 1.00 0.00 C

ATOM 2762 C VAL 347 20.441 62.562 29.447 1.00 0.00 C

ATOM 2763 O VAL 347 20.894 62.986 28.383 1.00 0.00 O

ATOM 2764 CB VAL 347 18.143 61.744 29.109 1.00 0.00 C

ATOM 2765 CG1 VAL 347 18.731 61.444 27.731 1.00 0.00 C

ATOM 2766 CG2 VAL 347 17.965 60.523 30.016 1.00 0.00 C

ATOM 2767 N THR 348 21.218 61.923 30.342 1.00 0.00 N

ATOM 2768 CA THR 348 22.625 61.801 30.081 1.00 0.00 C

ATOM 2769 C THR 348 22.996 60.389 29.779 1.00 0.00 C

ATOM 2770 O THR 348 23.221 59.605 30.697 1.00 0.00 O

ATOM 2771 CB THR 348 23.446 62.250 31.254 1.00 0.00 C

ATOM 2772 CG2 THR 348 24.932 62.034 30.943 1.00 0.00 C

ATOM 2773 OG1 THR 348 23.192 63.620 31.531 1.00 0.00 O

ATOM 2774 N ALA 349 23.106 60.027 28.481 1.00 0.00 N

ATOM 2775 CA ALA 349 23.537 58.688 28.170 1.00 0.00 C

ATOM 2776 C ALA 349 25.011 58.792 27.995 1.00 0.00 C

ATOM 2777 O ALA 349 25.565 58.389 26.975 1.00 0.00 O

ATOM 2778 CB ALA 349 22.943 58.182 26.841 1.00 0.00 C

ATOM 2779 N THR 350 25.691 59.298 29.041 1.00 0.00 N

ATOM 2780 CA THR 350 27.120 59.283 29.075 1.00 0.00 C

ATOM 2781 C THR 350 27.282 57.883 29.641 1.00 0.00 C

ATOM 2782 O THR 350 26.306 57.136 29.739 1.00 0.00 O

ATOM 2783 CB THR 350 27.698 60.292 30.023 1.00 0.00 C

ATOM 2784 CG2 THR 350 29.226 60.155 30.024 1.00 0.00 C

ATOM 2785 OG1 THR 350 27.340 61.608 29.624 1.00 0.00 O

ATOM 2786 N GLY 351 28.493 57.419 29.968 1.00 0.00 N

ATOM 2787 CA GLY 351 28.569 56.116 30.584 1.00 0.00 C

ATOM 2788 C GLY 351 28.968 56.353 31.987 1.00 0.00 C

ATOM 2789 O GLY 351 28.487 57.297 32.607 1.00 0.00 O

ATOM 2790 N LEU 352 29.884 55.500 32.491 1.00 0.00 N

ATOM 2791 CA LEU 352 30.308 55.518 33.859 1.00 0.00 C

ATOM 2792 C LEU 352 31.807 55.625 33.810 1.00 0.00 C

ATOM 2793 O LEU 352 32.436 55.086 32.899 1.00 0.00 O

ATOM 2794 CB LEU 352 29.904 54.201 34.541 1.00 0.00 C

ATOM 2795 CG LEU 352 28.462 53.801 34.131 1.00 0.00 C

ATOM 2796 CD1 LEU 352 28.423 53.115 32.754 1.00 0.00 C

ATOM 2797 CD2 LEU 352 27.730 52.988 35.191 1.00 0.00 C

ATOM 2798 N ASN 353 32.423 56.340 34.780 1.00 0.00 N

ATOM 2799 CA ASN 353 33.854 56.485 34.791 1.00 0.00 C

ATOM 2800 C ASN 353 34.373 55.102 35.037 1.00 0.00 C

ATOM 2801 O ASN 353 33.599 54.186 35.314 1.00 0.00 O

ATOM 2802 CB ASN 353 34.402 57.402 35.897 1.00 0.00 C

ATOM 2803 CG ASN 353 35.859 57.715 35.581 1.00 0.00 C

ATOM 2804 ND2 ASN 353 36.563 58.364 36.547 1.00 0.00 N

ATOM 2805 OD1 ASN 353 36.359 57.390 34.505 1.00 0.00 O

ATOM 2806 N VAL 354 35.695 54.888 34.898 1.00 0.00 N

ATOM 2807 CA VAL 354 36.180 53.552 35.078 1.00 0.00 C

ATOM 2808 C VAL 354 36.220 53.209 36.537 1.00 0.00 C

ATOM 2809 O VAL 354 36.842 53.918 37.324 1.00 0.00 O

ATOM 2810 CB VAL 354 37.554 53.336 34.517 1.00 0.00 C

ATOM 2811 CG1 VAL 354 38.006 51.905 34.854 1.00 0.00 C

ATOM 2812 CG2 VAL 354 37.512 53.639 33.010 1.00 0.00 C

ATOM 2813 N GLN 355 35.562 52.093 36.933 1.00 0.00 N

ATOM 2814 CA GLN 355 35.529 51.696 38.317 1.00 0.00 C

ATOM 2815 C GLN 355 35.361 50.219 38.473 1.00 0.00 C

ATOM 2816 O GLN 355 35.882 49.421 37.698 1.00 0.00 O

ATOM 2817 CB GLN 355 34.434 52.331 39.194 1.00 0.00 C

ATOM 2818 CG GLN 355 34.827 53.655 39.853 1.00 0.00 C

ATOM 2819 CD GLN 355 34.722 54.768 38.834 1.00 0.00 C

ATOM 2820 NE2 GLN 355 35.258 55.964 39.192 1.00 0.00 N

ATOM 2821 OE1 GLN 355 34.179 54.579 37.748 1.00 0.00 O

ATOM 2822 N LEU 356 34.625 49.839 39.542 1.00 0.00 N

ATOM 2823 CA LEU 356 34.469 48.478 39.960 1.00 0.00 C

ATOM 2824 C LEU 356 35.707 48.138 40.684 1.00 0.00 C

ATOM 2825 O LEU 356 35.922 48.584 41.808 1.00 0.00 O

ATOM 2826 CB LEU 356 34.230 47.429 38.865 1.00 0.00 C

ATOM 2827 CG LEU 356 32.757 47.351 38.443 1.00 0.00 C

ATOM 2828 CD1 LEU 356 32.515 46.173 37.496 1.00 0.00 C

ATOM 2829 CD2 LEU 356 31.830 47.322 39.668 1.00 0.00 C

ATOM 2830 N PHE 357 36.555 47.310 40.062 1.00 0.00 N

ATOM 2831 CA PHE 357 37.798 47.046 40.703 1.00 0.00 C

ATOM 2832 C PHE 357 38.453 48.387 40.733 1.00 0.00 C

ATOM 2833 O PHE 357 38.100 49.261 39.954 1.00 0.00 O

ATOM 2834 CB PHE 357 38.687 46.084 39.900 1.00 0.00 C

ATOM 2835 CG PHE 357 39.768 45.585 40.792 1.00 0.00 C

ATOM 2836 CD1 PHE 357 39.556 44.467 41.565 1.00 0.00 C

ATOM 2837 CD2 PHE 357 40.984 46.223 40.860 1.00 0.00 C

ATOM 2838 CE1 PHE 357 40.542 43.988 42.394 1.00 0.00 C

ATOM 2839 CE2 PHE 357 41.973 45.749 41.689 1.00 0.00 C

ATOM 2840 CZ PHE 357 41.754 44.630 42.456 1.00 0.00 C

ATOM 2841 N GLY 358 39.350 48.645 41.689 1.00 0.00 N

ATOM 2842 CA GLY 358 40.029 49.909 41.688 1.00 0.00 C

ATOM 2843 C GLY 358 39.363 50.805 42.681 1.00 0.00 C

ATOM 2844 O GLY 358 40.025 51.430 43.508 1.00 0.00 O

ATOM 2845 N GLY 359 38.023 50.905 42.619 1.00 0.00 N

ATOM 2846 CA GLY 359 37.332 51.698 43.592 1.00 0.00 C

ATOM 2847 C GLY 359 37.488 50.969 44.883 1.00 0.00 C

ATOM 2848 O GLY 359 37.641 51.562 45.950 1.00 0.00 O

ATOM 2849 N ILE 360 37.416 49.629 44.802 1.00 0.00 N

ATOM 2850 CA ILE 360 37.606 48.818 45.965 1.00 0.00 C

ATOM 2851 C ILE 360 39.077 48.608 46.066 1.00 0.00 C

ATOM 2852 O ILE 360 39.751 48.418 45.055 1.00 0.00 O

ATOM 2853 CB ILE 360 36.925 47.477 45.892 1.00 0.00 C

ATOM 2854 CG1 ILE 360 36.978 46.767 47.252 1.00 0.00 C

ATOM 2855 CG2 ILE 360 37.550 46.664 44.749 1.00 0.00 C

ATOM 2856 CD1 ILE 360 36.066 45.544 47.333 1.00 0.00 C

ATOM 2857 N THR 361 39.629 48.673 47.290 1.00 0.00 N

ATOM 2858 CA THR 361 41.054 48.558 47.409 1.00 0.00 C

ATOM 2859 C THR 361 41.427 47.125 47.646 1.00 0.00 C

ATOM 2860 O THR 361 40.803 46.448 48.462 1.00 0.00 O

ATOM 2861 CB THR 361 41.628 49.361 48.539 1.00 0.00 C

ATOM 2862 CG2 THR 361 41.120 48.783 49.871 1.00 0.00 C

ATOM 2863 OG1 THR 361 43.047 49.325 48.495 1.00 0.00 O

ATOM 2864 N LEU 362 42.452 46.625 46.908 1.00 0.00 N

ATOM 2865 CA LEU 362 42.899 45.254 47.051 1.00 0.00 C

ATOM 2866 C LEU 362 44.380 45.166 46.660 1.00 0.00 C

ATOM 2867 O LEU 362 44.911 46.188 46.228 1.00 0.00 O

ATOM 2868 CB LEU 362 42.240 44.294 46.043 1.00 0.00 C

ATOM 2869 CG LEU 362 40.711 44.404 45.919 1.00 0.00 C

ATOM 2870 CD1 LEU 362 40.005 44.123 47.245 1.00 0.00 C

ATOM 2871 CD2 LEU 362 40.299 45.741 45.289 1.00 0.00 C

ATOM 2872 N HIS 363 45.070 43.978 46.881 1.00 0.00 N

ATOM 2873 CA HIS 363 46.358 43.572 46.309 1.00 0.00 C

ATOM 2874 C HIS 363 47.484 43.347 47.293 1.00 0.00 C

ATOM 2875 O HIS 363 47.440 43.768 48.447 1.00 0.00 O

ATOM 2876 CB HIS 363 46.842 44.556 45.235 1.00 0.00 C

ATOM 2877 CG HIS 363 47.761 44.049 44.171 1.00 0.00 C

ATOM 2878 CD2 HIS 363 49.117 43.934 44.138 1.00 0.00 C

ATOM 2879 ND1 HIS 363 47.323 43.728 42.907 1.00 0.00 N

ATOM 2880 CE1 HIS 363 48.426 43.458 42.171 1.00 0.00 C

ATOM 2881 NE2 HIS 363 49.539 43.579 42.873 1.00 0.00 N

ATOM 2882 N LYS 364 48.555 42.658 46.817 1.00 0.00 N

ATOM 2883 CA LYS 364 49.735 42.371 47.596 1.00 0.00 C

ATOM 2884 C LYS 364 50.556 43.611 47.684 1.00 0.00 C

ATOM 2885 O LYS 364 50.391 44.535 46.890 1.00 0.00 O

ATOM 2886 CB LYS 364 50.653 41.284 47.004 1.00 0.00 C

ATOM 2887 CG LYS 364 50.144 39.852 47.173 1.00 0.00 C

ATOM 2888 CD LYS 364 50.912 38.830 46.329 1.00 0.00 C

ATOM 2889 CE LYS 364 52.410 38.762 46.636 1.00 0.00 C

ATOM 2890 NZ LYS 364 52.652 37.869 47.790 1.00 0.00 N

ATOM 2891 N ASP 365 51.431 43.657 48.711 1.00 0.00 N

ATOM 2892 CA ASP 365 52.334 44.732 49.009 1.00 0.00 C

ATOM 2893 C ASP 365 51.533 45.975 49.191 1.00 0.00 C

ATOM 2894 O ASP 365 52.056 47.086 49.133 1.00 0.00 O

ATOM 2895 CB ASP 365 53.475 44.943 47.978 1.00 0.00 C

ATOM 2896 CG ASP 365 52.971 45.395 46.610 1.00 0.00 C

ATOM 2897 OD1 ASP 365 52.142 46.342 46.546 1.00 0.00 O

ATOM 2898 OD2 ASP 365 53.415 44.785 45.601 1.00 0.00 O

ATOM 2899 N GLY 366 50.223 45.798 49.436 1.00 0.00 N

ATOM 2900 CA GLY 366 49.351 46.892 49.725 1.00 0.00 C

ATOM 2901 C GLY 366 49.272 47.818 48.553 1.00 0.00 C

ATOM 2902 O GLY 366 48.962 48.995 48.729 1.00 0.00 O

ATOM 2903 N LYS 367 49.538 47.345 47.319 1.00 0.00 N

ATOM 2904 CA LYS 367 49.473 48.313 46.260 1.00 0.00 C

ATOM 2905 C LYS 367 48.680 47.758 45.129 1.00 0.00 C

ATOM 2906 O LYS 367 48.691 46.554 44.913 1.00 0.00 O

ATOM 2907 CB LYS 367 50.853 48.701 45.702 1.00 0.00 C

ATOM 2908 CG LYS 367 50.805 49.844 44.687 1.00 0.00 C

ATOM 2909 CD LYS 367 52.177 50.456 44.398 1.00 0.00 C

ATOM 2910 CE LYS 367 52.141 51.601 43.383 1.00 0.00 C

ATOM 2911 NZ LYS 367 53.503 52.146 43.191 1.00 0.00 N

ATOM 2912 N PRO 368 47.921 48.584 44.453 1.00 0.00 N

ATOM 2913 CA PRO 368 47.227 48.148 43.264 1.00 0.00 C

ATOM 2914 C PRO 368 48.107 48.281 42.034 1.00 0.00 C

ATOM 2915 O PRO 368 48.809 49.284 41.929 1.00 0.00 O

ATOM 2916 CB PRO 368 45.951 48.987 43.178 1.00 0.00 C

ATOM 2917 CG PRO 368 46.226 50.194 44.091 1.00 0.00 C

ATOM 2918 CD PRO 368 47.197 49.638 45.144 1.00 0.00 C

ATOM 2919 N VAL 369 48.106 47.280 41.118 1.00 0.00 N

ATOM 2920 CA VAL 369 48.821 47.219 39.854 1.00 0.00 C

ATOM 2921 C VAL 369 48.119 47.889 38.701 1.00 0.00 C

ATOM 2922 O VAL 369 48.753 48.259 37.714 1.00 0.00 O

ATOM 2923 CB VAL 369 49.132 45.818 39.412 1.00 0.00 C

ATOM 2924 CG1 VAL 369 50.144 45.207 40.396 1.00 0.00 C

ATOM 2925 CG2 VAL 369 47.815 45.033 39.292 1.00 0.00 C

ATOM 2926 N VAL 370 46.791 48.070 38.792 1.00 0.00 N

ATOM 2927 CA VAL 370 45.943 48.436 37.685 1.00 0.00 C

ATOM 2928 C VAL 370 46.443 49.628 36.938 1.00 0.00 C

ATOM 2929 O VAL 370 46.202 49.696 35.733 1.00 0.00 O

ATOM 2930 CB VAL 370 44.540 48.747 38.118 1.00 0.00 C

ATOM 2931 CG1 VAL 370 43.917 47.473 38.714 1.00 0.00 C

ATOM 2932 CG2 VAL 370 44.580 49.935 39.095 1.00 0.00 C

ATOM 2933 N LEU 371 47.106 50.598 37.610 1.00 0.00 N

ATOM 2934 CA LEU 371 47.574 51.781 36.932 1.00 0.00 C

ATOM 2935 C LEU 371 48.388 51.314 35.769 1.00 0.00 C

ATOM 2936 O LEU 371 49.519 50.850 35.901 1.00 0.00 O

ATOM 2937 CB LEU 371 48.450 52.674 37.838 1.00 0.00 C

ATOM 2938 CG LEU 371 48.822 54.073 37.292 1.00 0.00 C

ATOM 2939 CD1 LEU 371 49.691 54.825 38.313 1.00 0.00 C

ATOM 2940 CD2 LEU 371 49.477 54.037 35.901 1.00 0.00 C

ATOM 2941 N SER 372 47.774 51.445 34.583 1.00 0.00 N

ATOM 2942 CA SER 372 48.261 50.979 33.323 1.00 0.00 C

ATOM 2943 C SER 372 46.994 50.527 32.694 1.00 0.00 C

ATOM 2944 O SER 372 45.922 50.933 33.137 1.00 0.00 O

ATOM 2945 CB SER 372 49.195 49.759 33.407 1.00 0.00 C

ATOM 2946 OG SER 372 49.620 49.377 32.107 1.00 0.00 O

ATOM 2947 N GLU 373 47.025 49.743 31.608 1.00 0.00 N

ATOM 2948 CA GLU 373 45.710 49.347 31.211 1.00 0.00 C

ATOM 2949 C GLU 373 45.140 48.450 32.269 1.00 0.00 C

ATOM 2950 O GLU 373 44.398 48.868 33.155 1.00 0.00 O

ATOM 2951 CB GLU 373 45.733 48.605 29.857 1.00 0.00 C

ATOM 2952 CG GLU 373 46.796 47.507 29.738 1.00 0.00 C

ATOM 2953 CD GLU 373 48.132 48.172 29.434 1.00 0.00 C

ATOM 2954 OE1 GLU 373 48.142 49.418 29.248 1.00 0.00 O

ATOM 2955 OE2 GLU 373 49.160 47.444 29.380 1.00 0.00 O

ATOM 2956 N THR 374 45.593 47.194 32.268 1.00 0.00 N

ATOM 2957 CA THR 374 45.205 46.210 33.221 1.00 0.00 C

ATOM 2958 C THR 374 46.061 45.067 32.867 1.00 0.00 C

ATOM 2959 O THR 374 46.379 44.865 31.697 1.00 0.00 O

ATOM 2960 CB THR 374 43.787 45.732 33.124 1.00 0.00 C

ATOM 2961 CG2 THR 374 43.584 44.637 34.183 1.00 0.00 C

ATOM 2962 OG1 THR 374 42.883 46.803 33.345 1.00 0.00 O

ATOM 2963 N LEU 375 46.477 44.284 33.861 1.00 0.00 N

ATOM 2964 CA LEU 375 47.291 43.191 33.466 1.00 0.00 C

ATOM 2965 C LEU 375 46.608 41.976 33.975 1.00 0.00 C

ATOM 2966 O LEU 375 46.123 41.959 35.105 1.00 0.00 O

ATOM 2967 CB LEU 375 48.701 43.225 34.076 1.00 0.00 C

ATOM 2968 CG LEU 375 49.539 44.430 33.616 1.00 0.00 C

ATOM 2969 CD1 LEU 375 48.897 45.755 34.057 1.00 0.00 C

ATOM 2970 CD2 LEU 375 51.001 44.298 34.071 1.00 0.00 C

ATOM 2971 N ALA 376 46.514 40.934 33.133 1.00 0.00 N

ATOM 2972 CA ALA 376 45.904 39.726 33.591 1.00 0.00 C

ATOM 2973 C ALA 376 46.505 38.592 32.831 1.00 0.00 C

ATOM 2974 O ALA 376 47.071 38.775 31.754 1.00 0.00 O

ATOM 2975 CB ALA 376 44.386 39.663 33.346 1.00 0.00 C

ATOM 2976 N TYR 377 46.420 37.380 33.410 1.00 0.00 N

ATOM 2977 CA TYR 377 46.869 36.198 32.739 1.00 0.00 C

ATOM 2978 C TYR 377 45.635 35.401 32.485 1.00 0.00 C

ATOM 2979 O TYR 377 44.950 34.978 33.414 1.00 0.00 O

ATOM 2980 CB TYR 377 47.850 35.348 33.570 1.00 0.00 C

ATOM 2981 CG TYR 377 48.163 34.102 32.809 1.00 0.00 C

ATOM 2982 CD1 TYR 377 48.835 34.158 31.609 1.00 0.00 C

ATOM 2983 CD2 TYR 377 47.813 32.871 33.315 1.00 0.00 C

ATOM 2984 CE1 TYR 377 49.127 33.006 30.916 1.00 0.00 C

ATOM 2985 CE2 TYR 377 48.105 31.716 32.626 1.00 0.00 C

ATOM 2986 CZ TYR 377 48.762 31.782 31.422 1.00 0.00 C

ATOM 2987 OH TYR 377 49.064 30.601 30.710 1.00 0.00 O

ATOM 2988 N LYS 378 45.303 35.218 31.193 1.00 0.00 N

ATOM 2989 CA LYS 378 44.154 34.465 30.804 1.00 0.00 C

ATOM 2990 C LYS 378 42.927 35.156 31.296 1.00 0.00 C

ATOM 2991 O LYS 378 41.833 34.603 31.229 1.00 0.00 O

ATOM 2992 CB LYS 378 44.203 33.015 31.306 1.00 0.00 C

ATOM 2993 CG LYS 378 45.198 32.166 30.511 1.00 0.00 C

ATOM 2994 CD LYS 378 45.651 30.888 31.218 1.00 0.00 C

ATOM 2995 CE LYS 378 44.700 30.417 32.316 1.00 0.00 C

ATOM 2996 NZ LYS 378 43.507 29.778 31.722 1.00 0.00 N

ATOM 2997 N GLY 379 43.092 36.403 31.778 1.00 0.00 N

ATOM 2998 CA GLY 379 42.016 37.249 32.204 1.00 0.00 C

ATOM 2999 C GLY 379 41.570 36.882 33.587 1.00 0.00 C

ATOM 3000 O GLY 379 40.855 37.651 34.230 1.00 0.00 O

ATOM 3001 N MET 380 41.926 35.670 34.059 1.00 0.00 N

ATOM 3002 CA MET 380 41.488 35.243 35.362 1.00 0.00 C

ATOM 3003 C MET 380 42.197 35.903 36.504 1.00 0.00 C

ATOM 3004 O MET 380 41.571 36.206 37.520 1.00 0.00 O

ATOM 3005 CB MET 380 41.521 33.723 35.623 1.00 0.00 C

ATOM 3006 CG MET 380 42.899 33.093 35.828 1.00 0.00 C

ATOM 3007 SD MET 380 43.710 32.556 34.301 1.00 0.00 S

ATOM 3008 CE MET 380 42.400 31.365 33.896 1.00 0.00 C

ATOM 3009 N MET 381 43.527 36.112 36.413 1.00 0.00 N

ATOM 3010 CA MET 381 44.158 36.718 37.552 1.00 0.00 C

ATOM 3011 C MET 381 45.378 37.466 37.117 1.00 0.00 C

ATOM 3012 O MET 381 45.831 37.342 35.981 1.00 0.00 O

ATOM 3013 CB MET 381 44.500 35.747 38.708 1.00 0.00 C

ATOM 3014 CG MET 381 45.141 36.420 39.937 1.00 0.00 C

ATOM 3015 SD MET 381 44.144 37.741 40.713 1.00 0.00 S

ATOM 3016 CE MET 381 45.367 39.081 40.678 1.00 0.00 C

ATOM 3017 N LEU 382 45.899 38.315 38.027 1.00 0.00 N

ATOM 3018 CA LEU 382 47.051 39.136 37.820 1.00 0.00 C

ATOM 3019 C LEU 382 48.108 38.744 38.819 1.00 0.00 C

ATOM 3020 O LEU 382 47.825 38.177 39.875 1.00 0.00 O

ATOM 3021 CB LEU 382 46.713 40.637 37.958 1.00 0.00 C

ATOM 3022 CG LEU 382 47.835 41.642 37.619 1.00 0.00 C

ATOM 3023 CD1 LEU 382 48.847 41.812 38.759 1.00 0.00 C

ATOM 3024 CD2 LEU 382 48.507 41.279 36.286 1.00 0.00 C

ATOM 3025 N SER 383 49.376 39.058 38.494 1.00 0.00 N

ATOM 3026 CA SER 383 50.521 38.641 39.259 1.00 0.00 C

ATOM 3027 C SER 383 50.558 39.217 40.650 1.00 0.00 C

ATOM 3028 O SER 383 51.211 38.683 41.533 1.00 0.00 O

ATOM 3029 CB SER 383 51.852 39.019 38.589 1.00 0.00 C

ATOM 3030 OG SER 383 52.940 38.583 39.391 1.00 0.00 O

ATOM 3031 N GLY 384 49.954 40.367 40.913 1.00 0.00 N

ATOM 3032 CA GLY 384 50.036 40.899 42.244 1.00 0.00 C

ATOM 3033 C GLY 384 49.177 40.190 43.238 1.00 0.00 C

ATOM 3034 O GLY 384 49.483 40.201 44.433 1.00 0.00 O

ATOM 3035 N VAL 385 48.016 39.645 42.826 1.00 0.00 N

ATOM 3036 CA VAL 385 47.279 39.111 43.926 1.00 0.00 C

ATOM 3037 C VAL 385 46.829 37.708 43.689 1.00 0.00 C

ATOM 3038 O VAL 385 46.132 37.347 42.743 1.00 0.00 O

ATOM 3039 CB VAL 385 46.125 39.963 44.366 1.00 0.00 C

ATOM 3040 CG1 VAL 385 44.882 39.673 43.515 1.00 0.00 C

ATOM 3041 CG2 VAL 385 45.950 39.749 45.876 1.00 0.00 C

ATOM 3042 N PRO 386 47.258 36.934 44.636 1.00 0.00 N

ATOM 3043 CA PRO 386 47.036 35.516 44.639 1.00 0.00 C

ATOM 3044 C PRO 386 45.643 35.192 45.077 1.00 0.00 C

ATOM 3045 O PRO 386 45.037 35.996 45.783 1.00 0.00 O

ATOM 3046 CB PRO 386 48.045 34.938 45.630 1.00 0.00 C

ATOM 3047 CG PRO 386 49.158 35.993 45.707 1.00 0.00 C

ATOM 3048 CD PRO 386 48.449 37.311 45.376 1.00 0.00 C

ATOM 3049 N ASN 387 45.130 34.013 44.672 1.00 0.00 N

ATOM 3050 CA ASN 387 43.854 33.521 45.110 1.00 0.00 C

ATOM 3051 C ASN 387 42.787 34.502 44.783 1.00 0.00 C

ATOM 3052 O ASN 387 41.739 34.530 45.429 1.00 0.00 O

ATOM 3053 CB ASN 387 43.779 33.264 46.624 1.00 0.00 C

ATOM 3054 CG ASN 387 44.628 32.050 46.956 1.00 0.00 C

ATOM 3055 ND2 ASN 387 44.708 31.717 48.272 1.00 0.00 N

ATOM 3056 OD1 ASN 387 45.193 31.408 46.072 1.00 0.00 O

ATOM 3057 N PHE 388 43.014 35.312 43.741 1.00 0.00 N

ATOM 3058 CA PHE 388 42.040 36.285 43.377 1.00 0.00 C

ATOM 3059 C PHE 388 41.633 35.930 41.986 1.00 0.00 C

ATOM 3060 O PHE 388 42.451 35.506 41.170 1.00 0.00 O

ATOM 3061 CB PHE 388 42.636 37.704 43.434 1.00 0.00 C

ATOM 3062 CG PHE 388 41.603 38.728 43.131 1.00 0.00 C

ATOM 3063 CD1 PHE 388 40.494 38.871 43.933 1.00 0.00 C

ATOM 3064 CD2 PHE 388 41.782 39.580 42.070 1.00 0.00 C

ATOM 3065 CE1 PHE 388 39.552 39.831 43.656 1.00 0.00 C

ATOM 3066 CE2 PHE 388 40.843 40.541 41.795 1.00 0.00 C

ATOM 3067 CZ PHE 388 39.728 40.666 42.581 1.00 0.00 C

ATOM 3068 N ALA 389 40.330 36.035 41.685 1.00 0.00 N

ATOM 3069 CA ALA 389 39.923 35.681 40.363 1.00 0.00 C

ATOM 3070 C ALA 389 39.025 36.757 39.867 1.00 0.00 C

ATOM 3071 O ALA 389 38.209 37.305 40.606 1.00 0.00 O

ATOM 3072 CB ALA 389 39.129 34.364 40.297 1.00 0.00 C

ATOM 3073 N PHE 390 39.166 37.082 38.575 1.00 0.00 N

ATOM 3074 CA PHE 390 38.347 38.089 37.981 1.00 0.00 C

ATOM 3075 C PHE 390 37.440 37.376 37.035 1.00 0.00 C

ATOM 3076 O PHE 390 37.878 36.520 36.266 1.00 0.00 O

ATOM 3077 CB PHE 390 39.121 39.060 37.076 1.00 0.00 C

ATOM 3078 CG PHE 390 40.160 39.805 37.834 1.00 0.00 C

ATOM 3079 CD1 PHE 390 41.431 39.291 37.956 1.00 0.00 C

ATOM 3080 CD2 PHE 390 39.868 41.019 38.407 1.00 0.00 C

ATOM 3081 CE1 PHE 390 42.401 39.978 38.646 1.00 0.00 C

ATOM 3082 CE2 PHE 390 40.835 41.711 39.095 1.00 0.00 C

ATOM 3083 CZ PHE 390 42.102 41.191 39.215 1.00 0.00 C

ATOM 3084 N ALA 391 36.140 37.712 37.081 1.00 0.00 N

ATOM 3085 CA ALA 391 35.218 37.167 36.135 1.00 0.00 C

ATOM 3086 C ALA 391 35.131 38.210 35.070 1.00 0.00 C

ATOM 3087 O ALA 391 34.873 39.377 35.360 1.00 0.00 O

ATOM 3088 CB ALA 391 33.805 36.965 36.696 1.00 0.00 C

ATOM 3089 N VAL 392 35.349 37.800 33.807 1.00 0.00 N

ATOM 3090 CA VAL 392 35.349 38.670 32.665 1.00 0.00 C

ATOM 3091 C VAL 392 36.291 39.785 32.867 1.00 0.00 C

ATOM 3092 O VAL 392 35.990 40.954 32.632 1.00 0.00 O

ATOM 3093 CB VAL 392 33.982 39.106 32.244 1.00 0.00 C

ATOM 3094 CG1 VAL 392 33.417 40.199 33.168 1.00 0.00 C

ATOM 3095 CG2 VAL 392 34.104 39.442 30.758 1.00 0.00 C

ATOM 3096 N GLY 393 37.493 39.404 33.319 1.00 0.00 N

ATOM 3097 CA GLY 393 38.538 40.348 33.458 1.00 0.00 C

ATOM 3098 C GLY 393 39.330 40.220 32.194 1.00 0.00 C

ATOM 3099 O GLY 393 39.495 39.124 31.674 1.00 0.00 O

ATOM 3100 N TYR 394 39.686 41.361 31.588 1.00 0.00 N

ATOM 3101 CA TYR 394 40.688 41.524 30.567 1.00 0.00 C

ATOM 3102 C TYR 394 40.891 40.326 29.659 1.00 0.00 C

ATOM 3103 O TYR 394 41.956 39.713 29.717 1.00 0.00 O

ATOM 3104 CB TYR 394 42.003 41.971 31.212 1.00 0.00 C

ATOM 3105 CG TYR 394 41.510 43.013 32.161 1.00 0.00 C

ATOM 3106 CD1 TYR 394 41.196 44.280 31.732 1.00 0.00 C

ATOM 3107 CD2 TYR 394 41.297 42.694 33.484 1.00 0.00 C

ATOM 3108 CE1 TYR 394 40.720 45.223 32.612 1.00 0.00 C

ATOM 3109 CE2 TYR 394 40.821 43.630 34.371 1.00 0.00 C

ATOM 3110 CZ TYR 394 40.536 44.900 33.935 1.00 0.00 C

ATOM 3111 OH TYR 394 40.048 45.866 34.841 1.00 0.00 O

ATOM 3112 N THR 395 39.857 39.947 28.863 1.00 0.00 N

ATOM 3113 CA THR 395 39.784 38.961 27.786 1.00 0.00 C

ATOM 3114 C THR 395 38.351 39.200 27.300 1.00 0.00 C

ATOM 3115 O THR 395 37.583 39.497 28.209 1.00 0.00 O

ATOM 3116 CB THR 395 39.827 37.521 28.229 1.00 0.00 C

ATOM 3117 CG2 THR 395 41.164 37.218 28.923 1.00 0.00 C

ATOM 3118 OG1 THR 395 38.742 37.243 29.102 1.00 0.00 O

ATOM 3119 N ASN 396 37.928 39.073 25.974 1.00 0.00 N

ATOM 3120 CA ASN 396 36.561 39.456 25.545 1.00 0.00 C

ATOM 3121 C ASN 396 35.822 38.575 24.544 1.00 0.00 C

ATOM 3122 O ASN 396 36.411 38.000 23.631 1.00 0.00 O

ATOM 3123 CB ASN 396 36.481 40.901 25.033 1.00 0.00 C

ATOM 3124 CG ASN 396 36.613 41.800 26.255 1.00 0.00 C

ATOM 3125 ND2 ASN 396 35.539 42.578 26.555 1.00 0.00 N

ATOM 3126 OD1 ASN 396 37.648 41.824 26.918 1.00 0.00 O

ATOM 3127 N SER 397 34.460 38.492 24.693 1.00 0.00 N

ATOM 3128 CA SER 397 33.582 37.713 23.835 1.00 0.00 C

ATOM 3129 C SER 397 32.167 37.758 24.364 1.00 0.00 C

ATOM 3130 O SER 397 31.805 38.655 25.125 1.00 0.00 O

ATOM 3131 CB SER 397 33.985 36.231 23.751 1.00 0.00 C

ATOM 3132 OG SER 397 33.886 35.625 25.031 1.00 0.00 O

ATOM 3133 N SER 398 31.303 36.806 23.919 1.00 0.00 N

ATOM 3134 CA SER 398 29.960 36.711 24.437 1.00 0.00 C

ATOM 3135 C SER 398 30.129 36.383 25.897 1.00 0.00 C

ATOM 3136 O SER 398 30.753 35.384 26.244 1.00 0.00 O

ATOM 3137 CB SER 398 29.120 35.593 23.796 1.00 0.00 C

ATOM 3138 OG SER 398 27.819 35.573 24.366 1.00 0.00 O

ATOM 3139 N TRP 399 29.570 37.230 26.787 1.00 0.00 N

ATOM 3140 CA TRP 399 29.766 37.149 28.216 1.00 0.00 C

ATOM 3141 C TRP 399 29.102 35.976 28.858 1.00 0.00 C

ATOM 3142 O TRP 399 29.663 35.411 29.785 1.00 0.00 O

ATOM 3143 CB TRP 399 29.355 38.447 28.924 1.00 0.00 C

ATOM 3144 CG TRP 399 30.189 39.602 28.424 1.00 0.00 C

ATOM 3145 CD1 TRP 399 29.827 40.867 28.066 1.00 0.00 C

ATOM 3146 CD2 TRP 399 31.595 39.489 28.151 1.00 0.00 C

ATOM 3147 CE2 TRP 399 32.016 40.716 27.643 1.00 0.00 C

ATOM 3148 CE3 TRP 399 32.460 38.443 28.301 1.00 0.00 C

ATOM 3149 NE1 TRP 399 30.923 41.556 27.601 1.00 0.00 N

ATOM 3150 CZ2 TRP 399 33.317 40.916 27.277 1.00 0.00 C

ATOM 3151 CZ3 TRP 399 33.772 38.649 27.937 1.00 0.00 C

ATOM 3152 CH2 TRP 399 34.192 39.863 27.435 1.00 0.00 C

ATOM 3153 N THR 400 27.891 35.567 28.445 1.00 0.00 N

ATOM 3154 CA THR 400 27.297 34.474 29.171 1.00 0.00 C

ATOM 3155 C THR 400 28.137 33.246 29.028 1.00 0.00 C

ATOM 3156 O THR 400 28.439 32.575 30.012 1.00 0.00 O

ATOM 3157 CB THR 400 25.897 34.160 28.717 1.00 0.00 C

ATOM 3158 CG2 THR 400 25.911 33.732 27.240 1.00 0.00 C

ATOM 3159 OG1 THR 400 25.338 33.137 29.529 1.00 0.00 O

ATOM 3160 N LEU 401 28.550 32.906 27.798 1.00 0.00 N

ATOM 3161 CA LEU 401 29.331 31.719 27.644 1.00 0.00 C

ATOM 3162 C LEU 401 30.672 31.927 28.256 1.00 0.00 C

ATOM 3163 O LEU 401 31.261 31.006 28.816 1.00 0.00 O

ATOM 3164 CB LEU 401 29.460 31.250 26.182 1.00 0.00 C

ATOM 3165 CG LEU 401 29.906 32.322 25.174 1.00 0.00 C

ATOM 3166 CD1 LEU 401 31.375 32.729 25.371 1.00 0.00 C

ATOM 3167 CD2 LEU 401 29.589 31.873 23.740 1.00 0.00 C

ATOM 3168 N LYS 402 31.201 33.154 28.155 1.00 0.00 N

ATOM 3169 CA LYS 402 32.496 33.427 28.694 1.00 0.00 C

ATOM 3170 C LYS 402 32.441 33.257 30.181 1.00 0.00 C

ATOM 3171 O LYS 402 33.349 32.673 30.769 1.00 0.00 O

ATOM 3172 CB LYS 402 32.956 34.864 28.362 1.00 0.00 C

ATOM 3173 CG LYS 402 34.361 35.248 28.839 1.00 0.00 C

ATOM 3174 CD LYS 402 34.508 35.338 30.358 1.00 0.00 C

ATOM 3175 CE LYS 402 33.545 36.339 31.000 1.00 0.00 C

ATOM 3176 NZ LYS 402 33.537 36.163 32.469 1.00 0.00 N

ATOM 3177 N VAL 403 31.367 33.757 30.828 1.00 0.00 N

ATOM 3178 CA VAL 403 31.264 33.710 32.257 1.00 0.00 C

ATOM 3179 C VAL 403 31.182 32.281 32.686 1.00 0.00 C

ATOM 3180 O VAL 403 31.821 31.891 33.660 1.00 0.00 O

ATOM 3181 CB VAL 403 30.090 34.449 32.842 1.00 0.00 C

ATOM 3182 CG1 VAL 403 30.190 35.926 32.428 1.00 0.00 C

ATOM 3183 CG2 VAL 403 28.781 33.748 32.457 1.00 0.00 C

ATOM 3184 N CYS 404 30.398 31.450 31.971 1.00 0.00 N

ATOM 3185 CA CYS 404 30.268 30.080 32.380 1.00 0.00 C

ATOM 3186 C CYS 404 31.578 29.354 32.258 1.00 0.00 C

ATOM 3187 O CYS 404 31.933 28.576 33.141 1.00 0.00 O

ATOM 3188 CB CYS 404 29.131 29.308 31.674 1.00 0.00 C

ATOM 3189 SG CYS 404 29.072 29.489 29.870 1.00 0.00 S

ATOM 3190 N LEU 405 32.351 29.582 31.177 1.00 0.00 N

ATOM 3191 CA LEU 405 33.608 28.891 31.079 1.00 0.00 C

ATOM 3192 C LEU 405 34.508 29.325 32.190 1.00 0.00 C

ATOM 3193 O LEU 405 35.196 28.496 32.782 1.00 0.00 O

ATOM 3194 CB LEU 405 34.394 29.110 29.770 1.00 0.00 C

ATOM 3195 CG LEU 405 33.913 28.267 28.574 1.00 0.00 C

ATOM 3196 CD1 LEU 405 32.518 28.679 28.092 1.00 0.00 C

ATOM 3197 CD2 LEU 405 34.960 28.266 27.451 1.00 0.00 C

ATOM 3198 N LEU 406 34.540 30.636 32.497 1.00 0.00 N

ATOM 3199 CA LEU 406 35.426 31.129 33.515 1.00 0.00 C

ATOM 3200 C LEU 406 35.041 30.605 34.859 1.00 0.00 C

ATOM 3201 O LEU 406 35.903 30.204 35.637 1.00 0.00 O

ATOM 3202 CB LEU 406 35.462 32.663 33.624 1.00 0.00 C

ATOM 3203 CG LEU 406 36.151 33.356 32.435 1.00 0.00 C

ATOM 3204 CD1 LEU 406 36.273 34.870 32.672 1.00 0.00 C

ATOM 3205 CD2 LEU 406 37.503 32.697 32.117 1.00 0.00 C

ATOM 3206 N CYS 407 33.734 30.600 35.172 1.00 0.00 N

ATOM 3207 CA CYS 407 33.293 30.153 36.459 1.00 0.00 C

ATOM 3208 C CYS 407 33.652 28.710 36.616 1.00 0.00 C

ATOM 3209 O CYS 407 34.121 28.293 37.673 1.00 0.00 O

ATOM 3210 CB CYS 407 31.771 30.276 36.646 1.00 0.00 C

ATOM 3211 SG CYS 407 31.207 32.004 36.644 1.00 0.00 S

ATOM 3212 N ASP 408 33.442 27.907 35.555 1.00 0.00 N

ATOM 3213 CA ASP 408 33.718 26.500 35.619 1.00 0.00 C

ATOM 3214 C ASP 408 35.184 26.294 35.845 1.00 0.00 C

ATOM 3215 O ASP 408 35.585 25.460 36.655 1.00 0.00 O

ATOM 3216 CB ASP 408 33.347 25.762 34.320 1.00 0.00 C

ATOM 3217 CG ASP 408 31.829 25.751 34.199 1.00 0.00 C

ATOM 3218 OD1 ASP 408 31.153 26.097 35.205 1.00 0.00 O

ATOM 3219 OD2 ASP 408 31.324 25.394 33.101 1.00 0.00 O

ATOM 3220 N HIS 409 36.024 27.063 35.129 1.00 0.00 N

ATOM 3221 CA HIS 409 37.449 26.925 35.209 1.00 0.00 C

ATOM 3222 C HIS 409 37.912 27.288 36.586 1.00 0.00 C

ATOM 3223 O HIS 409 38.734 26.588 37.173 1.00 0.00 O

ATOM 3224 CB HIS 409 38.191 27.841 34.220 1.00 0.00 C

ATOM 3225 CG HIS 409 39.677 27.643 34.249 1.00 0.00 C

ATOM 3226 CD2 HIS 409 40.642 28.329 34.920 1.00 0.00 C

ATOM 3227 ND1 HIS 409 40.334 26.655 33.551 1.00 0.00 N

ATOM 3228 CE1 HIS 409 41.655 26.791 33.832 1.00 0.00 C

ATOM 3229 NE2 HIS 409 41.890 27.794 34.659 1.00 0.00 N

ATOM 3230 N PHE 410 37.386 28.398 37.140 1.00 0.00 N

ATOM 3231 CA PHE 410 37.801 28.856 38.432 1.00 0.00 C

ATOM 3232 C PHE 410 37.424 27.830 39.448 1.00 0.00 C

ATOM 3233 O PHE 410 38.211 27.501 40.333 1.00 0.00 O

ATOM 3234 CB PHE 410 37.136 30.181 38.842 1.00 0.00 C

ATOM 3235 CG PHE 410 37.653 30.538 40.194 1.00 0.00 C

ATOM 3236 CD1 PHE 410 38.873 31.158 40.335 1.00 0.00 C

ATOM 3237 CD2 PHE 410 36.917 30.255 41.321 1.00 0.00 C

ATOM 3238 CE1 PHE 410 39.352 31.488 41.581 1.00 0.00 C

ATOM 3239 CE2 PHE 410 37.390 30.583 42.569 1.00 0.00 C

ATOM 3240 CZ PHE 410 38.611 31.201 42.701 1.00 0.00 C

ATOM 3241 N CYS 411 36.200 27.286 39.341 1.00 0.00 N

ATOM 3242 CA CYS 411 35.741 26.329 40.301 1.00 0.00 C

ATOM 3243 C CYS 411 36.626 25.122 40.253 1.00 0.00 C

ATOM 3244 O CYS 411 36.997 24.575 41.291 1.00 0.00 O

ATOM 3245 CB CYS 411 34.294 25.883 40.039 1.00 0.00 C

ATOM 3246 SG CYS 411 33.127 27.265 40.211 1.00 0.00 S

ATOM 3247 N ARG 412 36.999 24.681 39.038 1.00 0.00 N

ATOM 3248 CA ARG 412 37.821 23.516 38.880 1.00 0.00 C

ATOM 3249 C ARG 412 39.153 23.784 39.504 1.00 0.00 C

ATOM 3250 O ARG 412 39.731 22.912 40.152 1.00 0.00 O

ATOM 3251 CB ARG 412 38.031 23.154 37.402 1.00 0.00 C

ATOM 3252 CG ARG 412 38.899 21.917 37.180 1.00 0.00 C

ATOM 3253 CD ARG 412 38.897 21.439 35.727 1.00 0.00 C

ATOM 3254 NE ARG 412 39.171 22.624 34.865 1.00 0.00 N

ATOM 3255 CZ ARG 412 38.140 23.403 34.426 1.00 0.00 C

ATOM 3256 NH1 ARG 412 36.855 23.109 34.784 1.00 0.00 N

ATOM 3257 NH2 ARG 412 38.392 24.478 33.624 1.00 0.00 N

ATOM 3258 N LEU 413 39.671 25.014 39.324 1.00 0.00 N

ATOM 3259 CA LEU 413 40.949 25.394 39.850 1.00 0.00 C

ATOM 3260 C LEU 413 40.900 25.325 41.344 1.00 0.00 C

ATOM 3261 O LEU 413 41.819 24.809 41.979 1.00 0.00 O

ATOM 3262 CB LEU 413 41.332 26.836 39.472 1.00 0.00 C

ATOM 3263 CG LEU 413 42.700 27.279 40.021 1.00 0.00 C

ATOM 3264 CD1 LEU 413 43.840 26.436 39.432 1.00 0.00 C

ATOM 3265 CD2 LEU 413 42.915 28.788 39.828 1.00 0.00 C

ATOM 3266 N LEU 414 39.807 25.837 41.940 1.00 0.00 N

ATOM 3267 CA LEU 414 39.688 25.883 43.367 1.00 0.00 C

ATOM 3268 C LEU 414 39.652 24.474 43.863 1.00 0.00 C

ATOM 3269 O LEU 414 40.279 24.149 44.870 1.00 0.00 O

ATOM 3270 CB LEU 414 38.396 26.607 43.817 1.00 0.00 C

ATOM 3271 CG LEU 414 38.260 26.927 45.327 1.00 0.00 C

ATOM 3272 CD1 LEU 414 36.928 27.643 45.603 1.00 0.00 C

ATOM 3273 CD2 LEU 414 38.437 25.699 46.235 1.00 0.00 C

ATOM 3274 N GLY 415 38.915 23.595 43.160 1.00 0.00 N

ATOM 3275 CA GLY 415 38.778 22.243 43.608 1.00 0.00 C

ATOM 3276 C GLY 415 40.111 21.560 43.629 1.00 0.00 C

ATOM 3277 O GLY 415 40.431 20.873 44.599 1.00 0.00 O

ATOM 3278 N LEU 416 40.939 21.715 42.574 1.00 0.00 N

ATOM 3279 CA LEU 416 42.175 20.988 42.635 1.00 0.00 C

ATOM 3280 C LEU 416 43.038 21.522 43.729 1.00 0.00 C

ATOM 3281 O LEU 416 43.758 20.762 44.373 1.00 0.00 O

ATOM 3282 CB LEU 416 43.004 20.837 41.333 1.00 0.00 C

ATOM 3283 CG LEU 416 43.579 22.089 40.648 1.00 0.00 C

ATOM 3284 CD1 LEU 416 42.469 22.896 39.983 1.00 0.00 C

ATOM 3285 CD2 LEU 416 44.483 22.919 41.573 1.00 0.00 C

ATOM 3286 N MET 417 42.999 22.844 43.973 1.00 0.00 N

ATOM 3287 CA MET 417 43.840 23.403 44.992 1.00 0.00 C

ATOM 3288 C MET 417 43.490 22.808 46.315 1.00 0.00 C

ATOM 3289 O MET 417 44.373 22.395 47.062 1.00 0.00 O

ATOM 3290 CB MET 417 43.674 24.924 45.135 1.00 0.00 C

ATOM 3291 CG MET 417 44.348 25.710 44.016 1.00 0.00 C

ATOM 3292 SD MET 417 46.153 25.765 44.177 1.00 0.00 S

ATOM 3293 CE MET 417 46.161 27.184 45.309 1.00 0.00 C

ATOM 3294 N GLU 418 42.192 22.725 46.651 1.00 0.00 N

ATOM 3295 CA GLU 418 41.888 22.219 47.953 1.00 0.00 C

ATOM 3296 C GLU 418 42.234 20.768 48.063 1.00 0.00 C

ATOM 3297 O GLU 418 42.706 20.324 49.108 1.00 0.00 O

ATOM 3298 CB GLU 418 40.463 22.541 48.435 1.00 0.00 C

ATOM 3299 CG GLU 418 39.331 22.244 47.466 1.00 0.00 C

ATOM 3300 CD GLU 418 38.176 23.084 47.984 1.00 0.00 C

ATOM 3301 OE1 GLU 418 38.450 23.993 48.814 1.00 0.00 O

ATOM 3302 OE2 GLU 418 37.015 22.840 47.564 1.00 0.00 O

ATOM 3303 N ARG 419 42.029 19.979 46.993 1.00 0.00 N

ATOM 3304 CA ARG 419 42.373 18.591 47.101 1.00 0.00 C

ATOM 3305 C ARG 419 43.855 18.475 47.314 1.00 0.00 C

ATOM 3306 O ARG 419 44.313 17.695 48.147 1.00 0.00 O

ATOM 3307 CB ARG 419 42.001 17.772 45.849 1.00 0.00 C

ATOM 3308 CG ARG 419 42.697 18.219 44.561 1.00 0.00 C

ATOM 3309 CD ARG 419 42.304 17.383 43.341 1.00 0.00 C

ATOM 3310 NE ARG 419 43.045 17.916 42.165 1.00 0.00 N

ATOM 3311 CZ ARG 419 43.085 17.197 41.005 1.00 0.00 C

ATOM 3312 NH1 ARG 419 42.433 16.001 40.923 1.00 0.00 N

ATOM 3313 NH2 ARG 419 43.776 17.672 39.928 1.00 0.00 N

ATOM 3314 N GLU 420 44.637 19.269 46.556 1.00 0.00 N

ATOM 3315 CA GLU 420 46.074 19.271 46.575 1.00 0.00 C

ATOM 3316 C GLU 420 46.584 19.829 47.868 1.00 0.00 C

ATOM 3317 O GLU 420 47.662 19.454 48.325 1.00 0.00 O

ATOM 3318 CB GLU 420 46.674 20.092 45.427 1.00 0.00 C

ATOM 3319 CG GLU 420 46.412 19.492 44.044 1.00 0.00 C

ATOM 3320 CD GLU 420 47.263 18.237 43.907 1.00 0.00 C

ATOM 3321 OE1 GLU 420 47.997 17.911 44.878 1.00 0.00 O

ATOM 3322 OE2 GLU 420 47.190 17.588 42.830 1.00 0.00 O

ATOM 3323 N GLY 421 45.843 20.761 48.490 1.00 0.00 N

ATOM 3324 CA GLY 421 46.323 21.323 49.716 1.00 0.00 C

ATOM 3325 C GLY 421 47.127 22.544 49.397 1.00 0.00 C

ATOM 3326 O GLY 421 47.920 22.996 50.222 1.00 0.00 O

ATOM 3327 N TYR 422 46.958 23.109 48.182 1.00 0.00 N

ATOM 3328 CA TYR 422 47.691 24.300 47.857 1.00 0.00 C

ATOM 3329 C TYR 422 46.921 25.485 48.345 1.00 0.00 C

ATOM 3330 O TYR 422 45.721 25.612 48.107 1.00 0.00 O

ATOM 3331 CB TYR 422 47.941 24.544 46.360 1.00 0.00 C

ATOM 3332 CG TYR 422 48.824 23.468 45.839 1.00 0.00 C

ATOM 3333 CD1 TYR 422 50.148 23.419 46.197 1.00 0.00 C

ATOM 3334 CD2 TYR 422 48.325 22.491 45.015 1.00 0.00 C

ATOM 3335 CE1 TYR 422 50.968 22.423 45.724 1.00 0.00 C

ATOM 3336 CE2 TYR 422 49.138 21.491 44.535 1.00 0.00 C

ATOM 3337 CZ TYR 422 50.465 21.456 44.889 1.00 0.00 C

ATOM 3338 OH TYR 422 51.303 20.432 44.400 1.00 0.00 O

ATOM 3339 N ASN 423 47.609 26.344 49.122 1.00 0.00 N

ATOM 3340 CA ASN 423 47.070 27.552 49.679 1.00 0.00 C

ATOM 3341 C ASN 423 47.049 28.707 48.713 1.00 0.00 C

ATOM 3342 O ASN 423 46.164 29.557 48.812 1.00 0.00 O

ATOM 3343 CB ASN 423 47.831 28.014 50.931 1.00 0.00 C

ATOM 3344 CG ASN 423 47.605 26.975 52.022 1.00 0.00 C

ATOM 3345 ND2 ASN 423 46.319 26.776 52.416 1.00 0.00 N

ATOM 3346 OD1 ASN 423 48.549 26.355 52.510 1.00 0.00 O

ATOM 3347 N VAL 424 48.028 28.816 47.782 1.00 0.00 N

ATOM 3348 CA VAL 424 48.053 30.027 46.999 1.00 0.00 C

ATOM 3349 C VAL 424 48.389 29.767 45.558 1.00 0.00 C

ATOM 3350 O VAL 424 49.123 28.838 45.225 1.00 0.00 O

ATOM 3351 CB VAL 424 49.067 31.012 47.508 1.00 0.00 C

ATOM 3352 CG1 VAL 424 49.068 32.251 46.597 1.00 0.00 C

ATOM 3353 CG2 VAL 424 48.750 31.319 48.981 1.00 0.00 C

ATOM 3354 N CYS 425 47.828 30.610 44.660 1.00 0.00 N

ATOM 3355 CA CYS 425 48.115 30.573 43.251 1.00 0.00 C

ATOM 3356 C CYS 425 48.325 31.975 42.778 1.00 0.00 C

ATOM 3357 O CYS 425 47.624 32.895 43.201 1.00 0.00 O

ATOM 3358 CB CYS 425 46.992 30.010 42.368 1.00 0.00 C

ATOM 3359 SG CYS 425 46.995 28.203 42.303 1.00 0.00 S

ATOM 3360 N GLU 426 49.319 32.165 41.881 1.00 0.00 N

ATOM 3361 CA GLU 426 49.593 33.460 41.308 1.00 0.00 C

ATOM 3362 C GLU 426 50.065 33.256 39.907 1.00 0.00 C

ATOM 3363 O GLU 426 50.771 32.296 39.619 1.00 0.00 O

ATOM 3364 CB GLU 426 50.781 34.219 41.919 1.00 0.00 C

ATOM 3365 CG GLU 426 50.546 34.847 43.280 1.00 0.00 C

ATOM 3366 CD GLU 426 51.817 35.594 43.664 1.00 0.00 C

ATOM 3367 OE1 GLU 426 52.103 36.644 43.028 1.00 0.00 O

ATOM 3368 OE2 GLU 426 52.520 35.122 44.596 1.00 0.00 O

ATOM 3369 N PRO 427 49.707 34.131 39.016 1.00 0.00 N

ATOM 3370 CA PRO 427 50.209 34.010 37.675 1.00 0.00 C

ATOM 3371 C PRO 427 51.590 34.581 37.542 1.00 0.00 C

ATOM 3372 O PRO 427 51.966 35.434 38.346 1.00 0.00 O

ATOM 3373 CB PRO 427 49.179 34.692 36.768 1.00 0.00 C

ATOM 3374 CG PRO 427 48.286 35.499 37.726 1.00 0.00 C

ATOM 3375 CD PRO 427 48.382 34.725 39.047 1.00 0.00 C

ATOM 3376 N LYS 428 52.364 34.094 36.546 1.00 0.00 N

ATOM 3377 CA LYS 428 53.649 34.645 36.210 1.00 0.00 C

ATOM 3378 C LYS 428 53.316 35.727 35.233 1.00 0.00 C

ATOM 3379 O LYS 428 52.192 35.762 34.743 1.00 0.00 O

ATOM 3380 CB LYS 428 54.585 33.652 35.500 1.00 0.00 C

ATOM 3381 CG LYS 428 55.023 32.485 36.388 1.00 0.00 C

ATOM 3382 CD LYS 428 55.792 32.922 37.636 1.00 0.00 C

ATOM 3383 CE LYS 428 57.182 33.484 37.333 1.00 0.00 C

ATOM 3384 NZ LYS 428 57.062 34.829 36.726 1.00 0.00 N

ATOM 3385 N ALA 429 54.233 36.661 34.903 1.00 0.00 N

ATOM 3386 CA ALA 429 53.682 37.669 34.039 1.00 0.00 C

ATOM 3387 C ALA 429 54.002 37.504 32.569 1.00 0.00 C

ATOM 3388 O ALA 429 54.489 38.465 31.978 1.00 0.00 O

ATOM 3389 CB ALA 429 54.134 39.089 34.424 1.00 0.00 C

ATOM 3390 N PRO 430 53.760 36.390 31.910 1.00 0.00 N

ATOM 3391 CA PRO 430 53.872 36.421 30.463 1.00 0.00 C

ATOM 3392 C PRO 430 52.473 36.571 29.909 1.00 0.00 C

ATOM 3393 O PRO 430 51.544 36.501 30.706 1.00 0.00 O

ATOM 3394 CB PRO 430 54.480 35.087 30.034 1.00 0.00 C

ATOM 3395 CG PRO 430 55.168 34.558 31.297 1.00 0.00 C

ATOM 3396 CD PRO 430 54.334 35.157 32.436 1.00 0.00 C

ATOM 3397 N GLU 431 52.304 36.787 28.579 1.00 0.00 N

ATOM 3398 CA GLU 431 51.039 36.725 27.853 1.00 0.00 C

ATOM 3399 C GLU 431 50.464 38.074 27.394 1.00 0.00 C

ATOM 3400 O GLU 431 50.235 38.934 28.243 1.00 0.00 O

ATOM 3401 CB GLU 431 49.939 35.913 28.574 1.00 0.00 C

ATOM 3402 CG GLU 431 50.105 34.395 28.422 1.00 0.00 C

ATOM 3403 CD GLU 431 51.439 33.953 29.015 1.00 0.00 C

ATOM 3404 OE1 GLU 431 51.595 34.000 30.264 1.00 0.00 O

ATOM 3405 OE2 GLU 431 52.324 33.550 28.214 1.00 0.00 O

ATOM 3406 N GLY 432 50.188 38.269 26.044 1.00 0.00 N

ATOM 3407 CA GLY 432 49.555 39.466 25.460 1.00 0.00 C

ATOM 3408 C GLY 432 49.504 39.466 23.917 1.00 0.00 C

ATOM 3409 O GLY 432 50.492 39.784 23.257 1.00 0.00 O

ATOM 3410 N VAL 433 48.325 39.128 23.315 1.00 0.00 N

ATOM 3411 CA VAL 433 48.001 38.971 21.892 1.00 0.00 C

ATOM 3412 C VAL 433 47.673 40.160 20.985 1.00 0.00 C

ATOM 3413 O VAL 433 48.084 40.162 19.824 1.00 0.00 O

ATOM 3414 CB VAL 433 46.876 37.997 21.700 1.00 0.00 C

ATOM 3415 CG1 VAL 433 46.560 37.896 20.198 1.00 0.00 C

ATOM 3416 CG2 VAL 433 47.274 36.660 22.349 1.00 0.00 C

ATOM 3417 N GLU 434 46.921 41.193 21.431 1.00 0.00 N

ATOM 3418 CA GLU 434 46.336 42.104 20.463 1.00 0.00 C

ATOM 3419 C GLU 434 47.309 43.042 19.827 1.00 0.00 C

ATOM 3420 O GLU 434 47.503 44.167 20.272 1.00 0.00 O

ATOM 3421 CB GLU 434 45.197 42.961 21.048 1.00 0.00 C

ATOM 3422 CG GLU 434 44.562 43.922 20.039 1.00 0.00 C

ATOM 3423 CD GLU 434 43.397 44.621 20.726 1.00 0.00 C

ATOM 3424 OE1 GLU 434 42.519 43.901 21.271 1.00 0.00 O

ATOM 3425 OE2 GLU 434 43.368 45.881 20.717 1.00 0.00 O

ATOM 3426 N THR 435 47.890 42.637 18.690 1.00 0.00 N

ATOM 3427 CA THR 435 48.793 43.524 18.026 1.00 0.00 C

ATOM 3428 C THR 435 48.327 43.604 16.613 1.00 0.00 C

ATOM 3429 O THR 435 48.276 44.675 16.011 1.00 0.00 O

ATOM 3430 CB THR 435 50.198 43.002 18.014 1.00 0.00 C

ATOM 3431 CG2 THR 435 51.101 44.032 17.315 1.00 0.00 C

ATOM 3432 OG1 THR 435 50.645 42.791 19.346 1.00 0.00 O

ATOM 3433 N ARG 436 47.955 42.437 16.066 1.00 0.00 N

ATOM 3434 CA ARG 436 47.510 42.303 14.712 1.00 0.00 C

ATOM 3435 C ARG 436 46.193 42.992 14.460 1.00 0.00 C

ATOM 3436 O ARG 436 45.985 43.449 13.338 1.00 0.00 O

ATOM 3437 CB ARG 436 47.451 40.836 14.222 1.00 0.00 C

ATOM 3438 CG ARG 436 46.577 39.880 15.040 1.00 0.00 C

ATOM 3439 CD ARG 436 46.579 38.443 14.512 1.00 0.00 C

ATOM 3440 NE ARG 436 45.695 37.628 15.394 1.00 0.00 N

ATOM 3441 CZ ARG 436 46.218 36.936 16.449 1.00 0.00 C

ATOM 3442 NH1 ARG 436 47.556 36.993 16.711 1.00 0.00 N

ATOM 3443 NH2 ARG 436 45.403 36.180 17.241 1.00 0.00 N

ATOM 3444 N PRO 437 45.276 43.112 15.389 1.00 0.00 N

ATOM 3445 CA PRO 437 44.013 43.707 15.024 1.00 0.00 C

ATOM 3446 C PRO 437 44.036 45.182 14.782 1.00 0.00 C

ATOM 3447 O PRO 437 44.961 45.858 15.225 1.00 0.00 O

ATOM 3448 CB PRO 437 43.022 43.310 16.116 1.00 0.00 C

ATOM 3449 CG PRO 437 43.588 41.989 16.658 1.00 0.00 C

ATOM 3450 CD PRO 437 45.101 42.113 16.434 1.00 0.00 C

ATOM 3451 N LEU 438 43.009 45.673 14.059 1.00 0.00 N

ATOM 3452 CA LEU 438 42.783 47.051 13.724 1.00 0.00 C

ATOM 3453 C LEU 438 42.460 47.791 14.982 1.00 0.00 C

ATOM 3454 O LEU 438 42.654 49.001 15.074 1.00 0.00 O

ATOM 3455 CB LEU 438 41.615 47.255 12.742 1.00 0.00 C

ATOM 3456 CG LEU 438 41.899 46.706 11.332 1.00 0.00 C

ATOM 3457 CD1 LEU 438 40.709 46.931 10.386 1.00 0.00 C

ATOM 3458 CD2 LEU 438 43.215 47.269 10.773 1.00 0.00 C

ATOM 3459 N LEU 439 41.946 47.062 15.985 1.00 0.00 N

ATOM 3460 CA LEU 439 41.474 47.621 17.217 1.00 0.00 C

ATOM 3461 C LEU 439 42.579 48.361 17.911 1.00 0.00 C

ATOM 3462 O LEU 439 42.313 49.338 18.611 1.00 0.00 O

ATOM 3463 CB LEU 439 40.913 46.547 18.166 1.00 0.00 C

ATOM 3464 CG LEU 439 39.691 45.810 17.577 1.00 0.00 C

ATOM 3465 CD1 LEU 439 40.077 44.973 16.346 1.00 0.00 C

ATOM 3466 CD2 LEU 439 38.950 44.992 18.645 1.00 0.00 C

ATOM 3467 N ASP 440 43.846 47.933 17.740 1.00 0.00 N

ATOM 3468 CA ASP 440 44.921 48.568 18.451 1.00 0.00 C

ATOM 3469 C ASP 440 44.926 50.040 18.165 1.00 0.00 C

ATOM 3470 O ASP 440 45.008 50.844 19.091 1.00 0.00 O

ATOM 3471 CB ASP 440 46.310 47.994 18.101 1.00 0.00 C

ATOM 3472 CG ASP 440 46.612 48.206 16.622 1.00 0.00 C

ATOM 3473 OD1 ASP 440 45.652 48.228 15.807 1.00 0.00 O

ATOM 3474 OD2 ASP 440 47.820 48.339 16.286 1.00 0.00 O

ATOM 3475 N PHE 441 44.812 50.455 16.891 1.00 0.00 N

ATOM 3476 CA PHE 441 44.755 51.866 16.659 1.00 0.00 C

ATOM 3477 C PHE 441 43.330 52.198 16.899 1.00 0.00 C

ATOM 3478 O PHE 441 42.444 51.623 16.272 1.00 0.00 O

ATOM 3479 CB PHE 441 45.107 52.274 15.214 1.00 0.00 C

ATOM 3480 CG PHE 441 45.058 53.764 15.084 1.00 0.00 C

ATOM 3481 CD1 PHE 441 46.142 54.538 15.436 1.00 0.00 C

ATOM 3482 CD2 PHE 441 43.933 54.390 14.595 1.00 0.00 C

ATOM 3483 CE1 PHE 441 46.102 55.908 15.311 1.00 0.00 C

ATOM 3484 CE2 PHE 441 43.887 55.759 14.468 1.00 0.00 C

ATOM 3485 CZ PHE 441 44.973 56.522 14.826 1.00 0.00 C

ATOM 3486 N GLY 442 43.059 53.114 17.844 1.00 0.00 N

ATOM 3487 CA GLY 442 41.684 53.393 18.109 1.00 0.00 C

ATOM 3488 C GLY 442 41.610 54.518 19.077 1.00 0.00 C

ATOM 3489 O GLY 442 42.619 55.095 19.476 1.00 0.00 O

ATOM 3490 N ALA 443 40.369 54.844 19.470 1.00 0.00 N

ATOM 3491 CA ALA 443 40.085 55.899 20.386 1.00 0.00 C

ATOM 3492 C ALA 443 40.375 55.395 21.750 1.00 0.00 C

ATOM 3493 O ALA 443 40.868 54.279 21.910 1.00 0.00 O

ATOM 3494 CB ALA 443 38.624 56.377 20.348 1.00 0.00 C

ATOM 3495 N GLY 444 40.125 56.244 22.767 1.00 0.00 N

ATOM 3496 CA GLY 444 40.423 55.813 24.093 1.00 0.00 C

ATOM 3497 C GLY 444 39.637 54.578 24.324 1.00 0.00 C

ATOM 3498 O GLY 444 40.239 53.539 24.588 1.00 0.00 O

ATOM 3499 N TYR 445 38.294 54.621 24.137 1.00 0.00 N

ATOM 3500 CA TYR 445 37.591 53.390 24.355 1.00 0.00 C

ATOM 3501 C TYR 445 37.867 53.000 25.775 1.00 0.00 C

ATOM 3502 O TYR 445 38.317 53.812 26.582 1.00 0.00 O

ATOM 3503 CB TYR 445 38.065 52.289 23.379 1.00 0.00 C

ATOM 3504 CG TYR 445 37.149 51.114 23.426 1.00 0.00 C

ATOM 3505 CD1 TYR 445 35.895 51.185 22.859 1.00 0.00 C

ATOM 3506 CD2 TYR 445 37.552 49.932 24.001 1.00 0.00 C

ATOM 3507 CE1 TYR 445 35.047 50.103 22.888 1.00 0.00 C

ATOM 3508 CE2 TYR 445 36.709 48.847 24.032 1.00 0.00 C

ATOM 3509 CZ TYR 445 35.455 48.932 23.478 1.00 0.00 C

ATOM 3510 OH TYR 445 34.588 47.820 23.509 1.00 0.00 O

ATOM 3511 N VAL 446 37.543 51.760 26.153 1.00 0.00 N

ATOM 3512 CA VAL 446 37.939 51.355 27.466 1.00 0.00 C

ATOM 3513 C VAL 446 39.434 51.415 27.575 1.00 0.00 C

ATOM 3514 O VAL 446 39.989 51.315 28.667 1.00 0.00 O

ATOM 3515 CB VAL 446 37.274 50.111 27.994 1.00 0.00 C

ATOM 3516 CG1 VAL 446 38.056 49.467 29.152 1.00 0.00 C

ATOM 3517 CG2 VAL 446 35.952 50.643 28.574 1.00 0.00 C

ATOM 3518 N GLN 447 40.157 51.507 26.441 1.00 0.00 N

ATOM 3519 CA GLN 447 41.554 51.774 26.622 1.00 0.00 C

ATOM 3520 C GLN 447 41.617 53.078 27.352 1.00 0.00 C

ATOM 3521 O GLN 447 41.334 54.136 26.796 1.00 0.00 O

ATOM 3522 CB GLN 447 42.330 51.931 25.303 1.00 0.00 C

ATOM 3523 CG GLN 447 42.320 50.677 24.429 1.00 0.00 C

ATOM 3524 CD GLN 447 43.092 50.989 23.156 1.00 0.00 C

ATOM 3525 NE2 GLN 447 43.423 52.292 22.951 1.00 0.00 N

ATOM 3526 OE1 GLN 447 43.392 50.099 22.361 1.00 0.00 O

ATOM 3527 N ARG 448 41.966 53.025 28.650 1.00 0.00 N

ATOM 3528 CA ARG 448 41.986 54.221 29.430 1.00 0.00 C

ATOM 3529 C ARG 448 43.289 54.335 30.137 1.00 0.00 C

ATOM 3530 O ARG 448 43.596 53.541 31.024 1.00 0.00 O

ATOM 3531 CB ARG 448 40.904 54.268 30.520 1.00 0.00 C

ATOM 3532 CG ARG 448 40.944 55.556 31.345 1.00 0.00 C

ATOM 3533 CD ARG 448 39.939 55.590 32.499 1.00 0.00 C

ATOM 3534 NE ARG 448 40.380 54.578 33.501 1.00 0.00 N

ATOM 3535 CZ ARG 448 41.307 54.906 34.448 1.00 0.00 C

ATOM 3536 NH1 ARG 448 41.813 56.173 34.500 1.00 0.00 N

ATOM 3537 NH2 ARG 448 41.734 53.965 35.340 1.00 0.00 N

ATOM 3538 N ALA 449 44.077 55.352 29.742 1.00 0.00 N

ATOM 3539 CA ALA 449 45.325 55.665 30.367 1.00 0.00 C

ATOM 3540 C ALA 449 46.134 56.370 29.339 1.00 0.00 C

ATOM 3541 O ALA 449 45.866 56.258 28.144 1.00 0.00 O

ATOM 3542 CB ALA 449 46.162 54.450 30.807 1.00 0.00 C

ATOM 3543 N LEU 450 47.146 57.133 29.784 1.00 0.00 N

ATOM 3544 CA LEU 450 47.997 57.756 28.823 1.00 0.00 C

ATOM 3545 C LEU 450 48.878 56.652 28.367 1.00 0.00 C

ATOM 3546 O LEU 450 49.291 55.819 29.172 1.00 0.00 O

ATOM 3547 CB LEU 450 48.865 58.890 29.402 1.00 0.00 C

ATOM 3548 CG LEU 450 49.694 59.656 28.350 1.00 0.00 C

ATOM 3549 CD1 LEU 450 50.817 58.791 27.757 1.00 0.00 C

ATOM 3550 CD2 LEU 450 48.785 60.271 27.273 1.00 0.00 C

ATOM 3551 N ASP 451 49.173 56.603 27.058 1.00 0.00 N

ATOM 3552 CA ASP 451 49.927 55.505 26.548 1.00 0.00 C

ATOM 3553 C ASP 451 51.343 55.892 26.281 1.00 0.00 C

ATOM 3554 O ASP 451 51.812 55.811 25.149 1.00 0.00 O

ATOM 3555 CB ASP 451 49.325 54.955 25.238 1.00 0.00 C

ATOM 3556 CG ASP 451 49.267 56.060 24.184 1.00 0.00 C

ATOM 3557 OD1 ASP 451 49.489 57.248 24.540 1.00 0.00 O

ATOM 3558 OD2 ASP 451 49.004 55.721 23.000 1.00 0.00 O

ATOM 3559 N SER 452 52.097 56.291 27.318 1.00 0.00 N

ATOM 3560 CA SER 452 53.459 56.601 27.011 1.00 0.00 C

ATOM 3561 C SER 452 54.320 55.910 28.011 1.00 0.00 C

ATOM 3562 O SER 452 54.095 56.006 29.215 1.00 0.00 O

ATOM 3563 CB SER 452 53.788 58.102 27.091 1.00 0.00 C

ATOM 3564 OG SER 452 53.065 58.812 26.096 1.00 0.00 O

ATOM 3565 N MET 453 55.342 55.185 27.526 1.00 0.00 N

ATOM 3566 CA MET 453 56.246 54.540 28.428 1.00 0.00 C

ATOM 3567 C MET 453 57.543 55.265 28.304 1.00 0.00 C

ATOM 3568 O MET 453 57.901 55.733 27.224 1.00 0.00 O

ATOM 3569 CB MET 453 56.521 53.064 28.095 1.00 0.00 C

ATOM 3570 CG MET 453 55.346 52.128 28.381 1.00 0.00 C

ATOM 3571 SD MET 453 54.993 51.885 30.149 1.00 0.00 S

ATOM 3572 CE MET 453 54.231 53.514 30.395 1.00 0.00 C

ATOM 3573 N PRO 454 58.252 55.392 29.391 1.00 0.00 N

ATOM 3574 CA PRO 454 59.524 56.045 29.342 1.00 0.00 C

ATOM 3575 C PRO 454 60.383 55.221 28.459 1.00 0.00 C

ATOM 3576 O PRO 454 61.270 55.755 27.797 1.00 0.00 O

ATOM 3577 CB PRO 454 59.984 56.159 30.791 1.00 0.00 C

ATOM 3578 CG PRO 454 58.659 56.225 31.578 1.00 0.00 C

ATOM 3579 CD PRO 454 57.654 55.442 30.714 1.00 0.00 C

ATOM 3580 N ARG 455 60.118 53.909 28.418 1.00 0.00 N

ATOM 3581 CA ARG 455 60.854 53.091 27.517 1.00 0.00 C

ATOM 3582 C ARG 455 60.238 53.421 26.209 1.00 0.00 C

ATOM 3583 O ARG 455 60.428 54.518 25.684 1.00 0.00 O

ATOM 3584 CB ARG 455 60.675 51.583 27.765 1.00 0.00 C

ATOM 3585 CG ARG 455 61.269 51.114 29.095 1.00 0.00 C

ATOM 3586 CD ARG 455 61.211 49.598 29.296 1.00 0.00 C

ATOM 3587 NE ARG 455 61.821 49.302 30.624 1.00 0.00 N

ATOM 3588 CZ ARG 455 63.170 49.125 30.733 1.00 0.00 C

ATOM 3589 NH1 ARG 455 63.963 49.217 29.625 1.00 0.00 N

ATOM 3590 NH2 ARG 455 63.729 48.860 31.950 1.00 0.00 N

ATOM 3591 N GLN 456 59.499 52.472 25.626 1.00 0.00 N

ATOM 3592 CA GLN 456 58.851 52.805 24.400 1.00 0.00 C

ATOM 3593 C GLN 456 57.581 52.030 24.351 1.00 0.00 C

ATOM 3594 O GLN 456 57.420 51.016 25.027 1.00 0.00 O

ATOM 3595 CB GLN 456 59.692 52.452 23.157 1.00 0.00 C

ATOM 3596 CG GLN 456 59.036 52.809 21.819 1.00 0.00 C

ATOM 3597 CD GLN 456 58.163 51.643 21.380 1.00 0.00 C

ATOM 3598 NE2 GLN 456 58.327 50.474 22.055 1.00 0.00 N

ATOM 3599 OE1 GLN 456 57.355 51.768 20.461 1.00 0.00 O

ATOM 3600 N GLY 457 56.616 52.527 23.568 1.00 0.00 N

ATOM 3601 CA GLY 457 55.408 51.784 23.399 1.00 0.00 C

ATOM 3602 C GLY 457 54.324 52.421 24.192 1.00 0.00 C

ATOM 3603 O GLY 457 54.424 52.668 25.394 1.00 0.00 O

ATOM 3604 N PRO 458 53.291 52.713 23.460 1.00 0.00 N

ATOM 3605 CA PRO 458 52.116 53.258 24.076 1.00 0.00 C

ATOM 3606 C PRO 458 51.277 52.155 24.637 1.00 0.00 C

ATOM 3607 O PRO 458 51.324 51.044 24.111 1.00 0.00 O

ATOM 3608 CB PRO 458 51.388 54.043 22.987 1.00 0.00 C

ATOM 3609 CG PRO 458 52.500 54.423 21.998 1.00 0.00 C

ATOM 3610 CD PRO 458 53.520 53.284 22.141 1.00 0.00 C

ATOM 3611 N ARG 459 50.496 52.425 25.700 1.00 0.00 N

ATOM 3612 CA ARG 459 49.626 51.395 26.184 1.00 0.00 C

ATOM 3613 C ARG 459 48.294 52.014 26.489 1.00 0.00 C

ATOM 3614 O ARG 459 48.223 53.144 26.974 1.00 0.00 O

ATOM 3615 CB ARG 459 50.099 50.677 27.462 1.00 0.00 C

ATOM 3616 CG ARG 459 51.225 49.668 27.224 1.00 0.00 C

ATOM 3617 CD ARG 459 52.616 50.288 27.099 1.00 0.00 C

ATOM 3618 NE ARG 459 53.277 50.145 28.427 1.00 0.00 N

ATOM 3619 CZ ARG 459 53.966 49.005 28.729 1.00 0.00 C

ATOM 3620 NH1 ARG 459 54.062 48.000 27.811 1.00 0.00 N

ATOM 3621 NH2 ARG 459 54.564 48.870 29.949 1.00 0.00 N

ATOM 3622 N GLU 460 47.199 51.278 26.181 1.00 0.00 N

ATOM 3623 CA GLU 460 45.857 51.743 26.435 1.00 0.00 C

ATOM 3624 C GLU 460 44.989 50.541 26.736 1.00 0.00 C

ATOM 3625 O GLU 460 45.131 49.515 26.071 1.00 0.00 O

ATOM 3626 CB GLU 460 45.230 52.437 25.215 1.00 0.00 C

ATOM 3627 CG GLU 460 45.911 53.748 24.821 1.00 0.00 C

ATOM 3628 CD GLU 460 45.452 54.828 25.789 1.00 0.00 C

ATOM 3629 OE1 GLU 460 44.670 54.500 26.721 1.00 0.00 O

ATOM 3630 OE2 GLU 460 45.876 55.999 25.602 1.00 0.00 O

ATOM 3631 N PRO 461 44.123 50.607 27.738 1.00 0.00 N

ATOM 3632 CA PRO 461 43.286 49.457 28.028 1.00 0.00 C

ATOM 3633 C PRO 461 42.202 48.984 27.156 1.00 0.00 C

ATOM 3634 O PRO 461 41.046 49.201 27.504 1.00 0.00 O

ATOM 3635 CB PRO 461 42.882 49.556 29.504 1.00 0.00 C

ATOM 3636 CG PRO 461 43.429 50.914 29.976 1.00 0.00 C

ATOM 3637 CD PRO 461 44.545 51.247 28.973 1.00 0.00 C

ATOM 3638 N TRP 462 42.529 48.351 26.026 1.00 0.00 N

ATOM 3639 CA TRP 462 41.505 47.632 25.355 1.00 0.00 C

ATOM 3640 C TRP 462 41.471 46.427 26.172 1.00 0.00 C

ATOM 3641 O TRP 462 40.400 45.891 26.455 1.00 0.00 O

ATOM 3642 CB TRP 462 41.895 47.231 23.917 1.00 0.00 C

ATOM 3643 CG TRP 462 43.138 46.373 23.812 1.00 0.00 C

ATOM 3644 CD1 TRP 462 43.258 45.013 23.800 1.00 0.00 C

ATOM 3645 CD2 TRP 462 44.470 46.898 23.697 1.00 0.00 C

ATOM 3646 CE2 TRP 462 45.340 45.809 23.624 1.00 0.00 C

ATOM 3647 CE3 TRP 462 44.931 48.182 23.656 1.00 0.00 C

ATOM 3648 NE1 TRP 462 44.582 44.658 23.689 1.00 0.00 N

ATOM 3649 CZ2 TRP 462 46.689 45.990 23.511 1.00 0.00 C

ATOM 3650 CZ3 TRP 462 46.292 48.362 23.540 1.00 0.00 C

ATOM 3651 CH2 TRP 462 47.153 47.286 23.470 1.00 0.00 C

ATOM 3652 N VAL 463 42.714 46.117 26.632 1.00 0.00 N

ATOM 3653 CA VAL 463 43.186 44.931 27.285 1.00 0.00 C

ATOM 3654 C VAL 463 42.096 43.941 27.133 1.00 0.00 C

ATOM 3655 O VAL 463 41.450 43.494 28.080 1.00 0.00 O

ATOM 3656 CB VAL 463 43.495 45.143 28.739 1.00 0.00 C

ATOM 3657 CG1 VAL 463 42.257 45.753 29.413 1.00 0.00 C

ATOM 3658 CG2 VAL 463 43.943 43.805 29.351 1.00 0.00 C

ATOM 3659 N MET 464 41.872 43.635 25.844 1.00 0.00 N

ATOM 3660 CA MET 464 40.828 42.809 25.334 1.00 0.00 C

ATOM 3661 C MET 464 41.539 41.634 24.789 1.00 0.00 C

ATOM 3662 O MET 464 42.594 41.793 24.179 1.00 0.00 O

ATOM 3663 CB MET 464 40.187 43.458 24.090 1.00 0.00 C

ATOM 3664 CG MET 464 38.717 43.157 23.804 1.00 0.00 C

ATOM 3665 SD MET 464 37.570 44.252 24.694 1.00 0.00 S

ATOM 3666 CE MET 464 36.077 43.594 23.897 1.00 0.00 C

ATOM 3667 N SER 465 40.988 40.427 24.991 1.00 0.00 N

ATOM 3668 CA SER 465 41.659 39.292 24.436 1.00 0.00 C

ATOM 3669 C SER 465 41.094 39.093 23.074 1.00 0.00 C

ATOM 3670 O SER 465 39.885 38.939 22.905 1.00 0.00 O

ATOM 3671 CB SER 465 41.432 37.991 25.226 1.00 0.00 C

ATOM 3672 OG SER 465 42.129 36.919 24.609 1.00 0.00 O

ATOM 3673 N MET 466 41.966 39.094 22.050 1.00 0.00 N

ATOM 3674 CA MET 466 41.431 38.948 20.734 1.00 0.00 C

ATOM 3675 C MET 466 41.795 37.601 20.195 1.00 0.00 C

ATOM 3676 O MET 466 41.937 37.413 18.989 1.00 0.00 O

ATOM 3677 CB MET 466 41.985 39.990 19.747 1.00 0.00 C

ATOM 3678 CG MET 466 41.572 41.424 20.082 1.00 0.00 C

ATOM 3679 SD MET 466 39.790 41.747 19.920 1.00 0.00 S

ATOM 3680 CE MET 466 39.798 41.736 18.104 1.00 0.00 C

ATOM 3681 N ASP 467 41.899 36.590 21.062 1.00 0.00 N

ATOM 3682 CA ASP 467 42.167 35.291 20.531 1.00 0.00 C

ATOM 3683 C ASP 467 40.817 34.821 20.111 1.00 0.00 C

ATOM 3684 O ASP 467 39.984 35.608 19.669 1.00 0.00 O

ATOM 3685 CB ASP 467 42.731 34.307 21.571 1.00 0.00 C

ATOM 3686 CG ASP 467 43.276 33.096 20.826 1.00 0.00 C

ATOM 3687 OD1 ASP 467 43.058 33.018 19.587 1.00 0.00 O

ATOM 3688 OD2 ASP 467 43.925 32.237 21.482 1.00 0.00 O

ATOM 3689 N TYR 468 40.555 33.515 20.194 1.00 0.00 N

ATOM 3690 CA TYR 468 39.232 33.100 19.846 1.00 0.00 C

ATOM 3691 C TYR 468 38.670 32.499 21.090 1.00 0.00 C

ATOM 3692 O TYR 468 39.415 32.068 21.966 1.00 0.00 O

ATOM 3693 CB TYR 468 39.176 32.035 18.739 1.00 0.00 C

ATOM 3694 CG TYR 468 37.749 31.922 18.320 1.00 0.00 C

ATOM 3695 CD1 TYR 468 37.236 32.775 17.370 1.00 0.00 C

ATOM 3696 CD2 TYR 468 36.923 30.975 18.874 1.00 0.00 C

ATOM 3697 CE1 TYR 468 35.922 32.681 16.978 1.00 0.00 C

ATOM 3698 CE2 TYR 468 35.608 30.873 18.488 1.00 0.00 C

ATOM 3699 CZ TYR 468 35.105 31.728 17.537 1.00 0.00 C

ATOM 3700 OH TYR 468 33.756 31.628 17.137 1.00 0.00 O

ATOM 3701 N PHE 469 37.330 32.500 21.221 1.00 0.00 N

ATOM 3702 CA PHE 469 36.726 31.984 22.414 1.00 0.00 C

ATOM 3703 C PHE 469 37.093 30.541 22.529 1.00 0.00 C

ATOM 3704 O PHE 469 37.552 30.089 23.577 1.00 0.00 O

ATOM 3705 CB PHE 469 35.191 32.089 22.382 1.00 0.00 C

ATOM 3706 CG PHE 469 34.651 31.536 23.656 1.00 0.00 C

ATOM 3707 CD1 PHE 469 34.674 32.293 24.805 1.00 0.00 C

ATOM 3708 CD2 PHE 469 34.110 30.272 23.702 1.00 0.00 C

ATOM 3709 CE1 PHE 469 34.174 31.794 25.985 1.00 0.00 C

ATOM 3710 CE2 PHE 469 33.608 29.769 24.879 1.00 0.00 C

ATOM 3711 CZ PHE 469 33.641 30.529 26.023 1.00 0.00 C

ATOM 3712 N ARG 470 36.922 29.780 21.434 1.00 0.00 N

ATOM 3713 CA ARG 470 37.236 28.383 21.467 1.00 0.00 C

ATOM 3714 C ARG 470 38.706 28.259 21.678 1.00 0.00 C

ATOM 3715 O ARG 470 39.170 27.382 22.404 1.00 0.00 O

ATOM 3716 CB ARG 470 36.908 27.638 20.160 1.00 0.00 C

ATOM 3717 CG ARG 470 37.695 28.132 18.944 1.00 0.00 C

ATOM 3718 CD ARG 470 37.390 27.357 17.661 1.00 0.00 C

ATOM 3719 NE ARG 470 37.883 25.963 17.854 1.00 0.00 N

ATOM 3720 CZ ARG 470 37.575 24.995 16.943 1.00 0.00 C

ATOM 3721 NH1 ARG 470 36.809 25.300 15.854 1.00 0.00 N

ATOM 3722 NH2 ARG 470 38.032 23.721 17.120 1.00 0.00 N

ATOM 3723 N ASP 471 39.475 29.160 21.043 1.00 0.00 N

ATOM 3724 CA ASP 471 40.902 29.085 21.102 1.00 0.00 C

ATOM 3725 C ASP 471 41.348 29.224 22.521 1.00 0.00 C

ATOM 3726 O ASP 471 42.199 28.461 22.976 1.00 0.00 O

ATOM 3727 CB ASP 471 41.585 30.192 20.280 1.00 0.00 C

ATOM 3728 CG ASP 471 43.064 29.856 20.149 1.00 0.00 C

ATOM 3729 OD1 ASP 471 43.720 29.608 21.196 1.00 0.00 O

ATOM 3730 OD2 ASP 471 43.561 29.852 18.991 1.00 0.00 O

ATOM 3731 N VAL 472 40.786 30.187 23.279 1.00 0.00 N

ATOM 3732 CA VAL 472 41.291 30.308 24.614 1.00 0.00 C

ATOM 3733 C VAL 472 40.431 29.482 25.513 1.00 0.00 C

ATOM 3734 O VAL 472 39.727 29.996 26.378 1.00 0.00 O

ATOM 3735 CB VAL 472 41.267 31.721 25.123 1.00 0.00 C

ATOM 3736 CG1 VAL 472 41.824 31.750 26.558 1.00 0.00 C

ATOM 3737 CG2 VAL 472 42.047 32.605 24.136 1.00 0.00 C

ATOM 3738 N LYS 473 40.484 28.153 25.340 1.00 0.00 N

ATOM 3739 CA LYS 473 39.735 27.274 26.184 1.00 0.00 C

ATOM 3740 C LYS 473 40.338 27.308 27.552 1.00 0.00 C

ATOM 3741 O LYS 473 39.623 27.229 28.544 1.00 0.00 O

ATOM 3742 CB LYS 473 39.718 25.822 25.688 1.00 0.00 C

ATOM 3743 CG LYS 473 38.808 25.610 24.478 1.00 0.00 C

ATOM 3744 CD LYS 473 37.337 25.931 24.749 1.00 0.00 C

ATOM 3745 CE LYS 473 37.009 27.422 24.665 1.00 0.00 C

ATOM 3746 NZ LYS 473 35.543 27.611 24.655 1.00 0.00 N

ATOM 3747 N LEU 474 41.678 27.419 27.646 1.00 0.00 N

ATOM 3748 CA LEU 474 42.326 27.471 28.927 1.00 0.00 C

ATOM 3749 C LEU 474 42.226 26.120 29.560 1.00 0.00 C

ATOM 3750 O LEU 474 42.676 25.917 30.687 1.00 0.00 O

ATOM 3751 CB LEU 474 41.672 28.498 29.865 1.00 0.00 C

ATOM 3752 CG LEU 474 41.729 29.938 29.319 1.00 0.00 C

ATOM 3753 CD1 LEU 474 41.113 30.938 30.310 1.00 0.00 C

ATOM 3754 CD2 LEU 474 43.153 30.320 28.885 1.00 0.00 C

ATOM 3755 N LEU 475 41.644 25.146 28.838 1.00 0.00 N

ATOM 3756 CA LEU 475 41.579 23.809 29.351 1.00 0.00 C

ATOM 3757 C LEU 475 42.970 23.270 29.388 1.00 0.00 C

ATOM 3758 O LEU 475 43.350 22.556 30.315 1.00 0.00 O

ATOM 3759 CB LEU 475 40.716 22.861 28.500 1.00 0.00 C

ATOM 3760 CG LEU 475 39.203 23.129 28.623 1.00 0.00 C

ATOM 3761 CD1 LEU 475 38.694 22.789 30.033 1.00 0.00 C

ATOM 3762 CD2 LEU 475 38.844 24.561 28.203 1.00 0.00 C

ATOM 3763 N ARG 476 43.772 23.618 28.367 1.00 0.00 N

ATOM 3764 CA ARG 476 45.109 23.115 28.263 1.00 0.00 C

ATOM 3765 C ARG 476 45.889 23.568 29.453 1.00 0.00 C

ATOM 3766 O ARG 476 46.650 22.792 30.029 1.00 0.00 O

ATOM 3767 CB ARG 476 45.842 23.627 27.011 1.00 0.00 C

ATOM 3768 CG ARG 476 47.273 23.101 26.885 1.00 0.00 C

ATOM 3769 CD ARG 476 48.006 23.613 25.643 1.00 0.00 C

ATOM 3770 NE ARG 476 48.257 25.068 25.845 1.00 0.00 N

ATOM 3771 CZ ARG 476 49.105 25.738 25.011 1.00 0.00 C

ATOM 3772 NH1 ARG 476 49.725 25.075 23.991 1.00 0.00 N

ATOM 3773 NH2 ARG 476 49.334 27.071 25.196 1.00 0.00 N

ATOM 3774 N ARG 477 45.716 24.836 29.870 1.00 0.00 N

ATOM 3775 CA ARG 477 46.499 25.311 30.973 1.00 0.00 C

ATOM 3776 C ARG 477 46.149 24.496 32.177 1.00 0.00 C

ATOM 3777 O ARG 477 47.028 24.086 32.933 1.00 0.00 O

ATOM 3778 CB ARG 477 46.272 26.803 31.301 1.00 0.00 C

ATOM 3779 CG ARG 477 44.863 27.164 31.779 1.00 0.00 C

ATOM 3780 CD ARG 477 44.632 26.886 33.268 1.00 0.00 C

ATOM 3781 NE ARG 477 44.962 28.128 34.021 1.00 0.00 N

ATOM 3782 CZ ARG 477 46.260 28.433 34.314 1.00 0.00 C

ATOM 3783 NH1 ARG 477 47.267 27.623 33.875 1.00 0.00 N

ATOM 3784 NH2 ARG 477 46.551 29.554 35.037 1.00 0.00 N

ATOM 3785 N GLY 478 44.847 24.221 32.378 1.00 0.00 N

ATOM 3786 CA GLY 478 44.435 23.470 33.527 1.00 0.00 C

ATOM 3787 C GLY 478 45.011 22.098 33.426 1.00 0.00 C

ATOM 3788 O GLY 478 45.463 21.526 34.416 1.00 0.00 O

ATOM 3789 N ALA 479 45.004 21.534 32.206 1.00 0.00 N

ATOM 3790 CA ALA 479 45.474 20.197 32.015 1.00 0.00 C

ATOM 3791 C ALA 479 46.919 20.130 32.387 1.00 0.00 C

ATOM 3792 O ALA 479 47.344 19.186 33.052 1.00 0.00 O

ATOM 3793 CB ALA 479 45.352 19.725 30.556 1.00 0.00 C

ATOM 3794 N VAL 480 47.721 21.136 31.984 1.00 0.00 N

ATOM 3795 CA VAL 480 49.115 21.030 32.295 1.00 0.00 C

ATOM 3796 C VAL 480 49.394 21.716 33.599 1.00 0.00 C

ATOM 3797 O VAL 480 50.225 22.619 33.683 1.00 0.00 O

ATOM 3798 CB VAL 480 49.994 21.628 31.227 1.00 0.00 C

ATOM 3799 CG1 VAL 480 49.645 23.117 31.054 1.00 0.00 C

ATOM 3800 CG2 VAL 480 51.465 21.356 31.588 1.00 0.00 C

ATOM 3801 N THR 481 48.723 21.255 34.670 1.00 0.00 N

ATOM 3802 CA THR 481 48.961 21.727 36.004 1.00 0.00 C

ATOM 3803 C THR 481 49.006 23.225 36.067 1.00 0.00 C

ATOM 3804 O THR 481 49.890 23.787 36.710 1.00 0.00 O

ATOM 3805 CB THR 481 50.236 21.198 36.593 1.00 0.00 C

ATOM 3806 CG2 THR 481 50.186 19.661 36.571 1.00 0.00 C

ATOM 3807 OG1 THR 481 51.351 21.661 35.845 1.00 0.00 O

ATOM 3808 N ASP 482 48.035 23.910 35.435 1.00 0.00 N

ATOM 3809 CA ASP 482 47.942 25.345 35.481 1.00 0.00 C

ATOM 3810 C ASP 482 49.258 26.002 35.195 1.00 0.00 C

ATOM 3811 O ASP 482 49.797 26.738 36.021 1.00 0.00 O

ATOM 3812 CB ASP 482 47.402 25.870 36.816 1.00 0.00 C

ATOM 3813 CG ASP 482 45.957 25.409 36.927 1.00 0.00 C

ATOM 3814 OD1 ASP 482 45.207 25.566 35.927 1.00 0.00 O

ATOM 3815 OD2 ASP 482 45.589 24.876 38.007 1.00 0.00 O

ATOM 3816 N LYS 483 49.797 25.770 33.985 1.00 0.00 N

ATOM 3817 CA LYS 483 51.049 26.352 33.606 1.00 0.00 C

ATOM 3818 C LYS 483 50.887 27.841 33.610 1.00 0.00 C

ATOM 3819 O LYS 483 49.789 28.359 33.409 1.00 0.00 O

ATOM 3820 CB LYS 483 51.506 25.913 32.204 1.00 0.00 C

ATOM 3821 CG LYS 483 52.913 26.373 31.826 1.00 0.00 C

ATOM 3822 CD LYS 483 53.476 25.635 30.610 1.00 0.00 C

ATOM 3823 CE LYS 483 54.884 26.082 30.216 1.00 0.00 C

ATOM 3824 NZ LYS 483 54.836 27.446 29.647 1.00 0.00 N

ATOM 3825 N CYS 484 52.008 28.557 33.834 1.00 0.00 N

ATOM 3826 CA CYS 484 52.059 29.991 33.918 1.00 0.00 C

ATOM 3827 C CYS 484 51.450 30.439 35.212 1.00 0.00 C

ATOM 3828 O CYS 484 51.340 31.635 35.478 1.00 0.00 O

ATOM 3829 CB CYS 484 51.318 30.681 32.759 1.00 0.00 C

ATOM 3830 SG CYS 484 51.538 32.486 32.750 1.00 0.00 S

ATOM 3831 N LEU 485 51.074 29.480 36.078 1.00 0.00 N

ATOM 3832 CA LEU 485 50.545 29.817 37.367 1.00 0.00 C

ATOM 3833 C LEU 485 51.481 29.194 38.350 1.00 0.00 C

ATOM 3834 O LEU 485 52.009 28.110 38.111 1.00 0.00 O

ATOM 3835 CB LEU 485 49.150 29.213 37.617 1.00 0.00 C

ATOM 3836 CG LEU 485 48.548 29.550 38.991 1.00 0.00 C

ATOM 3837 CD1 LEU 485 48.267 31.056 39.117 1.00 0.00 C

ATOM 3838 CD2 LEU 485 47.308 28.690 39.280 1.00 0.00 C

ATOM 3839 N LYS 486 51.746 29.881 39.473 1.00 0.00 N

ATOM 3840 CA LYS 486 52.610 29.321 40.464 1.00 0.00 C

ATOM 3841 C LYS 486 51.723 28.823 41.555 1.00 0.00 C

ATOM 3842 O LYS 486 50.870 29.557 42.055 1.00 0.00 O

ATOM 3843 CB LYS 486 53.579 30.340 41.085 1.00 0.00 C

ATOM 3844 CG LYS 486 54.607 30.891 40.095 1.00 0.00 C

ATOM 3845 CD LYS 486 55.336 32.135 40.607 1.00 0.00 C

ATOM 3846 CE LYS 486 56.248 31.859 41.804 1.00 0.00 C

ATOM 3847 NZ LYS 486 56.911 33.110 42.234 1.00 0.00 N

ATOM 3848 N PHE 487 51.898 27.543 41.938 1.00 0.00 N

ATOM 3849 CA PHE 487 51.105 26.956 42.977 1.00 0.00 C

ATOM 3850 C PHE 487 51.983 26.820 44.169 1.00 0.00 C

ATOM 3851 O PHE 487 53.090 26.293 44.077 1.00 0.00 O

ATOM 3852 CB PHE 487 50.641 25.525 42.665 1.00 0.00 C

ATOM 3853 CG PHE 487 49.578 25.577 41.629 1.00 0.00 C

ATOM 3854 CD1 PHE 487 49.883 25.806 40.308 1.00 0.00 C

ATOM 3855 CD2 PHE 487 48.269 25.372 41.991 1.00 0.00 C

ATOM 3856 CE1 PHE 487 48.882 25.845 39.367 1.00 0.00 C

ATOM 3857 CE2 PHE 487 47.264 25.409 41.057 1.00 0.00 C

ATOM 3858 CZ PHE 487 47.575 25.648 39.744 1.00 0.00 C

ATOM 3859 N THR 488 51.515 27.295 45.336 1.00 0.00 N

ATOM 3860 CA THR 488 52.349 27.152 46.487 1.00 0.00 C

ATOM 3861 C THR 488 51.506 26.722 47.636 1.00 0.00 C

ATOM 3862 O THR 488 50.304 26.979 47.675 1.00 0.00 O

ATOM 3863 CB THR 488 53.028 28.425 46.899 1.00 0.00 C

ATOM 3864 CG2 THR 488 53.898 28.922 45.732 1.00 0.00 C

ATOM 3865 OG1 THR 488 52.061 29.406 47.244 1.00 0.00 O

ATOM 3866 N ALA 489 52.123 26.013 48.599 1.00 0.00 N

ATOM 3867 CA ALA 489 51.392 25.678 49.780 1.00 0.00 C

ATOM 3868 C ALA 489 51.919 26.606 50.823 1.00 0.00 C

ATOM 3869 O ALA 489 53.095 26.539 51.173 1.00 0.00 O

ATOM 3870 CB ALA 489 51.636 24.242 50.273 1.00 0.00 C

ATOM 3871 N VAL 490 51.058 27.500 51.348 1.00 0.00 N

ATOM 3872 CA VAL 490 51.497 28.454 52.325 1.00 0.00 C

ATOM 3873 C VAL 490 51.097 27.893 53.687 1.00 0.00 C

ATOM 3874 O VAL 490 50.250 28.525 54.375 1.00 0.00 O

ATOM 3875 CB VAL 490 50.847 29.800 52.160 1.00 0.00 C

ATOM 3876 CG1 VAL 490 51.374 30.753 53.248 1.00 0.00 C

ATOM 3877 CG2 VAL 490 51.120 30.291 50.730 1.00 0.00 C

ATOM 3878 OXT VAL 490 51.639 26.816 54.055 1.00 0.00 O

ATOM 3879 P FAD 491 28.379 40.239 35.173 1.00 0.00 P

ATOM 3880 PA FAD 491 27.302 42.797 36.106 1.00 0.00 P

ATOM 3881 O1P FAD 491 27.920 39.377 36.177 1.00 0.00 O

ATOM 3882 O2P FAD 491 29.943 39.939 34.773 1.00 0.00 O

ATOM 3883 O3P FAD 491 28.514 41.799 35.633 1.00 0.00 O

ATOM 3884 O4P FAD 491 26.387 43.598 24.949 1.00 0.00 O

ATOM 3885 O5P FAD 491 27.748 43.277 24.500 1.00 0.00 O

ATOM 3886 C5' FAD 491 27.856 40.363 32.682 1.00 0.00 C

ATOM 3887 O5' FAD 491 27.422 40.212 33.887 1.00 0.00 O

ATOM 3888 C4' FAD 491 27.144 40.929 31.511 1.00 0.00 C

ATOM 3889 O4' FAD 491 27.335 42.372 32.800 1.00 0.00 O

ATOM 3890 C3' FAD 491 27.532 41.553 30.363 1.00 0.00 C

ATOM 3891 O3' FAD 491 26.849 40.640 29.218 1.00 0.00 O

ATOM 3892 C2' FAD 491 27.131 43.079 30.136 1.00 0.00 C

ATOM 3893 O2' FAD 491 27.770 44.029 30.989 1.00 0.00 O

ATOM 3894 C1' FAD 491 27.507 43.548 28.848 1.00 0.00 C

ATOM 3895 C1B FAD 491 28.032 44.521 41.655 1.00 0.00 C

ATOM 3896 N1 FAD 491 27.301 41.392 26.820 1.00 0.00 N

ATOM 3897 N1A FAD 491 30.251 45.192 46.283 1.00 0.00 N

ATOM 3898 O1A FAD 491 26.038 42.600 35.371 1.00 0.00 O

ATOM 3899 C2 FAD 491 27.400 40.478 25.794 1.00 0.00 C

ATOM 3900 C2A FAD 491 29.134 44.586 45.962 1.00 0.00 C

ATOM 3901 C2B FAD 491 27.185 45.404 40.786 1.00 0.00 C

ATOM 3902 O2 FAD 491 28.222 39.600 25.629 1.00 0.00 O

ATOM 3903 O2A FAD 491 27.931 44.315 35.946 1.00 0.00 O

ATOM 3904 O2B FAD 491 26.211 46.131 41.510 1.00 0.00 O

ATOM 3905 C3B FAD 491 26.561 44.441 39.832 1.00 0.00 C

ATOM 3906 N3 FAD 491 26.493 40.536 24.816 1.00 0.00 N

ATOM 3907 N3A FAD 491 28.652 44.477 44.729 1.00 0.00 N

ATOM 3908 O3B FAD 491 25.276 44.095 40.363 1.00 0.00 O

ATOM 3909 C4 FAD 491 25.589 41.614 24.773 1.00 0.00 C

ATOM 3910 C4A FAD 491 29.301 45.057 43.711 1.00 0.00 C

ATOM 3911 C4B FAD 491 27.429 43.255 39.873 1.00 0.00 C

ATOM 3912 C4X FAD 491 25.654 42.595 25.758 1.00 0.00 C

ATOM 3913 O4 FAD 491 24.559 41.392 23.998 1.00 0.00 O

ATOM 3914 O4B FAD 491 28.500 43.526 40.795 1.00 0.00 O

ATOM 3915 C5A FAD 491 30.510 45.781 44.003 1.00 0.00 C

ATOM 3916 C5B FAD 491 28.065 42.888 38.593 1.00 0.00 C

ATOM 3917 C5X FAD 491 24.474 44.577 26.709 1.00 0.00 C

ATOM 3918 N5 FAD 491 24.461 43.474 25.824 1.00 0.00 N

ATOM 3919 O5B FAD 491 27.091 42.493 37.653 1.00 0.00 O

ATOM 3920 C6 FAD 491 23.478 45.567 26.613 1.00 0.00 C

ATOM 3921 C6A FAD 491 30.960 45.807 45.402 1.00 0.00 C

ATOM 3922 N6A FAD 491 32.078 46.523 45.711 1.00 0.00 N

ATOM 3923 C7 FAD 491 23.521 46.638 27.463 1.00 0.00 C

ATOM 3924 C7M FAD 491 22.568 47.829 27.273 1.00 0.00 C

ATOM 3925 N7A FAD 491 30.997 46.198 42.857 1.00 0.00 N

ATOM 3926 C8 FAD 491 24.611 46.707 28.443 1.00 0.00 C

ATOM 3927 C8A FAD 491 30.168 45.794 41.889 1.00 0.00 C

ATOM 3928 C8M FAD 491 24.675 47.936 29.365 1.00 0.00 C

ATOM 3929 C9 FAD 491 25.532 45.645 28.611 1.00 0.00 C

ATOM 3930 C9A FAD 491 25.574 44.618 27.715 1.00 0.00 C

ATOM 3931 N9A FAD 491 29.112 45.117 42.412 1.00 0.00 N

ATOM 3932 C10 FAD 491 26.517 42.538 26.840 1.00 0.00 C

ATOM 3933 N10 FAD 491 26.440 43.527 27.835 1.00 0.00 N

ATOM 3934 P2B NAP 492 32.370 60.359 22.831 1.00 0.00 P

ATOM 3935 PA NAP 492 29.499 55.100 26.512 1.00 0.00 P

ATOM 3936 PN NAP 492 27.078 53.823 27.682 1.00 0.00 P

ATOM 3937 C1B NAP 492 29.339 60.790 25.241 1.00 0.00 C

ATOM 3938 C1D NAP 492 31.446 50.092 27.614 1.00 0.00 C

ATOM 3939 N1A NAP 492 29.920 65.456 27.133 1.00 0.00 N

ATOM 3940 N1N NAP 492 32.744 49.308 27.554 1.00 0.00 N

ATOM 3941 O1A NAP 492 29.699 53.976 25.582 1.00 0.00 O

ATOM 3942 O1N NAP 492 26.932 53.337 26.293 1.00 0.00 O

ATOM 3943 O1X NAP 492 32.460 59.194 21.960 1.00 0.00 O

ATOM 3944 C2A NAP 492 29.290 65.087 26.013 1.00 0.00 C

ATOM 3945 C2B NAP 492 30.348 59.865 24.556 1.00 0.00 C

ATOM 3946 C2D NAP 492 30.656 49.808 26.322 1.00 0.00 C

ATOM 3947 C2N NAP 492 32.986 48.175 28.364 1.00 0.00 C

ATOM 3948 O2A NAP 492 30.782 55.556 27.450 1.00 0.00 O

ATOM 3949 O2B NAP 492 30.878 60.569 23.443 1.00 0.00 O

ATOM 3950 O2D NAP 492 31.315 50.514 25.241 1.00 0.00 O

ATOM 3951 O2N NAP 492 25.958 54.550 28.345 1.00 0.00 O

ATOM 3952 O2X NAP 492 32.721 61.780 22.090 1.00 0.00 O

ATOM 3953 C3B NAP 492 29.397 58.781 24.053 1.00 0.00 C

ATOM 3954 C3D NAP 492 29.235 50.335 26.632 1.00 0.00 C

ATOM 3955 C3N NAP 492 34.198 47.460 28.227 1.00 0.00 C

ATOM 3956 N3A NAP 492 29.213 63.817 25.660 1.00 0.00 N

ATOM 3957 O3 NAP 492 28.296 54.944 27.679 1.00 0.00 O

ATOM 3958 O3B NAP 492 28.631 59.234 22.908 1.00 0.00 O

ATOM 3959 O3D NAP 492 28.753 51.366 25.708 1.00 0.00 O

ATOM 3960 O3X NAP 492 33.345 60.310 24.123 1.00 0.00 O

ATOM 3961 C4A NAP 492 29.801 62.859 26.409 1.00 0.00 C

ATOM 3962 C4B NAP 492 28.441 58.636 25.241 1.00 0.00 C

ATOM 3963 C4D NAP 492 29.384 50.926 28.056 1.00 0.00 C

ATOM 3964 C4N NAP 492 35.123 47.884 27.253 1.00 0.00 C

ATOM 3965 O4B NAP 492 28.345 59.947 25.844 1.00 0.00 O

ATOM 3966 O4D NAP 492 30.390 49.991 28.645 1.00 0.00 O

ATOM 3967 C5A NAP 492 30.483 63.213 27.560 1.00 0.00 C

ATOM 3968 C5B NAP 492 28.970 57.643 26.260 1.00 0.00 C

ATOM 3969 C5D NAP 492 27.934 51.145 28.640 1.00 0.00 C

ATOM 3970 C5N NAP 492 34.913 49.010 26.448 1.00 0.00 C

ATOM 3971 O5B NAP 492 29.213 56.394 25.602 1.00 0.00 O

ATOM 3972 O5D NAP 492 27.599 52.616 28.768 1.00 0.00 O

ATOM 3973 C6A NAP 492 30.528 64.546 27.913 1.00 0.00 C

ATOM 3974 C6N NAP 492 33.730 49.717 26.598 1.00 0.00 C

ATOM 3975 N6A NAP 492 31.159 64.902 29.010 1.00 0.00 N

ATOM 3976 C7N NAP 492 34.485 46.216 29.150 1.00 0.00 C

ATOM 3977 N7A NAP 492 30.955 62.110 28.110 1.00 0.00 N

ATOM 3978 N7N NAP 492 33.547 45.929 30.030 1.00 0.00 N

ATOM 3979 O7N NAP 492 35.540 45.590 29.147 1.00 0.00 O

ATOM 3980 C8A NAP 492 30.614 61.080 27.326 1.00 0.00 C

ATOM 3981 N9A NAP 492 29.908 61.546 26.300 1.00 0.00 N

END
